# Research on Effect and Mechanism of Xuefu Zhuyu Decoction on CHD Based on Meta-Analysis and Network Pharmacology

**DOI:** 10.1155/2021/9473531

**Published:** 2021-02-13

**Authors:** Fuguang Kui, Wenwen Gu, Fan Gao, Yuji Niu, Wenwen Li, Yaru Zhang, Lijuan Guo, Junru Wang, Zhenzhen Guo, Shihong Cen, Gangjun Du

**Affiliations:** ^1^Institute of Pharmacy, Pharmaceutical College of Henan University, Jinming District, Kaifeng, Henan 475004, China; ^2^School of Pharmacy and Chemical Engineering, Zhengzhou University of Industry Technology, Xinzheng, Henan 451150, China

## Abstract

Xuefu Zhuyu Decoction (XFZY) is an ancient compound widely used in the treatment of coronary heart disease. However, its efficacy evaluation is not complete and its mechanism of action is not clear enough. In an attempt to address these problems, the efficacy was evaluated by meta-analysis and the mechanism was elucidated by the network pharmacology method. We systematically searched relevant studies in PubMed, Chinese National Knowledge Infrastructure Database (CNKI), Cochrane Library, Wanfang Data, and other databases from 2007 to 2019. The association between XFZY treatment and CHD was estimated by risk ratio (RR) and corresponding 95% confidence intervals (95% CIs). The compounds and the potential protein targets of XFZY were obtained from TCMSP, and active compounds were selected according to their oral bioavailability and drug similarity. The potential genes of coronary heart disease were obtained from TTD, OMIM, and GeneCards. The potential pathways related to genes were determined by GO and KEGG pathway enrichment analyses. The compound-target and compound-target-pathway networks were constructed. Molecular docking validates the component and the target. A total of 21 studies including 1844 patients were enrolled in the present meta-analysis, indicating that XFZY has a greater beneficial on total effect (fixed effect RR = 1.30; 95% Cl: 1.24–1.36; *P*=0.82; *I*^2^ = 0.0%) and electrocardiogram efficacy (fixed effect RR = 1.40; 95% Cl: 1.26–1.56; *P*=0.96; *I*^2^ = 0.0%) compared with the control group. A total of 1342 components in XFZY were obtained, among which, 241 were chosen as bioactive components. GO and KEGG analyses got top 10 significantly enriched terms and 10 enriched pathways. The C-T network included 192 compounds and 3085 targets, whereas the C-T-P network included 10 compounds, 109 targets, and 5 pathways. There was a good binding activity between the components and the targets. XFZY has the curative effect on coronary heart disease, and its mechanism is related to 10 compounds, 10 core targets, and 5 pathways.

## 1. Introduction

Coronary heart disease (CHD) is a leading cause of death and disability worldwide [1]. CHD is caused by narrowing or obstruction of blood vessels due to coronary atherosclerosis, myocardial ischemia, hypoxia, and necrosis. CHD has intervened effectively with the advances in cardiovascular medicine over the past decades. Despite great improvements in cardiovascular medicine, these developments now still generate to post-treat sequelae. Therefore, finding a more effective therapy method is an urgent need.

Traditional Chinese medicine (TCM), embracing centuries of knowledge and wisdom, is a medical practice [2] and has played a significant therapeutic role in various diseases [3]. Xuefu Zhuyu Decoction (XFZYD), from “Yiling Gaicuo,” is Chinese herbal formulas commonly used to treat hypertension and cardiovascular diseases in traditional Chinese medicine [4]. It consists of eleven herbs, namely, *Paeonia lactiflora* Pall (Chi Shao in Chinese); *Ligusticum chuanxiong* Hort (Chuan Xiong in Chinese); *Bupleurum chinensie* DC (Chai Hu in Chinese); *Carthamus tinctorius* L. (Hong Hua in Chinese); *Angelica sinensis* (Oliv) Diels (Dang Gui in Chinese); *Prunus persica* (L.) Batsch (Tao Ren in Chinese); *Achyranthes bidentata* BI (Niu Xi in Chinese); *Glycyrrhiza uralensis* Fisch (Gan Cao in Chinese); *Platycodon grandifloras* (Jacq.) A. DC (Jie Geng in Chinese); *Citrus aurantium* L. (Zhi Ke in Chinese); *Rehmannia glutinosa* Libosch (Sheng Di Huang in Chinese). According to clinical view of TCM, Qi is the commander of blood. Qi stagnation causes blood stasis, which leads to CHD [5]. Studies have shown that XFZY is regulating Qi and promoting blood circulation [6]. Clinical studies have shown that XFZY is effectively ameliorative to the clinical symptoms of CHD without side effect [7]. The therapeutic effects of XFZY on atherosclerosis and hyperlipidemia were validated [8]. XFZY will improve the phlegm and blood stasis pattern in CHD [9]. XFZY is very effective in the treatment myocardial fibrosis, atherosclerosis, hypertension, unstable angina pectoris, and myocardial ischemia-reperfusion injury [10]. However, these studies are not supported by a large amount of clinical data, or incomplete. Therefore, this paper will use meta-analysis to evaluate the efficacy of XFZY on CHD through the study of a large number of clinical data. Currently, network pharmacology has shown that for complex diseases, Chinese medicine formulas have the advantages of multitarget interventions and minimal side effect [11]. Therefore, this study will also perform network pharmacology to elucidate the mechanism of XFZY on CHD.

## 2. Methods

The reporting of this study was guided by the Preferred Reporting Items for Systematic Reviews and Meta-Analyses checklist. The registration application for the systematic review protocol has been submitted at PROSPERO, but the registration number has not been obtained.

### 2.1. Literature Search

We searched the main literature database at home and abroad, namely, PubMed, PubMed Pro, Embase, Chinese Scientific Journal Database (VIP), SinoMed, Cochrane Library, CNKI, and Wanfang Data, from 2007 to 2019, identifying available studies to be included in the meta-analysis. The keywords that were used are as follows: Xuefu Zhuyu Tang, Xuefu Zhuyu Decoction, XFZY, XFZYD, coronary heart disease, and CHD. Besides, we also screened all other possible reference lists from the studies selected to identify further relevant studies and reviews (see Supplementary File 1 for further information).

### 2.2. Criteria for Literature Inclusion

The inclusion criteria were as follows:

1. Included studies were required to have enrolled patients with a clear diagnosis of coronary heart disease; no restrictions on race, age, or sex were imposed
2. Randomized, clinical trial
3. Control group for routine treatment and experimental group for XFZY on the basis of the control group
4. Basis of disease diagnosis is Nomenclature and Diagnostic Criteria for Ischemic Heart Disease and Guiding Principles for Clinical Research of New Chinese Medicines (trial)
5. The course of treatment is four weeks or more
6. It conforms to the ethical and moral treatment standard
7. The patient has no other mental illness or serious primary disease
8. The patient is not lactation, pregnancy, or advanced stage of disease
9. One or more outcome indicators of the following must be involved: (1) total therapeutic effect; (2) ECG; (3) adverse reaction; (4) angina pectoris attack frequency and duration; (5) the total effect of TCM syndromes; (6) serum lipids index; (7) blood stream change; (8) the effect of electroacupuncturing acupoint; (9) vessel endothelial function and factors of vessel endothelium; (10) LVEF and PAF; (11) average hospital stay; (12) disappeared time of angina; (13) effects of decreased dose; (14) symptom improvement; (15) the total angina pectoris efficacy.

Therefore, exclusion criteria included the following:

1. Republished studies
2. Nonrandomized trials
3. Experiments on animals
4. Review
5. There are no clear diagnostic criteria or not meet inclusion criteria
6. Lack of required data for meta-analysis
7. Treatment duration is less than four weeks
8. There is no complete evaluation of efficacy

### 2.3. Data Extraction

Two authors searched the literature according to the established strategy, then extracted data from the included studies, and compared the results independently. Discrepancy was resolved by the third author's adjudication to avoid bias. Extracted data included author, year of publication, title of the study, diagnostic criteria ,sample size, course of treatment, intervention and control measures, evaluation standard, random scheme generation, allocation hiding, blind method, incomplete result data, selective reporting, and other biases.

### 2.4. Quality Assessment

The Cochrane Collaboration's Bias Risk Assessment Tool was used to assess the quality of the literature. The main assessment areas are as follows: allocation concealment, random sequence generation, incomplete outcome data, blinding method for patients/researchers and outcomes assessors, selective reporting, and other sources of bias. The results were judged as “low risk,” “high risk,” and “unclear.”

### 2.5. Statistical Analysis

Statistical analyses were conducted using RevMan5.3 (Review Manager 5.3). Between studies, heterogeneity of each study was assessed using *I*^2^ tests. If high heterogeneity (*I*^2^ > 50%) was observed, random effect models were applied; otherwise, fixed effect models were used. Pooled RR with corresponding 95% CIs was used to evaluate the effect of XFZY on CHD. We used a funnel plot to determine potential publication bias.

### 2.6. Active Ingredients

Compounds of ten ingredients in XFZY were gathered from TCM Systems Pharmacology Database (TCMSP, a unique systems pharmacology platform designed for herbal medicines). Compounds of Sheng Di Huang were obtained from Chemistry Database (http://www.organchem.csdb.cn/). The ADME (absorption, distribution, metabolism, and excretion) was used to filtrating active compounds. The active components were obtained by screening for both oral bioavailability (OB) value ≥30% and drug similarity (DL) value ≥0.18.

### 2.7. Potential Target Identification

The targets of the active ingredients in XFZY were obtained from the TCMSP database and Chemistry Database (http://www.organchem.csdb.cn/).

### 2.8. CHD-Related Gene Targets

Retrieving the Therapeutic Target Database (TTD, http://db.idrblab.net/ttd/), Online Mendelian Inheritance in Man (OMIM https://www.omim.org/), and GeneCards (https://www.genecards.org/) about CHD, CHD-related gene targets were collected.

### 2.9. Gene Ontology (GO) and KEGG Pathway Enrichment

To seek out the biological characteristics of bioactive targets of XFZY on CHD in detail, the GO and KEGG pathway enrichment analyses of bioactive targets were conducted via DAVID 6.8 (https://david.ncifcrf.gov/). The top 10 significantly enriched terms and 10 significantly enriched pathways of XFZY on CHD were visualization.

### 2.10. Network Construction and Analysis

To further characterize the mechanism of XFZY on CHD, the C-T and C-T-P networks were constructed via Cytoscape 3.2.1. In these networks, compounds, targets, and pathways were expressed as nodes, whereas the C-T and C-T-P interactions were expressed as edges.

### 2.11. Molecular Docking

The bioactive component and the target with the highest degree value were selected for molecular docking. Mol2 format of molecular structure of bioactive components was obtained from the TCMSP database, and PDB format of 3D molecular structure of corresponding target genes was obtained from the RCSB PDB database (https://www.rcsb.org/). LeDock software was used to dock the bioactive components with the target to obtain the docking affinity. SwissDock (http://www.swissdock.ch/) and Chimera 1.14 software were used to perform molecular docking visualization analysis of compounds with high docking affinity and stable conformation with target proteins.

## 3. Results

### 3.1. Search Results and Study Quality

**Figure 1:**
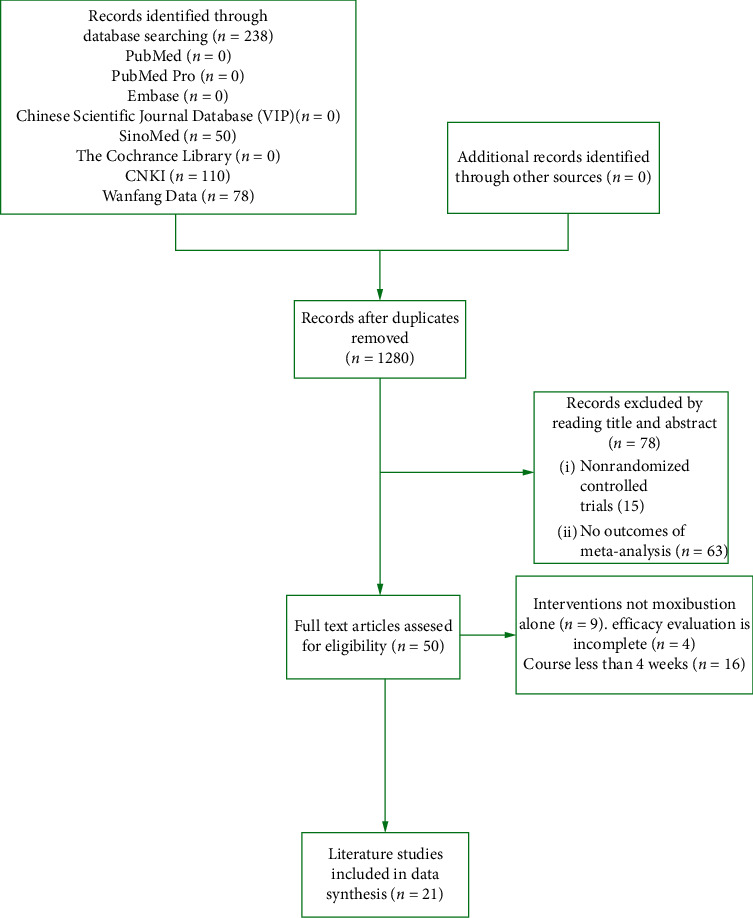
The flowchart of the study selection and exclusion criteria.

**Figure 2:**
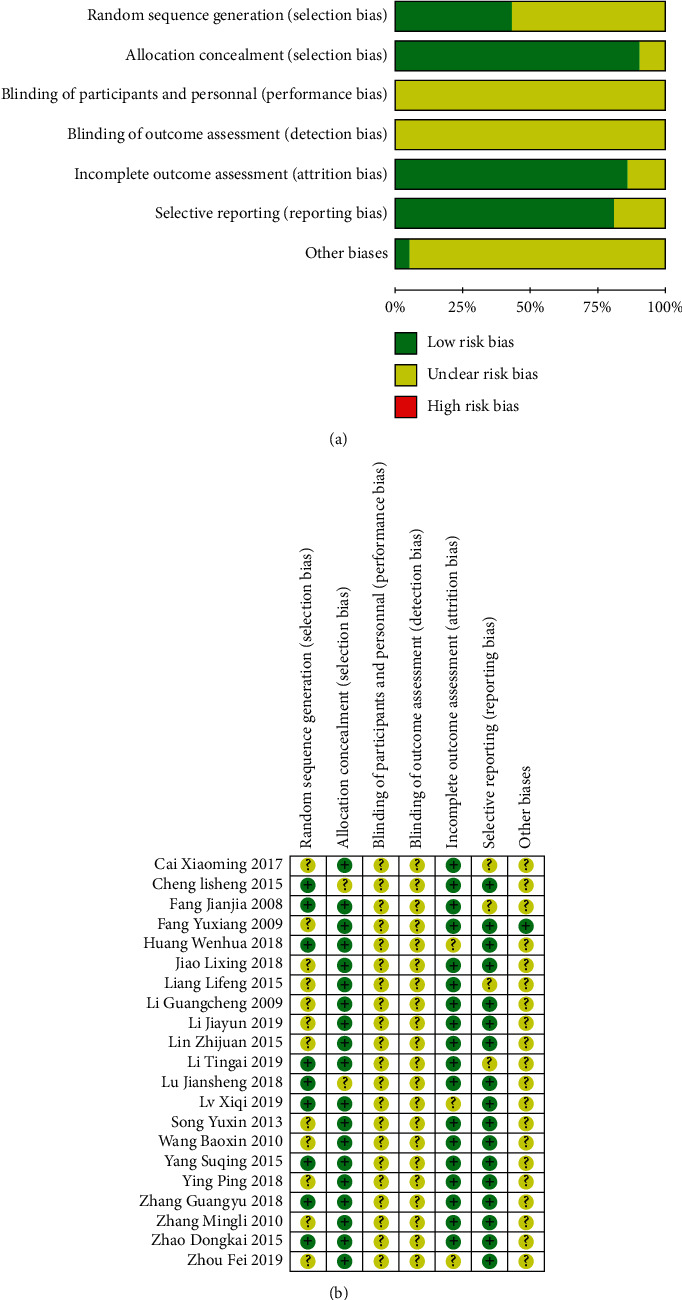
Risk bias graph of studies.

Figure 1 schematically shows the selection procedure for eligible articles. 238 references were obtained by searching all databases, among them, 50 papers from SinoMed, 110 papers from CNKI, and 78 papers from Wanfang Data. Removed duplicates, a total of 128 papers were identified in the initial search. However, 107 articles were excluded due to irrelevant content or not up to inclusion criteria after reading the titles and abstracts (shown in Supplementary File 4). After evaluation, 21 papers qualified for detailed evaluation. A total of 1844 patients from 21 included studies, 919 control groups and 925 treatment groups, were included in this meta-analysis. The detail data extracted from these included articles are listed in Table 1. Quality assessment of 21 available studies was performed by using the Risk Assessment Tool of the Cochrane Library. The risk of inclusion literature is shown in Figure 2.

### 3.2. Heterogeneity Detection and Pooled Analysis

**Figure 3:**
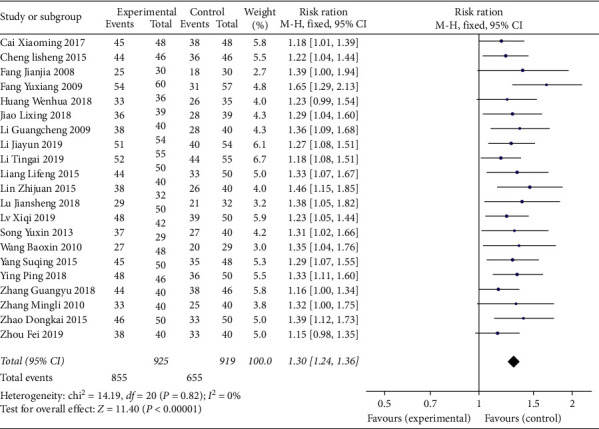
Comparison of total effect between the experimental group and the control group for coronary heart disease.

**Figure 4:**
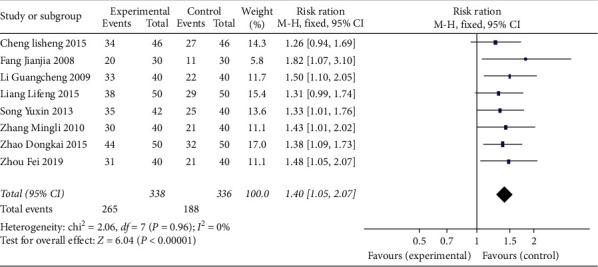
Comparison electrocardiogram efficacy between the experimental group and the control group for coronary heart disease.

We included 21 RCTs with a total of 1844 participants. All the 21 studies, including a total of 1844 patients, reported total effect of XFZY on CHD. We used a fixed effect model to analyze results because heterogeneity was low (*I*^2^ < 50%). Meta-analysis showed that patients with XFZY had more effective than control group (Figure 3) (fixed effect RR = 1.30; 95% Cl: 1.24–1.36; *P*=0.82; *I*^2^ = 0.0%). 8 studies, including a total of 674 patients, reported electrocardiogram efficacy of XFZY on CHD. We used a fixed effect model to analyze results. Meta-analysis showed that, compared with the control group, the experimental group with XFZY was more effective than the control group (Figure 4) (fixed effect RR = 1.40; 95% Cl: 1.26–1.56; *P*=0.96; *I*^2^ = 0.0%).

### 3.3. Publication Bias

**Figure 5:**
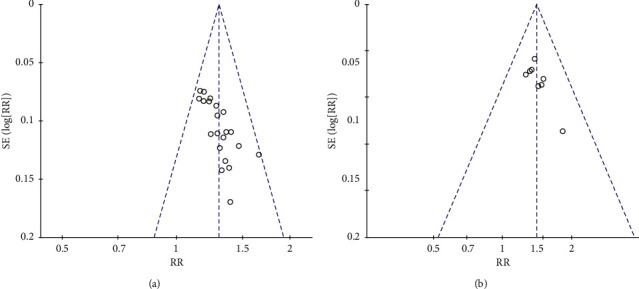
(a) The bias of the total effect in the treatment of coronary heart disease by Xuefu Zhuyu Decoction; (b) the bias of electrocardiogram efficacy in the treatment of coronary heart disease by Xuefu Zhuyu Decoction.

Funnel plots were made to evaluate publication bias. The result indicated that no clear bias in the total effect (Figure 5(a)**)** and electrocardiogram efficacy (Figure 5(b)).

### 3.4. Potential Active Moieties of XFZY

A total of 1342 chemical moieties obtained from the XFZY formula were collected from TCMSP. By ADME filtering, only 222 compounds met the principle of both oral bioavailability (OB) value ≥30% and drug similarity (DL) value ≥0.18. The number of potential active compounds from CS, CX, CH, HH, DG, TR, NX, GC, JG, and ZK was 27, 7, 17, 22, 2, 23, 20, 92, and 7, 5, respectively. 19 chemical moieties of SD were collected from the Chemistry Database (shown in Supplementary File 2). The compounds were chosen as potential active moieties for further analyses.

### 3.5. Putative Targets for the Active Compounds of XFZY

Putative targets corresponding to 241 components were obtained from TCMSP and Chemistry Database. Removing duplicates, 163 protein targets were obtained for further analyses (shown in Supplementary File 3).

### 3.6. Targets of Coronary Heart Disease

In total, 6785 targets of CHD were obtained from TTD, OMIM, and GeneCards (shown in Supplementary File 5).

### 3.7. GO and Pathway Enrichment Analysis

**Figure 6:**
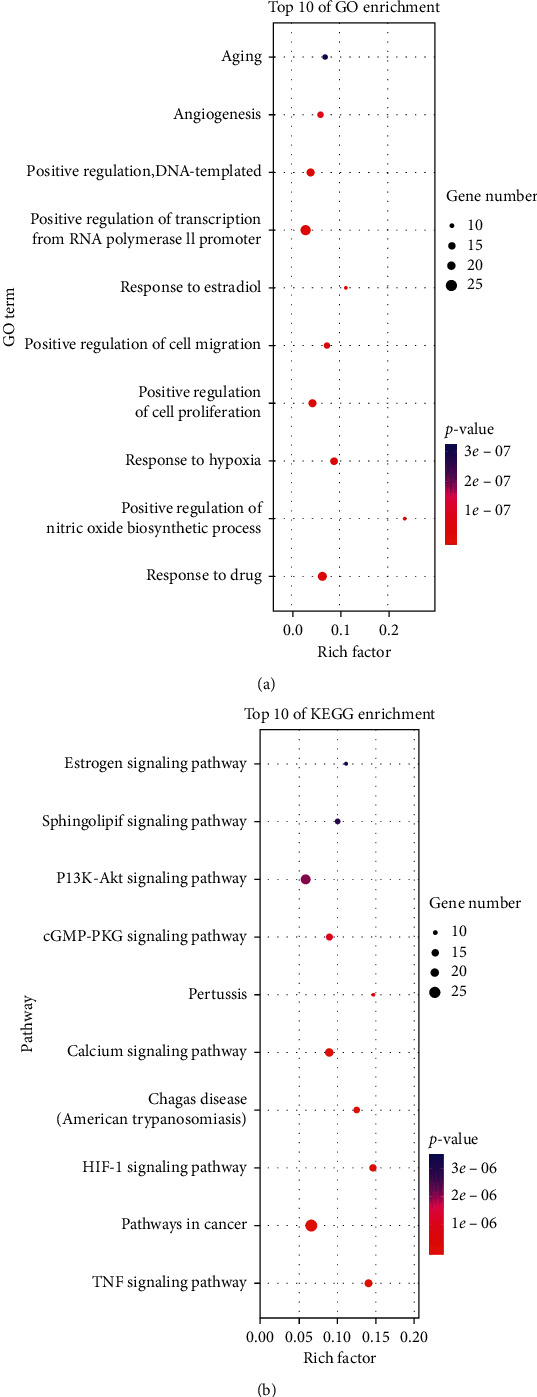
The 10 most significance of gene ontology (a) and pathway enrichment (b) analysis of therapy target genes of XFZY on CHD.

DAVID 5.6 was used to perform the GO and KEGG pathway enrichment of XFZY for CHD. The top 10 significantly enriched terms are shown (Figure 6(a)), which exerted its therapeutic effects on CHD involving “response to drug,” “positive regulation of nitric oxide biosynthetic process,” “response to hypoxia,” “positive regulation of cell proliferation,” “positive regulation of cell migration,” “response to estradiol,” “positive regulation of transcription,” “angiogenesis,” and “aging biological process.” The top 10 significantly enriched pathways of XFZY on CHD are shown (Figure 6(b)).

### 3.8. Network Construction and Analysis

#### 3.8.1. Compound-Target Network (C-T Network)

**Figure 7:**
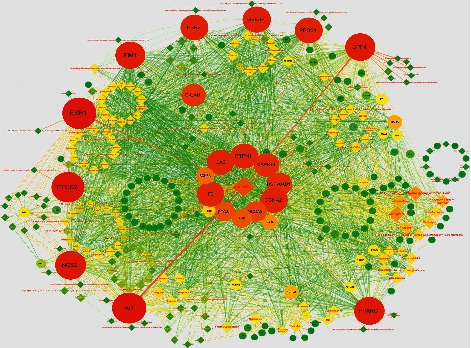
The compound-target network of XFZY on CHD. The circles represent potential protein targets, and the rhombus represents bioactive compounds. The edges represent the interactions between them, and nodes' sizes are proportional to their degree.

We performed bioactive target identification via WebGestalt (http://webgestalt.org/). There were 124 protein targets, which were the intersection of two parts, the targets of active compounds, and the targets of CHD, defined as bioactive targets for XFZY treating CHD. There were 192 corresponding compounds of bioactive targets. The compound-target network was constructed via Cytoscape3.2.1. The nodes represent compounds, targets, and edges which represent their interaction (Figure 7). The node color reflects the degree value. The edge thickness reflects the correlation between nodes. The result suggested that the top 10 compounds are as follows: quercetin; 7-methoxy-2-methyl isoflavone; (2R)-7-hydroxy-2-(4-hydroxyphenyl)chroman-4-one; beta-sitosterol; kaempferol; luteolin; medicarpin; formononetin; shinpterocarpin; 2-[(3R)-8,8-dimehyl-3,4-dihydro-2H-pyrano[6,5-f]chromen-3-yl]-5-mehoxyphenol. It means that these compounds may play a major role in the therapeutic effects. The top 10 targets are as follows: AR, ESR1, PTGS2, NOS2, PPARG, PIM1, DPP4, GSK3B, PRSS1, and ESR2. These targets may be the main targets of drug action. In addition, it can be seen that an effective component corresponds to multiple targets, and a target corresponds to multiple effective components.

#### 3.8.2. Compound-Target-Pathway Network (C-T-P Network)

**Figure 8:**
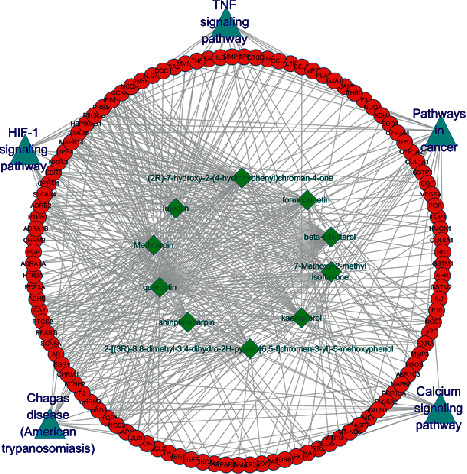
The compound-target-pathway network of XFZY on CHD. The read circles represent potential protein targets, the green rhombuses represent bioactive compounds, and the blue triangles represent signaling pathways. The edges represent the interactions between them.

To further characterize the molecular mechanism by which XFZY on CHD, a compound-target-pathway network was performed based on all involved compounds, targets, and their corresponding significant signal pathways. Therefore, five signal pathways that may be related to CHD were selected from the top 10 KEGG genes in the treatment of CHD by XFZY, constructing the component-target-pathway network (Figure 8). The five signal pathways are follows: TNF signaling pathway, pathways in cancer, calcium signaling pathway, HIF-1 signaling pathway, and Chagas disease (American trypanosomiasis), respectively.

### 3.9. Molecular Docking

**Figure 9:**
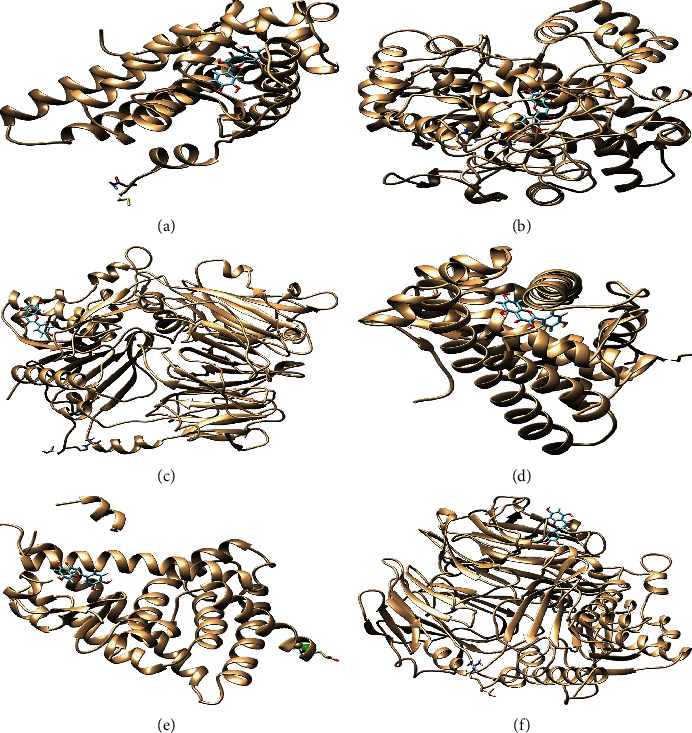
Pattern diagram of molecular docking: (a) quercetin-AR; (b) quercetin-PTGS2; (c) kaempferol-DPP4; (d) kaempferol1-AR; (e) luteolin-PPARG; (f) luteolin-DPP4.

For gaining docking affinity, LeDock software was used to dock quercetin; 7-methoxy-2-methyl isoflavone; (2R)-7-hydroxy-2-(4-hydroxyphenyl) chroman-4-one; beta-sitosterol; kaempferol, and other XFZY bioactive components with AR, ESR1, PTGS2, NOS2, and other targets, respectively (Table 2). The affinity of compounds and targets is greater than −4.25 kcal/mol, which indicates that the compound had a certain affinity for the protein crystal structure. Quercetin and AR, quercetin and PTGS2, kaempferol and DPP4, kaempferol and AR, luteolin and PPARG, and luteolin and DPP4 were selected for visualization with Chimera 1.14 software and SwissDock (http://www.swissdock.ch/) (Figure 9).

## 4. Discussion

TCM has obvious advantages in treating many diseases, but it also has many problems. For example, on the one hand, the clinical efficacy evaluation is incomplete or lacks a large amount of clinical data support. To solve the problem, meta-analysis was performed. Meta-analysis is a systematic, quantitative, and comprehensive statistical method based on previous research results. It is often used to evaluate intervention randomized controlled trials in evidence-based medicine, which can objectively evaluate the clinical efficacy. Therefore, in this article, we compiled the current evidence on the total effect of XFZY on CHD in 1,844 individuals from 21 studies, and electrocardiogram efficacy of XFZY on CHD in 674 individuals from 8 studies. This evidence objectively suggested that XFZY has a sound clinical effect on CHD and there are no significant adverse reactions.

In clinical practice, blood lipids are usually used as an indicator to evaluate the curative effect. Of course, it assuredly is too limited to evaluate the effectiveness and safety of CHD in terms of total effect and electrocardiogram efficacy. Unfortunately, inclusion criteria have no other enough indicators to draw forest plot.

On the other hand, the composition of TCM is complex and the mechanism of action has not been effectively elaborated, which are the biggest bottleneck in the development and popularization of TCM. Hence, we used network pharmacology to identify major components and elucidate mechanisms of action. Network pharmacology comprehends disease as an interconnected complex biological network and cognizes the mechanisms of TCM action via network topology [33]. It is a promising approach to expound the mechanisms of TCM [34]. The results of C-T and C-T-P networks showed that quercetin; 7-methoxy-2-methyl isoflavone; (2R)-7-hydroxy-2-(4-hydroxyphenyl)chroman-4-one; beta-sitosterol; kaempferol; luteolin; medicarpin; formononetin; and shinpterocarpin; 2-[(3R)-8,8-dimehyl-3,4-dihydro-2H-pyrano[6,5-f]chromen-3-yl-5-mehoxyphenol are the main ingredients at work according degree from large to small when XFZY plays curative effect and that the main targets are as follows: AR, ESR1, PTGS2, NOS2, PPARG, PIM1, DPP4, GSK3B, PRSS1, and ESR2, which mainly involves the following five signaling pathways, namely, TNF signaling pathway, pathways in cancer, calcium signaling pathway, HIF-1 signaling pathway, and Chagas disease (American trypanosomiasis).

According to the TCM prescription principles of monarch, minister, assistant, and guide, *Prunus persica* (L.) *Batsch* and *Carthamus tinctorius* L. are the monarch drug, *Achyranthes bidentata* BI, *Ligusticum chuanxiong* Hort, and *Paeonia lactiflora* Pall are the minister drug, *Rehmannia glutinosa* Libosch, *Angelica sinensis* (Oliv) Diels, Citrus aurantium L., and *Platycodon grandifloras* (Jacq.) A. DC are the assistant drug, and *Glycyrrhiza uralensis* Fisch is the guide drug. *Carthamus tinctorius* L. and *Prunus persica* (L.) Batsch can activate blood and dissolve stasis. *Paeonia lactiflora* Pall, *Ligusticum chuanxiong* Hort, and *Achyranthes bidentata* BI promote stasis metabolism and excretion. *Rehmannia glutinosa* Libosch, *Platycodon grandifloras* (Jacq.) A. DC, *Citrus aurantium* L., and *Angelica sinensis* (Oliv) Diels clear heat and activate blood, promoting circulation of Qi and blood. *Glycyrrhiza uralensis* Fisch harmonizes other medicines. In the supplemental document, we find main ingredients as follows: quercetin which originates from *Bupleurum chinensie* DC, *Glycyrrhiza uralensis* Fisch, *Achyranthes bidentata* BI, and *Carthamus tinctorius* L.; 7-methoxy-2-methyl isoflavone which originates from *Glycyrrhiza uralensis* Fisch; (2R)-7-hydroxy-2-(4-hydroxyphenyl)chroman-4-one which originates from *Glycyrrhiza uralensis* Fisch; beta-sitosterol which originates from *Paeonia lactiflora* Pall, *Carthamus tinctorius* L., *Angelica sinensis* (Oliv) Diels, *Citrus aurantium* L., *Achyranthes bidentata* BI, and *Prunus persica* (L.) Batsch; kaempferol which originates from *Bupleurum chinensie* DC, *Carthamus tinctorius* L., *Glycyrrhiza uralensis* Fisch, and *Achyranthes bidentata* BI; luteolin which originates from *Carthamus tinctorius* L. and *Platycodon grandifloras* (Jacq.) A. DC; medicarpin which originates from *Glycyrrhiza uralensis* Fisch; formononetin which originates from *Glycyrrhiza uralensis* Fisch; shinpterocarpin which originates from *Glycyrrhiza uralensis* Fisch; 2-[(3R)-8,8-dimehyl-3,4-dihydro-2H-pyrano[6,5-f]chromen-3-yl-5-mehoxyphenol which originates from Glycyrrhiza uralensis Fisch.

Quercetin is an important flavonoid that has been normally viewed as a great antioxidant and anti-inflammatory molecule [35–37] and can prevent endothelial dysfunction and myocardial ischemia [38]. Beta-sitosterol exerted protective actions against myocardial injury [39] and is used in the treatment of hypercholesterolemia [40]. Kaempferol has antioxidant, anti-inflammatory effect [41] and protects against cardiac hypertrophy [42]. Luteolin can improve ventricular function and reduce coronary reperfusion [43] and thrombotic tendency [44]. Medicarpin and its metabolites were transported in most organs of the body [45] and can promote the publication of other ingredients. Formononetin substantially attenuates the generation of atherosclerosis [46]. 7-methoxy-2-methyl isoflavone; (2R)-7-hydroxy-2-(4-hydroxyphenyl)chroman-4-one; shinpterocarpin; and 2-[(3R)-8,8-dimehyl-3,4-dihydro-2H-pyrano[6,5-f]chromen-3-yl-5-mehoxyphenol can reduce the incidence of ischemia reperfusion injuries and chronic inflammatory disease. It is well known that reducing inflammatory factor can attenuate the development of cardiac hypertrophy possibly [47]. The above ten compounds can adjust a variety of physiological activities for the treatment of coronary heart disease. The literature confirms the predicted results of network pharmacology. Generally speaking, the action of traditional Chinese medicine is consistent with the pharmacological action of its components.

The molecular docking results showed that the active components were well bound to target proteins. The affinity of quercetin and AR is −6.90 kcal/mol. The affinity of quercetin and PTGS2 is -7.07 kcal/mol. The affinity of kaempferol and DPP4 is −5.96 kcal/mol. The affinity of kaempferol1 and AR is −6.77 kcal/mol. The affinity of luteolin and PPARG is −5.75 kcal/mol. The affinity of luteolin and DPP4 is −6.01 kcal/mol.

Androgens work by binding to androgen receptors. Androgen is a key early event in atherosclerosis [48]. ESR1 and ESR2 mediate estrogen action. Estrogen protects against atherosclerosis [49]. PTGS2 has been linked to atherosclerosis, stroke, and other CVDs [50]. NOS2 is a subtype of NOS, which can affect the composition of NO. NO bioavailability plays a vital role in the pathophysiology of cardiovascular disease [51]. Peroxisome proliferator-activated receptor gamma (PPARG) is involved in the transcription of atherosclerosis and related diseases [52]. PIM1 has cardioprotective action [53]. The loss of DPP4 activity may affect the antithrombogenic nature [54]. Manipulating GSK-3beta is a promising strategy for myocardial protection in coronary artery disease and heart failure [55].PRSS1 is mainly involved in proteolysis and digestion.

In addition to pathways in cancer and Chagas disease (American trypanosomiasis), the pathway of Xuefu Zhuyu Decoction in the treatment of CHD is also involved in the following signaling pathways: TNF signaling pathway, calcium signaling pathway, and HIF-1 signaling pathway. TNF-*α* increasing ROS levels and decreasing nitric oxide production in blood vessels leads to endothelial dysfunction. It contributes also to the development of atherosclerotic plaques [56]. Dysregulation of these Ca^2+^ fluxes will lead to different heart and vascular pathologies [57]. HIF plays an essential role in the complex progression of atherosclerosis [58].

According to the literature, it is not difficult to find that the targets of network pharmacology are related to the regulation of various physiological functions such as reducing inflammatory response, sex hormone regulation, NO regulation, and calcium ion regulation. Furthermore, the pathways are mainly involving calcium regulation, arteriosclerosis development, and other biological functions.

These results will provide a reliable quality evaluation standard for the treatment of CHD by Xuefu Zhuyu Decoction. The existing TCM quality evaluation system needs more effective supplement to control the quality of compound prescription general [59]. Usually, content-based quality control is a commonly adopted method at present, which may not ensure biological activity in vivo or cannot be absorbed effectually. Network pharmacology can intuitively reflect the importance of components according degree. When evaluating the quality of traditional Chinese medicinal materials, the ingredients in the C-T network with high degree value should be considered first, rather than those with high content. For XFZY treat CHD, ten compounds, quercetin; 7-methoxy-2-methyl isoflavone; (2R)-7-hydroxy-2-(4-hydroxyphenyl)chroman-4-one; beta-sitosterol; kaempferol; luteolin; medicarpin; formononetin; shinpterocarpin; and 2-[(3R)-8,8-dimehyl-3,4- dihydro-2H-pyrano[6,5-f]chromen-3-yl-5-mehoxyphenol, are suggested to selected as marker compounds for quality control. According to the importance of ingredients in the treatment of diseases, we can establish a quality control system more in line with the rules of TCM medication.

## 5. Conclusion

In summary, XFZY has the curative effect on coronary heart disease and its mechanism is related to 10 compounds, 10 core targets, and 5 pathways. This study may provide a novel strategy for the understanding of TCM mechanism and make a positive contribution to the standardization of quality control. However, there may still be some potential shortcomings in this study. For example, this study lacks further experiment and more clinical sample.

## Figures and Tables

**Table 1:** The characteristics of all included studies.

Author and reference	Year	Total sample	Control/treatment	Period of treatment (day)	Drug		Evaluation standard
					C	T	
Li Jiayun [12]	2019	108	54/54	60	(1), (4), (6)	(11)	1, 3, 9
Lu Jiansheng [13]	2018	64	32/32	60	(1), (3), (6)	(11)	1, 3, 7, 8, 11
Jiao Lixing [14]	2015	78	39/39	30	(1), (10)	(11)	1, 3, 8, 12
Zhao Dongkai [15]	2015	100	50/50	30	(14)	(11)	1, 2, 4
Huang Wenhua [16]	2018	71	35/36	30	(1), (2)	(11)	1, 4
Li Tingai [17]	2019	110	55/55	30	(1), (2), (3)③	(11)	1, 5
Fang Yuxiang [18]	2009	117	57/60	28	(1), (2)②	(11)	1, 7, 8, 13
Ying Ping [19]	2018	100	50/50	60	(1), (3), (4)	(11)	1, 6, 7
Wang Baoxin [20]	2010	58	29/29	30	(1), (8)	(11)	1, 4
Fang Jianjia [21]	2008	60	30/30	28	(2), (3)	(11)	1, 2, 14
Lin Zhijuan [22]	2015	70	30/40	30	(1), (2), (3), (9), (13)	(11)	2, 6, 15
Song Yuxin [23]	2013	82	40/42	28	(1), (6), (7), (9)	(11)	1, 2, 5, 7
Lv Xiqi [24]	2019	100	50/50	60	(1), (3), (5)	(11)	1, 7
Li Guangcheng [25]	2009	80	40/40	30	(1), (2)	(11)	1, 2, 5
Yang Suqing [26]	2015	96	48/48	28	(1), (2), (3)	(11)	1, 3, 5
Zhang Guangyu [27]	2018	92	46/46	28	(1), (3), (8)	(11)	1, 3, 4
Liang Lifeng [28]	2015	100	50/50	28	(6), (7), (9)	(11)	1, 2, 7
Cai Xiaoming [29]	2017	96	48/48	28	(6), (7), (9)	(11)	1
Zhou Fei [30]	2019	80	40/40	28	(6), (7)	(11)	1, 2, 6
Zhang Mingli [31]	2010	80	40/40	28	(2), (7), (9)	(11)	1, 2, 7
Cheng Lisheng [32]	2015	92	46/46	28	(3), (4), (10)	(11)	1, 2, 4, 7

Abbreviations: (1) aspirin; (2) nitroglycerin; (3) isosorbide dinitrate/isosorbide mononitrate; (4) metoprolol; (5) nifedipine; (6) nitrates; (7) *β*-blocker; (8) Betocloc; (9) calcium antagonist; (10) captopril; (11) Xuefu Zhuyu Decoction based on the control group; (12) lipid-lowering agents and pain relief drugs; (13) clopidogrel and atorvastatin; (14) Compound Salvia Tablets. 1 Total therapeutic effect; 2 ECG; 3 adverse reaction; 4 angina pectoris attack frequency and duration; 5 the total effective of TCM syndromes; 6 serum lipid index; 7 blood stream change; 8 the effect of electroacupuncturing acupoint; 9 vessel endothelial function and factors of vessel endothelium; 10 LVEF and PAF; 11 average hospital stay; 12 disappeared time of angina; 13 effects of decreased dose; 14 symptom improvement; 15 the total angina pectoris efficacy.

**Table 2:** Molecular docking of bioactive ingredients of XFZY and corresponding targets.

Ingredient	Target name	PDB ID	Affinity (kcal/mol)
Quercetin	AR	2pir	−6.90
Quercetin	PTGS2	5f1a	−7.07
Quercetin	P1M1	2o3p	−6.27
Quercetin	NOS2	4ux6	−6.72
7-Methoxy-2-methyl isoflavone	PTGS2	5f1a	−4.88
(2R)-7-Hydroxy-2-(4-hydroxyphenyl)chroman-4-one	P1M1	2o3p	−4.59
Kaempferol	DPP4	5t4f	−5.96
Kaempferol	AR	2pir	−6.77
Kaempferol	ESR1	2i0j	−6.09
Kaempferol	PPARG	6c5t	−5.97
Luteolin	PPARG	2pob	−5.75
Luteolin	AR	2pir	−6.74
Luteolin	DPP4	5t4f	−6.01
Medicarpin	ESR1	2i0j	−4.89
Formononetin	PRSS1	1trn	−5.08
Shinpterocarpin	PPARG	6c5t	−4.53
2-[(3R)-8,8-Dimethyl-3,4-dihydro-2H-pyrano[6,5-f]chromen-3-yl]-5-methoxyphenol	DPP4	5y7k	−4.95

## Data Availability

The data used to support the findings of the study are available from the corresponding author upon request

## Supplementary Materials

Supplementary Materials Supplementary File 1: a detailed description of the methods and results of literature retrieval. Supplementary File 2: 241 compounds of XFZY, from the TCMSP database, met the principle of both oral bioavailability (OB) value ≥30% and drug similarity (DL) value ≥0.18. The number of potential active compounds from CS (*Paeonia lactiflora* Pall), CX (*Ligusticum chuanxiong* Hort), CH (*Bupleurum chinensie* DC), HH (*Carthamus tinctorius* L.), DG (*Angelica sinensis* (Oliv) Diels), TR (*Prunus persica* (L.) Batsch), NX (*Achyranthes bidentata* BI), GC (*Glycyrrhiza uralensis* Fisch), JG (*Platycodon grandifloras* (Jacq.) A. DC), and ZK (*Citrus aurantium* L.) was 27, 7, 17, 22, 2, 23, 20, 92, 7, and 5, respectively. 19 chemical moieties of SD (Rehmannia glutinosa Libosch) were collected from the Chemistry Database. Supplementary File 3 Protein targets of 241 corresponding components were obtained from TCMSP and Chemistry Database. Supplementary File 4: literature exclusion reasons. Supplementary File 5: 6785 targets of CHD were obtained from TTD, OMIM, and GeneCards.
