# Supplementary material for: Research on Effect and Mechanism of Xuefu Zhuyu Decoction on CHD Based on Meta-Analysis and Network Pharmacology
Source: Evid Based Complement Alternat Med. 2021 Feb 13;2021:9473531. doi: 10.1155/2021/9473531 (PMC7896852; doi:10.1155/2021/9473531)
Supplement: Supplementary Materials — Supplementary File 1: a detailed description of the methods and results of literature retrieval. Supplementary File 2: 241 compounds of XFZY, from the TCMSP database, met the principle of both oral bioavailability (OB) value ≥30% and drug similarity (DL) value ≥0.18. The number of potential active compounds from CS (Paeonia lactiflora Pall), CX (Ligusticum chuanxiong Hort), CH (Bupleurum chinensie DC), HH (Carthamus tinctorius L.), DG (Angelica sinensis (Oliv) Diels), TR (Prunus persica (L.) Batsch), NX (Achyranthes bidentata BI), GC (Glycyrrhiza uralensis Fisch), JG (Platycodon grandifloras (Jacq.) A. DC), and ZK (Citrus aurantium L.) was 27, 7, 17, 22, 2, 23, 20, 92, 7, and 5, respectively. 19 chemical moieties of SD (Rehmannia glutinosa Libosch) were collected from the Chemistry Database. Supplementary File 3 Protein targets of 241 corresponding components were obtained from TCMSP and Chemistry Database. Supplementary File 4: literature exclusion reasons. Supplementary File 5: 6785 targets of CHD were obtained from TTD, OMIM, and GeneCards. [file 9473531.f1.docx]

supplementary material(1)

**search strategy:**

We searched PubMed, PubMed Pro, Embase，Chinese Scientific Journal Database (VIP), SinoMed，The Cochrance Library, CNKI, Wanfang data, from 2007 to 2019, identifying available studies to be included in the meta-analysis. The keywords that were used are as follows: Xuefu Zhuyu tang, Xuefu Zhuyu Decoction，XFZY, XFZYD, coronary heart disease, CHD. Besides, we also screened all other possible reference lists from the studies selected to identify further relevant studies and reviews.

The results as follow:

PubMed=0

PubMed Pro=0

Embase=0

Chinese Scientific Journal Database (VIP)=0

SinoMed=50

The Cochrance Library=0

CNKI=110

Wanfang data=78

In the database, CNKI contains all the results of SinoMed. CNKI contains 60 results of Wanfang data. Last, we get 128 included criteria.

supplementary material(2)

**compounds for XFZY**

| Name | Active Ingredients | OB | DL | Source |
| --- | --- | --- | --- | --- |
| CS  (Paeonia lactiflora Pall) | beta-sitosterol | 36.91391 | 0.75123 | TCMSP |
|  | sitosterol | 36.91391 | 0.7512 | TCMSP |
|  | Stigmasterol | 43.82985 | 0.75665 | TCMSP |
|  | (+)-catechin | 54.82643 | 0.24164 | TCMSP |
|  | ellagic acid | 43.06456 | 0.43417 | TCMSP |
|  | paeoniflorgenone | 87.59312 | 0.36678 | TCMSP |
|  | Lactiflorin | 49.12132 | 0.79711 | TCMSP |
|  | paeoniflorin | 53.87038 | 0.78709 | TCMSP |
|  | paeoniflorin_qt | 68.17576 | 0.39507 | TCMSP |
|  | baicalein | 33.51892 | 0.20888 | TCMSP |
|  | Baicalin | 40.12361 | 0.75264 | TCMSP |
|  | Spinasterol | 42.97937 | 0.75534 | TCMSP |
|  | (1S,2S,4R)-trans-2-hydroxy-1,8-cineole-B-D-glucopyranoside | 30.25241 | 0.27464 | TCMSP |
|  | (2R,3R)-4-methoxyl-distylin | 59.98325 | 0.29949 | TCMSP |
|  | 1-o-beta-d-glucopyranosyl-8-o-benzoylpaeonisuffrone_qt | 36.01306 | 0.29897 | TCMSP |
|  | 1-o-beta-d-glucopyranosylpaeonisuffrone_qt | 65.08187 | 0.35391 | TCMSP |
|  | stigmast-7-en-3-ol | 37.42312 | 0.75088 | TCMSP |
|  | benzoyl paeoniflorin | 31.13867 | 0.54227 | TCMSP |
|  | Albiflorin | 30.24614 | 0.77038 | TCMSP |
|  | Albiflorin_qt | 48.70012 | 0.32628 | TCMSP |
|  | 4-ethyl-paeoniflorin_qt | 56.86958 | 0.44483 | TCMSP |
|  | 4-o-methyl-paeoniflorin_qt | 56.70352 | 0.42562 | TCMSP |
|  | 8-debenzoylpaeonidanin | 31.74315 | 0.45389 | TCMSP |
|  | Paeoniflorigenone | 65.33411 | 0.36711 | TCMSP |
|  | 9-ethyl-neo-paeoniaflorin A_qt | 64.41989 | 0.29598 | TCMSP |
|  | evofolinB | 64.73662 | 0.22232 | TCMSP |
|  | isobenzoylpaeoniflorin | 31.13867 | 0.54234 | TCMSP |
| CX  (Ligusticum chuanxiong Hort) | sitosterol | 36.91391 | 0.7512 | TCMSP |
|  | FA | 68.96044 | 0.7057 | TCMSP |
|  | Mandenol | 41.9962 | 0.19321 | TCMSP |
|  | Myricanone | 40.59757 | 0.51262 | TCMSP |
|  | Perlolyrine | 65.94775 | 0.2747 | TCMSP |
|  | senkyunone | 47.66395 | 0.24435 | TCMSP |
|  | wallichilide | 42.31068 | 0.70639 | TCMSP |
| CH  (Bupleurum chinensie DC) | quercetin | 46.43335 | 0.27525 | TCMSP |
|  | isorhamnetin | 49.60438 | 0.306 | TCMSP |
|  | kaempferol | 41.88225 | 0.24066 | TCMSP |
|  | Stigmasterol | 43.82985 | 0.75665 | TCMSP |
|  | petunidin | 30.04554 | 0.30712 | TCMSP |
|  | Linoleyl acetate | 42.10077 | 0.19845 | TCMSP |
|  | Baicalin | 40.12361 | 0.75264 | TCMSP |
|  | 3,5,6,7-tetramethoxy-2-(3,4,5-trimethoxyphenyl)chromone | 31.97496 | 0.59317 | TCMSP |
|  | Areapillin | 48.96435 | 0.41394 | TCMSP |
|  | Longikaurin A | 47.72215 | 0.53015 | TCMSP |
|  | Octalupine | 47.82225 | 0.27864 | TCMSP |
|  | Sainfuran | 79.90979 | 0.23331 | TCMSP |
|  | Troxerutin | 31.59657 | 0.28256 | TCMSP |
|  | (+)-Anomalin | 46.05534 | 0.6566 | TCMSP |
|  | saikosaponin c_qt | 30.50493 | 0.63193 | TCMSP |
|  | α-spinasterol | 42.97937 | 0.75693 | TCMSP |
|  | Cubebin | 57.12813 | 0.63988 | TCMSP |
| HH  (Carthamus tinctorius L) | luteolin | 36.16263 | 0.24552 | TCMSP |
|  | quercetin | 46.43335 | 0.27525 | TCMSP |
|  | beta-sitosterol | 36.91391 | 0.75123 | TCMSP |
|  | kaempferol | 41.88225 | 0.24066 | TCMSP |
|  | Stigmasterol | 43.82985 | 0.75665 | TCMSP |
|  | CLR | 37.8739 | 0.67677 | TCMSP |
|  | poriferast-5-en-3beta-ol | 36.91391 | 0.75034 | TCMSP |
|  | Flavoxanthin | 60.41294 | 0.55609 | TCMSP |
|  | 4-[(E)-4-(3,5-dimethoxy-4-oxo-1-cyclohexa-2,5-dienylidene)but-2-enylidene]-2,6-dimethoxycyclohexa-2,5-dien-1-one | 48.46631 | 0.36494 | TCMSP |
|  | lignan | 43.31816 | 0.65067 | TCMSP |
|  | lupeol-palmitate | 33.98365 | 0.31954 | TCMSP |
|  | Phytoene | 39.56307 | 0.50463 | TCMSP |
|  | phytofluene | 43.18173 | 0.50316 | TCMSP |
|  | Pyrethrin II | 48.35707 | 0.35025 | TCMSP |
|  | 6-Hydroxykaempferol | 62.13267 | 0.27266 | TCMSP |
|  | baicalein | 33.51892 | 0.20888 | TCMSP |
|  | qt_carthamone | 51.02582 | 0.20055 | TCMSP |
|  | 6-Hydroxynaringenin | 33.22921 | 0.24203 | TCMSP |
|  | quercetagetin | 45.00699 | 0.30991 | TCMSP |
|  | 7,8-dimethyl-1H-pyrimido[5,6-g]quinoxaline-2,4-dione | 45.75094 | 0.18605 | TCMSP |
|  | beta-carotene | 37.18433 | 0.58358 | TCMSP |
|  | Baicalin | 40.12361 | 0.75264 | TCMSP |
| DG  (Angelica sinensis（Oliv）Diels) | beta-sitosterol | 36.91391 | 0.75123 | TCMSP |
|  | Stigmasterol | 43.82985 | 0.75665 | TCMSP |
| TR  (Prunus persica（L.）Batsch) | hederagenin | 36.91391 | 0.75072 | TCMSP |
|  | beta-sitosterol | 36.91391 | 0.75123 | TCMSP |
|  | campesterol | 37.57682 | 0.71476 | TCMSP |
|  | Sitosterol alpha1 | 43.28127 | 0.78354 | TCMSP |
|  | 2,3-didehydro GA70 | 63.29363 | 0.49632 | TCMSP |
|  | 2,3-didehydro GA77 | 88.08055 | 0.53017 | TCMSP |
|  | GA119 | 76.36423 | 0.49382 | TCMSP |
|  | GA120 | 84.84964 | 0.45279 | TCMSP |
|  | GA121-isolactone | 72.69926 | 0.5371 | TCMSP |
|  | GA122 | 64.79329 | 0.49617 | TCMSP |
|  | GA122-isolactone | 88.11097 | 0.5371 | TCMSP |
|  | gibberellin 17 | 94.64115 | 0.49443 | TCMSP |
|  | 4a-formyl-7alpha-hydroxy-1-methyl-8-methylidene-4aalpha,4bbeta-gibbane-1alpha,10beta-dicarboxylic acid | 88.59516 | 0.46382 | TCMSP |
|  | GA30 | 61.71774 | 0.54002 | TCMSP |
|  | Gibberellin A44 | 101.6132 | 0.54105 | TCMSP |
|  | GA54 | 64.20665 | 0.5349 | TCMSP |
|  | GA60 | 93.16869 | 0.53004 | TCMSP |
|  | GA63 | 65.54356 | 0.53773 | TCMSP |
|  | gibberellin 7 | 73.80062 | 0.49609 | TCMSP |
|  | GA77 | 87.89416 | 0.52764 | TCMSP |
|  | GA87 | 68.85255 | 0.57188 | TCMSP |
|  | 3-O-p-coumaroylquinic acid | 37.6279 | 0.28636 | TCMSP |
|  | Populoside_qt | 108.8855 | 0.20476 | TCMSP |
| NX  (Achyranthes bidentata BI) | beta-daucosterol_qt | 36.91391 | 0.75382 | TCMSP |
|  | quercetin | 46.43335 | 0.27525 | TCMSP |
|  | wogonin | 30.68457 | 0.22942 | TCMSP |
|  | beta-sitosterol | 36.91391 | 0.75123 | TCMSP |
|  | kaempferol | 41.88225 | 0.24066 | TCMSP |
|  | Stigmasterol | 43.82985 | 0.75665 | TCMSP |
|  | palmatine | 64.60111 | 0.64524 | TCMSP |
|  | poriferasta-7,22E-dien-3beta-ol | 42.97937 | 0.75555 | TCMSP |
|  | berberine | 36.86125 | 0.77665 | TCMSP |
|  | coptisine | 30.67185 | 0.85647 | TCMSP |
|  | delta 7-stigmastenol | 37.42312 | 0.75103 | TCMSP |
|  | baicalein | 33.51892 | 0.20888 | TCMSP |
|  | Baicalin | 40.12361 | 0.75264 | TCMSP |
|  | epiberberine | 43.09233 | 0.7761 | TCMSP |
|  | Inophyllum E | 38.80967 | 0.85408 | TCMSP |
|  | Spinasterol | 42.97937 | 0.75534 | TCMSP |
|  | 28-norolean-17-en-3-ol | 35.93106 | 0.77805 | TCMSP |
|  | bidentatoside,ii_qt | 31.75996 | 0.58629 | TCMSP |
|  | Spinoside A | 41.75034 | 0.40276 | TCMSP |
|  | β-ecdysterone | 44.23031 | 0.82405 | TCMSP |
| GC  (Glycyrrhiza uralensis Fisch) | quercetin | 46.43335 | 0.27525 | TCMSP |
|  | Mairin | 55.37707 | 0.7761 | TCMSP |
|  | Jaranol | 50.82882 | 0.29148 | TCMSP |
|  | isorhamnetin | 49.60438 | 0.306 | TCMSP |
|  | sitosterol | 36.91391 | 0.7512 | TCMSP |
|  | formononetin | 69.67388 | 0.21202 | TCMSP |
|  | Calycosin | 47.75183 | 0.24278 | TCMSP |
|  | kaempferol | 41.88225 | 0.24066 | TCMSP |
|  | licochalcone a | 40.78965 | 0.28517 | TCMSP |
|  | Vestitol | 74.65519 | 0.20935 | TCMSP |
|  | Inermine | 75.18306 | 0.53754 | TCMSP |
|  | DFV | 32.76272 | 0.18316 | TCMSP |
|  | Glycyrol | 90.77578 | 0.66819 | TCMSP |
|  | Medicarpin | 49.21982 | 0.3351 | TCMSP |
|  | Lupiwighteone | 51.63569 | 0.36739 | TCMSP |
|  | 7-Methoxy-2-methyl isoflavone | 42.56474 | 0.19946 | TCMSP |
|  | naringenin | 59.2939 | 0.21128 | TCMSP |
|  | (2S)-2-[4-hydroxy-3-(3-methylbut-2-enyl)phenyl]-8,8-dimethyl-2,3-dihydropyrano[2,3-f]chromen-4-one | 31.78703 | 0.72403 | TCMSP |
|  | euchrenone | 30.28726 | 0.57386 | TCMSP |
|  | glyasperin B | 65.22439 | 0.43851 | TCMSP |
|  | glyasperin F | 75.8368 | 0.53514 | TCMSP |
|  | Glyasperin C | 45.56381 | 0.39947 | TCMSP |
|  | Isotrifoliol | 31.94479 | 0.42422 | TCMSP |
|  | (E)-1-(2,4-dihydroxyphenyl)-3-(2,2-dimethylchromen-6-yl)prop-2-en-1-one | 39.61686 | 0.35077 | TCMSP |
|  | kanzonols W | 50.48008 | 0.51704 | TCMSP |
|  | (2S)-6-(2,4-dihydroxyphenyl)-2-(2-hydroxypropan-2-yl)-4-methoxy-2,3-dihydrofuro[3,2-g]chromen-7-one | 60.25041 | 0.63433 | TCMSP |
|  | Semilicoisoflavone B | 48.77755 | 0.54732 | TCMSP |
|  | Glepidotin A | 44.72187 | 0.34685 | TCMSP |
|  | Glepidotin B | 64.46292 | 0.34485 | TCMSP |
|  | Phaseolinisoflavan | 32.00811 | 0.44538 | TCMSP |
|  | Glypallichalcone | 61.59706 | 0.18993 | TCMSP |
|  | 8-(6-hydroxy-2-benzofuranyl)-2,2-dimethyl-5-chromenol | 58.43728 | 0.38106 | TCMSP |
|  | Licochalcone B | 76.75735 | 0.1935 | TCMSP |
|  | licochalcone G | 49.25496 | 0.32325 | TCMSP |
|  | 3-(2,4-dihydroxyphenyl)-8-(1,1-dimethylprop-2-enyl)-7-hydroxy-5-methoxy-coumarin | 59.62247 | 0.42894 | TCMSP |
|  | Licoricone | 63.57846 | 0.4712 | TCMSP |
|  | Gancaonin A | 51.07519 | 0.40378 | TCMSP |
|  | Gancaonin B | 48.7944 | 0.44924 | TCMSP |
|  | licorice glycoside E | 32.88743 | 0.27218 | TCMSP |
|  | 3-(3,4-dihydroxyphenyl)-5,7-dihydroxy-8-(3-methylbut-2-enyl)chromone | 66.37125 | 0.41392 | TCMSP |
|  | 5,7-dihydroxy-3-(4-methoxyphenyl)-8-(3-methylbut-2-enyl)chromone | 30.48878 | 0.41002 | TCMSP |
|  | 2-(3,4-dihydroxyphenyl)-5,7-dihydroxy-6-(3-methylbut-2-enyl)chromone | 44.15196 | 0.41482 | TCMSP |
|  | Glycyrin | 52.60657 | 0.47466 | TCMSP |
|  | Licocoumarone | 33.21085 | 0.3568 | TCMSP |
|  | Licoisoflavone | 41.61022 | 0.41646 | TCMSP |
|  | Licoisoflavone B | 38.92871 | 0.54714 | TCMSP |
|  | licoisoflavanone | 52.46625 | 0.54488 | TCMSP |
|  | shinpterocarpin | 80.29528 | 0.72746 | TCMSP |
|  | (E)-3-[3,4-dihydroxy-5-(3-methylbut-2-enyl)phenyl]-1-(2,4-dihydroxyphenyl)prop-2-en-1-one | 46.26792 | 0.3062 | TCMSP |
|  | liquiritin | 65.69011 | 0.73893 | TCMSP |
|  | licopyranocoumarin | 80.36001 | 0.6535 | TCMSP |
|  | 3,22-Dihydroxy-11-oxo-delta(12)-oleanene-27-alpha-methoxycarbonyl-29-oic acid | 34.31942 | 0.54718 | TCMSP |
|  | Glyzaglabrin | 61.06889 | 0.35347 | TCMSP |
|  | Glabridin | 53.24514 | 0.46967 | TCMSP |
|  | Glabranin | 52.89566 | 0.31208 | TCMSP |
|  | Glabrene | 46.26686 | 0.43902 | TCMSP |
|  | Glabrone | 52.51217 | 0.49645 | TCMSP |
|  | 1,3-dihydroxy-9-methoxy-6-benzofurano[3,2-c]chromenone | 48.14154 | 0.42831 | TCMSP |
|  | 1,3-dihydroxy-8,9-dimethoxy-6-benzofurano[3,2-c]chromenone | 62.90135 | 0.52759 | TCMSP |
|  | Eurycarpin A | 43.27728 | 0.37429 | TCMSP |
|  | glycyroside | 37.25032 | 0.79156 | TCMSP |
|  | (-)-Medicocarpin | 40.99397 | 0.95059 | TCMSP |
|  | Sigmoidin-B | 34.88109 | 0.41455 | TCMSP |
|  | (2R)-7-hydroxy-2-(4-hydroxyphenyl)chroman-4-one | 71.12299 | 0.18303 | TCMSP |
|  | (2S)-7-hydroxy-2-(4-hydroxyphenyl)-8-(3-methylbut-2-enyl)chroman-4-one | 36.56537 | 0.32291 | TCMSP |
|  | Isoglycyrol | 44.69923 | 0.83845 | TCMSP |
|  | Isolicoflavonol | 45.16999 | 0.41859 | TCMSP |
|  | HMO | 38.36542 | 0.21067 | TCMSP |
|  | 1-Methoxyphaseollidin | 69.98098 | 0.63739 | TCMSP |
|  | Quercetin der. | 46.44939 | 0.3343 | TCMSP |
|  | 3'-Hydroxy-4'-O-Methylglabridin | 43.71495 | 0.57406 | TCMSP |
|  | 3'-Methoxyglabridin | 46.16151 | 0.57393 | TCMSP |
|  | 2-[(3R)-8,8-dimethyl-3,4-dihydro-2H-pyrano[6,5-f]chromen-3-yl]-5-methoxyphenol | 36.21429 | 0.52122 | TCMSP |
|  | Inflacoumarin A | 39.7091 | 0.32613 | TCMSP |
|  | icos-5-enoic acid | 30.70294 | 0.19725 | TCMSP |
|  | Kanzonol F | 32.46833 | 0.89364 | TCMSP |
|  | 6-prenylated eriodictyol | 39.22383 | 0.41259 | TCMSP |
|  | 7,2',4'-trihydroxy－5-methoxy-3－arylcoumarin | 83.71437 | 0.27136 | TCMSP |
|  | 7-Acetoxy-2-methylisoflavone | 38.92333 | 0.26217 | TCMSP |
|  | 8-prenylated eriodictyol | 53.79476 | 0.40383 | TCMSP |
|  | gadelaidic acid | 30.70294 | 0.19725 | TCMSP |
|  | Gancaonin G | 60.43521 | 0.39404 | TCMSP |
|  | Gancaonin H | 50.10372 | 0.78416 | TCMSP |
|  | Licoagrocarpin | 58.8139 | 0.58498 | TCMSP |
|  | Glyasperins M | 72.67081 | 0.59274 | TCMSP |
|  | Glycyrrhiza flavonol A | 41.27528 | 0.59512 | TCMSP |
|  | Licoagroisoflavone | 57.28224 | 0.48679 | TCMSP |
|  | 18α-hydroxyglycyrrhetic acid | 41.16139 | 0.7091 | TCMSP |
|  | Odoratin | 49.94822 | 0.30487 | TCMSP |
|  | Phaseol | 78.76622 | 0.57867 | TCMSP |
|  | Xambioona | 54.84916 | 0.87419 | TCMSP |
|  | dehydroglyasperins C | 53.82326 | 0.37006 | TCMSP |
| JG  (Platycodon grandifloras  (Jacq.)A.DC) | luteolin | 36.16263 | 0.24552 | TCMSP |
|  | acacetin | 34.97357 | 0.24082 | TCMSP |
|  | Spinasterol | 42.97937 | 0.75534 | TCMSP |
|  | cis-Dihydroquercetin | 66.437 | 0.27344 | TCMSP |
|  | 2-O-methyl-3―O-β-D-glucopyranosyl platycogenate A | 45.15023 | 0.25226 | TCMSP |
|  | dimethyl 2-O-methyl-3-O-a-D-glucopyranosyl platycogenate A | 39.20758 | 0.25368 | TCMSP |
|  | robinin | 39.84373 | 0.70731 | TCMSP |
| ZK  (Citrusaurantium L) | beta-sitosterol | 36.91391 | 0.75123 | TCMSP |
|  | Hesperetin | 70.31209 | 0.27252 | TCMSP |
|  | naringenin | 59.2939 | 0.21128 | TCMSP |
|  | nobiletin | 61.66944 | 0.51652 | TCMSP |
|  | Marmin | 38.22698 | 0.31358 | TCMSP |
| SD  (Rehmannia glutinosa Libosch) | behenic acid |  |  | Chemistry  Datebase |
|  | arachidic acid |  |  | Chemistry Datebase |
|  | pelargonic acid |  |  | Chemistry Datebase |
|  | rehmaionoside A |  |  | Chemistry Datebase |
|  | capric acid |  |  | Chemistry Datebase |
|  | isoacteoside |  |  | Chemistry Datebase |
|  | daucosterin |  |  | Chemistry Datebase |
|  | 1-O-β-D-glucopyranosyl-2-N-2'-hydroxypalmitoyl sphinga-4-trans,8-cis-dienine | | | Chemistry Datebase |
|  | phenyl acetic acid |  |  | Chemistry Datebase |
|  | monomelittoside |  |  | Chemistry Datebase |
|  | kusaginin |  |  | Chemistry Datebase |
|  | aucuboside |  |  | Chemistry Datebase |
|  | glucosamine |  |  | Chemistry Datebase |
|  | palmitoleic acid |  |  | Chemistry Datebase |
|  | jionoside A1 |  |  | Chemistry Datebase |
|  | rehmannioside D |  |  | Chemistry Datebase |
|  | rehmaionoside B |  |  | Chemistry Datebase |
|  | jionoside B1 |  |  | Chemistry Datebase |
|  | phosphoric acid |  |  | Chemistry Datebase |

supplementary material(3)

**compounds-target**

| Active Ingredients | Targets |
| --- | --- |
| beta-sitosterol | BCL2 |
| beta-sitosterol | PON1 |
| beta-sitosterol | JUN |
| beta-sitosterol | MAP1S |
| beta-sitosterol | NOS2 |
| beta-sitosterol | PTGS1 |
| beta-sitosterol | DRD1 |
| beta-sitosterol | CHRM3 |
| beta-sitosterol | F2 |
| beta-sitosterol | KCNH2 |
| beta-sitosterol | CHRM1 |
| beta-sitosterol | ESR1 |
| beta-sitosterol | AR |
| beta-sitosterol | SCN5A |
| beta-sitosterol | PPARG |
| beta-sitosterol | PTGS2 |
| beta-sitosterol | CA2 |
| beta-sitosterol | ACHE |
| beta-sitosterol | PDE3A |
| beta-sitosterol | HTR2A |
| beta-sitosterol | ADRA1A |
| beta-sitosterol | PGR |
| beta-sitosterol | CHRM2 |
| beta-sitosterol | ADRA1B |
| beta-sitosterol | PTPN1 |
| beta-sitosterol | ADRB2 |
| beta-sitosterol | SLC6A4 |
| beta-sitosterol | OPRM1 |
| beta-sitosterol | ESR2 |
| beta-sitosterol | NR3C1 |
| beta-sitosterol | DPP4 |
| beta-sitosterol | MAPK14 |
| beta-sitosterol | GSK3B |
| beta-sitosterol | HSP90AB1 |
| beta-sitosterol | PIK3CG |
| beta-sitosterol | PRSS1 |
| beta-sitosterol | PIM1 |
| beta-sitosterol | CCNA2 |
| beta-sitosterol | NCOA2 |
| sitosterol | ESR1 |
| sitosterol | AR |
| sitosterol | PGR |
| sitosterol | NR3C2 |
| sitosterol | NR3C1 |
| sitosterol | NCOA2 |
| Stigmasterol | NOS2 |
| Stigmasterol | PTGS1 |
| Stigmasterol | CHRM3 |
| Stigmasterol | F2 |
| Stigmasterol | CHRM1 |
| Stigmasterol | ESR1 |
| Stigmasterol | AR |
| Stigmasterol | ADRB1 |
| Stigmasterol | SCN5A |
| Stigmasterol | PPARG |
| Stigmasterol | PTGS2 |
| Stigmasterol | NOS3 |
| Stigmasterol | ADRA2A |
| Stigmasterol | CA2 |
| Stigmasterol | RXRA |
| Stigmasterol | ACHE |
| Stigmasterol | HTR2A |
| Stigmasterol | SLC6A2 |
| Stigmasterol | ADRA1A |
| Stigmasterol | ADRA1B |
| Stigmasterol | ADRB2 |
| Stigmasterol | DPP4 |
| Stigmasterol | PLAU |
| Stigmasterol | MAOB |
| Stigmasterol | ADH1C |
| Stigmasterol | PRSS1 |
| (+)-catechin | CAT |
| (+)-catechin | NOS2 |
| (+)-catechin | PTGS1 |
| (+)-catechin | PPARG |
| (+)-catechin | RXRA |
| (+)-catechin | PTPN1 |
| (+)-catechin | ESR2 |
| (+)-catechin | DPP4 |
| (+)-catechin | MAPK14 |
| (+)-catechin | GSK3B |
| (+)-catechin | HSP90AB1 |
| (+)-catechin | PIM1 |
| (+)-catechin | CCNA2 |
| (+)-catechin | NCOA2 |
| (+)-catechin | CALM1 |
| ellagic acid | MMP2 |
| ellagic acid | GSTP1 |
| ellagic acid | VEGFA |
| ellagic acid | GSTA1 |
| ellagic acid | GSTM1 |
| ellagic acid | GSTM2 |
| ellagic acid | ESR1 |
| ellagic acid | AR |
| ellagic acid | PGR |
| ellagic acid | HSP90AB1 |
| paeoniflorgenone | DPP4 |
| paeoniflorin | TNF |
| paeoniflorin | IL6 |
| baicalein | BCL2 |
| baicalein | PTGS2 |
| baicalein | MPO |
| baicalein | CDC2 |
| baicalein | TP53 |
| baicalein | VEGFA |
| baicalein | AHR |
| baicalein | EGLN1 |
| baicalein | CYC1 |
| baicalein | NOS2 |
| baicalein | PTGS1 |
| baicalein | ESR1 |
| baicalein | AR |
| baicalein | PPARG |
| baicalein | CA2 |
| baicalein | PDE3A |
| baicalein | PTPN1 |
| baicalein | ESR2 |
| baicalein | DPP4 |
| baicalein | MAPK14 |
| baicalein | GSK3B |
| baicalein | HSP90AB1 |
| baicalein | PIK3CG |
| baicalein | PRSS1 |
| baicalein | PIM1 |
| baicalein | CCNA2 |
| baicalein | NCOA2 |
| baicalein | NCOA1 |
| baicalein | CALM1 |
| Baicalin | F10 |
| Baicalin | CA2 |
| Baicalin | PTPN1 |
| Baicalin | GSK3B |
| Baicalin | PIM1 |
| Baicalin | CCNA2 |
| Spinasterol | ESR1 |
| Spinasterol | AR |
| Spinasterol | PGR |
| Spinasterol | NR3C2 |
| Spinasterol | NR3C1 |
| Spinasterol | NCOA2 |
| (2R,3R)-4-methoxyl-distylin | NOS2 |
| (2R,3R)-4-methoxyl-distylin | PTGS1 |
| (2R,3R)-4-methoxyl-distylin | ESR1 |
| (2R,3R)-4-methoxyl-distylin | AR |
| (2R,3R)-4-methoxyl-distylin | PPARG |
| (2R,3R)-4-methoxyl-distylin | PTGS2 |
| (2R,3R)-4-methoxyl-distylin | PTPN1 |
| (2R,3R)-4-methoxyl-distylin | ESR2 |
| (2R,3R)-4-methoxyl-distylin | DPP4 |
| (2R,3R)-4-methoxyl-distylin | MAPK14 |
| (2R,3R)-4-methoxyl-distylin | GSK3B |
| (2R,3R)-4-methoxyl-distylin | HSP90AB1 |
| (2R,3R)-4-methoxyl-distylin | PIM1 |
| (2R,3R)-4-methoxyl-distylin | CCNA2 |
| 1-o-beta-d-glucopyranosyl-8-o-benzoylpaeonisuffrone_qt | DPP4 |
| 1-o-beta-d-glucopyranosylpaeonisuffrone_qt | DPP4 |
| stigmast-7-en-3-ol | ESR1 |
| stigmast-7-en-3-ol | AR |
| stigmast-7-en-3-ol | PGR |
| Albiflorin_qt | DPP4 |
| Paeoniflorigenone | DPP4 |
| 9-ethyl-neo-paeoniaflorin A_qt | DPP4 |
| FA | F2 |
| FA | GSK3B |
| Mandenol | PTGS1 |
| Mandenol | F2 |
| Mandenol | PPARG |
| Mandenol | PTGS2 |
| Mandenol | NOS3 |
| Mandenol | ACHE |
| Mandenol | DPP4 |
| Mandenol | NCOA2 |
| Myricanone | NOS2 |
| Myricanone | PTGS1 |
| Myricanone | F2 |
| Myricanone | KCNH2 |
| Myricanone | ESR1 |
| Myricanone | AR |
| Myricanone | SCN5A |
| Myricanone | PPARG |
| Myricanone | PTGS2 |
| Myricanone | F7 |
| Myricanone | KDR |
| Myricanone | RXRA |
| Myricanone | PDE3A |
| Myricanone | ADRB2 |
| Myricanone | ESR2 |
| Myricanone | DPP4 |
| Myricanone | MAPK14 |
| Myricanone | GSK3B |
| Myricanone | HSP90AB1 |
| Myricanone | PIM1 |
| Myricanone | CCNA2 |
| Myricanone | NCOA1 |
| Perlolyrine | F2 |
| Perlolyrine | ESR1 |
| Perlolyrine | PTGS2 |
| Perlolyrine | RXRA |
| Perlolyrine | ESR2 |
| Perlolyrine | MAPK14 |
| Perlolyrine | GSK3B |
| Perlolyrine | PIM1 |
| Perlolyrine | CCNA2 |
| wallichilide | F2 |
| wallichilide | ESR1 |
| wallichilide | AR |
| wallichilide | PTGS2 |
| wallichilide | NR3C2 |
| wallichilide | NR3C1 |
| wallichilide | NCOA2 |
| quercetin | NOS2 |
| quercetin | PTGS1 |
| quercetin | INSR |
| quercetin | ESR1 |
| quercetin | AR |
| quercetin | BCL2 |
| quercetin | ALOX5 |
| quercetin | PTGS2 |
| quercetin | ODC1 |
| quercetin | ACACA |
| quercetin | MMP2 |
| quercetin | TNF |
| quercetin | ESR2 |
| quercetin | MGAM |
| quercetin | IL6 |
| quercetin | MMP1 |
| quercetin | MAPK1 |
| quercetin | PON1 |
| quercetin | CTSD |
| quercetin | IFNG |
| quercetin | JUN |
| quercetin | CCL2 |
| quercetin | IL1B |
| quercetin | GSK3B |
| quercetin | SELE |
| quercetin | MPO |
| quercetin | PLAT |
| quercetin | GJA1 |
| quercetin | VCAM1 |
| quercetin | THBD |
| quercetin | F3 |
| quercetin | NQO1 |
| quercetin | XDH |
| quercetin | SOD1 |
| quercetin | CYP3A4 |
| quercetin | TP53 |
| quercetin | CYP1A2 |
| quercetin | COL1A1 |
| quercetin | GSTP1 |
| quercetin | EGF |
| quercetin | VEGFA |
| quercetin | POR |
| quercetin | TOP1 |
| quercetin | HMOX1 |
| quercetin | COL8A1 |
| quercetin | RB1 |
| quercetin | GSTM1 |
| quercetin | AHR |
| quercetin | GSTM2 |
| quercetin | IL2 |
| quercetin | F2 |
| quercetin | KCNH2 |
| quercetin | SCN5A |
| quercetin | PPARG |
| quercetin | F10 |
| quercetin | NOS3 |
| quercetin | CA2 |
| quercetin | F7 |
| quercetin | RXRA |
| quercetin | ACHE |
| quercetin | PTPN1 |
| quercetin | ADRB2 |
| quercetin | DPP4 |
| quercetin | MAPK14 |
| quercetin | MMP3 |
| quercetin | HSP90AB1 |
| quercetin | PIK3CG |
| quercetin | MAOB |
| quercetin | PRSS1 |
| quercetin | PIM1 |
| quercetin | CCNA2 |
| quercetin | NCOA2 |
| isorhamnetin | NOS2 |
| isorhamnetin | XDH |
| isorhamnetin | PTGS1 |
| isorhamnetin | F2 |
| isorhamnetin | ESR1 |
| isorhamnetin | AR |
| isorhamnetin | PPARG |
| isorhamnetin | PTGS2 |
| isorhamnetin | NOS3 |
| isorhamnetin | CA2 |
| isorhamnetin | F7 |
| isorhamnetin | ACHE |
| isorhamnetin | PTPN1 |
| isorhamnetin | ESR2 |
| isorhamnetin | DPP4 |
| isorhamnetin | MAPK14 |
| isorhamnetin | GSK3B |
| isorhamnetin | HSP90AB1 |
| isorhamnetin | PIK3CG |
| isorhamnetin | MAOB |
| isorhamnetin | PRSS1 |
| isorhamnetin | PIM1 |
| isorhamnetin | CCNA2 |
| isorhamnetin | NCOA2 |
| isorhamnetin | NCOA1 |
| isorhamnetin | CALM1 |
| kaempferol | NOS2 |
| kaempferol | INSR |
| kaempferol | ESR1 |
| kaempferol | BCL2 |
| kaempferol | ALOX5 |
| kaempferol | PTGS2 |
| kaempferol | AKR1C3 |
| kaempferol | TNF |
| kaempferol | ESR2 |
| kaempferol | MMP1 |
| kaempferol | JUN |
| kaempferol | SELE |
| kaempferol | VCAM1 |
| kaempferol | XDH |
| kaempferol | CYP3A4 |
| kaempferol | MAPK8 |
| kaempferol | CYP1A2 |
| kaempferol | GSTP1 |
| kaempferol | HMOX1 |
| kaempferol | GSTM1 |
| kaempferol | AHR |
| kaempferol | GSTM2 |
| kaempferol | PPP3CA |
| kaempferol | PTGS1 |
| kaempferol | F2 |
| kaempferol | CHRM1 |
| kaempferol | AR |
| kaempferol | PPARG |
| kaempferol | NOS3 |
| kaempferol | CA2 |
| kaempferol | F7 |
| kaempferol | ACHE |
| kaempferol | SLC6A2 |
| kaempferol | PGR |
| kaempferol | ADRA1B |
| kaempferol | PTPN1 |
| kaempferol | DPP4 |
| kaempferol | MAPK14 |
| kaempferol | GSK3B |
| kaempferol | HSP90AB1 |
| kaempferol | PIK3CG |
| kaempferol | PRSS1 |
| kaempferol | PIM1 |
| kaempferol | CCNA2 |
| kaempferol | NCOA2 |
| kaempferol | CALM1 |
| petunidin | NOS2 |
| petunidin | PTGS1 |
| petunidin | ESR1 |
| petunidin | AR |
| petunidin | PPARG |
| petunidin | PTGS2 |
| petunidin | PTPN1 |
| petunidin | ESR2 |
| petunidin | DPP4 |
| petunidin | MAPK14 |
| petunidin | GSK3B |
| petunidin | HSP90AB1 |
| petunidin | PRSS1 |
| petunidin | PIM1 |
| petunidin | CCNA2 |
| petunidin | NCOA2 |
| Linoleyl acetate | PTGS1 |
| Linoleyl acetate | F2 |
| Linoleyl acetate | PPARG |
| Linoleyl acetate | PTGS2 |
| Linoleyl acetate | NOS3 |
| Linoleyl acetate | RXRA |
| Linoleyl acetate | ACHE |
| Linoleyl acetate | NCOA2 |
| 3,5,6,7-tetramethoxy-2-(3,4,5-trimethoxyphenyl)chromone | F2 |
| 3,5,6,7-tetramethoxy-2-(3,4,5-trimethoxyphenyl)chromone | ESR1 |
| 3,5,6,7-tetramethoxy-2-(3,4,5-trimethoxyphenyl)chromone | AR |
| 3,5,6,7-tetramethoxy-2-(3,4,5-trimethoxyphenyl)chromone | F10 |
| 3,5,6,7-tetramethoxy-2-(3,4,5-trimethoxyphenyl)chromone | PTGS2 |
| 3,5,6,7-tetramethoxy-2-(3,4,5-trimethoxyphenyl)chromone | CA2 |
| 3,5,6,7-tetramethoxy-2-(3,4,5-trimethoxyphenyl)chromone | F7 |
| 3,5,6,7-tetramethoxy-2-(3,4,5-trimethoxyphenyl)chromone | ACHE |
| 3,5,6,7-tetramethoxy-2-(3,4,5-trimethoxyphenyl)chromone | ESR2 |
| 3,5,6,7-tetramethoxy-2-(3,4,5-trimethoxyphenyl)chromone | PRSS1 |
| 3,5,6,7-tetramethoxy-2-(3,4,5-trimethoxyphenyl)chromone | PIM1 |
| 3,5,6,7-tetramethoxy-2-(3,4,5-trimethoxyphenyl)chromone | NCOA2 |
| 3,5,6,7-tetramethoxy-2-(3,4,5-trimethoxyphenyl)chromone | CALM1 |
| Areapillin | NOS2 |
| Areapillin | F2 |
| Areapillin | ESR1 |
| Areapillin | AR |
| Areapillin | SCN5A |
| Areapillin | PPARG |
| Areapillin | F10 |
| Areapillin | PTGS2 |
| Areapillin | CA2 |
| Areapillin | F7 |
| Areapillin | PTPN1 |
| Areapillin | ESR2 |
| Areapillin | DPP4 |
| Areapillin | MAPK14 |
| Areapillin | GSK3B |
| Areapillin | HSP90AB1 |
| Areapillin | PRSS1 |
| Areapillin | PIM1 |
| Areapillin | CCNA2 |
| Areapillin | NCOA2 |
| Areapillin | NCOA1 |
| Areapillin | CALM1 |
| Longikaurin A | CHRM1 |
| Longikaurin A | PRSS1 |
| (+)-Anomalin | NOS2 |
| (+)-Anomalin | F2 |
| (+)-Anomalin | KCNH2 |
| (+)-Anomalin | ESR1 |
| (+)-Anomalin | AR |
| (+)-Anomalin | F10 |
| (+)-Anomalin | PTGS2 |
| (+)-Anomalin | CA2 |
| (+)-Anomalin | DPP4 |
| saikosaponin c_qt | AR |
| saikosaponin c_qt | NR3C1 |
| α-spinasterol | ESR1 |
| α-spinasterol | AR |
| α-spinasterol | PGR |
| α-spinasterol | NR3C2 |
| α-spinasterol | NR3C1 |
| α-spinasterol | NCOA2 |
| Cubebin | NOS2 |
| Cubebin | PTGS1 |
| Cubebin | F2 |
| Cubebin | ESR1 |
| Cubebin | AR |
| Cubebin | PPARG |
| Cubebin | F10 |
| Cubebin | PTGS2 |
| Cubebin | ADRB2 |
| Cubebin | ESR2 |
| Cubebin | DPP4 |
| Cubebin | MAPK14 |
| Cubebin | GSK3B |
| Cubebin | HSP90AB1 |
| Cubebin | PRSS1 |
| Cubebin | PIM1 |
| Cubebin | CCNA2 |
| luteolin | NOS2 |
| luteolin | INSR |
| luteolin | AR |
| luteolin | PTGS2 |
| luteolin | MMP2 |
| luteolin | TNF |
| luteolin | MET |
| luteolin | IL6 |
| luteolin | MMP1 |
| luteolin | MAPK1 |
| luteolin | IFNG |
| luteolin | JUN |
| luteolin | XDH |
| luteolin | TP53 |
| luteolin | GSTP1 |
| luteolin | VEGFA |
| luteolin | TOP1 |
| luteolin | HMOX1 |
| luteolin | RB1 |
| luteolin | CASP7 |
| luteolin | IL2 |
| luteolin | PTGS1 |
| luteolin | ESR1 |
| luteolin | PPARG |
| luteolin | CA2 |
| luteolin | PTPN1 |
| luteolin | ESR2 |
| luteolin | DPP4 |
| luteolin | MAPK14 |
| luteolin | GSK3B |
| luteolin | HSP90AB1 |
| luteolin | PIK3CG |
| luteolin | PRSS1 |
| luteolin | PIM1 |
| luteolin | CCNA2 |
| luteolin | NCOA2 |
| CLR | ESR1 |
| CLR | AR |
| CLR | PGR |
| CLR | NR3C2 |
| CLR | NR3C1 |
| CLR | DPP4 |
| CLR | PRSS1 |
| CLR | NCOA2 |
| poriferast-5-en-3beta-ol | ESR1 |
| poriferast-5-en-4beta-ol | AR |
| poriferast-5-en-5beta-ol | PGR |
| poriferast-5-en-6beta-ol | NR3C1 |
| poriferast-5-en-7beta-ol | NCOA2 |
| 4-[(E)-4-(3,5-dimethoxy-4-oxo-1-cyclohexa-2,5-dienylidene)but-2-enylidene]-2,6-dimethoxycyclohexa-2,5-dien-1-one | NOS2 |
| 4-[(E)-4-(3,5-dimethoxy-4-oxo-1-cyclohexa-2,5-dienylidene)but-2-enylidene]-2,6-dimethoxycyclohexa-2,5-dien-1-one | F2 |
| 4-[(E)-4-(3,5-dimethoxy-4-oxo-1-cyclohexa-2,5-dienylidene)but-2-enylidene]-2,6-dimethoxycyclohexa-2,5-dien-1-one | ESR1 |
| 4-[(E)-4-(3,5-dimethoxy-4-oxo-1-cyclohexa-2,5-dienylidene)but-2-enylidene]-2,6-dimethoxycyclohexa-2,5-dien-1-one | AR |
| 4-[(E)-4-(3,5-dimethoxy-4-oxo-1-cyclohexa-2,5-dienylidene)but-2-enylidene]-2,6-dimethoxycyclohexa-2,5-dien-1-one | PTGS2 |
| 4-[(E)-4-(3,5-dimethoxy-4-oxo-1-cyclohexa-2,5-dienylidene)but-2-enylidene]-2,6-dimethoxycyclohexa-2,5-dien-1-one | NOS3 |
| 4-[(E)-4-(3,5-dimethoxy-4-oxo-1-cyclohexa-2,5-dienylidene)but-2-enylidene]-2,6-dimethoxycyclohexa-2,5-dien-1-one | CA2 |
| 4-[(E)-4-(3,5-dimethoxy-4-oxo-1-cyclohexa-2,5-dienylidene)but-2-enylidene]-2,6-dimethoxycyclohexa-2,5-dien-1-one | NCOA2 |
| lignan | F2 |
| lignan | ESR1 |
| lignan | AR |
| lignan | F10 |
| lignan | PTGS2 |
| lignan | CA2 |
| lignan | PRSS1 |
| lignan | PIM1 |
| lignan | NCOA2 |
| lignan | KCNMA1 |
| lignan | CALM1 |
| Pyrethrin II | F2 |
| Pyrethrin II | ESR1 |
| Pyrethrin II | AR |
| Pyrethrin II | PTGS2 |
| Pyrethrin II | NCOA2 |
| 6-Hydroxykaempferol | NOS2 |
| 6-Hydroxykaempferol | PTGS1 |
| 6-Hydroxykaempferol | ESR1 |
| 6-Hydroxykaempferol | AR |
| 6-Hydroxykaempferol | PPARG |
| 6-Hydroxykaempferol | PTGS2 |
| 6-Hydroxykaempferol | CA2 |
| 6-Hydroxykaempferol | PTPN1 |
| 6-Hydroxykaempferol | ESR2 |
| 6-Hydroxykaempferol | DPP4 |
| 6-Hydroxykaempferol | MAPK14 |
| 6-Hydroxykaempferol | HSP90AB1 |
| 6-Hydroxykaempferol | PIK3CG |
| 6-Hydroxykaempferol | PRSS1 |
| 6-Hydroxykaempferol | PIM1 |
| 6-Hydroxykaempferol | NCOA2 |
| qt_carthamone | NOS2 |
| qt_carthamone | PTGS1 |
| qt_carthamone | F2 |
| qt_carthamone | ESR1 |
| qt_carthamone | AR |
| qt_carthamone | PPARG |
| qt_carthamone | PTGS2 |
| qt_carthamone | NOS3 |
| qt_carthamone | CA2 |
| qt_carthamone | PTPN1 |
| qt_carthamone | DPP4 |
| qt_carthamone | MAPK14 |
| qt_carthamone | HSP90AB1 |
| qt_carthamone | CCNA2 |
| quercetagetin | NOS2 |
| quercetagetin | ESR1 |
| quercetagetin | AR |
| quercetagetin | PPARG |
| quercetagetin | PTGS2 |
| quercetagetin | CA2 |
| quercetagetin | PTPN1 |
| quercetagetin | ESR2 |
| quercetagetin | DPP4 |
| quercetagetin | MAPK14 |
| quercetagetin | HSP90AB1 |
| quercetagetin | PIK3CG |
| quercetagetin | PRSS1 |
| quercetagetin | PIM1 |
| 7,8-dimethyl-1H-pyrimido[5,6-g]quinoxaline-2,4-dione | NOS2 |
| 7,8-dimethyl-1H-pyrimido[5,6-g]quinoxaline-2,4-dione | PTGS1 |
| 7,8-dimethyl-1H-pyrimido[5,6-g]quinoxaline-2,4-dione | F2 |
| 7,8-dimethyl-1H-pyrimido[5,6-g]quinoxaline-2,4-dione | ESR1 |
| 7,8-dimethyl-1H-pyrimido[5,6-g]quinoxaline-2,4-dione | AR |
| 7,8-dimethyl-1H-pyrimido[5,6-g]quinoxaline-2,4-dione | PTGS2 |
| 7,8-dimethyl-1H-pyrimido[5,6-g]quinoxaline-2,4-dione | CA2 |
| 7,8-dimethyl-1H-pyrimido[5,6-g]quinoxaline-2,4-dione | PTPN1 |
| 7,8-dimethyl-1H-pyrimido[5,6-g]quinoxaline-2,4-dione | DPP4 |
| 7,8-dimethyl-1H-pyrimido[5,6-g]quinoxaline-2,4-dione | MAPK14 |
| 7,8-dimethyl-1H-pyrimido[5,6-g]quinoxaline-2,4-dione | HSP90AB1 |
| 7,8-dimethyl-1H-pyrimido[5,6-g]quinoxaline-2,4-dione | PIM1 |
| 7,8-dimethyl-1H-pyrimido[5,6-g]quinoxaline-2,4-dione | CCNA2 |
| beta-carotene | BCL2 |
| beta-carotene | PTGS2 |
| beta-carotene | MMP2 |
| beta-carotene | MMP1 |
| beta-carotene | JUN |
| beta-carotene | GJA1 |
| beta-carotene | F3 |
| beta-carotene | CYP3A4 |
| beta-carotene | CYP1A2 |
| beta-carotene | MMP10 |
| beta-carotene | VEGFA |
| beta-carotene | HMOX1 |
| beta-carotene | CASP7 |
| hederagenin | PTGS1 |
| hederagenin | CHRM3 |
| hederagenin | F2 |
| hederagenin | CHRM1 |
| hederagenin | ESR1 |
| hederagenin | AR |
| hederagenin | SCN5A |
| hederagenin | PPARG |
| hederagenin | PTGS2 |
| hederagenin | NOS3 |
| hederagenin | CA2 |
| hederagenin | RXRA |
| hederagenin | ACHE |
| hederagenin | PDE3A |
| hederagenin | SLC6A2 |
| hederagenin | PGR |
| hederagenin | ADRA1B |
| hederagenin | NR3C1 |
| hederagenin | DPP4 |
| hederagenin | ADH1C |
| hederagenin | LYZ |
| hederagenin | PRSS1 |
| hederagenin | NCOA2 |
| campesterol | NOS2 |
| campesterol | PTGS1 |
| campesterol | ESR1 |
| campesterol | AR |
| campesterol | PPARG |
| campesterol | PTGS2 |
| campesterol | CA2 |
| campesterol | PGR |
| campesterol | PTPN1 |
| campesterol | ESR2 |
| campesterol | MAPK14 |
| campesterol | GSK3B |
| campesterol | HSP90AB1 |
| campesterol | PIK3CG |
| campesterol | PIM1 |
| campesterol | CCNA2 |
| campesterol | NCOA2 |
| campesterol | ESR1 |
| campesterol | AR |
| campesterol | PTGS2 |
| campesterol | ACHE |
| campesterol | PGR |
| campesterol | NR3C2 |
| campesterol | NR3C1 |
| campesterol | DPP4 |
| campesterol | ADH1C |
| campesterol | PRSS1 |
| 2,3-didehydro GA70 | NOS2 |
| 2,3-didehydro GA70 | PTGS1 |
| 2,3-didehydro GA70 | F2 |
| 2,3-didehydro GA70 | CHRM1 |
| 2,3-didehydro GA70 | PTGS2 |
| 2,3-didehydro GA70 | NOS3 |
| 2,3-didehydro GA70 | ACHE |
| 2,3-didehydro GA70 | SLC6A2 |
| 2,3-didehydro GA70 | DPP4 |
| 2,3-didehydro GA70 | PRSS1 |
| 2,3-didehydro GA77 | F2 |
| 2,3-didehydro GA77 | ESR1 |
| 2,3-didehydro GA77 | AR |
| 2,3-didehydro GA77 | PTGS2 |
| 2,3-didehydro GA77 | NOS3 |
| 2,3-didehydro GA77 | CA2 |
| 2,3-didehydro GA77 | ACHE |
| 2,3-didehydro GA77 | DPP4 |
| 2,3-didehydro GA77 | NCOA2 |
| GA120 | NOS2 |
| GA120 | CHRM3 |
| GA120 | F2 |
| GA120 | CHRM1 |
| GA120 | PTGS2 |
| GA120 | ACHE |
| GA120 | DPP4 |
| GA121-isolactone | ESR1 |
| GA121-isolactone | AR |
| GA121-isolactone | PGR |
| GA122-isolactone | ESR1 |
| GA122-isolactone | AR |
| GA122-isolactone | PGR |
| 4a-formyl-7alpha-hydroxy-1-methyl-8-methylidene-4aalpha,4bbeta-gibbane-1alpha,10beta-dicarboxylic acid | NOS2 |
| 4a-formyl-7alpha-hydroxy-1-methyl-8-methylidene-4aalpha,4bbeta-gibbane-1alpha,10beta-dicarboxylic acid | ESR1 |
| 4a-formyl-7alpha-hydroxy-1-methyl-8-methylidene-4aalpha,4bbeta-gibbane-1alpha,10beta-dicarboxylic acid | AR |
| 4a-formyl-7alpha-hydroxy-1-methyl-8-methylidene-4aalpha,4bbeta-gibbane-1alpha,10beta-dicarboxylic acid | PGR |
| 4a-formyl-7alpha-hydroxy-1-methyl-8-methylidene-4aalpha,4bbeta-gibbane-1alpha,10beta-dicarboxylic acid | NR3C2 |
| 4a-formyl-7alpha-hydroxy-1-methyl-8-methylidene-4aalpha,4bbeta-gibbane-1alpha,10beta-dicarboxylic acid | NR3C1 |
| Gibberellin A44 | F2 |
| Gibberellin A44 | ESR1 |
| Gibberellin A44 | AR |
| Gibberellin A44 | ACHE |
| Gibberellin A44 | NR3C2 |
| Gibberellin A44 | DPP4 |
| GA54 | NOS2 |
| GA54 | F2 |
| GA54 | ESR1 |
| GA54 | AR |
| GA54 | PPARG |
| GA54 | F10 |
| GA54 | PTGS2 |
| GA54 | NOS3 |
| GA54 | CA2 |
| GA54 | PTPN1 |
| GA54 | DPP4 |
| GA54 | GSK3B |
| GA54 | HSP90AB1 |
| GA54 | PRSS1 |
| GA54 | NCOA2 |
| GA54 | CALM1 |
| GA60 | NOS2 |
| GA60 | F2 |
| GA60 | NOS3 |
| GA60 | ACHE |
| GA63 | F2 |
| GA63 | PTGS2 |
| GA63 | ACHE |
| gibberellin 7 | NOS2 |
| gibberellin 7 | CHRM3 |
| gibberellin 7 | F2 |
| gibberellin 7 | CHRM1 |
| gibberellin 7 | ESR1 |
| gibberellin 7 | PTGS2 |
| gibberellin 7 | NOS3 |
| gibberellin 7 | PDE3A |
| gibberellin 7 | SLC6A3 |
| gibberellin 7 | ADRB2 |
| gibberellin 7 | SLC6A4 |
| GA77 | F2 |
| GA77 | GABRA2 |
| GA77 | GABRA1 |
| GA77 | DPP4 |
| GA77 | PRSS1 |
| GA87 | PTGS2 |
| GA87 | CA2 |
| GA87 | DPP4 |
| 3-O-p-coumaroylquinic acid | NOS2 |
| 3-O-p-coumaroylquinic acid | PTGS1 |
| 3-O-p-coumaroylquinic acid | ESR1 |
| 3-O-p-coumaroylquinic acid | AR |
| 3-O-p-coumaroylquinic acid | PPARG |
| 3-O-p-coumaroylquinic acid | PTGS2 |
| 3-O-p-coumaroylquinic acid | CA2 |
| 3-O-p-coumaroylquinic acid | PTPN1 |
| 3-O-p-coumaroylquinic acid | ESR2 |
| 3-O-p-coumaroylquinic acid | DPP4 |
| 3-O-p-coumaroylquinic acid | MAPK14 |
| 3-O-p-coumaroylquinic acid | GSK3B |
| 3-O-p-coumaroylquinic acid | HSP90AB1 |
| 3-O-p-coumaroylquinic acid | PIK3CG |
| 3-O-p-coumaroylquinic acid | PRSS1 |
| 3-O-p-coumaroylquinic acid | PIM1 |
| 3-O-p-coumaroylquinic acid | CCNA2 |
| 3-O-p-coumaroylquinic acid | NCOA2 |
| 3-O-p-coumaroylquinic acid | CALM1 |
| beta-daucosterol_qt | ESR1 |
| beta-daucosterol_qt | AR |
| beta-daucosterol_qt | PGR |
| wogonin | NOS2 |
| wogonin | PTGS1 |
| wogonin | BCL2 |
| wogonin | PTGS2 |
| wogonin | KDR |
| wogonin | TNF |
| wogonin | IL6 |
| wogonin | MMP1 |
| wogonin | JUN |
| wogonin | CCL2 |
| wogonin | GSK3B |
| wogonin | TP53 |
| wogonin | ESR1 |
| wogonin | AR |
| wogonin | SCN5A |
| wogonin | PPARG |
| wogonin | CA2 |
| wogonin | RXRA |
| wogonin | PDE3A |
| wogonin | PTPN1 |
| wogonin | ADRB2 |
| wogonin | ESR2 |
| wogonin | DPP4 |
| wogonin | MAPK14 |
| wogonin | HSP90AB1 |
| wogonin | PIK3CG |
| wogonin | PRSS1 |
| wogonin | PIM1 |
| wogonin | CCNA2 |
| wogonin | CALM1 |
| palmatine | NOS2 |
| palmatine | PTGS1 |
| palmatine | F2 |
| palmatine | KCNH2 |
| palmatine | ESR1 |
| palmatine | AR |
| palmatine | SCN5A |
| palmatine | PPARG |
| palmatine | PTGS2 |
| palmatine | NOS3 |
| palmatine | CA2 |
| palmatine | F7 |
| palmatine | RXRA |
| palmatine | ACHE |
| palmatine | PTPN1 |
| palmatine | ADRB2 |
| palmatine | ESR2 |
| palmatine | DPP4 |
| palmatine | MAPK14 |
| palmatine | GSK3B |
| palmatine | HSP90AB1 |
| palmatine | PRSS1 |
| palmatine | PIM1 |
| palmatine | CCNA2 |
| palmatine | NCOA2 |
| palmatine | CALM1 |
| poriferasta-7,22E-dien-3beta-ol | ESR1 |
| poriferasta-7,22E-dien-3beta-ol | AR |
| poriferasta-7,22E-dien-3beta-ol | PGR |
| poriferasta-7,22E-dien-3beta-ol | NR3C2 |
| poriferasta-7,22E-dien-3beta-ol | NR3C1 |
| poriferasta-7,22E-dien-3beta-ol | NCOA2 |
| berberine | NOS2 |
| berberine | PTGS1 |
| berberine | F2 |
| berberine | KCNH2 |
| berberine | ESR1 |
| berberine | AR |
| berberine | SCN5A |
| berberine | PPARG |
| berberine | F10 |
| berberine | PTGS2 |
| berberine | NOS3 |
| berberine | CA2 |
| berberine | RXRA |
| berberine | ACHE |
| berberine | ADRB2 |
| berberine | ESR2 |
| berberine | DPP4 |
| berberine | MAPK14 |
| berberine | GSK3B |
| berberine | HSP90AB1 |
| berberine | PRSS1 |
| berberine | PIM1 |
| berberine | CCNA2 |
| berberine | NCOA2 |
| berberine | CALM1 |
| coptisine | NOS2 |
| coptisine | PTGS1 |
| coptisine | F2 |
| coptisine | KCNH2 |
| coptisine | ESR1 |
| coptisine | AR |
| coptisine | SCN5A |
| coptisine | PPARG |
| coptisine | PTGS2 |
| coptisine | NOS3 |
| coptisine | ACHE |
| coptisine | ESR2 |
| coptisine | DPP4 |
| coptisine | MAPK14 |
| coptisine | GSK3B |
| coptisine | PRSS1 |
| coptisine | PIM1 |
| coptisine | CCNA2 |
| delta 7-stigmastenol | ESR1 |
| delta 7-stigmastenol | AR |
| delta 7-stigmastenol | PGR |
| epiberberine | NOS2 |
| epiberberine | F2 |
| epiberberine | KCNH2 |
| epiberberine | ESR1 |
| epiberberine | AR |
| epiberberine | PPARG |
| epiberberine | PTGS2 |
| epiberberine | NOS3 |
| epiberberine | RXRA |
| epiberberine | ACHE |
| epiberberine | ESR2 |
| epiberberine | DPP4 |
| epiberberine | MAPK14 |
| epiberberine | GSK3B |
| epiberberine | PRSS1 |
| epiberberine | PIM1 |
| epiberberine | CCNA2 |
| epiberberine | NCOA2 |
| Inophyllum E | PTGS1 |
| Inophyllum E | F2 |
| Inophyllum E | ESR1 |
| Inophyllum E | AR |
| Inophyllum E | F10 |
| Inophyllum E | PTGS2 |
| Inophyllum E | CA2 |
| Inophyllum E | ESR2 |
| Inophyllum E | GSK3B |
| Inophyllum E | PIM1 |
| 28-norolean-17-en-3-ol | ESR1 |
| 28-norolean-17-en-3-ol | AR |
| 28-norolean-17-en-3-ol | PGR |
| Mairin | ESR1 |
| Mairin | AR |
| Mairin | PGR |
| Mairin | NR3C1 |
| Jaranol | NOS2 |
| Jaranol | PTGS1 |
| Jaranol | ESR1 |
| Jaranol | AR |
| Jaranol | SCN5A |
| Jaranol | PPARG |
| Jaranol | PTGS2 |
| Jaranol | CA2 |
| Jaranol | PTPN1 |
| Jaranol | ESR2 |
| Jaranol | DPP4 |
| Jaranol | MAPK14 |
| Jaranol | GSK3B |
| Jaranol | HSP90AB1 |
| Jaranol | PRSS1 |
| Jaranol | PIM1 |
| Jaranol | CCNA2 |
| Jaranol | NCOA2 |
| Jaranol | CALM1 |
| formononetin | ESR1 |
| formononetin | ESR2 |
| formononetin | JUN |
| formononetin | MT-ND6 |
| formononetin | ATP5F1B |
| formononetin | NOS2 |
| formononetin | PTGS1 |
| formononetin | F2 |
| formononetin | CHRM1 |
| formononetin | AR |
| formononetin | PPARG |
| formononetin | PTGS2 |
| formononetin | NOS3 |
| formononetin | CA2 |
| formononetin | RXRA |
| formononetin | ACHE |
| formononetin | PDE3A |
| formononetin | ADRA1A |
| formononetin | PTPN1 |
| formononetin | SLC6A3 |
| formononetin | ADRB2 |
| formononetin | SLC6A4 |
| formononetin | DPP4 |
| formononetin | MAPK14 |
| formononetin | GSK3B |
| formononetin | HSP90AB1 |
| formononetin | MAOB |
| formononetin | PRSS1 |
| formononetin | PIM1 |
| formononetin | CCNA2 |
| formononetin | CALM1 |
| Calycosin | NOS2 |
| Calycosin | PTGS1 |
| Calycosin | ESR1 |
| Calycosin | AR |
| Calycosin | PPARG |
| Calycosin | PTGS2 |
| Calycosin | CA2 |
| Calycosin | RXRA |
| Calycosin | PDE3A |
| Calycosin | PTPN1 |
| Calycosin | ADRB2 |
| Calycosin | ESR2 |
| Calycosin | DPP4 |
| Calycosin | MAPK14 |
| Calycosin | GSK3B |
| Calycosin | HSP90AB1 |
| Calycosin | PRSS1 |
| Calycosin | PIM1 |
| Calycosin | CCNA2 |
| Calycosin | NCOA2 |
| Calycosin | CALM1 |
| licochalcone a | BCL2 |
| licochalcone a | MAPK1 |
| licochalcone a | RB1 |
| licochalcone a | CCNA2 |
| licochalcone a | NOS2 |
| licochalcone a | PTGS1 |
| licochalcone a | F2 |
| licochalcone a | CHRM1 |
| licochalcone a | ESR1 |
| licochalcone a | AR |
| licochalcone a | SCN5A |
| licochalcone a | PPARG |
| licochalcone a | F10 |
| licochalcone a | PTGS2 |
| licochalcone a | CA2 |
| licochalcone a | ADRA1B |
| licochalcone a | PTPN1 |
| licochalcone a | ADRB2 |
| licochalcone a | ESR2 |
| licochalcone a | DPP4 |
| licochalcone a | MAPK14 |
| licochalcone a | GSK3B |
| licochalcone a | HSP90AB1 |
| licochalcone a | PRSS1 |
| licochalcone a | PIM1 |
| licochalcone a | NCOA2 |
| licochalcone a | CALM1 |
| Vestitol | NOS2 |
| Vestitol | PTGS1 |
| Vestitol | CHRM1 |
| Vestitol | ESR1 |
| Vestitol | AR |
| Vestitol | SCN5A |
| Vestitol | PPARG |
| Vestitol | PTGS2 |
| Vestitol | CA2 |
| Vestitol | RXRA |
| Vestitol | PDE3A |
| Vestitol | HTR2A |
| Vestitol | ADRA1A |
| Vestitol | ADRA1B |
| Vestitol | PTPN1 |
| Vestitol | ADRB2 |
| Vestitol | SLC6A4 |
| Vestitol | ESR2 |
| Vestitol | DPP4 |
| Vestitol | MAPK14 |
| Vestitol | GSK3B |
| Vestitol | HSP90AB1 |
| Vestitol | PRSS1 |
| Vestitol | PIM1 |
| Vestitol | CCNA2 |
| Vestitol | CALM1 |
| Inermine | NOS2 |
| Inermine | PTGS1 |
| Inermine | CHRM3 |
| Inermine | CHRM1 |
| Inermine | ESR1 |
| Inermine | AR |
| Inermine | SCN5A |
| Inermine | PPARG |
| Inermine | PTGS2 |
| Inermine | HTR3A |
| Inermine | CA2 |
| Inermine | RXRA |
| Inermine | ADRA1B |
| Inermine | PTPN1 |
| Inermine | ADRB2 |
| Inermine | ADRA1D |
| Inermine | OPRM1 |
| Inermine | ESR2 |
| Inermine | DPP4 |
| Inermine | MAPK14 |
| Inermine | GSK3B |
| Inermine | HSP90AB1 |
| Inermine | PIK3CG |
| Inermine | PRSS1 |
| Inermine | PIM1 |
| Inermine | CCNA2 |
| Inermine | CALM1 |
| DFV | NOS2 |
| DFV | PTGS1 |
| DFV | ESR1 |
| DFV | AR |
| DFV | PPARG |
| DFV | PTGS2 |
| DFV | CA2 |
| DFV | RXRA |
| DFV | PTPN1 |
| DFV | ADRB2 |
| DFV | SLC6A4 |
| DFV | ESR2 |
| DFV | DPP4 |
| DFV | MAPK14 |
| DFV | GSK3B |
| DFV | HSP90AB1 |
| DFV | PIK3CG |
| DFV | MAOB |
| DFV | PIM1 |
| DFV | CCNA2 |
| Glycyrol | NOS2 |
| Glycyrol | F2 |
| Glycyrol | ESR1 |
| Glycyrol | PPARG |
| Glycyrol | PTGS2 |
| Glycyrol | KDR |
| Glycyrol | PTPN1 |
| Glycyrol | MAPK14 |
| Glycyrol | GSK3B |
| Glycyrol | PIM1 |
| Glycyrol | CCNA2 |
| Medicarpin | NOS2 |
| Medicarpin | PTGS1 |
| Medicarpin | DRD1 |
| Medicarpin | CHRM3 |
| Medicarpin | CHRM1 |
| Medicarpin | ESR1 |
| Medicarpin | AR |
| Medicarpin | SCN5A |
| Medicarpin | PPARG |
| Medicarpin | PTGS2 |
| Medicarpin | CA2 |
| Medicarpin | RXRA |
| Medicarpin | OPRD1 |
| Medicarpin | PDE3A |
| Medicarpin | HTR2A |
| Medicarpin | ADRA1A |
| Medicarpin | ADRA1B |
| Medicarpin | PTPN1 |
| Medicarpin | ADRB2 |
| Medicarpin | ADRA1D |
| Medicarpin | SLC6A4 |
| Medicarpin | OPRM1 |
| Medicarpin | ESR2 |
| Medicarpin | DPP4 |
| Medicarpin | MAPK14 |
| Medicarpin | GSK3B |
| Medicarpin | MAPK10 |
| Medicarpin | HSP90AB1 |
| Medicarpin | PIK3CG |
| Medicarpin | PRSS1 |
| Medicarpin | PIM1 |
| Medicarpin | CCNA2 |
| Medicarpin | CALM1 |
| Lupiwighteone | NOS2 |
| Lupiwighteone | F2 |
| Lupiwighteone | ESR1 |
| Lupiwighteone | AR |
| Lupiwighteone | SCN5A |
| Lupiwighteone | PPARG |
| Lupiwighteone | F10 |
| Lupiwighteone | PTGS2 |
| Lupiwighteone | CA2 |
| Lupiwighteone | PTPN1 |
| Lupiwighteone | ESR2 |
| Lupiwighteone | DPP4 |
| Lupiwighteone | MAPK14 |
| Lupiwighteone | GSK3B |
| Lupiwighteone | HSP90AB1 |
| Lupiwighteone | PRSS1 |
| Lupiwighteone | PIM1 |
| Lupiwighteone | CCNA2 |
| Lupiwighteone | NCOA2 |
| Lupiwighteone | CALM1 |
| 7-Methoxy-2-methyl isoflavone | NOS2 |
| 7-Methoxy-2-methyl isoflavone | PTGS1 |
| 7-Methoxy-2-methyl isoflavone | DRD1 |
| 7-Methoxy-2-methyl isoflavone | CHRM3 |
| 7-Methoxy-2-methyl isoflavone | F2 |
| 7-Methoxy-2-methyl isoflavone | CHRM1 |
| 7-Methoxy-2-methyl isoflavone | ESR1 |
| 7-Methoxy-2-methyl isoflavone | AR |
| 7-Methoxy-2-methyl isoflavone | ADRB1 |
| 7-Methoxy-2-methyl isoflavone | SCN5A |
| 7-Methoxy-2-methyl isoflavone | PPARG |
| 7-Methoxy-2-methyl isoflavone | PTGS2 |
| 7-Methoxy-2-methyl isoflavone | NOS3 |
| 7-Methoxy-2-methyl isoflavone | CA2 |
| 7-Methoxy-2-methyl isoflavone | RXRA |
| 7-Methoxy-2-methyl isoflavone | ACHE |
| 7-Methoxy-2-methyl isoflavone | PDE3A |
| 7-Methoxy-2-methyl isoflavone | ADRA1B |
| 7-Methoxy-2-methyl isoflavone | PTPN1 |
| 7-Methoxy-2-methyl isoflavone | ADRB2 |
| 7-Methoxy-2-methyl isoflavone | ADRA1D |
| 7-Methoxy-2-methyl isoflavone | SLC6A4 |
| 7-Methoxy-2-methyl isoflavone | OPRM1 |
| 7-Methoxy-2-methyl isoflavone | ESR2 |
| 7-Methoxy-2-methyl isoflavone | DPP4 |
| 7-Methoxy-2-methyl isoflavone | MAPK14 |
| 7-Methoxy-2-methyl isoflavone | GSK3B |
| 7-Methoxy-2-methyl isoflavone | HSP90AB1 |
| 7-Methoxy-2-methyl isoflavone | LTA4H |
| 7-Methoxy-2-methyl isoflavone | MAOB |
| 7-Methoxy-2-methyl isoflavone | PRSS1 |
| 7-Methoxy-2-methyl isoflavone | PIM1 |
| 7-Methoxy-2-methyl isoflavone | CCNA2 |
| 7-Methoxy-2-methyl isoflavone | NCOA2 |
| 7-Methoxy-2-methyl isoflavone | NCOA1 |
| 7-Methoxy-2-methyl isoflavone | CALM1 |
| naringenin | NOS2 |
| naringenin | ESR1 |
| naringenin | BCL2 |
| naringenin | ESR2 |
| naringenin | MAPK1 |
| naringenin | SOD1 |
| naringenin | GSTP1 |
| naringenin | CAT |
| naringenin | PTGS1 |
| naringenin | AR |
| naringenin | PPARG |
| naringenin | PTGS2 |
| naringenin | CA2 |
| naringenin | PTPN1 |
| naringenin | DPP4 |
| naringenin | MAPK14 |
| naringenin | GSK3B |
| naringenin | HSP90AB1 |
| naringenin | PIK3CG |
| naringenin | CCNA2 |
| (2S)-2-[4-hydroxy-3-(3-methylbut-2-enyl)phenyl]-8,8-dimethyl-2,3-dihydropyrano[2,3-f]chromen-4-one | NOS2 |
| (2S)-2-[4-hydroxy-3-(3-methylbut-2-enyl)phenyl]-8,8-dimethyl-2,3-dihydropyrano[2,3-f]chromen-5-one | F2 |
| (2S)-2-[4-hydroxy-3-(3-methylbut-2-enyl)phenyl]-8,8-dimethyl-2,3-dihydropyrano[2,3-f]chromen-6-one | KCNH2 |
| (2S)-2-[4-hydroxy-3-(3-methylbut-2-enyl)phenyl]-8,8-dimethyl-2,3-dihydropyrano[2,3-f]chromen-7-one | ESR1 |
| (2S)-2-[4-hydroxy-3-(3-methylbut-2-enyl)phenyl]-8,8-dimethyl-2,3-dihydropyrano[2,3-f]chromen-8-one | AR |
| (2S)-2-[4-hydroxy-3-(3-methylbut-2-enyl)phenyl]-8,8-dimethyl-2,3-dihydropyrano[2,3-f]chromen-9-one | PPARG |
| (2S)-2-[4-hydroxy-3-(3-methylbut-2-enyl)phenyl]-8,8-dimethyl-2,3-dihydropyrano[2,3-f]chromen-10-one | F10 |
| (2S)-2-[4-hydroxy-3-(3-methylbut-2-enyl)phenyl]-8,8-dimethyl-2,3-dihydropyrano[2,3-f]chromen-11-one | PTGS2 |
| (2S)-2-[4-hydroxy-3-(3-methylbut-2-enyl)phenyl]-8,8-dimethyl-2,3-dihydropyrano[2,3-f]chromen-12-one | CA2 |
| (2S)-2-[4-hydroxy-3-(3-methylbut-2-enyl)phenyl]-8,8-dimethyl-2,3-dihydropyrano[2,3-f]chromen-13-one | PTPN1 |
| (2S)-2-[4-hydroxy-3-(3-methylbut-2-enyl)phenyl]-8,8-dimethyl-2,3-dihydropyrano[2,3-f]chromen-14-one | ESR2 |
| (2S)-2-[4-hydroxy-3-(3-methylbut-2-enyl)phenyl]-8,8-dimethyl-2,3-dihydropyrano[2,3-f]chromen-15-one | DPP4 |
| (2S)-2-[4-hydroxy-3-(3-methylbut-2-enyl)phenyl]-8,8-dimethyl-2,3-dihydropyrano[2,3-f]chromen-16-one | MAPK14 |
| (2S)-2-[4-hydroxy-3-(3-methylbut-2-enyl)phenyl]-8,8-dimethyl-2,3-dihydropyrano[2,3-f]chromen-17-one | GSK3B |
| (2S)-2-[4-hydroxy-3-(3-methylbut-2-enyl)phenyl]-8,8-dimethyl-2,3-dihydropyrano[2,3-f]chromen-18-one | PRSS1 |
| (2S)-2-[4-hydroxy-3-(3-methylbut-2-enyl)phenyl]-8,8-dimethyl-2,3-dihydropyrano[2,3-f]chromen-19-one | PIM1 |
| (2S)-2-[4-hydroxy-3-(3-methylbut-2-enyl)phenyl]-8,8-dimethyl-2,3-dihydropyrano[2,3-f]chromen-20-one | CALM1 |
| euchrenone | NOS2 |
| euchrenone | F2 |
| euchrenone | KCNH2 |
| euchrenone | ESR1 |
| euchrenone | AR |
| euchrenone | SCN5A |
| euchrenone | PPARG |
| euchrenone | F10 |
| euchrenone | PTGS2 |
| euchrenone | PTPN1 |
| euchrenone | ESR2 |
| euchrenone | DPP4 |
| euchrenone | MAPK14 |
| euchrenone | PRSS1 |
| euchrenone | PIM1 |
| euchrenone | CALM1 |
| glyasperin B | NOS2 |
| glyasperin B | F2 |
| glyasperin B | ESR1 |
| glyasperin B | AR |
| glyasperin B | PPARG |
| glyasperin B | F10 |
| glyasperin B | PTGS2 |
| glyasperin B | CA2 |
| glyasperin B | F7 |
| glyasperin B | KDR |
| glyasperin B | ACHE |
| glyasperin B | PTPN1 |
| glyasperin B | ESR2 |
| glyasperin B | DPP4 |
| glyasperin B | GSK3B |
| glyasperin B | HSP90AB1 |
| glyasperin B | PRSS1 |
| glyasperin B | PIM1 |
| glyasperin B | CCNA2 |
| glyasperin B | NCOA2 |
| glyasperin B | CALM1 |
| glyasperin F | NOS2 |
| glyasperin F | PTGS1 |
| glyasperin F | F2 |
| glyasperin F | ESR1 |
| glyasperin F | AR |
| glyasperin F | SCN5A |
| glyasperin F | PPARG |
| glyasperin F | F10 |
| glyasperin F | PTGS2 |
| glyasperin F | CA2 |
| glyasperin F | PTPN1 |
| glyasperin F | ESR2 |
| glyasperin F | DPP4 |
| glyasperin F | MAPK14 |
| glyasperin F | GSK3B |
| glyasperin F | HSP90AB1 |
| glyasperin F | PRSS1 |
| glyasperin F | PIM1 |
| glyasperin F | CCNA2 |
| glyasperin F | CALM1 |
| Glyasperin C | NOS2 |
| Glyasperin C | F2 |
| Glyasperin C | KCNH2 |
| Glyasperin C | ESR1 |
| Glyasperin C | AR |
| Glyasperin C | SCN5A |
| Glyasperin C | PPARG |
| Glyasperin C | F10 |
| Glyasperin C | PTGS2 |
| Glyasperin C | CA2 |
| Glyasperin C | RXRA |
| Glyasperin C | ACHE |
| Glyasperin C | PTPN1 |
| Glyasperin C | ESR2 |
| Glyasperin C | DPP4 |
| Glyasperin C | MAPK14 |
| Glyasperin C | GSK3B |
| Glyasperin C | HSP90AB1 |
| Glyasperin C | PRSS1 |
| Glyasperin C | PIM1 |
| Glyasperin C | CCNA2 |
| Glyasperin C | NCOA2 |
| Glyasperin C | CALM1 |
| Isotrifoliol | NOS2 |
| Isotrifoliol | ESR1 |
| Isotrifoliol | AR |
| Isotrifoliol | PTGS2 |
| Isotrifoliol | PTPN1 |
| Isotrifoliol | ESR2 |
| Isotrifoliol | MAPK14 |
| Isotrifoliol | GSK3B |
| Isotrifoliol | HSP90AB1 |
| Isotrifoliol | PIK3CG |
| Isotrifoliol | PIM1 |
| Isotrifoliol | CCNA2 |
| (E)-1-(2,4-dihydroxyphenyl)-3-(2,2-dimethylchromen-6-yl)prop-2-en-1-one | NOS2 |
| (E)-1-(2,4-dihydroxyphenyl)-3-(2,2-dimethylchromen-6-yl)prop-2-en-1-one | PTGS1 |
| (E)-1-(2,4-dihydroxyphenyl)-3-(2,2-dimethylchromen-6-yl)prop-2-en-1-one | F2 |
| (E)-1-(2,4-dihydroxyphenyl)-3-(2,2-dimethylchromen-6-yl)prop-2-en-1-one | ESR1 |
| (E)-1-(2,4-dihydroxyphenyl)-3-(2,2-dimethylchromen-6-yl)prop-2-en-1-one | AR |
| (E)-1-(2,4-dihydroxyphenyl)-3-(2,2-dimethylchromen-6-yl)prop-2-en-1-one | SCN5A |
| (E)-1-(2,4-dihydroxyphenyl)-3-(2,2-dimethylchromen-6-yl)prop-2-en-1-one | PPARG |
| (E)-1-(2,4-dihydroxyphenyl)-3-(2,2-dimethylchromen-6-yl)prop-2-en-1-one | F10 |
| (E)-1-(2,4-dihydroxyphenyl)-3-(2,2-dimethylchromen-6-yl)prop-2-en-1-one | PTGS2 |
| (E)-1-(2,4-dihydroxyphenyl)-3-(2,2-dimethylchromen-6-yl)prop-2-en-1-one | CA2 |
| (E)-1-(2,4-dihydroxyphenyl)-3-(2,2-dimethylchromen-6-yl)prop-2-en-1-one | RXRA |
| (E)-1-(2,4-dihydroxyphenyl)-3-(2,2-dimethylchromen-6-yl)prop-2-en-1-one | ACHE |
| (E)-1-(2,4-dihydroxyphenyl)-3-(2,2-dimethylchromen-6-yl)prop-2-en-1-one | ADRA1B |
| (E)-1-(2,4-dihydroxyphenyl)-3-(2,2-dimethylchromen-6-yl)prop-2-en-1-one | ESR2 |
| (E)-1-(2,4-dihydroxyphenyl)-3-(2,2-dimethylchromen-6-yl)prop-2-en-1-one | DPP4 |
| (E)-1-(2,4-dihydroxyphenyl)-3-(2,2-dimethylchromen-6-yl)prop-2-en-1-one | MAPK14 |
| (E)-1-(2,4-dihydroxyphenyl)-3-(2,2-dimethylchromen-6-yl)prop-2-en-1-one | GSK3B |
| (E)-1-(2,4-dihydroxyphenyl)-3-(2,2-dimethylchromen-6-yl)prop-2-en-1-one | PRSS1 |
| (E)-1-(2,4-dihydroxyphenyl)-3-(2,2-dimethylchromen-6-yl)prop-2-en-1-one | PIM1 |
| (E)-1-(2,4-dihydroxyphenyl)-3-(2,2-dimethylchromen-6-yl)prop-2-en-1-one | CCNA2 |
| (E)-1-(2,4-dihydroxyphenyl)-3-(2,2-dimethylchromen-6-yl)prop-2-en-1-one | NCOA2 |
| (E)-1-(2,4-dihydroxyphenyl)-3-(2,2-dimethylchromen-6-yl)prop-2-en-1-one | CALM1 |
| kanzonols W | NOS2 |
| kanzonols W | PTGS1 |
| kanzonols W | F2 |
| kanzonols W | ESR1 |
| kanzonols W | AR |
| kanzonols W | SCN5A |
| kanzonols W | PPARG |
| kanzonols W | F10 |
| kanzonols W | PTGS2 |
| kanzonols W | CA2 |
| kanzonols W | RXRA |
| kanzonols W | ACHE |
| kanzonols W | PTPN1 |
| kanzonols W | ESR2 |
| kanzonols W | MAPK14 |
| kanzonols W | GSK3B |
| kanzonols W | PRSS1 |
| kanzonols W | PIM1 |
| kanzonols W | CCNA2 |
| kanzonols W | NCOA2 |
| kanzonols W | NCOA1 |
| kanzonols W | CALM1 |
| (2S)-6-(2,4-dihydroxyphenyl)-2-(2-hydroxypropan-2-yl)-4-methoxy-2,3-dihydrofuro[3,2-g]chromen-7-one | NOS2 |
| (2S)-6-(2,4-dihydroxyphenyl)-2-(2-hydroxypropan-2-yl)-4-methoxy-2,3-dihydrofuro[3,2-g]chromen-7-one | F2 |
| (2S)-6-(2,4-dihydroxyphenyl)-2-(2-hydroxypropan-2-yl)-4-methoxy-2,3-dihydrofuro[3,2-g]chromen-7-one | ESR1 |
| (2S)-6-(2,4-dihydroxyphenyl)-2-(2-hydroxypropan-2-yl)-4-methoxy-2,3-dihydrofuro[3,2-g]chromen-7-one | AR |
| (2S)-6-(2,4-dihydroxyphenyl)-2-(2-hydroxypropan-2-yl)-4-methoxy-2,3-dihydrofuro[3,2-g]chromen-7-one | PPARG |
| (2S)-6-(2,4-dihydroxyphenyl)-2-(2-hydroxypropan-2-yl)-4-methoxy-2,3-dihydrofuro[3,2-g]chromen-7-one | F10 |
| (2S)-6-(2,4-dihydroxyphenyl)-2-(2-hydroxypropan-2-yl)-4-methoxy-2,3-dihydrofuro[3,2-g]chromen-7-one | PTGS2 |
| (2S)-6-(2,4-dihydroxyphenyl)-2-(2-hydroxypropan-2-yl)-4-methoxy-2,3-dihydrofuro[3,2-g]chromen-7-one | CA2 |
| (2S)-6-(2,4-dihydroxyphenyl)-2-(2-hydroxypropan-2-yl)-4-methoxy-2,3-dihydrofuro[3,2-g]chromen-7-one | F7 |
| (2S)-6-(2,4-dihydroxyphenyl)-2-(2-hydroxypropan-2-yl)-4-methoxy-2,3-dihydrofuro[3,2-g]chromen-7-one | KDR |
| (2S)-6-(2,4-dihydroxyphenyl)-2-(2-hydroxypropan-2-yl)-4-methoxy-2,3-dihydrofuro[3,2-g]chromen-7-one | ACHE |
| (2S)-6-(2,4-dihydroxyphenyl)-2-(2-hydroxypropan-2-yl)-4-methoxy-2,3-dihydrofuro[3,2-g]chromen-7-one | PTPN1 |
| (2S)-6-(2,4-dihydroxyphenyl)-2-(2-hydroxypropan-2-yl)-4-methoxy-2,3-dihydrofuro[3,2-g]chromen-7-one | ESR2 |
| (2S)-6-(2,4-dihydroxyphenyl)-2-(2-hydroxypropan-2-yl)-4-methoxy-2,3-dihydrofuro[3,2-g]chromen-7-one | DPP4 |
| (2S)-6-(2,4-dihydroxyphenyl)-2-(2-hydroxypropan-2-yl)-4-methoxy-2,3-dihydrofuro[3,2-g]chromen-7-one | MAPK14 |
| (2S)-6-(2,4-dihydroxyphenyl)-2-(2-hydroxypropan-2-yl)-4-methoxy-2,3-dihydrofuro[3,2-g]chromen-7-one | GSK3B |
| (2S)-6-(2,4-dihydroxyphenyl)-2-(2-hydroxypropan-2-yl)-4-methoxy-2,3-dihydrofuro[3,2-g]chromen-7-one | PRSS1 |
| (2S)-6-(2,4-dihydroxyphenyl)-2-(2-hydroxypropan-2-yl)-4-methoxy-2,3-dihydrofuro[3,2-g]chromen-7-one | PIM1 |
| (2S)-6-(2,4-dihydroxyphenyl)-2-(2-hydroxypropan-2-yl)-4-methoxy-2,3-dihydrofuro[3,2-g]chromen-7-one | CCNA2 |
| (2S)-6-(2,4-dihydroxyphenyl)-2-(2-hydroxypropan-2-yl)-4-methoxy-2,3-dihydrofuro[3,2-g]chromen-7-one | CALM1 |
| Semilicoisoflavone B | NOS2 |
| Semilicoisoflavone B | F2 |
| Semilicoisoflavone B | ESR1 |
| Semilicoisoflavone B | AR |
| Semilicoisoflavone B | SCN5A |
| Semilicoisoflavone B | PPARG |
| Semilicoisoflavone B | F10 |
| Semilicoisoflavone B | PTGS2 |
| Semilicoisoflavone B | CA2 |
| Semilicoisoflavone B | F7 |
| Semilicoisoflavone B | ACHE |
| Semilicoisoflavone B | PTPN1 |
| Semilicoisoflavone B | GSK3B |
| Semilicoisoflavone B | HSP90AB1 |
| Semilicoisoflavone B | PRSS1 |
| Semilicoisoflavone B | PIM1 |
| Semilicoisoflavone B | CCNA2 |
| Semilicoisoflavone B | CALM1 |
| Glepidotin A | NOS2 |
| Glepidotin A | PTGS1 |
| Glepidotin A | F2 |
| Glepidotin A | ESR1 |
| Glepidotin A | AR |
| Glepidotin A | SCN5A |
| Glepidotin A | PPARG |
| Glepidotin A | F10 |
| Glepidotin A | PTGS2 |
| Glepidotin A | NOS3 |
| Glepidotin A | CA2 |
| Glepidotin A | F7 |
| Glepidotin A | KDR |
| Glepidotin A | RXRA |
| Glepidotin A | PDE3A |
| Glepidotin A | PTPN1 |
| Glepidotin A | ESR2 |
| Glepidotin A | DPP4 |
| Glepidotin A | MAPK14 |
| Glepidotin A | GSK3B |
| Glepidotin A | HSP90AB1 |
| Glepidotin A | PRSS1 |
| Glepidotin A | PIM1 |
| Glepidotin A | CCNA2 |
| Glepidotin A | CALM1 |
| Glepidotin B | NOS2 |
| Glepidotin B | PTGS1 |
| Glepidotin B | F2 |
| Glepidotin B | ESR1 |
| Glepidotin B | AR |
| Glepidotin B | SCN5A |
| Glepidotin B | PPARG |
| Glepidotin B | F10 |
| Glepidotin B | PTGS2 |
| Glepidotin B | NOS3 |
| Glepidotin B | CA2 |
| Glepidotin B | F7 |
| Glepidotin B | RXRA |
| Glepidotin B | PDE3A |
| Glepidotin B | ADRA1B |
| Glepidotin B | PTPN1 |
| Glepidotin B | ESR2 |
| Glepidotin B | DPP4 |
| Glepidotin B | MAPK14 |
| Glepidotin B | GSK3B |
| Glepidotin B | HSP90AB1 |
| Glepidotin B | PRSS1 |
| Glepidotin B | PIM1 |
| Glepidotin B | NCOA1 |
| Glepidotin B | CALM1 |
| Phaseolinisoflavan | NOS2 |
| Phaseolinisoflavan | F2 |
| Phaseolinisoflavan | CHRM1 |
| Phaseolinisoflavan | ESR1 |
| Phaseolinisoflavan | AR |
| Phaseolinisoflavan | SCN5A |
| Phaseolinisoflavan | PPARG |
| Phaseolinisoflavan | F10 |
| Phaseolinisoflavan | PTGS2 |
| Phaseolinisoflavan | CA2 |
| Phaseolinisoflavan | RXRA |
| Phaseolinisoflavan | ACHE |
| Phaseolinisoflavan | ADRA1B |
| Phaseolinisoflavan | PTPN1 |
| Phaseolinisoflavan | ADRB2 |
| Phaseolinisoflavan | ESR2 |
| Phaseolinisoflavan | DPP4 |
| Phaseolinisoflavan | MAPK14 |
| Phaseolinisoflavan | GSK3B |
| Phaseolinisoflavan | PRSS1 |
| Phaseolinisoflavan | PIM1 |
| Phaseolinisoflavan | CCNA2 |
| Phaseolinisoflavan | NCOA1 |
| Phaseolinisoflavan | CALM1 |
| Glypallichalcone | NOS2 |
| Glypallichalcone | PTGS1 |
| Glypallichalcone | F2 |
| Glypallichalcone | CHRM1 |
| Glypallichalcone | ESR1 |
| Glypallichalcone | AR |
| Glypallichalcone | SCN5A |
| Glypallichalcone | PPARG |
| Glypallichalcone | PTGS2 |
| Glypallichalcone | CA2 |
| Glypallichalcone | PDE3A |
| Glypallichalcone | ADRA1B |
| Glypallichalcone | SLC6A3 |
| Glypallichalcone | ADRB2 |
| Glypallichalcone | SLC6A4 |
| Glypallichalcone | ESR2 |
| Glypallichalcone | DPP4 |
| Glypallichalcone | MAPK14 |
| Glypallichalcone | GSK3B |
| Glypallichalcone | HSP90AB1 |
| Glypallichalcone | Cell division protein kinase 2 |
| Glypallichalcone | LTA4H |
| Glypallichalcone | MAOB |
| Glypallichalcone | PRSS1 |
| Glypallichalcone | CCNA2 |
| Glypallichalcone | NCOA1 |
| Glypallichalcone | CALM1 |
| 8-(6-hydroxy-2-benzofuranyl)-2,2-dimethyl-5-chromenol | NOS2 |
| 8-(6-hydroxy-2-benzofuranyl)-2,2-dimethyl-5-chromenol | ESR1 |
| 8-(6-hydroxy-2-benzofuranyl)-2,2-dimethyl-5-chromenol | AR |
| 8-(6-hydroxy-2-benzofuranyl)-2,2-dimethyl-5-chromenol | PPARG |
| 8-(6-hydroxy-2-benzofuranyl)-2,2-dimethyl-5-chromenol | PTGS2 |
| 8-(6-hydroxy-2-benzofuranyl)-2,2-dimethyl-5-chromenol | RXRA |
| 8-(6-hydroxy-2-benzofuranyl)-2,2-dimethyl-5-chromenol | PTPN1 |
| 8-(6-hydroxy-2-benzofuranyl)-2,2-dimethyl-5-chromenol | ESR2 |
| 8-(6-hydroxy-2-benzofuranyl)-2,2-dimethyl-5-chromenol | MAPK14 |
| 8-(6-hydroxy-2-benzofuranyl)-2,2-dimethyl-5-chromenol | GSK3B |
| 8-(6-hydroxy-2-benzofuranyl)-2,2-dimethyl-5-chromenol | HSP90AB1 |
| 8-(6-hydroxy-2-benzofuranyl)-2,2-dimethyl-5-chromenol | PIK3CG |
| 8-(6-hydroxy-2-benzofuranyl)-2,2-dimethyl-5-chromenol | PIM1 |
| 8-(6-hydroxy-2-benzofuranyl)-2,2-dimethyl-5-chromenol | CCNA2 |
| Licochalcone B | NOS2 |
| Licochalcone B | PTGS1 |
| Licochalcone B | F2 |
| Licochalcone B | ESR1 |
| Licochalcone B | AR |
| Licochalcone B | PPARG |
| Licochalcone B | PTGS2 |
| Licochalcone B | CA2 |
| Licochalcone B | PDE3A |
| Licochalcone B | PTPN1 |
| Licochalcone B | ADRB2 |
| Licochalcone B | ESR2 |
| Licochalcone B | DPP4 |
| Licochalcone B | MAPK14 |
| Licochalcone B | GSK3B |
| Licochalcone B | HSP90AB1 |
| Licochalcone B | PRSS1 |
| Licochalcone B | PIM1 |
| Licochalcone B | CCNA2 |
| Licochalcone B | CALM1 |
| licochalcone G | NOS2 |
| licochalcone G | F2 |
| licochalcone G | ESR1 |
| licochalcone G | AR |
| licochalcone G | PPARG |
| licochalcone G | F10 |
| licochalcone G | PTGS2 |
| licochalcone G | KDR |
| licochalcone G | PTPN1 |
| licochalcone G | ESR2 |
| licochalcone G | DPP4 |
| licochalcone G | MAPK14 |
| licochalcone G | GSK3B |
| licochalcone G | HSP90AB1 |
| licochalcone G | PRSS1 |
| licochalcone G | PIM1 |
| licochalcone G | CCNA2 |
| licochalcone G | NCOA2 |
| licochalcone G | CALM1 |
| 3-(2,4-dihydroxyphenyl)-8-(1,1-dimethylprop-2-enyl)-7-hydroxy-5-methoxy-coumarin | NOS2 |
| 3-(2,4-dihydroxyphenyl)-8-(1,1-dimethylprop-2-enyl)-7-hydroxy-5-methoxy-coumarin | F2 |
| 3-(2,4-dihydroxyphenyl)-8-(1,1-dimethylprop-2-enyl)-7-hydroxy-5-methoxy-coumarin | KCNH2 |
| 3-(2,4-dihydroxyphenyl)-8-(1,1-dimethylprop-2-enyl)-7-hydroxy-5-methoxy-coumarin | ESR1 |
| 3-(2,4-dihydroxyphenyl)-8-(1,1-dimethylprop-2-enyl)-7-hydroxy-5-methoxy-coumarin | AR |
| 3-(2,4-dihydroxyphenyl)-8-(1,1-dimethylprop-2-enyl)-7-hydroxy-5-methoxy-coumarin | PPARG |
| 3-(2,4-dihydroxyphenyl)-8-(1,1-dimethylprop-2-enyl)-7-hydroxy-5-methoxy-coumarin | F10 |
| 3-(2,4-dihydroxyphenyl)-8-(1,1-dimethylprop-2-enyl)-7-hydroxy-5-methoxy-coumarin | PTGS2 |
| 3-(2,4-dihydroxyphenyl)-8-(1,1-dimethylprop-2-enyl)-7-hydroxy-5-methoxy-coumarin | CA2 |
| 3-(2,4-dihydroxyphenyl)-8-(1,1-dimethylprop-2-enyl)-7-hydroxy-5-methoxy-coumarin | F7 |
| 3-(2,4-dihydroxyphenyl)-8-(1,1-dimethylprop-2-enyl)-7-hydroxy-5-methoxy-coumarin | KDR |
| 3-(2,4-dihydroxyphenyl)-8-(1,1-dimethylprop-2-enyl)-7-hydroxy-5-methoxy-coumarin | PTPN1 |
| 3-(2,4-dihydroxyphenyl)-8-(1,1-dimethylprop-2-enyl)-7-hydroxy-5-methoxy-coumarin | ESR2 |
| 3-(2,4-dihydroxyphenyl)-8-(1,1-dimethylprop-2-enyl)-7-hydroxy-5-methoxy-coumarin | DPP4 |
| 3-(2,4-dihydroxyphenyl)-8-(1,1-dimethylprop-2-enyl)-7-hydroxy-5-methoxy-coumarin | MAPK14 |
| 3-(2,4-dihydroxyphenyl)-8-(1,1-dimethylprop-2-enyl)-7-hydroxy-5-methoxy-coumarin | GSK3B |
| 3-(2,4-dihydroxyphenyl)-8-(1,1-dimethylprop-2-enyl)-7-hydroxy-5-methoxy-coumarin | HSP90AB1 |
| 3-(2,4-dihydroxyphenyl)-8-(1,1-dimethylprop-2-enyl)-7-hydroxy-5-methoxy-coumarin | PRSS1 |
| 3-(2,4-dihydroxyphenyl)-8-(1,1-dimethylprop-2-enyl)-7-hydroxy-5-methoxy-coumarin | PIM1 |
| 3-(2,4-dihydroxyphenyl)-8-(1,1-dimethylprop-2-enyl)-7-hydroxy-5-methoxy-coumarin | NCOA2 |
| 3-(2,4-dihydroxyphenyl)-8-(1,1-dimethylprop-2-enyl)-7-hydroxy-5-methoxy-coumarin | NCOA1 |
| 3-(2,4-dihydroxyphenyl)-8-(1,1-dimethylprop-2-enyl)-7-hydroxy-5-methoxy-coumarin | CALM1 |
| Licoricone | NOS2 |
| Licoricone | F2 |
| Licoricone | KCNH2 |
| Licoricone | ESR1 |
| Licoricone | AR |
| Licoricone | PPARG |
| Licoricone | F10 |
| Licoricone | PTGS2 |
| Licoricone | CA2 |
| Licoricone | KDR |
| Licoricone | PTPN1 |
| Licoricone | PRSS1 |
| Licoricone | PIM1 |
| Licoricone | NCOA2 |
| Licoricone | CALM1 |
| Gancaonin A | NOS2 |
| Gancaonin A | F2 |
| Gancaonin A | ESR1 |
| Gancaonin A | AR |
| Gancaonin A | SCN5A |
| Gancaonin A | PPARG |
| Gancaonin A | F10 |
| Gancaonin A | PTGS2 |
| Gancaonin A | CA2 |
| Gancaonin A | ACHE |
| Gancaonin A | PTPN1 |
| Gancaonin A | ESR2 |
| Gancaonin A | DPP4 |
| Gancaonin A | GSK3B |
| Gancaonin A | HSP90AB1 |
| Gancaonin A | PRSS1 |
| Gancaonin A | PIM1 |
| Gancaonin A | CCNA2 |
| Gancaonin A | NCOA2 |
| Gancaonin A | CALM1 |
| Gancaonin B | NOS2 |
| Gancaonin B | F2 |
| Gancaonin B | ESR1 |
| Gancaonin B | AR |
| Gancaonin B | PPARG |
| Gancaonin B | F10 |
| Gancaonin B | PTGS2 |
| Gancaonin B | CA2 |
| Gancaonin B | F7 |
| Gancaonin B | KDR |
| Gancaonin B | ADRA1B |
| Gancaonin B | PTPN1 |
| Gancaonin B | ADRB2 |
| Gancaonin B | ESR2 |
| Gancaonin B | DPP4 |
| Gancaonin B | GSK3B |
| Gancaonin B | HSP90AB1 |
| Gancaonin B | PRSS1 |
| Gancaonin B | PIM1 |
| Gancaonin B | CCNA2 |
| Gancaonin B | NCOA2 |
| Gancaonin B | CALM1 |
| 3-(3,4-dihydroxyphenyl)-5,7-dihydroxy-8-(3-methylbut-2-enyl)chromone | NOS2 |
| 3-(3,4-dihydroxyphenyl)-5,7-dihydroxy-8-(3-methylbut-2-enyl)chromone | F2 |
| 3-(3,4-dihydroxyphenyl)-5,7-dihydroxy-8-(3-methylbut-2-enyl)chromone | ESR1 |
| 3-(3,4-dihydroxyphenyl)-5,7-dihydroxy-8-(3-methylbut-2-enyl)chromone | AR |
| 3-(3,4-dihydroxyphenyl)-5,7-dihydroxy-8-(3-methylbut-2-enyl)chromone | PPARG |
| 3-(3,4-dihydroxyphenyl)-5,7-dihydroxy-8-(3-methylbut-2-enyl)chromone | F10 |
| 3-(3,4-dihydroxyphenyl)-5,7-dihydroxy-8-(3-methylbut-2-enyl)chromone | PTGS2 |
| 3-(3,4-dihydroxyphenyl)-5,7-dihydroxy-8-(3-methylbut-2-enyl)chromone | PTPN1 |
| 3-(3,4-dihydroxyphenyl)-5,7-dihydroxy-8-(3-methylbut-2-enyl)chromone | MAPK14 |
| 3-(3,4-dihydroxyphenyl)-5,7-dihydroxy-8-(3-methylbut-2-enyl)chromone | GSK3B |
| 3-(3,4-dihydroxyphenyl)-5,7-dihydroxy-8-(3-methylbut-2-enyl)chromone | HSP90AB1 |
| 3-(3,4-dihydroxyphenyl)-5,7-dihydroxy-8-(3-methylbut-2-enyl)chromone | PRSS1 |
| 3-(3,4-dihydroxyphenyl)-5,7-dihydroxy-8-(3-methylbut-2-enyl)chromone | PIM1 |
| 3-(3,4-dihydroxyphenyl)-5,7-dihydroxy-8-(3-methylbut-2-enyl)chromone | CCNA2 |
| 3-(3,4-dihydroxyphenyl)-5,7-dihydroxy-8-(3-methylbut-2-enyl)chromone | NCOA2 |
| 3-(3,4-dihydroxyphenyl)-5,7-dihydroxy-8-(3-methylbut-2-enyl)chromone | CALM1 |
| 5,7-dihydroxy-3-(4-methoxyphenyl)-8-(3-methylbut-2-enyl)chromone | NOS2 |
| 5,7-dihydroxy-3-(4-methoxyphenyl)-8-(3-methylbut-2-enyl)chromone | KCNH2 |
| 5,7-dihydroxy-3-(4-methoxyphenyl)-8-(3-methylbut-2-enyl)chromone | ESR1 |
| 5,7-dihydroxy-3-(4-methoxyphenyl)-8-(3-methylbut-2-enyl)chromone | AR |
| 5,7-dihydroxy-3-(4-methoxyphenyl)-8-(3-methylbut-2-enyl)chromone | PPARG |
| 5,7-dihydroxy-3-(4-methoxyphenyl)-8-(3-methylbut-2-enyl)chromone | F10 |
| 5,7-dihydroxy-3-(4-methoxyphenyl)-8-(3-methylbut-2-enyl)chromone | PTGS2 |
| 5,7-dihydroxy-3-(4-methoxyphenyl)-8-(3-methylbut-2-enyl)chromone | CA2 |
| 5,7-dihydroxy-3-(4-methoxyphenyl)-8-(3-methylbut-2-enyl)chromone | PTPN1 |
| 5,7-dihydroxy-3-(4-methoxyphenyl)-8-(3-methylbut-2-enyl)chromone | ESR2 |
| 5,7-dihydroxy-3-(4-methoxyphenyl)-8-(3-methylbut-2-enyl)chromone | DPP4 |
| 5,7-dihydroxy-3-(4-methoxyphenyl)-8-(3-methylbut-2-enyl)chromone | MAPK14 |
| 5,7-dihydroxy-3-(4-methoxyphenyl)-8-(3-methylbut-2-enyl)chromone | GSK3B |
| 5,7-dihydroxy-3-(4-methoxyphenyl)-8-(3-methylbut-2-enyl)chromone | HSP90AB1 |
| 5,7-dihydroxy-3-(4-methoxyphenyl)-8-(3-methylbut-2-enyl)chromone | PRSS1 |
| 5,7-dihydroxy-3-(4-methoxyphenyl)-8-(3-methylbut-2-enyl)chromone | PIM1 |
| 5,7-dihydroxy-3-(4-methoxyphenyl)-8-(3-methylbut-2-enyl)chromone | CCNA2 |
| 5,7-dihydroxy-3-(4-methoxyphenyl)-8-(3-methylbut-2-enyl)chromone | NCOA2 |
| 5,7-dihydroxy-3-(4-methoxyphenyl)-8-(3-methylbut-2-enyl)chromone | CALM1 |
| 2-(3,4-dihydroxyphenyl)-5,7-dihydroxy-6-(3-methylbut-2-enyl)chromone | NOS2 |
| 2-(3,4-dihydroxyphenyl)-5,7-dihydroxy-6-(3-methylbut-2-enyl)chromone | F2 |
| 2-(3,4-dihydroxyphenyl)-5,7-dihydroxy-6-(3-methylbut-2-enyl)chromone | ESR1 |
| 2-(3,4-dihydroxyphenyl)-5,7-dihydroxy-6-(3-methylbut-2-enyl)chromone | AR |
| 2-(3,4-dihydroxyphenyl)-5,7-dihydroxy-6-(3-methylbut-2-enyl)chromone | SCN5A |
| 2-(3,4-dihydroxyphenyl)-5,7-dihydroxy-6-(3-methylbut-2-enyl)chromone | PPARG |
| 2-(3,4-dihydroxyphenyl)-5,7-dihydroxy-6-(3-methylbut-2-enyl)chromone | F10 |
| 2-(3,4-dihydroxyphenyl)-5,7-dihydroxy-6-(3-methylbut-2-enyl)chromone | PTGS2 |
| 2-(3,4-dihydroxyphenyl)-5,7-dihydroxy-6-(3-methylbut-2-enyl)chromone | CA2 |
| 2-(3,4-dihydroxyphenyl)-5,7-dihydroxy-6-(3-methylbut-2-enyl)chromone | F7 |
| 2-(3,4-dihydroxyphenyl)-5,7-dihydroxy-6-(3-methylbut-2-enyl)chromone | PTPN1 |
| 2-(3,4-dihydroxyphenyl)-5,7-dihydroxy-6-(3-methylbut-2-enyl)chromone | ADRB2 |
| 2-(3,4-dihydroxyphenyl)-5,7-dihydroxy-6-(3-methylbut-2-enyl)chromone | DPP4 |
| 2-(3,4-dihydroxyphenyl)-5,7-dihydroxy-6-(3-methylbut-2-enyl)chromone | GSK3B |
| 2-(3,4-dihydroxyphenyl)-5,7-dihydroxy-6-(3-methylbut-2-enyl)chromone | HSP90AB1 |
| 2-(3,4-dihydroxyphenyl)-5,7-dihydroxy-6-(3-methylbut-2-enyl)chromone | PRSS1 |
| 2-(3,4-dihydroxyphenyl)-5,7-dihydroxy-6-(3-methylbut-2-enyl)chromone | PIM1 |
| 2-(3,4-dihydroxyphenyl)-5,7-dihydroxy-6-(3-methylbut-2-enyl)chromone | CCNA2 |
| 2-(3,4-dihydroxyphenyl)-5,7-dihydroxy-6-(3-methylbut-2-enyl)chromone | CALM1 |
| Glycyrin | NOS2 |
| Glycyrin | F2 |
| Glycyrin | KCNH2 |
| Glycyrin | ESR1 |
| Glycyrin | AR |
| Glycyrin | PPARG |
| Glycyrin | F10 |
| Glycyrin | PTGS2 |
| Glycyrin | CA2 |
| Glycyrin | KDR |
| Glycyrin | ESR2 |
| Glycyrin | DPP4 |
| Glycyrin | PRSS1 |
| Glycyrin | PIM1 |
| Glycyrin | NCOA2 |
| Glycyrin | CALM1 |
| Licocoumarone | ESR1 |
| Licocoumarone | AR |
| Licocoumarone | PPARG |
| Licocoumarone | PTPN1 |
| Licocoumarone | ESR2 |
| Licocoumarone | MAPK14 |
| Licocoumarone | GSK3B |
| Licocoumarone | HSP90AB1 |
| Licocoumarone | CCNA2 |
| Licoisoflavone | NOS2 |
| Licoisoflavone | F2 |
| Licoisoflavone | ESR1 |
| Licoisoflavone | AR |
| Licoisoflavone | PPARG |
| Licoisoflavone | F10 |
| Licoisoflavone | PTGS2 |
| Licoisoflavone | KDR |
| Licoisoflavone | PTPN1 |
| Licoisoflavone | DPP4 |
| Licoisoflavone | MAPK14 |
| Licoisoflavone | HSP90AB1 |
| Licoisoflavone | PRSS1 |
| Licoisoflavone | PIM1 |
| Licoisoflavone | CCNA2 |
| Licoisoflavone | NCOA2 |
| Licoisoflavone | CALM1 |
| Licoisoflavone B | NOS2 |
| Licoisoflavone B | F2 |
| Licoisoflavone B | ESR1 |
| Licoisoflavone B | AR |
| Licoisoflavone B | PPARG |
| Licoisoflavone B | F10 |
| Licoisoflavone B | PTGS2 |
| Licoisoflavone B | CA2 |
| Licoisoflavone B | ACHE |
| Licoisoflavone B | PTPN1 |
| Licoisoflavone B | ESR2 |
| Licoisoflavone B | GSK3B |
| Licoisoflavone B | PRSS1 |
| Licoisoflavone B | PIM1 |
| Licoisoflavone B | CCNA2 |
| Licoisoflavone B | CALM1 |
| licoisoflavanone | NOS2 |
| licoisoflavanone | PTGS1 |
| licoisoflavanone | F2 |
| licoisoflavanone | ESR1 |
| licoisoflavanone | AR |
| licoisoflavanone | SCN5A |
| licoisoflavanone | PPARG |
| licoisoflavanone | F10 |
| licoisoflavanone | PTGS2 |
| licoisoflavanone | CA2 |
| licoisoflavanone | F7 |
| licoisoflavanone | ACHE |
| licoisoflavanone | PTPN1 |
| licoisoflavanone | ESR2 |
| licoisoflavanone | GSK3B |
| licoisoflavanone | HSP90AB1 |
| licoisoflavanone | PRSS1 |
| licoisoflavanone | PIM1 |
| licoisoflavanone | CCNA2 |
| licoisoflavanone | NCOA1 |
| licoisoflavanone | CALM1 |
| shinpterocarpin | NOS2 |
| shinpterocarpin | PTGS1 |
| shinpterocarpin | CHRM3 |
| shinpterocarpin | F2 |
| shinpterocarpin | KCNH2 |
| shinpterocarpin | CHRM1 |
| shinpterocarpin | ESR1 |
| shinpterocarpin | AR |
| shinpterocarpin | SCN5A |
| shinpterocarpin | PPARG |
| shinpterocarpin | PTGS2 |
| shinpterocarpin | HTR3A |
| shinpterocarpin | CA2 |
| shinpterocarpin | RXRA |
| shinpterocarpin | OPRD1 |
| shinpterocarpin | ACHE |
| shinpterocarpin | ADRA1B |
| shinpterocarpin | PTPN1 |
| shinpterocarpin | ADRB2 |
| shinpterocarpin | ADRA1D |
| shinpterocarpin | OPRM1 |
| shinpterocarpin | ESR2 |
| shinpterocarpin | DPP4 |
| shinpterocarpin | MAPK14 |
| shinpterocarpin | GSK3B |
| shinpterocarpin | PIK3CG |
| shinpterocarpin | PRSS1 |
| shinpterocarpin | PIM1 |
| shinpterocarpin | CCNA2 |
| shinpterocarpin | NCOA1 |
| shinpterocarpin | CALM1 |
| (E)-3-[3,4-dihydroxy-5-(3-methylbut-2-enyl)phenyl]-1-(2,4-dihydroxyphenyl)prop-2-en-1-one | NOS2 |
| (E)-3-[3,4-dihydroxy-5-(3-methylbut-2-enyl)phenyl]-1-(2,4-dihydroxyphenyl)prop-2-en-1-one | F2 |
| (E)-3-[3,4-dihydroxy-5-(3-methylbut-2-enyl)phenyl]-1-(2,4-dihydroxyphenyl)prop-2-en-1-one | ESR1 |
| (E)-3-[3,4-dihydroxy-5-(3-methylbut-2-enyl)phenyl]-1-(2,4-dihydroxyphenyl)prop-2-en-1-one | AR |
| (E)-3-[3,4-dihydroxy-5-(3-methylbut-2-enyl)phenyl]-1-(2,4-dihydroxyphenyl)prop-2-en-1-one | PPARG |
| (E)-3-[3,4-dihydroxy-5-(3-methylbut-2-enyl)phenyl]-1-(2,4-dihydroxyphenyl)prop-2-en-1-one | PTGS2 |
| (E)-3-[3,4-dihydroxy-5-(3-methylbut-2-enyl)phenyl]-1-(2,4-dihydroxyphenyl)prop-2-en-1-one | PTPN1 |
| (E)-3-[3,4-dihydroxy-5-(3-methylbut-2-enyl)phenyl]-1-(2,4-dihydroxyphenyl)prop-2-en-1-one | ESR2 |
| (E)-3-[3,4-dihydroxy-5-(3-methylbut-2-enyl)phenyl]-1-(2,4-dihydroxyphenyl)prop-2-en-1-one | DPP4 |
| (E)-3-[3,4-dihydroxy-5-(3-methylbut-2-enyl)phenyl]-1-(2,4-dihydroxyphenyl)prop-2-en-1-one | MAPK14 |
| (E)-3-[3,4-dihydroxy-5-(3-methylbut-2-enyl)phenyl]-1-(2,4-dihydroxyphenyl)prop-2-en-1-one | GSK3B |
| (E)-3-[3,4-dihydroxy-5-(3-methylbut-2-enyl)phenyl]-1-(2,4-dihydroxyphenyl)prop-2-en-1-one | HSP90AB1 |
| (E)-3-[3,4-dihydroxy-5-(3-methylbut-2-enyl)phenyl]-1-(2,4-dihydroxyphenyl)prop-2-en-1-one | PRSS1 |
| (E)-3-[3,4-dihydroxy-5-(3-methylbut-2-enyl)phenyl]-1-(2,4-dihydroxyphenyl)prop-2-en-1-one | PIM1 |
| (E)-3-[3,4-dihydroxy-5-(3-methylbut-2-enyl)phenyl]-1-(2,4-dihydroxyphenyl)prop-2-en-1-one | CCNA2 |
| (E)-3-[3,4-dihydroxy-5-(3-methylbut-2-enyl)phenyl]-1-(2,4-dihydroxyphenyl)prop-2-en-1-one | NCOA2 |
| (E)-3-[3,4-dihydroxy-5-(3-methylbut-2-enyl)phenyl]-1-(2,4-dihydroxyphenyl)prop-2-en-1-one | CALM1 |
| liquiritin | SOD1 |
| liquiritin | NOS2 |
| liquiritin | F2 |
| liquiritin | ESR1 |
| liquiritin | AR |
| liquiritin | PPARG |
| liquiritin | F10 |
| liquiritin | PTGS2 |
| liquiritin | CA2 |
| liquiritin | F7 |
| liquiritin | KDR |
| liquiritin | DPP4 |
| liquiritin | PIM1 |
| liquiritin | CCNA2 |
| liquiritin | CALM1 |
| licopyranocoumarin | NOS2 |
| licopyranocoumarin | F2 |
| licopyranocoumarin | ESR1 |
| licopyranocoumarin | AR |
| licopyranocoumarin | PPARG |
| licopyranocoumarin | F10 |
| licopyranocoumarin | PTGS2 |
| licopyranocoumarin | CA2 |
| licopyranocoumarin | F7 |
| licopyranocoumarin | KDR |
| licopyranocoumarin | ACHE |
| licopyranocoumarin | PTPN1 |
| licopyranocoumarin | PRSS1 |
| licopyranocoumarin | PIM1 |
| licopyranocoumarin | CCNA2 |
| licopyranocoumarin | CALM1 |
| G101aglabrin | NOS2 |
| G101aglabrin | PTGS1 |
| G101aglabrin | ESR1 |
| G101aglabrin | AR |
| G101aglabrin | PPARG |
| G101aglabrin | PTGS2 |
| G101aglabrin | CA2 |
| G101aglabrin | PTPN1 |
| G101aglabrin | ESR2 |
| G101aglabrin | DPP4 |
| G101aglabrin | MAPK14 |
| G101aglabrin | GSK3B |
| G101aglabrin | HSP90AB1 |
| G101aglabrin | PIK3CG |
| G101aglabrin | PRSS1 |
| G101aglabrin | PIM1 |
| G101aglabrin | CCNA2 |
| Glabridin | NOS2 |
| Glabridin | F2 |
| Glabridin | CHRM1 |
| Glabridin | ESR1 |
| Glabridin | AR |
| Glabridin | SCN5A |
| Glabridin | PPARG |
| Glabridin | PTGS2 |
| Glabridin | CA2 |
| Glabridin | RXRA |
| Glabridin | ACHE |
| Glabridin | ADRA1B |
| Glabridin | PTPN1 |
| Glabridin | ADRB2 |
| Glabridin | ESR2 |
| Glabridin | DPP4 |
| Glabridin | MAPK14 |
| Glabridin | GSK3B |
| Glabridin | PRSS1 |
| Glabridin | PIM1 |
| Glabridin | CCNA2 |
| Glabridin | NCOA2 |
| Glabridin | NCOA1 |
| Glabridin | CALM1 |
| Glabranin | NOS2 |
| Glabranin | PTGS1 |
| Glabranin | F2 |
| Glabranin | ESR1 |
| Glabranin | AR |
| Glabranin | SCN5A |
| Glabranin | PPARG |
| Glabranin | F10 |
| Glabranin | PTGS2 |
| Glabranin | NOS3 |
| Glabranin | PDE3A |
| Glabranin | PTPN1 |
| Glabranin | ESR2 |
| Glabranin | DPP4 |
| Glabranin | MAPK14 |
| Glabranin | GSK3B |
| Glabranin | HSP90AB1 |
| Glabranin | PRSS1 |
| Glabranin | PIM1 |
| Glabranin | CCNA2 |
| Glabranin | CALM1 |
| Glabrene | NOS2 |
| Glabrene | PTGS1 |
| Glabrene | F2 |
| Glabrene | ESR1 |
| Glabrene | AR |
| Glabrene | SCN5A |
| Glabrene | PPARG |
| Glabrene | F10 |
| Glabrene | PTGS2 |
| Glabrene | CA2 |
| Glabrene | RXRA |
| Glabrene | PTPN1 |
| Glabrene | ADRB2 |
| Glabrene | ESR2 |
| Glabrene | DPP4 |
| Glabrene | MAPK14 |
| Glabrene | GSK3B |
| Glabrene | HSP90AB1 |
| Glabrene | PRSS1 |
| Glabrene | PIM1 |
| Glabrene | NCOA2 |
| Glabrene | CALM1 |
| Glabrone | NOS2 |
| Glabrone | PTGS1 |
| Glabrone | F2 |
| Glabrone | ESR1 |
| Glabrone | AR |
| Glabrone | SCN5A |
| Glabrone | PPARG |
| Glabrone | F10 |
| Glabrone | PTGS2 |
| Glabrone | CA2 |
| Glabrone | RXRA |
| Glabrone | ACHE |
| Glabrone | PTPN1 |
| Glabrone | ESR2 |
| Glabrone | DPP4 |
| Glabrone | MAPK14 |
| Glabrone | GSK3B |
| Glabrone | PRSS1 |
| Glabrone | PIM1 |
| Glabrone | CCNA2 |
| Glabrone | CALM1 |
| 1,3-dihydroxy-9-methoxy-6-benzofurano[3,2-c]chromenone | ESR1 |
| 1,3-dihydroxy-9-methoxy-6-benzofurano[3,2-c]chromenone | PPARG |
| 1,3-dihydroxy-9-methoxy-6-benzofurano[3,2-c]chromenone | PTPN1 |
| 1,3-dihydroxy-9-methoxy-6-benzofurano[3,2-c]chromenone | ESR2 |
| 1,3-dihydroxy-9-methoxy-6-benzofurano[3,2-c]chromenone | MAPK14 |
| 1,3-dihydroxy-9-methoxy-6-benzofurano[3,2-c]chromenone | GSK3B |
| 1,3-dihydroxy-9-methoxy-6-benzofurano[3,2-c]chromenone | HSP90AB1 |
| 1,3-dihydroxy-9-methoxy-6-benzofurano[3,2-c]chromenone | CCNA2 |
| 1,3-dihydroxy-9-methoxy-6-benzofurano[3,2-c]chromenone | ESR1 |
| 1,3-dihydroxy-9-methoxy-6-benzofurano[3,2-c]chromenone | AR |
| 1,3-dihydroxy-9-methoxy-6-benzofurano[3,2-c]chromenone | PPARG |
| 1,3-dihydroxy-9-methoxy-6-benzofurano[3,2-c]chromenone | PTPN1 |
| 1,3-dihydroxy-9-methoxy-6-benzofurano[3,2-c]chromenone | ESR2 |
| 1,3-dihydroxy-9-methoxy-6-benzofurano[3,2-c]chromenone | MAPK14 |
| 1,3-dihydroxy-9-methoxy-6-benzofurano[3,2-c]chromenone | GSK3B |
| 1,3-dihydroxy-9-methoxy-6-benzofurano[3,2-c]chromenone | HSP90AB1 |
| 1,3-dihydroxy-9-methoxy-6-benzofurano[3,2-c]chromenone | CCNA2 |
| Eurycarpin A | NOS2 |
| Eurycarpin A | F2 |
| Eurycarpin A | ESR1 |
| Eurycarpin A | AR |
| Eurycarpin A | SCN5A |
| Eurycarpin A | PPARG |
| Eurycarpin A | F10 |
| Eurycarpin A | PTGS2 |
| Eurycarpin A | PTPN1 |
| Eurycarpin A | ESR2 |
| Eurycarpin A | DPP4 |
| Eurycarpin A | MAPK14 |
| Eurycarpin A | GSK3B |
| Eurycarpin A | HSP90AB1 |
| Eurycarpin A | Cell division protein kinase 2 |
| Eurycarpin A | CHEK1 |
| Eurycarpin A | PRSS1 |
| Eurycarpin A | PIM1 |
| Eurycarpin A | CCNA2 |
| Eurycarpin A | CALM1 |
| (-)-Medicocarpin | ESR1 |
| (-)-Medicocarpin | AR |
| (-)-Medicocarpin | PPARG |
| (-)-Medicocarpin | PTGS2 |
| (-)-Medicocarpin | CA2 |
| (-)-Medicocarpin | ACHE |
| (-)-Medicocarpin | CCNA2 |
| Sigmoidin-B | NOS2 |
| Sigmoidin-B | F2 |
| Sigmoidin-B | ESR1 |
| Sigmoidin-B | AR |
| Sigmoidin-B | PPARG |
| Sigmoidin-B | F10 |
| Sigmoidin-B | PTGS2 |
| Sigmoidin-B | KDR |
| Sigmoidin-B | PTPN1 |
| Sigmoidin-B | ESR2 |
| Sigmoidin-B | DPP4 |
| Sigmoidin-B | MAPK14 |
| Sigmoidin-B | GSK3B |
| Sigmoidin-B | HSP90AB1 |
| Sigmoidin-B | Cell division protein kinase 2 |
| Sigmoidin-B | PRSS1 |
| Sigmoidin-B | PIM1 |
| Sigmoidin-B | CCNA2 |
| Sigmoidin-B | CALM1 |
| (2R)-7-hydroxy-2-(4-hydroxyphenyl)chroman-4-one | NOS2 |
| (2R)-7-hydroxy-2-(4-hydroxyphenyl)chroman-4-one | PTGS1 |
| (2R)-7-hydroxy-2-(4-hydroxyphenyl)chroman-4-one | ESR1 |
| (2R)-7-hydroxy-2-(4-hydroxyphenyl)chroman-4-one | AR |
| (2R)-7-hydroxy-2-(4-hydroxyphenyl)chroman-4-one | PPARG |
| (2R)-7-hydroxy-2-(4-hydroxyphenyl)chroman-4-one | PTGS2 |
| (2R)-7-hydroxy-2-(4-hydroxyphenyl)chroman-4-one | CA2 |
| (2R)-7-hydroxy-2-(4-hydroxyphenyl)chroman-4-one | RXRA |
| (2R)-7-hydroxy-2-(4-hydroxyphenyl)chroman-4-one | PDE3A |
| (2R)-7-hydroxy-2-(4-hydroxyphenyl)chroman-4-one | PTPN1 |
| (2R)-7-hydroxy-2-(4-hydroxyphenyl)chroman-4-one | ADRB2 |
| (2R)-7-hydroxy-2-(4-hydroxyphenyl)chroman-4-one | SLC6A4 |
| (2R)-7-hydroxy-2-(4-hydroxyphenyl)chroman-4-one | ESR2 |
| (2R)-7-hydroxy-2-(4-hydroxyphenyl)chroman-4-one | DPP4 |
| (2R)-7-hydroxy-2-(4-hydroxyphenyl)chroman-4-one | MAPK14 |
| (2R)-7-hydroxy-2-(4-hydroxyphenyl)chroman-4-one | GSK3B |
| (2R)-7-hydroxy-2-(4-hydroxyphenyl)chroman-4-one | HSP90AB1 |
| (2R)-7-hydroxy-2-(4-hydroxyphenyl)chroman-4-one | PIK3CG |
| (2R)-7-hydroxy-2-(4-hydroxyphenyl)chroman-4-one | MAOB |
| (2R)-7-hydroxy-2-(4-hydroxyphenyl)chroman-4-one | PIM1 |
| (2R)-7-hydroxy-2-(4-hydroxyphenyl)chroman-4-one | CCNA2 |
| (2R)-7-hydroxy-2-(4-hydroxyphenyl)chroman-4-one | CALM1 |
| (2R)-7-hydroxy-2-(4-hydroxyphenyl)chroman-4-one | NOS2 |
| (2R)-7-hydroxy-2-(4-hydroxyphenyl)chroman-4-one | PTGS1 |
| (2R)-7-hydroxy-2-(4-hydroxyphenyl)chroman-4-one | F2 |
| (2R)-7-hydroxy-2-(4-hydroxyphenyl)chroman-4-one | ESR1 |
| (2R)-7-hydroxy-2-(4-hydroxyphenyl)chroman-4-one | AR |
| (2R)-7-hydroxy-2-(4-hydroxyphenyl)chroman-4-one | SCN5A |
| (2R)-7-hydroxy-2-(4-hydroxyphenyl)chroman-4-one | PPARG |
| (2R)-7-hydroxy-2-(4-hydroxyphenyl)chroman-4-one | F10 |
| (2R)-7-hydroxy-2-(4-hydroxyphenyl)chroman-4-one | PTGS2 |
| (2R)-7-hydroxy-2-(4-hydroxyphenyl)chroman-4-one | CA2 |
| (2R)-7-hydroxy-2-(4-hydroxyphenyl)chroman-4-one | PDE3A |
| (2R)-7-hydroxy-2-(4-hydroxyphenyl)chroman-4-one | ADRA1B |
| (2R)-7-hydroxy-2-(4-hydroxyphenyl)chroman-4-one | PTPN1 |
| (2R)-7-hydroxy-2-(4-hydroxyphenyl)chroman-4-one | ADRB2 |
| (2R)-7-hydroxy-2-(4-hydroxyphenyl)chroman-4-one | ESR2 |
| (2R)-7-hydroxy-2-(4-hydroxyphenyl)chroman-4-one | DPP4 |
| (2R)-7-hydroxy-2-(4-hydroxyphenyl)chroman-4-one | MAPK14 |
| (2R)-7-hydroxy-2-(4-hydroxyphenyl)chroman-4-one | GSK3B |
| (2R)-7-hydroxy-2-(4-hydroxyphenyl)chroman-4-one | HSP90AB1 |
| (2R)-7-hydroxy-2-(4-hydroxyphenyl)chroman-4-one | PRSS1 |
| (2R)-7-hydroxy-2-(4-hydroxyphenyl)chroman-4-one | PIM1 |
| (2R)-7-hydroxy-2-(4-hydroxyphenyl)chroman-4-one | CCNA2 |
| (2R)-7-hydroxy-2-(4-hydroxyphenyl)chroman-4-one | CALM1 |
| Isoglycyrol | NOS2 |
| Isoglycyrol | ESR1 |
| Isoglycyrol | AR |
| Isoglycyrol | PTGS2 |
| Isoglycyrol | PTPN1 |
| Isoglycyrol | DPP4 |
| Isoglycyrol | GSK3B |
| Isoglycyrol | PRSS1 |
| Isoglycyrol | PIM1 |
| Isolicoflavonol | NOS2 |
| Isolicoflavonol | F2 |
| Isolicoflavonol | ESR1 |
| Isolicoflavonol | AR |
| Isolicoflavonol | PPARG |
| Isolicoflavonol | F10 |
| Isolicoflavonol | PTGS2 |
| Isolicoflavonol | CA2 |
| Isolicoflavonol | PTPN1 |
| Isolicoflavonol | ESR2 |
| Isolicoflavonol | GSK3B |
| Isolicoflavonol | HSP90AB1 |
| Isolicoflavonol | PRSS1 |
| Isolicoflavonol | PIM1 |
| Isolicoflavonol | CCNA2 |
| Isolicoflavonol | NCOA2 |
| Isolicoflavonol | CALM1 |
| HMO | NOS2 |
| HMO | PTGS1 |
| HMO | CHRM1 |
| HMO | ESR1 |
| HMO | AR |
| HMO | SCN5A |
| HMO | PPARG |
| HMO | PTGS2 |
| HMO | CA2 |
| HMO | RXRA |
| HMO | PDE3A |
| HMO | PTPN1 |
| HMO | ADRB2 |
| HMO | SLC6A4 |
| HMO | ESR2 |
| HMO | DPP4 |
| HMO | MAPK14 |
| HMO | GSK3B |
| HMO | MAOB |
| HMO | PRSS1 |
| HMO | PIM1 |
| HMO | CCNA2 |
| HMO | CALM1 |
| 1-Methoxyphaseollidin | NOS2 |
| 1-Methoxyphaseollidin | PTGS1 |
| 1-Methoxyphaseollidin | F2 |
| 1-Methoxyphaseollidin | KCNH2 |
| 1-Methoxyphaseollidin | ESR1 |
| 1-Methoxyphaseollidin | AR |
| 1-Methoxyphaseollidin | SCN5A |
| 1-Methoxyphaseollidin | PPARG |
| 1-Methoxyphaseollidin | F10 |
| 1-Methoxyphaseollidin | PTGS2 |
| 1-Methoxyphaseollidin | NOS3 |
| 1-Methoxyphaseollidin | KDR |
| 1-Methoxyphaseollidin | RXRA |
| 1-Methoxyphaseollidin | ADRA1B |
| 1-Methoxyphaseollidin | ADRB2 |
| 1-Methoxyphaseollidin | ADRA1D |
| 1-Methoxyphaseollidin | ESR2 |
| 1-Methoxyphaseollidin | DPP4 |
| 1-Methoxyphaseollidin | MAPK14 |
| 1-Methoxyphaseollidin | GSK3B |
| 1-Methoxyphaseollidin | HSP90AB1 |
| 1-Methoxyphaseollidin | PIK3CG |
| 1-Methoxyphaseollidin | PRSS1 |
| 1-Methoxyphaseollidin | PIM1 |
| 1-Methoxyphaseollidin | CCNA2 |
| 1-Methoxyphaseollidin | NCOA2 |
| 1-Methoxyphaseollidin | NCOA1 |
| 1-Methoxyphaseollidin | CALM1 |
| Quercetin der. | NOS2 |
| Quercetin der. | PTGS1 |
| Quercetin der. | ESR1 |
| Quercetin der. | AR |
| Quercetin der. | SCN5A |
| Quercetin der. | PPARG |
| Quercetin der. | PTGS2 |
| Quercetin der. | CA2 |
| Quercetin der. | PTPN1 |
| Quercetin der. | ESR2 |
| Quercetin der. | DPP4 |
| Quercetin der. | MAPK14 |
| Quercetin der. | GSK3B |
| Quercetin der. | HSP90AB1 |
| Quercetin der. | PRSS1 |
| Quercetin der. | PIM1 |
| Quercetin der. | CCNA2 |
| Quercetin der. | NCOA2 |
| Quercetin der. | CALM1 |
| 3'-Hydroxy-4'-O-Methylglabridin | NOS2 |
| 3'-Hydroxy-4'-O-Methylglabridin | PTGS1 |
| 3'-Hydroxy-4'-O-Methylglabridin | F2 |
| 3'-Hydroxy-4'-O-Methylglabridin | KCNH2 |
| 3'-Hydroxy-4'-O-Methylglabridin | ESR1 |
| 3'-Hydroxy-4'-O-Methylglabridin | AR |
| 3'-Hydroxy-4'-O-Methylglabridin | SCN5A |
| 3'-Hydroxy-4'-O-Methylglabridin | PPARG |
| 3'-Hydroxy-4'-O-Methylglabridin | F10 |
| 3'-Hydroxy-4'-O-Methylglabridin | PTGS2 |
| 3'-Hydroxy-4'-O-Methylglabridin | CA2 |
| 3'-Hydroxy-4'-O-Methylglabridin | F7 |
| 3'-Hydroxy-4'-O-Methylglabridin | KDR |
| 3'-Hydroxy-4'-O-Methylglabridin | ADRA1B |
| 3'-Hydroxy-4'-O-Methylglabridin | PTPN1 |
| 3'-Hydroxy-4'-O-Methylglabridin | ADRB2 |
| 3'-Hydroxy-4'-O-Methylglabridin | ESR2 |
| 3'-Hydroxy-4'-O-Methylglabridin | DPP4 |
| 3'-Hydroxy-4'-O-Methylglabridin | MAPK14 |
| 3'-Hydroxy-4'-O-Methylglabridin | GSK3B |
| 3'-Hydroxy-4'-O-Methylglabridin | HSP90AB1 |
| 3'-Hydroxy-4'-O-Methylglabridin | PRSS1 |
| 3'-Hydroxy-4'-O-Methylglabridin | PIM1 |
| 3'-Hydroxy-4'-O-Methylglabridin | CCNA2 |
| 3'-Hydroxy-4'-O-Methylglabridin | NCOA2 |
| 3'-Hydroxy-4'-O-Methylglabridin | NCOA1 |
| 3'-Hydroxy-4'-O-Methylglabridin | KCNMA1 |
| 3'-Hydroxy-4'-O-Methylglabridin | CALM1 |
| 3'-Methoxyglabridin | NOS2 |
| 3'-Mehoxyglabridin | PTGS1 |
| 3'-Mehoxyglabridin | F2 |
| 3'-Mehoxyglabridin | KCNH2 |
| 3'-Mehoxyglabridin | ESR1 |
| 3'-Mehoxyglabridin | AR |
| 3'-Mehoxyglabridin | SCN5A |
| 3'-Mehoxyglabridin | PPARG |
| 3'-Mehoxyglabridin | F10 |
| 3'-Mehoxyglabridin | PTGS2 |
| 3'-Mehoxyglabridin | CA2 |
| 3'-Mehoxyglabridin | F7 |
| 3'-Mehoxyglabridin | RXRA |
| 3'-Mehoxyglabridin | ACHE |
| 3'-Mehoxyglabridin | ADRA1B |
| 3'-Mehoxyglabridin | PTPN1 |
| 3'-Mehoxyglabridin | ADRB2 |
| 3'-Mehoxyglabridin | ESR2 |
| 3'-Mehoxyglabridin | DPP4 |
| 3'-Mehoxyglabridin | MAPK14 |
| 3'-Mehoxyglabridin | GSK3B |
| 3'-Mehoxyglabridin | HSP90AB1 |
| 3'-Mehoxyglabridin | PRSS1 |
| 3'-Mehoxyglabridin | PIM1 |
| 3'-Mehoxyglabridin | CCNA2 |
| 3'-Mehoxyglabridin | NCOA2 |
| 3'-Mehoxyglabridin | NCOA1 |
| 3'-Mehoxyglabridin | KCNMA1 |
| 3'-Mehoxyglabridin | CALM1 |
| 2-[(3R)-8,8-dimehyl-3,4-dihydro-2H-pyrano[6,5-f]chromen-3-yl]-5-mehoxyphenol | NOS2 |
| 2-[(3R)-8,8-dimehyl-3,4-dihydro-2H-pyrano[6,5-f]chromen-3-yl]-5-mehoxyphenol | PTGS1 |
| 2-[(3R)-8,8-dimehyl-3,4-dihydro-2H-pyrano[6,5-f]chromen-3-yl]-5-mehoxyphenol | CHRM3 |
| 2-[(3R)-8,8-dimehyl-3,4-dihydro-2H-pyrano[6,5-f]chromen-3-yl]-5-mehoxyphenol | F2 |
| 2-[(3R)-8,8-dimehyl-3,4-dihydro-2H-pyrano[6,5-f]chromen-3-yl]-5-mehoxyphenol | KCNH2 |
| 2-[(3R)-8,8-dimehyl-3,4-dihydro-2H-pyrano[6,5-f]chromen-3-yl]-5-mehoxyphenol | CHRM1 |
| 2-[(3R)-8,8-dimehyl-3,4-dihydro-2H-pyrano[6,5-f]chromen-3-yl]-5-mehoxyphenol | ESR1 |
| 2-[(3R)-8,8-dimehyl-3,4-dihydro-2H-pyrano[6,5-f]chromen-3-yl]-5-mehoxyphenol | AR |
| 2-[(3R)-8,8-dimehyl-3,4-dihydro-2H-pyrano[6,5-f]chromen-3-yl]-5-mehoxyphenol | SCN5A |
| 2-[(3R)-8,8-dimehyl-3,4-dihydro-2H-pyrano[6,5-f]chromen-3-yl]-5-mehoxyphenol | PPARG |
| 2-[(3R)-8,8-dimehyl-3,4-dihydro-2H-pyrano[6,5-f]chromen-3-yl]-5-mehoxyphenol | F10 |
| 2-[(3R)-8,8-dimehyl-3,4-dihydro-2H-pyrano[6,5-f]chromen-3-yl]-5-mehoxyphenol | PTGS2 |
| 2-[(3R)-8,8-dimehyl-3,4-dihydro-2H-pyrano[6,5-f]chromen-3-yl]-5-mehoxyphenol | NOS3 |
| 2-[(3R)-8,8-dimehyl-3,4-dihydro-2H-pyrano[6,5-f]chromen-3-yl]-5-mehoxyphenol | CA2 |
| 2-[(3R)-8,8-dimehyl-3,4-dihydro-2H-pyrano[6,5-f]chromen-3-yl]-5-mehoxyphenol | RXRA |
| 2-[(3R)-8,8-dimehyl-3,4-dihydro-2H-pyrano[6,5-f]chromen-3-yl]-5-mehoxyphenol | ACHE |
| 2-[(3R)-8,8-dimehyl-3,4-dihydro-2H-pyrano[6,5-f]chromen-3-yl]-5-mehoxyphenol | ADRA1B |
| 2-[(3R)-8,8-dimehyl-3,4-dihydro-2H-pyrano[6,5-f]chromen-3-yl]-5-mehoxyphenol | PTPN1 |
| 2-[(3R)-8,8-dimehyl-3,4-dihydro-2H-pyrano[6,5-f]chromen-3-yl]-5-mehoxyphenol | ADRB2 |
| 2-[(3R)-8,8-dimehyl-3,4-dihydro-2H-pyrano[6,5-f]chromen-3-yl]-5-mehoxyphenol | ESR2 |
| 2-[(3R)-8,8-dimehyl-3,4-dihydro-2H-pyrano[6,5-f]chromen-3-yl]-5-mehoxyphenol | DPP4 |
| 2-[(3R)-8,8-dimehyl-3,4-dihydro-2H-pyrano[6,5-f]chromen-3-yl]-5-mehoxyphenol | MAPK14 |
| 2-[(3R)-8,8-dimehyl-3,4-dihydro-2H-pyrano[6,5-f]chromen-3-yl]-5-mehoxyphenol | GSK3B |
| 2-[(3R)-8,8-dimehyl-3,4-dihydro-2H-pyrano[6,5-f]chromen-3-yl]-5-mehoxyphenol | PRSS1 |
| 2-[(3R)-8,8-dimehyl-3,4-dihydro-2H-pyrano[6,5-f]chromen-3-yl]-5-mehoxyphenol | PIM1 |
| 2-[(3R)-8,8-dimehyl-3,4-dihydro-2H-pyrano[6,5-f]chromen-3-yl]-5-mehoxyphenol | CCNA2 |
| 2-[(3R)-8,8-dimehyl-3,4-dihydro-2H-pyrano[6,5-f]chromen-3-yl]-5-mehoxyphenol | NCOA2 |
| 2-[(3R)-8,8-dimehyl-3,4-dihydro-2H-pyrano[6,5-f]chromen-3-yl]-5-mehoxyphenol | NCOA1 |
| 2-[(3R)-8,8-dimehyl-3,4-dihydro-2H-pyrano[6,5-f]chromen-3-yl]-5-mehoxyphenol | KCNMA1 |
| 2-[(3R)-8,8-dimehyl-3,4-dihydro-2H-pyrano[6,5-f]chromen-3-yl]-5-mehoxyphenol | CALM1 |
| Inflacoumarin A | NOS2 |
| Inflacoumarin A | PTGS1 |
| Inflacoumarin A | F2 |
| Inflacoumarin A | ESR1 |
| Inflacoumarin A | AR |
| Inflacoumarin A | SCN5A |
| Inflacoumarin A | PPARG |
| Inflacoumarin A | F10 |
| Inflacoumarin A | PTGS2 |
| Inflacoumarin A | CA2 |
| Inflacoumarin A | PTPN1 |
| Inflacoumarin A | ADRB2 |
| Inflacoumarin A | ESR2 |
| Inflacoumarin A | DPP4 |
| Inflacoumarin A | MAPK14 |
| Inflacoumarin A | GSK3B |
| Inflacoumarin A | HSP90AB1 |
| Inflacoumarin A | PRSS1 |
| Inflacoumarin A | PIM1 |
| Inflacoumarin A | CCNA2 |
| Inflacoumarin A | NCOA2 |
| Inflacoumarin A | CALM1 |
| icos-5-enoic acid | F2 |
| icos-5-enoic acid | PPARG |
| icos-5-enoic acid | NOS3 |
| icos-5-enoic acid | ACHE |
| icos-5-enoic acid | NCOA2 |
| Kanzonol F | ESR1 |
| Kanzonol F | AR |
| Kanzonol F | F10 |
| Kanzonol F | PTGS2 |
| Kanzonol F | CA2 |
| Kanzonol F | ESR2 |
| Kanzonol F | PIM1 |
| Kanzonol F | NCOA2 |
| Kanzonol F | CALM1 |
| 6-prenylated eriodictyol | NOS2 |
| 6-prenylated eriodictyol | F2 |
| 6-prenylated eriodictyol | ESR1 |
| 6-prenylated eriodictyol | AR |
| 6-prenylated eriodictyol | SCN5A |
| 6-prenylated eriodictyol | PPARG |
| 6-prenylated eriodictyol | F10 |
| 6-prenylated eriodictyol | PTGS2 |
| 6-prenylated eriodictyol | CA2 |
| 6-prenylated eriodictyol | F7 |
| 6-prenylated eriodictyol | PTPN1 |
| 6-prenylated eriodictyol | ESR2 |
| 6-prenylated eriodictyol | DPP4 |
| 6-prenylated eriodictyol | MAPK14 |
| 6-prenylated eriodictyol | GSK3B |
| 6-prenylated eriodictyol | HSP90AB1 |
| 6-prenylated eriodictyol | PRSS1 |
| 6-prenylated eriodictyol | PIM1 |
| 6-prenylated eriodictyol | CCNA2 |
| 6-prenylated eriodictyol | CALM1 |
| 7,2',4'-trihydroxy－5-mehoxy-3－arylcoumarin | NOS2 |
| 7,2',4'-trihydroxy－5-mehoxy-3－arylcoumarin | PTGS1 |
| 7,2',4'-trihydroxy－5-mehoxy-3－arylcoumarin | ESR1 |
| 7,2',4'-trihydroxy－5-mehoxy-3－arylcoumarin | AR |
| 7,2',4'-trihydroxy－5-mehoxy-3－arylcoumarin | PPARG |
| 7,2',4'-trihydroxy－5-mehoxy-3－arylcoumarin | PTGS2 |
| 7,2',4'-trihydroxy－5-mehoxy-3－arylcoumarin | CA2 |
| 7,2',4'-trihydroxy－5-mehoxy-3－arylcoumarin | PTPN1 |
| 7,2',4'-trihydroxy－5-mehoxy-3－arylcoumarin | ESR2 |
| 7,2',4'-trihydroxy－5-mehoxy-3－arylcoumarin | DPP4 |
| 7,2',4'-trihydroxy－5-mehoxy-3－arylcoumarin | MAPK14 |
| 7,2',4'-trihydroxy－5-mehoxy-3－arylcoumarin | GSK3B |
| 7,2',4'-trihydroxy－5-mehoxy-3－arylcoumarin | HSP90AB1 |
| 7,2',4'-trihydroxy－5-mehoxy-3－arylcoumarin | PIM1 |
| 7,2',4'-trihydroxy－5-mehoxy-3－arylcoumarin | CCNA2 |
| 7-Acetoxy-2-mehylisoflavone | NOS2 |
| 7-Acetoxy-2-mehylisoflavone | PTGS1 |
| 7-Acetoxy-2-mehylisoflavone | F2 |
| 7-Acetoxy-2-mehylisoflavone | ESR1 |
| 7-Acetoxy-2-mehylisoflavone | AR |
| 7-Acetoxy-2-mehylisoflavone | SCN5A |
| 7-Acetoxy-2-mehylisoflavone | PPARG |
| 7-Acetoxy-2-mehylisoflavone | PTGS2 |
| 7-Acetoxy-2-mehylisoflavone | NOS3 |
| 7-Acetoxy-2-mehylisoflavone | CA2 |
| 7-Acetoxy-2-mehylisoflavone | RXRA |
| 7-Acetoxy-2-mehylisoflavone | ACHE |
| 7-Acetoxy-2-mehylisoflavone | PDE3A |
| 7-Acetoxy-2-mehylisoflavone | ADRA1B |
| 7-Acetoxy-2-mehylisoflavone | PTPN1 |
| 7-Acetoxy-2-mehylisoflavone | ADRB2 |
| 7-Acetoxy-2-mehylisoflavone | ADRA1D |
| 7-Acetoxy-2-mehylisoflavone | ESR2 |
| 7-Acetoxy-2-mehylisoflavone | DPP4 |
| 7-Acetoxy-2-mehylisoflavone | MAPK14 |
| 7-Acetoxy-2-mehylisoflavone | GSK3B |
| 7-Acetoxy-2-mehylisoflavone | HSP90AB1 |
| 7-Acetoxy-2-mehylisoflavone | PRSS1 |
| 7-Acetoxy-2-mehylisoflavone | PIM1 |
| 7-Acetoxy-2-mehylisoflavone | CCNA2 |
| 7-Acetoxy-2-mehylisoflavone | NCOA2 |
| 7-Acetoxy-2-mehylisoflavone | CALM1 |
| 8-prenylated eriodictyol | NOS2 |
| 8-prenylated eriodictyol | F2 |
| 8-prenylated eriodictyol | ESR1 |
| 8-prenylated eriodictyol | AR |
| 8-prenylated eriodictyol | SCN5A |
| 8-prenylated eriodictyol | PPARG |
| 8-prenylated eriodictyol | F10 |
| 8-prenylated eriodictyol | PTGS2 |
| 8-prenylated eriodictyol | F7 |
| 8-prenylated eriodictyol | PTPN1 |
| 8-prenylated eriodictyol | ESR2 |
| 8-prenylated eriodictyol | DPP4 |
| 8-prenylated eriodictyol | MAPK14 |
| 8-prenylated eriodictyol | GSK3B |
| 8-prenylated eriodictyol | HSP90AB1 |
| 8-prenylated eriodictyol | PRSS1 |
| 8-prenylated eriodictyol | PIM1 |
| 8-prenylated eriodictyol | NCOA1 |
| 8-prenylated eriodictyol | CALM1 |
| gadelaidic acid | F2 |
| gadelaidic acid | PPARG |
| gadelaidic acid | NOS3 |
| gadelaidic acid | ACHE |
| gadelaidic acid | NCOA2 |
| Gancaonin G | NOS2 |
| Gancaonin G | F2 |
| Gancaonin G | ESR1 |
| Gancaonin G | AR |
| Gancaonin G | PPARG |
| Gancaonin G | F10 |
| Gancaonin G | PTGS2 |
| Gancaonin G | NOS3 |
| Gancaonin G | CA2 |
| Gancaonin G | PTPN1 |
| Gancaonin G | ESR2 |
| Gancaonin G | DPP4 |
| Gancaonin G | MAPK14 |
| Gancaonin G | GSK3B |
| Gancaonin G | HSP90AB1 |
| Gancaonin G | PRSS1 |
| Gancaonin G | PIM1 |
| Gancaonin G | CCNA2 |
| Gancaonin G | NCOA2 |
| Gancaonin G | CALM1 |
| Gancaonin H | ESR1 |
| Gancaonin H | AR |
| Gancaonin H | F10 |
| Gancaonin H | PTGS2 |
| Gancaonin H | CA2 |
| Gancaonin H | KDR |
| Gancaonin H | PTPN1 |
| Gancaonin H | HSP90AB1 |
| Gancaonin H | PRSS1 |
| Gancaonin H | PIM1 |
| Gancaonin H | CCNA2 |
| Gancaonin H | NCOA2 |
| Gancaonin H | CALM1 |
| Licoagrocarpin | NOS2 |
| Licoagrocarpin | PTGS1 |
| Licoagrocarpin | CHRM3 |
| Licoagrocarpin | F2 |
| Licoagrocarpin | KCNH2 |
| Licoagrocarpin | CHRM1 |
| Licoagrocarpin | ESR1 |
| Licoagrocarpin | AR |
| Licoagrocarpin | SCN5A |
| Licoagrocarpin | PPARG |
| Licoagrocarpin | F10 |
| Licoagrocarpin | PTGS2 |
| Licoagrocarpin | NOS3 |
| Licoagrocarpin | CA2 |
| Licoagrocarpin | RXRA |
| Licoagrocarpin | ACHE |
| Licoagrocarpin | ADRA1B |
| Licoagrocarpin | ADRB2 |
| Licoagrocarpin | ESR2 |
| Licoagrocarpin | DPP4 |
| Licoagrocarpin | MAPK14 |
| Licoagrocarpin | GSK3B |
| Licoagrocarpin | HSP90AB1 |
| Licoagrocarpin | PRSS1 |
| Licoagrocarpin | PIM1 |
| Licoagrocarpin | CCNA2 |
| Licoagrocarpin | NCOA2 |
| Licoagrocarpin | CALM1 |
| Glyasperins M | NOS2 |
| Glyasperins M | PTGS1 |
| Glyasperins M | F2 |
| Glyasperins M | KCNH2 |
| Glyasperins M | ESR1 |
| Glyasperins M | AR |
| Glyasperins M | SCN5A |
| Glyasperins M | PPARG |
| Glyasperins M | F10 |
| Glyasperins M | PTGS2 |
| Glyasperins M | CA2 |
| Glyasperins M | F7 |
| Glyasperins M | KDR |
| Glyasperins M | ACHE |
| Glyasperins M | PTPN1 |
| Glyasperins M | ESR2 |
| Glyasperins M | DPP4 |
| Glyasperins M | GSK3B |
| Glyasperins M | HSP90AB1 |
| Glyasperins M | PRSS1 |
| Glyasperins M | PIM1 |
| Glyasperins M | CCNA2 |
| Glyasperins M | NCOA2 |
| Glyasperins M | NCOA1 |
| Glyasperins M | KCNMA1 |
| Glyasperins M | CALM1 |
| Glycyrrhiza flavonol A | NOS2 |
| Glycyrrhiza flavonol A | ESR1 |
| Glycyrrhiza flavonol A | AR |
| Glycyrrhiza flavonol A | F10 |
| Glycyrrhiza flavonol A | PTGS2 |
| Glycyrrhiza flavonol A | CA2 |
| Glycyrrhiza flavonol A | F7 |
| Glycyrrhiza flavonol A | ACHE |
| Glycyrrhiza flavonol A | PTPN1 |
| Glycyrrhiza flavonol A | ESR2 |
| Glycyrrhiza flavonol A | DPP4 |
| Glycyrrhiza flavonol A | GSK3B |
| Glycyrrhiza flavonol A | HSP90AB1 |
| Glycyrrhiza flavonol A | PRSS1 |
| Glycyrrhiza flavonol A | PIM1 |
| Glycyrrhiza flavonol A | CCNA2 |
| Glycyrrhiza flavonol A | CALM1 |
| Licoagroisoflavone | NOS2 |
| Licoagroisoflavone | F2 |
| Licoagroisoflavone | ESR1 |
| Licoagroisoflavone | AR |
| Licoagroisoflavone | SCN5A |
| Licoagroisoflavone | PPARG |
| Licoagroisoflavone | F10 |
| Licoagroisoflavone | PTGS2 |
| Licoagroisoflavone | CA2 |
| Licoagroisoflavone | PTPN1 |
| Licoagroisoflavone | ESR2 |
| Licoagroisoflavone | DPP4 |
| Licoagroisoflavone | MAPK14 |
| Licoagroisoflavone | GSK3B |
| Licoagroisoflavone | PRSS1 |
| Licoagroisoflavone | PIM1 |
| Licoagroisoflavone | CCNA2 |
| Licoagroisoflavone | CALM1 |
| 18α-hydroxyglycyrrhetic acid | AR |
| 18α-hydroxyglycyrrhetic acid | NR3C1 |
| Odoratin | NOS2 |
| Odoratin | PTGS1 |
| Odoratin | ESR1 |
| Odoratin | AR |
| Odoratin | SCN5A |
| Odoratin | PPARG |
| Odoratin | PTGS2 |
| Odoratin | CA2 |
| Odoratin | RXRA |
| Odoratin | PTPN1 |
| Odoratin | ESR2 |
| Odoratin | DPP4 |
| Odoratin | MAPK14 |
| Odoratin | GSK3B |
| Odoratin | HSP90AB1 |
| Odoratin | PRSS1 |
| Odoratin | PIM1 |
| Odoratin | CCNA2 |
| Odoratin | NCOA2 |
| Odoratin | CALM1 |
| Phaseol | NOS2 |
| Phaseol | F2 |
| Phaseol | ESR1 |
| Phaseol | AR |
| Phaseol | PPARG |
| Phaseol | PTGS2 |
| Phaseol | KDR |
| Phaseol | PTPN1 |
| Phaseol | ESR2 |
| Phaseol | MAPK14 |
| Phaseol | GSK3B |
| Phaseol | HSP90AB1 |
| Phaseol | PIM1 |
| Phaseol | CCNA2 |
| Xambioona | NOS2 |
| Xambioona | F2 |
| Xambioona | ESR1 |
| Xambioona | AR |
| Xambioona | F10 |
| Xambioona | PTGS2 |
| Xambioona | CA2 |
| Xambioona | ESR2 |
| Xambioona | GSK3B |
| Xambioona | PIM1 |
| Xambioona | NCOA2 |
| Xambioona | CALM1 |
| dehydroglyasperins C | NOS2 |
| dehydroglyasperins C | F2 |
| dehydroglyasperins C | ESR1 |
| dehydroglyasperins C | AR |
| dehydroglyasperins C | SCN5A |
| dehydroglyasperins C | PPARG |
| dehydroglyasperins C | F10 |
| dehydroglyasperins C | PTGS2 |
| dehydroglyasperins C | CA2 |
| dehydroglyasperins C | PTPN1 |
| dehydroglyasperins C | ADRB2 |
| dehydroglyasperins C | ESR2 |
| dehydroglyasperins C | DPP4 |
| dehydroglyasperins C | MAPK14 |
| dehydroglyasperins C | HSP90AB1 |
| dehydroglyasperins C | PRSS1 |
| dehydroglyasperins C | PIM1 |
| dehydroglyasperins C | CCNA2 |
| dehydroglyasperins C | NCOA2 |
| dehydroglyasperins C | CALM1 |
| acacetin | NOS2 |
| acacetin | BCL2 |
| acacetin | PTGS2 |
| acacetin | TP53 |
| acacetin | PTGS1 |
| acacetin | ESR1 |
| acacetin | AR |
| acacetin | PPARG |
| acacetin | CA2 |
| acacetin | PDE3A |
| acacetin | PTPN1 |
| acacetin | ADRB2 |
| acacetin | ESR2 |
| acacetin | DPP4 |
| acacetin | MAPK14 |
| acacetin | GSK3B |
| acacetin | HSP90AB1 |
| acacetin | Cell division protein kinase 2 |
| acacetin | PIK3CG |
| acacetin | Beta-lactamase |
| acacetin | CHEK1 |
| acacetin | mRNA of PKA Catalytic Subunit C-alpha |
| acacetin | PRSS1 |
| acacetin | PIM1 |
| acacetin | CCNA2 |
| acacetin | NCOA2 |
| acacetin | NCOA1 |
| acacetin | CALM1 |
| cis-Dihydroquercetin | NOS2 |
| cis-Dihydroquercetin | PTGS1 |
| cis-Dihydroquercetin | ESR1 |
| cis-Dihydroquercetin | AR |
| cis-Dihydroquercetin | PPARG |
| cis-Dihydroquercetin | PTGS2 |
| cis-Dihydroquercetin | CA2 |
| cis-Dihydroquercetin | RXRA |
| cis-Dihydroquercetin | PTPN1 |
| cis-Dihydroquercetin | ESR2 |
| cis-Dihydroquercetin | DPP4 |
| cis-Dihydroquercetin | MAPK14 |
| cis-Dihydroquercetin | GSK3B |
| cis-Dihydroquercetin | HSP90AB1 |
| cis-Dihydroquercetin | PIK3CG |
| cis-Dihydroquercetin | PRSS1 |
| cis-Dihydroquercetin | PIM1 |
| cis-Dihydroquercetin | CCNA2 |
| Hesperetin | NOS2 |
| Hesperetin | PTGS1 |
| Hesperetin | ESR1 |
| Hesperetin | AR |
| Hesperetin | SCN5A |
| Hesperetin | PPARG |
| Hesperetin | PTGS2 |
| Hesperetin | CA2 |
| Hesperetin | PTPN1 |
| Hesperetin | ESR2 |
| Hesperetin | DPP4 |
| Hesperetin | MAPK14 |
| Hesperetin | GSK3B |
| Hesperetin | HSP90AB1 |
| Hesperetin | PIK3CG |
| Hesperetin | PRSS1 |
| Hesperetin | PIM1 |
| Hesperetin | CCNA2 |
| Hesperetin | NCOA2 |
| Hesperetin | NCOA1 |
| Hesperetin | CALM1 |
| nobiletin | BCL2 |
| nobiletin | PTGS2 |
| nobiletin | JUN |
| nobiletin | TP53 |
| nobiletin | MAPK8 |
| nobiletin | NOS2 |
| nobiletin | PTGS1 |
| nobiletin | F2 |
| nobiletin | KCNH2 |
| nobiletin | ESR1 |
| nobiletin | AR |
| nobiletin | SCN5A |
| nobiletin | PPARG |
| nobiletin | F10 |
| nobiletin | CA2 |
| nobiletin | F7 |
| nobiletin | PTPN1 |
| nobiletin | ESR2 |
| nobiletin | DPP4 |
| nobiletin | GSK3B |
| nobiletin | HSP90AB1 |
| nobiletin | PRSS1 |
| nobiletin | PIM1 |
| nobiletin | CCNA2 |
| nobiletin | NCOA2 |
| nobiletin | KCNMA1 |
| nobiletin | CALM1 |
| Marmin | NOS2 |
| Marmin | F2 |
| Marmin | ESR1 |
| Marmin | AR |
| Marmin | PPARG |
| Marmin | PTGS2 |
| Marmin | NOS3 |
| Marmin | CA2 |
| Marmin | PDE3A |
| Marmin | ADRB2 |
| Marmin | DPP4 |
| Marmin | GSK3B |
| Marmin | PRSS1 |
| Fatty acid binding protein adipocyte | FABP4 |
| Fatty acid binding protein muscle | FABP3 |
| Peroxisome proliferator-activated receptor alpha | PPARA |
| Fatty acid binding protein adipocyte | FABP4 |
| Interleukin-2 | IL2 |
| Matrix mealloproteinase 2 | MMP2 |
| Matrix mealloproteinase 3 | MMP12 |
| Fatty acid binding protein adipocyte | FABP4 |
| Protein kinase C alpha | PRKCA |
| Interleukin-2 | IL2 |
| Aldose reductase | AKR1B1 |
| C44bonic anhydrase II | CA2 |
| Protein kinase C alpha | PRKCA |
| Beta-glucocerebrosidase | GBA |
| Peroxisome proliferator-activated receptor gamma | PPARG |
| Matrix Mealloproteinase 2 | MMP2 |
| Interleukin-2 | IL2 |
| Matrix mealloproteinase 2 | MMP2 |

**supplementary material (4)**

We retrieved 128 papers. Among these literatures, 15 literatures were not grouped according to the principle of random allocation. 63 literatures lacked the content required for mate analysis. 4 literatures lacked complete efficacy evaluation. 9 literatures violate inclusion criteria (3). 16 literatures violate inclusion criteria (5). Last, 21 literatures were included for further study.

**supplementary material (5)**

| **Gene Symbol** | **Description** | **Gifts** |
| --- | --- | --- |
| IL6 | Interleukin 6 | 50 |
| ACE | Angiotensin I Converting Enzyme | 49 |
| APOE | Apolipoprotein E | 50 |
| TNF | Tumor Necrosis Factor | 51 |
| ALB | Albumin | 50 |
| SCN5A | Sodium Voltage-Gated Channel Alpha Subunit 5 | 50 |
| APOB | Apolipoprotein B | 45 |
| NOS3 | Nitric Oxide Synthase 3 | 51 |
| LMNA | Lamin A/C | 47 |
| IL10 | Interleukin 10 | 47 |
| IL1B | Interleukin 1 Beta | 48 |
| APOA1 | Apolipoprotein A1 | 48 |
| ABCA1 | ATP Binding Cassette Subfamily A Member 1 | 48 |
| INS | Insulin | 48 |
| GATA4 | GATA Binding Protein 4 | 48 |
| VWF | Von Willebrand Factor | 48 |
| PRKAG2 | Protein Kinase AMP-Activated Non-Catalytic Subunit Gamma 2 | 50 |
| VEGFA | Vascular Endothelial Growth Factor A | 48 |
| EDN1 | Endothelin 1 | 47 |
| ELN | Elastin | 44 |
| NKX2-5 | NK2 Homeobox 5 | 44 |
| TGFB1 | Transforming Growth Factor Beta 1 | 52 |
| LDLR | Low Density Lipoprotein Receptor | 49 |
| CD40LG | CD40 Ligand | 47 |
| F2 | Coagulation Factor II, Thrombin | 48 |
| LPL | Lipoprotein Lipase | 49 |
| GJA1 | Gap Junction Protein Alpha 1 | 50 |
| PON1 | Paraoxonase 1 | 45 |
| MYH6 | Myosin Heavy Chain 6 | 45 |
| MTHFR | Methylenetetrahydrofolate Reductase | 47 |
| TNNI3 | Troponin I3, Cardiac Type | 48 |
| PPARG | Peroxisome Proliferator Activated Receptor Gamma | 52 |
| NPPA | Natriuretic Peptide A | 46 |
| TNNT2 | Troponin T2, Cardiac Type | 48 |
| SERPINE1 | Serpin Family E Member 1 | 50 |
| CCL2 | C-C Motif Chemokine Ligand 2 | 48 |
| TLR4 | Toll Like Receptor 4 | 51 |
| TP53 | Tumor Protein P53 | 54 |
| MYH7 | Myosin Heavy Chain 7 | 47 |
| AGTR1 | Angiotensin II Receptor Type 1 | 51 |
| NOTCH1 | Notch Receptor 1 | 51 |
| LPA | Lipoprotein(A) | 41 |
| CRP | C-Reactive Protein | 46 |
| TBX5 | T-Box Transcription Factor 5 | 45 |
| TTN | Titin | 47 |
| IFNG | Interferon Gamma | 48 |
| REN | Renin | 48 |
| ICAM1 | Intercellular Adhesion Molecule 1 | 50 |
| MMP3 | Matrix Metallopeptidase 3 | 51 |
| FBN1 | Fibrillin 1 | 45 |
| GATA6 | GATA Binding Protein 6 | 47 |
| RYR2 | Ryanodine Receptor 2 | 47 |
| CETP | Cholesteryl Ester Transfer Protein | 46 |
| KCNQ1 | Potassium Voltage-Gated Channel Subfamily Q Member 1 | 49 |
| JAG1 | Jagged Canonical Notch Ligand 1 | 50 |
| IGF1 | Insulin Like Growth Factor 1 | 50 |
| CDKN2A | Cyclin Dependent Kinase Inhibitor 2A | 51 |
| KCNJ5 | Potassium Inwardly Rectifying Channel Subfamily J Member 5 | 47 |
| FN1 | Fibronectin 1 | 50 |
| KCNH2 | Potassium Voltage-Gated Channel Subfamily H Member 2 | 49 |
| RYR1 | Ryanodine Receptor 1 | 47 |
| LEP | Leptin | 47 |
| PSEN1 | Presenilin 1 | 52 |
| AGT | Angiotensinogen | 49 |
| MPO | Myeloperoxidase | 50 |
| IL1RN | Interleukin 1 Receptor Antagonist | 48 |
| LIPC | Lipase C, Hepatic Type | 44 |
| NPPB | Natriuretic Peptide B | 44 |
| APOC3 | Apolipoprotein C3 | 43 |
| TTR | Transthyretin | 49 |
| SERPINC1 | Serpin Family C Member 1 | 48 |
| CAT | Catalase | 50 |
| ACTA2 | Actin Alpha 2, Smooth Muscle | 48 |
| MYBPC3 | Myosin Binding Protein C3 | 46 |
| KNG1 | Kininogen 1 | 44 |
| MMP2 | Matrix Metallopeptidase 2 | 53 |
| CACNA1C | Calcium Voltage-Gated Channel Subunit Alpha1 C | 48 |
| ADRB1 | Adrenoceptor Beta 1 | 48 |
| COG2 | Component Of Oligomeric Golgi Complex 2 | 40 |
| PCSK9 | Proprotein Convertase Subtilisin/Kexin Type 9 | 51 |
| TBX1 | T-Box Transcription Factor 1 | 42 |
| GJA5 | Gap Junction Protein Alpha 5 | 45 |
| MIR21 | MicroRNA 21 | 24 |
| SOD1 | Superoxide Dismutase 1 | 51 |
| EPO | Erythropoietin | 41 |
| HMOX1 | Heme Oxygenase 1 | 52 |
| PTPN11 | Protein Tyrosine Phosphatase Non-Receptor Type 11 | 53 |
| AKT1 | AKT Serine/Threonine Kinase 1 | 54 |
| TLR2 | Toll Like Receptor 2 | 51 |
| HFE | Homeostatic Iron Regulator | 43 |
| STAT3 | Signal Transducer And Activator Of Transcription 3 | 52 |
| DSP | Desmoplakin | 49 |
| TNFRSF1A | TNF Receptor Superfamily Member 1A | 49 |
| DMD | Dystrophin | 46 |
| ESR1 | Estrogen Receptor 1 | 53 |
| GLA | Galactosidase Alpha | 48 |
| HLA-DRB1 | Major Histocompatibility Complex, Class II, DR Beta 1 | 46 |
| THBD | Thrombomodulin | 44 |
| MIR126 | MicroRNA 126 | 22 |
| TGFB2 | Transforming Growth Factor Beta 2 | 50 |
| ADRB2 | Adrenoceptor Beta 2 | 48 |
| APOA5 | Apolipoprotein A5 | 44 |
| CTNNB1 | Catenin Beta 1 | 53 |
| TGFB3 | Transforming Growth Factor Beta 3 | 47 |
| LCAT | Lecithin-Cholesterol Acyltransferase | 47 |
| MMP1 | Matrix Metallopeptidase 1 | 51 |
| CD36 | CD36 Molecule | 48 |
| CST3 | Cystatin C | 44 |
| HRAS | HRas Proto-Oncogene, GTPase | 52 |
| COMT | Catechol-O-Methyltransferase | 51 |
| SMAD4 | SMAD Family Member 4 | 50 |
| ENG | Endoglin | 46 |
| CD40 | CD40 Molecule | 48 |
| MIR146A | MicroRNA 146a | 22 |
| SPP1 | Secreted Phosphoprotein 1 | 46 |
| ENPP1 | Ectonucleotide Pyrophosphatase/Phosphodiesterase 1 | 47 |
| APP | Amyloid Beta Precursor Protein | 51 |
| GAA | Glucosidase Alpha, Acid | 47 |
| PON2 | Paraoxonase 2 | 42 |
| NOS2 | Nitric Oxide Synthase 2 | 49 |
| TNFRSF11B | TNF Receptor Superfamily Member 11b | 47 |
| PLA2G7 | Phospholipase A2 Group VII | 50 |
| CTLA4 | Cytotoxic T-Lymphocyte Associated Protein 4 | 45 |
| MYH11 | Myosin Heavy Chain 11 | 45 |
| RETN | Resistin | 43 |
| MIR17 | MicroRNA 17 | 21 |
| HSPD1 | Heat Shock Protein Family D (Hsp60) Member 1 | 47 |
| CAV1 | Caveolin 1 | 48 |
| TNFRSF1B | TNF Receptor Superfamily Member 1B | 47 |
| MMP9 | Matrix Metallopeptidase 9 | 52 |
| TBX20 | T-Box Transcription Factor 20 | 41 |
| CXCR4 | C-X-C Motif Chemokine Receptor 4 | 52 |
| ZIC3 | Zic Family Member 3 | 46 |
| CXCL12 | C-X-C Motif Chemokine Ligand 12 | 45 |
| SMAD3 | SMAD Family Member 3 | 49 |
| FAS | Fas Cell Surface Death Receptor | 50 |
| MIR145 | MicroRNA 145 | 21 |
| TGFBR2 | Transforming Growth Factor Beta Receptor 2 | 51 |
| MIR155 | MicroRNA 155 | 18 |
| ACTC1 | Actin Alpha Cardiac Muscle 1 | 42 |
| KCNJ2 | Potassium Inwardly Rectifying Channel Subfamily J Member 2 | 48 |
| CFTR | CF Transmembrane Conductance Regulator | 51 |
| PLG | Plasminogen | 48 |
| CXCL8 | C-X-C Motif Chemokine Ligand 8 | 41 |
| TGFBR1 | Transforming Growth Factor Beta Receptor 1 | 52 |
| ANK2 | Ankyrin 2 | 41 |
| DES | Desmin | 48 |
| SOD2 | Superoxide Dismutase 2 | 51 |
| MAPK1 | Mitogen-Activated Protein Kinase 1 | 51 |
| HCN4 | Hyperpolarization Activated Cyclic Nucleotide Gated Potassium Channel 4 | 47 |
| CDKN2B | Cyclin Dependent Kinase Inhibitor 2B | 47 |
| TRPM4 | Transient Receptor Potential Cation Channel Subfamily M Member 4 | 45 |
| FLNA | Filamin A | 49 |
| MEF2A | Myocyte Enhancer Factor 2A | 47 |
| LOX | Lysyl Oxidase | 44 |
| PRODH | Proline Dehydrogenase 1 | 45 |
| KCNE2 | Potassium Voltage-Gated Channel Subfamily E Regulatory Subunit 2 | 41 |
| CCR5 | C-C Motif Chemokine Receptor 5 | 46 |
| F7 | Coagulation Factor VII | 47 |
| ADIPOQ | Adiponectin, C1Q And Collagen Domain Containing | 45 |
| F8 | Coagulation Factor VIII | 45 |
| MGP | Matrix Gla Protein | 42 |
| HTR2A | 5-Hydroxytryptamine Receptor 2A | 47 |
| PKP2 | Plakophilin 2 | 45 |
| EDNRA | Endothelin Receptor Type A | 49 |
| F5 | Coagulation Factor V | 45 |
| NPC1 | NPC Intracellular Cholesterol Transporter 1 | 47 |
| H19 | H19 Imprinted Maternally Expressed Transcript | 28 |
| CYBA | Cytochrome B-245 Alpha Chain | 47 |
| ELANE | Elastase, Neutrophil Expressed | 46 |
| ABCG5 | ATP Binding Cassette Subfamily G Member 5 | 44 |
| NR3C2 | Nuclear Receptor Subfamily 3 Group C Member 2 | 48 |
| BMPR2 | Bone Morphogenetic Protein Receptor Type 2 | 50 |
| LDB3 | LIM Domain Binding 3 | 41 |
| JUP | Junction Plakoglobin | 47 |
| SMPD1 | Sphingomyelin Phosphodiesterase 1 | 47 |
| PRKG1 | Protein Kinase CGMP-Dependent 1 | 51 |
| TMEM43 | Transmembrane Protein 43 | 40 |
| ITGB3 | Integrin Subunit Beta 3 | 49 |
| EDNRB | Endothelin Receptor Type B | 49 |
| EGFR | Epidermal Growth Factor Receptor | 54 |
| SLC6A4 | Solute Carrier Family 6 Member 4 | 47 |
| ABCG8 | ATP Binding Cassette Subfamily G Member 8 | 42 |
| SOS1 | SOS Ras/Rac Guanine Nucleotide Exchange Factor 1 | 47 |
| MIR223 | MicroRNA 223 | 21 |
| LAMP2 | Lysosomal Associated Membrane Protein 2 | 44 |
| POMC | Proopiomelanocortin | 48 |
| DSG2 | Desmoglein 2 | 45 |
| SELP | Selectin P | 45 |
| HLA-DQB1 | Major Histocompatibility Complex, Class II, DQ Beta 1 | 44 |
| ABCC9 | ATP Binding Cassette Subfamily C Member 9 | 44 |
| KCNE1 | Potassium Voltage-Gated Channel Subfamily E Regulatory Subunit 1 | 44 |
| GHRL | Ghrelin And Obestatin Prepropeptide | 43 |
| GATA5 | GATA Binding Protein 5 | 39 |
| APOA2 | Apolipoprotein A2 | 44 |
| COL3A1 | Collagen Type III Alpha 1 Chain | 47 |
| CFH | Complement Factor H | 45 |
| FOXP3 | Forkhead Box P3 | 46 |
| CRELD1 | Cysteine Rich With EGF Like Domains 1 | 39 |
| BMP4 | Bone Morphogenetic Protein 4 | 49 |
| HAND2 | Heart And Neural Crest Derivatives Expressed 2 | 43 |
| MTR | 5-Methyltetrahydrofolate-Homocysteine Methyltransferase | 46 |
| DSC2 | Desmocollin 2 | 45 |
| TAZ | Tafazzin | 44 |
| NAGLU | N-Acetyl-Alpha-Glucosaminidase | 43 |
| CYP11B2 | Cytochrome P450 Family 11 Subfamily B Member 2 | 47 |
| IL18 | Interleukin 18 | 44 |
| LRP6 | LDL Receptor Related Protein 6 | 48 |
| EGF | Epidermal Growth Factor | 51 |
| PSEN2 | Presenilin 2 | 49 |
| FOS | Fos Proto-Oncogene, AP-1 Transcription Factor Subunit | 50 |
| EMD | Emerin | 45 |
| SLC17A5 | Solute Carrier Family 17 Member 5 | 44 |
| TPM1 | Tropomyosin 1 | 48 |
| GSR | Glutathione-Disulfide Reductase | 48 |
| MT-CO1 | Mitochondrially Encoded Cytochrome C Oxidase I | 32 |
| PITX2 | Paired Like Homeodomain 2 | 47 |
| MME | Membrane Metalloendopeptidase | 50 |
| CCN2 | Cellular Communication Network Factor 2 | 39 |
| OLR1 | Oxidized Low Density Lipoprotein Receptor 1 | 44 |
| FCGR2A | Fc Fragment Of IgG Receptor IIa | 45 |
| RAF1 | Raf-1 Proto-Oncogene, Serine/Threonine Kinase | 54 |
| C3 | Complement C3 | 47 |
| FGF23 | Fibroblast Growth Factor 23 | 45 |
| MBL2 | Mannose Binding Lectin 2 | 47 |
| IL13 | Interleukin 13 | 44 |
| PLAT | Plasminogen Activator, Tissue Type | 49 |
| PKD2 | Polycystin 2, Transient Receptor Potential Cation Channel | 46 |
| ATP2A2 | ATPase Sarcoplasmic/Endoplasmic Reticulum Ca2+ Transporting 2 | 51 |
| BSCL2 | BSCL2 Lipid Droplet Biogenesis Associated, Seipin | 42 |
| B2M | Beta-2-Microglobulin | 48 |
| CACNB2 | Calcium Voltage-Gated Channel Auxiliary Subunit Beta 2 | 47 |
| CSRP3 | Cysteine And Glycine Rich Protein 3 | 41 |
| SELE | Selectin E | 44 |
| TBX2 | T-Box Transcription Factor 2 | 45 |
| STAT1 | Signal Transducer And Activator Of Transcription 1 | 53 |
| CRYAB | Crystallin Alpha B | 45 |
| IRS1 | Insulin Receptor Substrate 1 | 47 |
| F3 | Coagulation Factor III, Tissue Factor | 45 |
| MYLK | Myosin Light Chain Kinase | 52 |
| XDH | Xanthine Dehydrogenase | 47 |
| AVP | Arginine Vasopressin | 45 |
| FLT1 | Fms Related Receptor Tyrosine Kinase 1 | 51 |
| ADAMTS13 | ADAM Metallopeptidase With Thrombospondin Type 1 Motif 13 | 45 |
| GP1BA | Glycoprotein Ib Platelet Subunit Alpha | 45 |
| GNAS | GNAS Complex Locus | 50 |
| TEK | TEK Receptor Tyrosine Kinase | 50 |
| MIR150 | MicroRNA 150 | 21 |
| JAK2 | Janus Kinase 2 | 54 |
| VCAM1 | Vascular Cell Adhesion Molecule 1 | 45 |
| LTA | Lymphotoxin Alpha | 42 |
| HADHA | Hydroxyacyl-CoA Dehydrogenase Trifunctional Multienzyme Complex Subunit Alpha | 45 |
| INSR | Insulin Receptor | 54 |
| CCL3 | C-C Motif Chemokine Ligand 3 | 39 |
| FXN | Frataxin | 46 |
| COL1A1 | Collagen Type I Alpha 1 Chain | 50 |
| CHD7 | Chromodomain Helicase DNA Binding Protein 7 | 45 |
| MIR221 | MicroRNA 221 | 20 |
| BBS2 | Bardet-Biedl Syndrome 2 | 41 |
| PTPRC | Protein Tyrosine Phosphatase Receptor Type C | 51 |
| PRDM16 | PR/SET Domain 16 | 45 |
| MEF2C | Myocyte Enhancer Factor 2C | 48 |
| ITGA2B | Integrin Subunit Alpha 2b | 50 |
| MYL2 | Myosin Light Chain 2 | 49 |
| ITGAM | Integrin Subunit Alpha M | 46 |
| NR2F2 | Nuclear Receptor Subfamily 2 Group F Member 2 | 48 |
| CFC1 | Cripto, FRL-1, Cryptic Family 1 | 37 |
| IDUA | Alpha-L-Iduronidase | 42 |
| MAP2K2 | Mitogen-Activated Protein Kinase Kinase 2 | 53 |
| ABCC6 | ATP Binding Cassette Subfamily C Member 6 | 45 |
| ECE1 | Endothelin Converting Enzyme 1 | 47 |
| HGF | Hepatocyte Growth Factor | 52 |
| TBX4 | T-Box Transcription Factor 4 | 41 |
| NODAL | Nodal Growth Differentiation Factor | 42 |
| CYP2C9 | Cytochrome P450 Family 2 Subfamily C Member 9 | 48 |
| FLNC | Filamin C | 44 |
| ACTB | Actin Beta | 49 |
| PTH | Parathyroid Hormone | 47 |
| MYD88 | MYD88 Innate Immune Signal Transduction Adaptor | 50 |
| HMGCR | 3-Hydroxy-3-Methylglutaryl-CoA Reductase | 45 |
| ABCA4 | ATP Binding Cassette Subfamily A Member 4 | 44 |
| TLR3 | Toll Like Receptor 3 | 52 |
| KCNJ8 | Potassium Inwardly Rectifying Channel Subfamily J Member 8 | 43 |
| IL4 | Interleukin 4 | 46 |
| CITED2 | Cbp/P300 Interacting Transactivator With Glu/Asp Rich Carboxy-Terminal Domain 2 | 44 |
| NR3C1 | Nuclear Receptor Subfamily 3 Group C Member 1 | 50 |
| MIR29B1 | MicroRNA 29b-1 | 21 |
| MYPN | Myopalladin | 41 |
| ERCC6 | ERCC Excision Repair 6, Chromatin Remodeling Factor | 45 |
| AGTR2 | Angiotensin II Receptor Type 2 | 43 |
| CDKN2B-AS1 | CDKN2B Antisense RNA 1 | 21 |
| MIR208A | MicroRNA 208a | 18 |
| CPT2 | Carnitine Palmitoyltransferase 2 | 48 |
| TBX18 | T-Box Transcription Factor 18 | 41 |
| VCL | Vinculin | 47 |
| SRC | SRC Proto-Oncogene, Non-Receptor Tyrosine Kinase | 51 |
| LRP1 | LDL Receptor Related Protein 1 | 47 |
| MIR210 | MicroRNA 210 | 21 |
| PLN | Phospholamban | 44 |
| GLB1 | Galactosidase Beta 1 | 48 |
| NRG1 | Neuregulin 1 | 46 |
| ANKRD1 | Ankyrin Repeat Domain 1 | 42 |
| MIR483 | MicroRNA 483 | 18 |
| ISL1 | ISL LIM Homeobox 1 | 45 |
| GPT | Glutamic--Pyruvic Transaminase | 41 |
| MT-CYB | Mitochondrially Encoded Cytochrome B | 31 |
| ABL1 | ABL Proto-Oncogene 1, Non-Receptor Tyrosine Kinase | 52 |
| PKD1 | Polycystin 1, Transient Receptor Potential Channel Interacting | 45 |
| CYP2D6 | Cytochrome P450 Family 2 Subfamily D Member 6 | 48 |
| MYL3 | Myosin Light Chain 3 | 44 |
| CAV3 | Caveolin 3 | 43 |
| F9 | Coagulation Factor IX | 46 |
| HLA-B | Major Histocompatibility Complex, Class I, B | 45 |
| FGA | Fibrinogen Alpha Chain | 47 |
| IL2 | Interleukin 2 | 45 |
| SDHA | Succinate Dehydrogenase Complex Flavoprotein Subunit A | 46 |
| CYBB | Cytochrome B-245 Beta Chain | 47 |
| CASP3 | Caspase 3 | 50 |
| GFAP | Glial Fibrillary Acidic Protein | 47 |
| TERT | Telomerase Reverse Transcriptase | 51 |
| GGT1 | Gamma-Glutamyltransferase 1 | 46 |
| ACTN2 | Actinin Alpha 2 | 47 |
| IL17A | Interleukin 17A | 42 |
| CCL11 | C-C Motif Chemokine Ligand 11 | 43 |
| LIPA | Lipase A, Lysosomal Acid Type | 47 |
| ALOX5 | Arachidonate 5-Lipoxygenase | 48 |
| CALR | Calreticulin | 51 |
| ERBB2 | Erb-B2 Receptor Tyrosine Kinase 2 | 54 |
| EVC2 | EvC Ciliary Complex Subunit 2 | 39 |
| AKAP9 | A-Kinase Anchoring Protein 9 | 43 |
| MYC | MYC Proto-Oncogene, BHLH Transcription Factor | 51 |
| SLC25A4 | Solute Carrier Family 25 Member 4 | 48 |
| MIR22 | MicroRNA 22 | 20 |
| CASQ2 | Calsequestrin 2 | 44 |
| CX3CR1 | C-X3-C Motif Chemokine Receptor 1 | 44 |
| CD4 | CD4 Molecule | 49 |
| CACNA1D | Calcium Voltage-Gated Channel Subunit Alpha1 D | 47 |
| CYP7A1 | Cytochrome P450 Family 7 Subfamily A Member 1 | 42 |
| BMP6 | Bone Morphogenetic Protein 6 | 43 |
| KRAS | KRAS Proto-Oncogene, GTPase | 51 |
| MIR499A | MicroRNA 499a | 21 |
| RBM20 | RNA Binding Motif Protein 20 | 35 |
| BAG3 | BAG Cochaperone 3 | 44 |
| MIR214 | MicroRNA 214 | 20 |
| LAMA2 | Laminin Subunit Alpha 2 | 43 |
| NOD2 | Nucleotide Binding Oligomerization Domain Containing 2 | 48 |
| FKRP | Fukutin Related Protein | 40 |
| MIR27A | MicroRNA 27a | 22 |
| MIR30A | MicroRNA 30a | 20 |
| EVC | EvC Ciliary Complex Subunit 1 | 38 |
| SLC22A5 | Solute Carrier Family 22 Member 5 | 46 |
| ADRB3 | Adrenoceptor Beta 3 | 45 |
| ACTA1 | Actin Alpha 1, Skeletal Muscle | 47 |
| MB | Myoglobin | 43 |
| PPARGC1A | PPARG Coactivator 1 Alpha | 46 |
| SERPINA3 | Serpin Family A Member 3 | 43 |
| P2RY12 | Purinergic Receptor P2Y12 | 48 |
| ABCB1 | ATP Binding Cassette Subfamily B Member 1 | 51 |
| MYOT | Myotilin | 41 |
| DTNA | Dystrobrevin Alpha | 42 |
| ITGA2 | Integrin Subunit Alpha 2 | 45 |
| HP | Haptoglobin | 44 |
| DCN | Decorin | 47 |
| FBN2 | Fibrillin 2 | 41 |
| BDNF | Brain Derived Neurotrophic Factor | 47 |
| SMAD6 | SMAD Family Member 6 | 46 |
| MYOCD | Myocardin | 41 |
| MIR195 | MicroRNA 195 | 19 |
| CD55 | CD55 Molecule (Cromer Blood Group) | 47 |
| KITLG | KIT Ligand | 44 |
| NEFL | Neurofilament Light | 46 |
| PIK3C2A | Phosphatidylinositol-4-Phosphate 3-Kinase Catalytic Subunit Type 2 Alpha | 47 |
| IGF2R | Insulin Like Growth Factor 2 Receptor | 45 |
| WT1 | WT1 Transcription Factor | 49 |
| SGCD | Sarcoglycan Delta | 45 |
| ALMS1 | ALMS1 Centrosome And Basal Body Associated Protein | 41 |
| NR1H4 | Nuclear Receptor Subfamily 1 Group H Member 4 | 48 |
| KCNJ11 | Potassium Inwardly Rectifying Channel Subfamily J Member 11 | 47 |
| KDR | Kinase Insert Domain Receptor | 53 |
| BMP2 | Bone Morphogenetic Protein 2 | 47 |
| F13A1 | Coagulation Factor XIII A Chain | 45 |
| TCAP | Titin-Cap | 42 |
| CASR | Calcium Sensing Receptor | 50 |
| TIMP1 | TIMP Metallopeptidase Inhibitor 1 | 45 |
| LDLRAP1 | Low Density Lipoprotein Receptor Adaptor Protein 1 | 43 |
| MIR34A | MicroRNA 34a | 22 |
| SCN4B | Sodium Voltage-Gated Channel Beta Subunit 4 | 43 |
| CASP8 | Caspase 8 | 52 |
| CACNA1S | Calcium Voltage-Gated Channel Subunit Alpha1 S | 48 |
| KCND3 | Potassium Voltage-Gated Channel Subfamily D Member 3 | 46 |
| PECAM1 | Platelet And Endothelial Cell Adhesion Molecule 1 | 40 |
| LEPR | Leptin Receptor | 49 |
| ADM | Adrenomedullin | 44 |
| CEP290 | Centrosomal Protein 290 | 40 |
| AHSG | Alpha 2-HS Glycoprotein | 44 |
| BRAF | B-Raf Proto-Oncogene, Serine/Threonine Kinase | 54 |
| G6PD | Glucose-6-Phosphate Dehydrogenase | 50 |
| F10 | Coagulation Factor X | 48 |
| SNTA1 | Syntrophin Alpha 1 | 43 |
| MIR486-1 | MicroRNA 486-1 | 16 |
| MAPT | Microtubule Associated Protein Tau | 50 |
| COL5A1 | Collagen Type V Alpha 1 Chain | 45 |
| MKKS | McKusick-Kaufman Syndrome | 39 |
| MED13L | Mediator Complex Subunit 13L | 39 |
| FGF2 | Fibroblast Growth Factor 2 | 47 |
| GNB3 | G Protein Subunit Beta 3 | 47 |
| ABCC8 | ATP Binding Cassette Subfamily C Member 8 | 45 |
| MIR222 | MicroRNA 222 | 21 |
| CACNA2D1 | Calcium Voltage-Gated Channel Auxiliary Subunit Alpha2delta 1 | 45 |
| MMP13 | Matrix Metallopeptidase 13 | 50 |
| TKT | Transketolase | 46 |
| KL | Klotho | 44 |
| GALNS | Galactosamine (N-Acetyl)-6-Sulfatase | 44 |
| NFE2L2 | Nuclear Factor, Erythroid 2 Like 2 | 48 |
| EYA4 | EYA Transcriptional Coactivator And Phosphatase 4 | 43 |
| SALL1 | Spalt Like Transcription Factor 1 | 44 |
| SYNE1 | Spectrin Repeat Containing Nuclear Envelope Protein 1 | 41 |
| PROM1 | Prominin 1 | 44 |
| ATM | ATM Serine/Threonine Kinase | 54 |
| ZMPSTE24 | Zinc Metallopeptidase STE24 | 43 |
| ACADVL | Acyl-CoA Dehydrogenase Very Long Chain | 45 |
| SCO2 | Synthesis Of Cytochrome C Oxidase 2 | 45 |
| AR | Androgen Receptor | 53 |
| PPARA | Peroxisome Proliferator Activated Receptor Alpha | 45 |
| TNNT1 | Troponin T1, Slow Skeletal Type | 43 |
| TNNC1 | Troponin C1, Slow Skeletal And Cardiac Type | 45 |
| DAG1 | Dystroglycan 1 | 46 |
| ADAM17 | ADAM Metallopeptidase Domain 17 | 51 |
| SERPINF2 | Serpin Family F Member 2 | 44 |
| HIF1A | Hypoxia Inducible Factor 1 Subunit Alpha | 47 |
| GDF1 | Growth Differentiation Factor 1 | 37 |
| MIR181A1 | MicroRNA 181a-1 | 18 |
| ACADM | Acyl-CoA Dehydrogenase Medium Chain | 47 |
| PIGL | Phosphatidylinositol Glycan Anchor Biosynthesis Class L | 41 |
| FKTN | Fukutin | 39 |
| AGPAT2 | 1-Acylglycerol-3-Phosphate O-Acyltransferase 2 | 45 |
| WRN | WRN RecQ Like Helicase | 45 |
| BMPR1A | Bone Morphogenetic Protein Receptor Type 1A | 51 |
| LAMA4 | Laminin Subunit Alpha 4 | 44 |
| ARSB | Arylsulfatase B | 44 |
| NEXN | Nexilin F-Actin Binding Protein | 39 |
| NPC2 | NPC Intracellular Cholesterol Transporter 2 | 41 |
| RBP4 | Retinol Binding Protein 4 | 44 |
| IL1A | Interleukin 1 Alpha | 44 |
| MIR142 | MicroRNA 142 | 20 |
| PAH | Phenylalanine Hydroxylase | 48 |
| TRDN | Triadin | 41 |
| FMR1 | FMRP Translational Regulator 1 | 44 |
| BBS1 | Bardet-Biedl Syndrome 1 | 38 |
| MTOR | Mechanistic Target Of Rapamycin Kinase | 54 |
| NF1 | Neurofibromin 1 | 48 |
| FCGR2B | Fc Fragment Of IgG Receptor IIb | 47 |
| MIR140 | MicroRNA 140 | 22 |
| PSAP | Prosaposin | 46 |
| SYNE2 | Spectrin Repeat Containing Nuclear Envelope Protein 2 | 39 |
| DYNC2H1 | Dynein Cytoplasmic 2 Heavy Chain 1 | 38 |
| CALM1 | Calmodulin 1 | 45 |
| PDCD1 | Programmed Cell Death 1 | 48 |
| DYNC2LI1 | Dynein Cytoplasmic 2 Light Intermediate Chain 1 | 39 |
| ARG1 | Arginase 1 | 50 |
| FABP3 | Fatty Acid Binding Protein 3 | 44 |
| SLC2A10 | Solute Carrier Family 2 Member 10 | 44 |
| SMAD9 | SMAD Family Member 9 | 45 |
| GP6 | Glycoprotein VI Platelet | 44 |
| HBB | Hemoglobin Subunit Beta | 45 |
| GHR | Growth Hormone Receptor | 47 |
| TFAP2B | Transcription Factor AP-2 Beta | 44 |
| NF2 | Neurofibromin 2 | 48 |
| H2AC18 | H2A Clustered Histone 18 | 26 |
| LBR | Lamin B Receptor | 47 |
| BCL2 | BCL2 Apoptosis Regulator | 51 |
| FBLN5 | Fibulin 5 | 44 |
| PTGS2 | Prostaglandin-Endoperoxide Synthase 2 | 48 |
| ADRA2B | Adrenoceptor Alpha 2B | 45 |
| CIITA | Class II Major Histocompatibility Complex Transactivator | 45 |
| F11 | Coagulation Factor XI | 45 |
| CBS | Cystathionine Beta-Synthase | 48 |
| PTEN | Phosphatase And Tensin Homolog | 52 |
| TPM2 | Tropomyosin 2 | 44 |
| PLEC | Plectin | 42 |
| MIR143 | MicroRNA 143 | 22 |
| BLK | BLK Proto-Oncogene, Src Family Tyrosine Kinase | 51 |
| ALPL | Alkaline Phosphatase, Biomineralization Associated | 50 |
| SAA1 | Serum Amyloid A1 | 42 |
| CELA2A | Chymotrypsin Like Elastase 2A | 39 |
| PF4 | Platelet Factor 4 | 41 |
| ACVRL1 | Activin A Receptor Like Type 1 | 50 |
| TH | Tyrosine Hydroxylase | 51 |
| MYL4 | Myosin Light Chain 4 | 44 |
| CORIN | Corin, Serine Peptidase | 44 |
| MYOZ2 | Myozenin 2 | 39 |
| ZFPM2 | Zinc Finger Protein, FOG Family Member 2 | 41 |
| NEU1 | Neuraminidase 1 | 44 |
| POLG | DNA Polymerase Gamma, Catalytic Subunit | 45 |
| AGL | Amylo-Alpha-1, 6-Glucosidase, 4-Alpha-Glucanotransferase | 45 |
| VCP | Valosin Containing Protein | 48 |
| PDE4D | Phosphodiesterase 4D | 48 |
| SGCA | Sarcoglycan Alpha | 41 |
| PRKAR1A | Protein Kinase CAMP-Dependent Type I Regulatory Subunit Alpha | 51 |
| LRP5 | LDL Receptor Related Protein 5 | 48 |
| TNFRSF11A | TNF Receptor Superfamily Member 11a | 45 |
| NPY | Neuropeptide Y | 45 |
| ADD1 | Adducin 1 | 44 |
| SGSH | N-Sulfoglucosamine Sulfohydrolase | 44 |
| C1S | Complement C1s | 45 |
| MLXIPL | MLX Interacting Protein Like | 41 |
| COX5A | Cytochrome C Oxidase Subunit 5A | 43 |
| GCK | Glucokinase | 50 |
| COL5A2 | Collagen Type V Alpha 2 Chain | 41 |
| TIMP3 | TIMP Metallopeptidase Inhibitor 3 | 45 |
| GBE1 | 1,4-Alpha-Glucan Branching Enzyme 1 | 44 |
| RPGRIP1L | RPGRIP1 Like | 39 |
| TYR | Tyrosinase | 47 |
| AVPR2 | Arginine Vasopressin Receptor 2 | 47 |
| TRPV4 | Transient Receptor Potential Cation Channel Subfamily V Member 4 | 49 |
| SLC25A20 | Solute Carrier Family 25 Member 20 | 46 |
| TNNI3K | TNNI3 Interacting Kinase | 40 |
| JPH2 | Junctophilin 2 | 40 |
| ABCA3 | ATP Binding Cassette Subfamily A Member 3 | 48 |
| MIR144 | MicroRNA 144 | 16 |
| VHL | Von Hippel-Lindau Tumor Suppressor | 47 |
| GDNF | Glial Cell Derived Neurotrophic Factor | 47 |
| PNPLA2 | Patatin Like Phospholipase Domain Containing 2 | 44 |
| PTGIS | Prostaglandin I2 Synthase | 47 |
| PTPN22 | Protein Tyrosine Phosphatase Non-Receptor Type 22 | 46 |
| SCN2B | Sodium Voltage-Gated Channel Beta Subunit 2 | 43 |
| VKORC1 | Vitamin K Epoxide Reductase Complex Subunit 1 | 47 |
| SORL1 | Sortilin Related Receptor 1 | 43 |
| MIR125A | MicroRNA 125a | 21 |
| SHH | Sonic Hedgehog Signaling Molecule | 50 |
| PINK1 | PTEN Induced Kinase 1 | 47 |
| RUNX2 | RUNX Family Transcription Factor 2 | 47 |
| FGB | Fibrinogen Beta Chain | 45 |
| MTTP | Microsomal Triglyceride Transfer Protein | 44 |
| PDE5A | Phosphodiesterase 5A | 44 |
| COL4A1 | Collagen Type IV Alpha 1 Chain | 47 |
| SPTA1 | Spectrin Alpha, Erythrocytic 1 | 43 |
| TPM3 | Tropomyosin 3 | 47 |
| IL2RA | Interleukin 2 Receptor Subunit Alpha | 50 |
| LCN2 | Lipocalin 2 | 43 |
| CDH2 | Cadherin 2 | 50 |
| NPR2 | Natriuretic Peptide Receptor 2 | 47 |
| MAP2K1 | Mitogen-Activated Protein Kinase Kinase 1 | 54 |
| MRAP | Melanocortin 2 Receptor Accessory Protein | 37 |
| MT-ND1 | Mitochondrially Encoded NADH:Ubiquinone Oxidoreductase Core Subunit 1 | 32 |
| PHACTR1 | Phosphatase And Actin Regulator 1 | 37 |
| XK | X-Linked Kx Blood Group | 40 |
| TMPO | Thymopoietin | 45 |
| CALCA | Calcitonin Related Polypeptide Alpha | 43 |
| PYGM | Glycogen Phosphorylase, Muscle Associated | 46 |
| NPHP3 | Nephrocystin 3 | 39 |
| FIG4 | FIG4 Phosphoinositide 5-Phosphatase | 43 |
| CTNNA3 | Catenin Alpha 3 | 39 |
| IGF2 | Insulin Like Growth Factor 2 | 48 |
| GAPDH | Glyceraldehyde-3-Phosphate Dehydrogenase | 48 |
| MTRR | 5-Methyltetrahydrofolate-Homocysteine Methyltransferase Reductase | 43 |
| CALM2 | Calmodulin 2 | 44 |
| EEF1A2 | Eukaryotic Translation Elongation Factor 1 Alpha 2 | 45 |
| GPX1 | Glutathione Peroxidase 1 | 48 |
| TSC2 | TSC Complex Subunit 2 | 50 |
| DNASE1 | Deoxyribonuclease 1 | 41 |
| CCL5 | C-C Motif Chemokine Ligand 5 | 43 |
| NRAS | NRAS Proto-Oncogene, GTPase | 50 |
| BBS4 | Bardet-Biedl Syndrome 4 | 41 |
| IL12B | Interleukin 12B | 44 |
| TNFSF11 | TNF Superfamily Member 11 | 47 |
| BAZ1B | Bromodomain Adjacent To Zinc Finger Domain 1B | 40 |
| GPD1L | Glycerol-3-Phosphate Dehydrogenase 1 Like | 42 |
| APLN | Apelin | 37 |
| DNAJC19 | DnaJ Heat Shock Protein Family (Hsp40) Member C19 | 41 |
| HAMP | Hepcidin Antimicrobial Peptide | 44 |
| CAVIN1 | Caveolae Associated Protein 1 | 32 |
| ACTG1 | Actin Gamma 1 | 50 |
| NPHP1 | Nephrocystin 1 | 43 |
| SH2B3 | SH2B Adaptor Protein 3 | 46 |
| IDS | Iduronate 2-Sulfatase | 48 |
| MSTN | Myostatin | 45 |
| SPARC | Secreted Protein Acidic And Cysteine Rich | 50 |
| AGER | Advanced Glycosylation End-Product Specific Receptor | 44 |
| MAPK14 | Mitogen-Activated Protein Kinase 14 | 51 |
| SERPINA1 | Serpin Family A Member 1 | 49 |
| NFKBIA | NFKB Inhibitor Alpha | 50 |
| MFAP5 | Microfibril Associated Protein 5 | 41 |
| MYH9 | Myosin Heavy Chain 9 | 49 |
| C4A | Complement C4A (Rodgers Blood Group) | 42 |
| MIR92B | MicroRNA 92b | 16 |
| FGFR1 | Fibroblast Growth Factor Receptor 1 | 55 |
| TNFSF4 | TNF Superfamily Member 4 | 40 |
| CP | Ceruloplasmin | 47 |
| CSF3 | Colony Stimulating Factor 3 | 40 |
| KIT | KIT Proto-Oncogene, Receptor Tyrosine Kinase | 53 |
| MIR122 | MicroRNA 122 | 21 |
| ACAN | Aggrecan | 45 |
| MFN2 | Mitofusin 2 | 48 |
| TXNRD2 | Thioredoxin Reductase 2 | 46 |
| FOXE3 | Forkhead Box E3 | 35 |
| IL6ST | Interleukin 6 Signal Transducer | 45 |
| OTC | Ornithine Carbamoyltransferase | 47 |
| MIR30E | MicroRNA 30e | 21 |
| LTBP2 | Latent Transforming Growth Factor Beta Binding Protein 2 | 43 |
| SLC2A4 | Solute Carrier Family 2 Member 4 | 45 |
| MIAT | Myocardial Infarction Associated Transcript | 23 |
| CYP19A1 | Cytochrome P450 Family 19 Subfamily A Member 1 | 48 |
| GATAD1 | GATA Zinc Finger Domain Containing 1 | 37 |
| XYLT2 | Xylosyltransferase 2 | 45 |
| MIR182 | MicroRNA 182 | 20 |
| SDHB | Succinate Dehydrogenase Complex Iron Sulfur Subunit B | 47 |
| GNPTAB | N-Acetylglucosamine-1-Phosphate Transferase Subunits Alpha And Beta | 41 |
| NEK9 | NIMA Related Kinase 9 | 45 |
| CDKN1C | Cyclin Dependent Kinase Inhibitor 1C | 47 |
| APOH | Apolipoprotein H | 44 |
| MIR23A | MicroRNA 23a | 20 |
| HSPA4 | Heat Shock Protein Family A (Hsp70) Member 4 | 41 |
| S100B | S100 Calcium Binding Protein B | 45 |
| ITGB2 | Integrin Subunit Beta 2 | 50 |
| SGCB | Sarcoglycan Beta | 40 |
| RPGR | Retinitis Pigmentosa GTPase Regulator | 40 |
| NSD1 | Nuclear Receptor Binding SET Domain Protein 1 | 43 |
| CD34 | CD34 Molecule | 43 |
| SELL | Selectin L | 42 |
| GUCY1A1 | Guanylate Cyclase 1 Soluble Subunit Alpha 1 | 36 |
| MASP2 | Mannan Binding Lectin Serine Peptidase 2 | 44 |
| CDKN1A | Cyclin Dependent Kinase Inhibitor 1A | 50 |
| GTF2IRD1 | GTF2I Repeat Domain Containing 1 | 42 |
| DCAF8 | DDB1 And CUL4 Associated Factor 8 | 38 |
| DNM2 | Dynamin 2 | 49 |
| GDF15 | Growth Differentiation Factor 15 | 41 |
| MYBPC1 | Myosin Binding Protein C1 | 43 |
| MIR29A | MicroRNA 29a | 21 |
| TFAM | Transcription Factor A, Mitochondrial | 43 |
| MTM1 | Myotubularin 1 | 44 |
| EPHX2 | Epoxide Hydrolase 2 | 47 |
| BTD | Biotinidase | 44 |
| ANKH | ANKH Inorganic Pyrophosphate Transport Regulator | 40 |
| DNAH5 | Dynein Axonemal Heavy Chain 5 | 40 |
| HGD | Homogentisate 1,2-Dioxygenase | 44 |
| FASLG | Fas Ligand | 47 |
| YY1AP1 | YY1 Associated Protein 1 | 38 |
| SGCG | Sarcoglycan Gamma | 42 |
| ARL13B | ADP Ribosylation Factor Like GTPase 13B | 37 |
| COL2A1 | Collagen Type II Alpha 1 Chain | 48 |
| CLU | Clusterin | 46 |
| MIR199A1 | MicroRNA 199a-1 | 18 |
| PIK3CA | Phosphatidylinositol-4,5-Bisphosphate 3-Kinase Catalytic Subunit Alpha | 52 |
| NLRP3 | NLR Family Pyrin Domain Containing 3 | 47 |
| LIMK1 | LIM Domain Kinase 1 | 49 |
| DNM1L | Dynamin 1 Like | 47 |
| SQSTM1 | Sequestosome 1 | 48 |
| EFEMP2 | EGF Containing Fibulin Extracellular Matrix Protein 2 | 43 |
| RERE | Arginine-Glutamic Acid Dipeptide Repeats | 41 |
| HJV | Hemojuvelin BMP Co-Receptor | 35 |
| MEN1 | Menin 1 | 46 |
| GNS | Glucosamine (N-Acetyl)-6-Sulfatase | 44 |
| ADCY5 | Adenylate Cyclase 5 | 48 |
| NRXN1 | Neurexin 1 | 47 |
| MT-ATP6 | Mitochondrially Encoded ATP Synthase Membrane Subunit 6 | 31 |
| GYS1 | Glycogen Synthase 1 | 49 |
| GGCX | Gamma-Glutamyl Carboxylase | 46 |
| NDUFS4 | NADH:Ubiquinone Oxidoreductase Subunit S4 | 44 |
| SDHD | Succinate Dehydrogenase Complex Subunit D | 44 |
| ZNF687 | Zinc Finger Protein 687 | 37 |
| NOS1 | Nitric Oxide Synthase 1 | 49 |
| IFT80 | Intraflagellar Transport 80 | 37 |
| DMPK | DM1 Protein Kinase | 48 |
| ALOX5AP | Arachidonate 5-Lipoxygenase Activating Protein | 44 |
| MIR296 | MicroRNA 296 | 17 |
| FOXC2 | Forkhead Box C2 | 44 |
| PGM1 | Phosphoglucomutase 1 | 48 |
| THBS2 | Thrombospondin 2 | 45 |
| PPBP | Pro-Platelet Basic Protein | 43 |
| DRD2 | Dopamine Receptor D2 | 50 |
| CYCS | Cytochrome C, Somatic | 48 |
| BGN | Biglycan | 43 |
| HLA-A | Major Histocompatibility Complex, Class I, A | 46 |
| TF | Transferrin | 49 |
| NT5E | 5'-Nucleotidase Ecto | 51 |
| CTSA | Cathepsin A | 45 |
| MYRF | Myelin Regulatory Factor | 34 |
| BRCA2 | BRCA2 DNA Repair Associated | 49 |
| IL23R | Interleukin 23 Receptor | 44 |
| EP300 | E1A Binding Protein P300 | 50 |
| PALLD | Palladin, Cytoskeletal Associated Protein | 42 |
| IFIH1 | Interferon Induced With Helicase C Domain 1 | 47 |
| HSPB1 | Heat Shock Protein Family B (Small) Member 1 | 51 |
| HGSNAT | Heparan-Alpha-Glucosaminide N-Acetyltransferase | 39 |
| RAC1 | Rac Family Small GTPase 1 | 49 |
| SLC8A1 | Solute Carrier Family 8 Member A1 | 44 |
| DNAH8 | Dynein Axonemal Heavy Chain 8 | 37 |
| ETFDH | Electron Transfer Flavoprotein Dehydrogenase | 44 |
| CHKB | Choline Kinase Beta | 45 |
| MIR33A | MicroRNA 33a | 18 |
| PTGS1 | Prostaglandin-Endoperoxide Synthase 1 | 46 |
| CLIP2 | CAP-Gly Domain Containing Linker Protein 2 | 37 |
| NKX2-6 | NK2 Homeobox 6 | 36 |
| PIK3R1 | Phosphoinositide-3-Kinase Regulatory Subunit 1 | 51 |
| BBS7 | Bardet-Biedl Syndrome 7 | 38 |
| ITGA7 | Integrin Subunit Alpha 7 | 47 |
| KALRN | Kalirin RhoGEF Kinase | 41 |
| PDGFRA | Platelet Derived Growth Factor Receptor Alpha | 55 |
| NOTCH2 | Notch Receptor 2 | 50 |
| PTCH1 | Patched 1 | 50 |
| LRP8 | LDL Receptor Related Protein 8 | 43 |
| GSN | Gelsolin | 48 |
| BRCA1 | BRCA1 DNA Repair Associated | 50 |
| MC4R | Melanocortin 4 Receptor | 45 |
| NOTCH3 | Notch Receptor 3 | 49 |
| TBL2 | Transducin Beta Like 2 | 39 |
| EPOR | Erythropoietin Receptor | 48 |
| LGALS3 | Galectin 3 | 44 |
| XYLT1 | Xylosyltransferase 1 | 43 |
| MT-ND4 | Mitochondrially Encoded NADH:Ubiquinone Oxidoreductase Core Subunit 4 | 31 |
| GTF2I | General Transcription Factor IIi | 42 |
| MAPK8 | Mitogen-Activated Protein Kinase 8 | 50 |
| ALDH2 | Aldehyde Dehydrogenase 2 Family Member | 50 |
| ANGPT1 | Angiopoietin 1 | 45 |
| FUCA1 | Alpha-L-Fucosidase 1 | 47 |
| CYP2C19 | Cytochrome P450 Family 2 Subfamily C Member 19 | 46 |
| MT-CO3 | Mitochondrially Encoded Cytochrome C Oxidase III | 30 |
| NGF | Nerve Growth Factor | 50 |
| SOD3 | Superoxide Dismutase 3 | 40 |
| CALM3 | Calmodulin 3 | 42 |
| FAH | Fumarylacetoacetate Hydrolase | 47 |
| FGFR2 | Fibroblast Growth Factor Receptor 2 | 54 |
| RB1 | RB Transcriptional Corepressor 1 | 49 |
| NDE1 | NudE Neurodevelopment Protein 1 | 43 |
| POSTN | Periostin | 43 |
| DDX58 | DExD/H-Box Helicase 58 | 47 |
| DDX41 | DEAD-Box Helicase 41 | 43 |
| TSC1 | TSC Complex Subunit 1 | 48 |
| HSD11B1 | Hydroxysteroid 11-Beta Dehydrogenase 1 | 49 |
| APOA4 | Apolipoprotein A4 | 42 |
| MIR424 | MicroRNA 424 | 17 |
| NCF1 | Neutrophil Cytosolic Factor 1 | 48 |
| BANF1 | BAF Nuclear Assembly Factor 1 | 42 |
| MIR196A2 | MicroRNA 196a-2 | 21 |
| ANXA5 | Annexin A5 | 46 |
| SCARB1 | Scavenger Receptor Class B Member 1 | 45 |
| MIR320A | MicroRNA 320a | 20 |
| PHEX | Phosphate Regulating Endopeptidase Homolog X-Linked | 40 |
| BTK | Bruton Tyrosine Kinase | 53 |
| MT-TL1 | Mitochondrially Encoded TRNA-Leu (UUA/G) 1 | 15 |
| SLC9A1 | Solute Carrier Family 9 Member A1 | 51 |
| CREBBP | CREB Binding Protein | 52 |
| COL1A2 | Collagen Type I Alpha 2 Chain | 47 |
| MT-CO2 | Mitochondrially Encoded Cytochrome C Oxidase II | 32 |
| SURF1 | SURF1 Cytochrome C Oxidase Assembly Factor | 43 |
| SPEG | Striated Muscle Enriched Protein Kinase | 41 |
| GYG1 | Glycogenin 1 | 45 |
| SBF2 | SET Binding Factor 2 | 40 |
| DPP6 | Dipeptidyl Peptidase Like 6 | 43 |
| PPP1CB | Protein Phosphatase 1 Catalytic Subunit Beta | 45 |
| SOX9 | SRY-Box Transcription Factor 9 | 47 |
| TECRL | Trans-2,3-Enoyl-CoA Reductase Like | 37 |
| SLC34A3 | Solute Carrier Family 34 Member 3 | 41 |
| ACE2 | Angiotensin I Converting Enzyme 2 | 48 |
| CYP21A2 | Cytochrome P450 Family 21 Subfamily A Member 2 | 45 |
| BBS5 | Bardet-Biedl Syndrome 5 | 39 |
| BBS10 | Bardet-Biedl Syndrome 10 | 40 |
| CHDS3 | Coronary Heart Disease, Susceptibility To, 3 | 2 |
| NOS1AP | Nitric Oxide Synthase 1 Adaptor Protein | 37 |
| SPG7 | SPG7 Matrix AAA Peptidase Subunit, Paraplegin | 43 |
| CHDS2 | Coronary Heart Disease, Susceptibility To, 2 | 2 |
| PARK7 | Parkinsonism Associated Deglycase | 45 |
| MEFV | MEFV Innate Immuity Regulator, Pyrin | 43 |
| CYP17A1 | Cytochrome P450 Family 17 Subfamily A Member 1 | 48 |
| MT-ND5 | Mitochondrially Encoded NADH:Ubiquinone Oxidoreductase Core Subunit 5 | 31 |
| MAT2A | Methionine Adenosyltransferase 2A | 47 |
| SIRT1 | Sirtuin 1 | 49 |
| CSF2 | Colony Stimulating Factor 2 | 44 |
| PDE3A | Phosphodiesterase 3A | 48 |
| PCCA | Propionyl-CoA Carboxylase Subunit Alpha | 45 |
| ATXN2 | Ataxin 2 | 42 |
| PGF | Placental Growth Factor | 43 |
| GUSB | Glucuronidase Beta | 47 |
| TIMP2 | TIMP Metallopeptidase Inhibitor 2 | 44 |
| SMARCA4 | SWI/SNF Related, Matrix Associated, Actin Dependent Regulator Of Chromatin, Subfamily A, Member 4 | 50 |
| CRH | Corticotropin Releasing Hormone | 44 |
| HNF1A | HNF1 Homeobox A | 45 |
| REST | RE1 Silencing Transcription Factor | 44 |
| ERCC4 | ERCC Excision Repair 4, Endonuclease Catalytic Subunit | 45 |
| FOXC1 | Forkhead Box C1 | 41 |
| NPPC | Natriuretic Peptide C | 40 |
| TARDBP | TAR DNA Binding Protein | 45 |
| EDN3 | Endothelin 3 | 47 |
| ADA | Adenosine Deaminase | 51 |
| CEP19 | Centrosomal Protein 19 | 33 |
| ANGPTL3 | Angiopoietin Like 3 | 45 |
| MIR423 | MicroRNA 423 | 18 |
| DOLK | Dolichol Kinase | 39 |
| TREX1 | Three Prime Repair Exonuclease 1 | 42 |
| CDH23 | Cadherin Related 23 | 41 |
| IGF1R | Insulin Like Growth Factor 1 Receptor | 54 |
| HMGB1 | High Mobility Group Box 1 | 44 |
| MSR1 | Macrophage Scavenger Receptor 1 | 46 |
| HAND1 | Heart And Neural Crest Derivatives Expressed 1 | 41 |
| SHBG | Sex Hormone Binding Globulin | 40 |
| FTO | FTO Alpha-Ketoglutarate Dependent Dioxygenase | 44 |
| MT-ND2 | Mitochondrially Encoded NADH:Ubiquinone Oxidoreductase Core Subunit 2 | 32 |
| STAT5B | Signal Transducer And Activator Of Transcription 5B | 49 |
| AKT2 | AKT Serine/Threonine Kinase 2 | 54 |
| MIF | Macrophage Migration Inhibitory Factor | 49 |
| MEG3 | Maternally Expressed 3 | 29 |
| PON3 | Paraoxonase 3 | 44 |
| UMOD | Uromodulin | 41 |
| PRKACA | Protein Kinase CAMP-Activated Catalytic Subunit Alpha | 51 |
| HLA-DQA1 | Major Histocompatibility Complex, Class II, DQ Alpha 1 | 42 |
| CHDS4 | Coronary Heart Disease, Susceptibility To, 4 | 2 |
| SLC1A2 | Solute Carrier Family 1 Member 2 | 48 |
| MRAS | Muscle RAS Oncogene Homolog | 45 |
| KCNA5 | Potassium Voltage-Gated Channel Subfamily A Member 5 | 45 |
| MIR133B | MicroRNA 133b | 21 |
| FHL1 | Four And A Half LIM Domains 1 | 45 |
| LMNB1 | Lamin B1 | 47 |
| ACP5 | Acid Phosphatase 5, Tartrate Resistant | 47 |
| HADHB | Hydroxyacyl-CoA Dehydrogenase Trifunctional Multienzyme Complex Subunit Beta | 47 |
| HLA-DPB1 | Major Histocompatibility Complex, Class II, DP Beta 1 | 43 |
| SMC1A | Structural Maintenance Of Chromosomes 1A | 45 |
| CA8 | Carbonic Anhydrase 8 | 47 |
| CHDS8 | Coronary Heart Disease, Susceptibility To, 8 | 2 |
| CHDS9 | Coronary Heart Disease, Suscpetibility To, 9 | 2 |
| HBA1 | Hemoglobin Subunit Alpha 1 | 42 |
| GCG | Glucagon | 41 |
| G6PC | Glucose-6-Phosphatase Catalytic Subunit | 44 |
| GPC3 | Glypican 3 | 45 |
| DNMT1 | DNA Methyltransferase 1 | 50 |
| GATA3 | GATA Binding Protein 3 | 49 |
| PDGFRB | Platelet Derived Growth Factor Receptor Beta | 55 |
| ATP2A1 | ATPase Sarcoplasmic/Endoplasmic Reticulum Ca2+ Transporting 1 | 48 |
| ARF1 | ADP Ribosylation Factor 1 | 46 |
| SLC20A2 | Solute Carrier Family 20 Member 2 | 46 |
| BBS12 | Bardet-Biedl Syndrome 12 | 35 |
| UTS2 | Urotensin 2 | 41 |
| CHDS1 | Coronary Heart Disease, Susceptibility To, 1 | 2 |
| MT-TK | Mitochondrially Encoded TRNA-Lys (AAA/G) | 14 |
| SELENON | Selenoprotein N | 32 |
| SLC10A2 | Solute Carrier Family 10 Member 2 | 43 |
| FAM20C | FAM20C Golgi Associated Secretory Pathway Kinase | 41 |
| CTSD | Cathepsin D | 52 |
| CASP1 | Caspase 1 | 50 |
| IL6R | Interleukin 6 Receptor | 48 |
| WASHC5 | WASH Complex Subunit 5 | 32 |
| MT-ND6 | Mitochondrially Encoded NADH:Ubiquinone Oxidoreductase Core Subunit 6 | 32 |
| ENO2 | Enolase 2 | 47 |
| ATXN3 | Ataxin 3 | 44 |
| WDR35 | WD Repeat Domain 35 | 38 |
| CTNNA1 | Catenin Alpha 1 | 47 |
| MMP14 | Matrix Metallopeptidase 14 | 51 |
| PAPPA | Pappalysin 1 | 41 |
| DMP1 | Dentin Matrix Acidic Phosphoprotein 1 | 42 |
| MED25 | Mediator Complex Subunit 25 | 39 |
| SHOC2 | SHOC2 Leucine Rich Repeat Scaffold Protein | 41 |
| PSMA6 | Proteasome 20S Subunit Alpha 6 | 46 |
| BCHE | Butyrylcholinesterase | 48 |
| TWNK | Twinkle MtDNA Helicase | 33 |
| TLR5 | Toll Like Receptor 5 | 47 |
| HSPG2 | Heparan Sulfate Proteoglycan 2 | 45 |
| LTBP3 | Latent Transforming Growth Factor Beta Binding Protein 3 | 41 |
| RHOA | Ras Homolog Family Member A | 46 |
| HNF1B | HNF1 Homeobox B | 44 |
| MAPK3 | Mitogen-Activated Protein Kinase 3 | 49 |
| SERPINA6 | Serpin Family A Member 6 | 43 |
| ABCA12 | ATP Binding Cassette Subfamily A Member 12 | 42 |
| ABCC2 | ATP Binding Cassette Subfamily C Member 2 | 47 |
| HSP90AA1 | Heat Shock Protein 90 Alpha Family Class A Member 1 | 48 |
| ARSA | Arylsulfatase A | 47 |
| HNRNPA1 | Heterogeneous Nuclear Ribonucleoprotein A1 | 45 |
| POMGNT1 | Protein O-Linked Mannose N-Acetylglucosaminyltransferase 1 (Beta 1,2-) | 45 |
| GALNT3 | Polypeptide N-Acetylgalactosaminyltransferase 3 | 45 |
| CCND1 | Cyclin D1 | 52 |
| IFT140 | Intraflagellar Transport 140 | 39 |
| TET2 | Tet Methylcytosine Dioxygenase 2 | 44 |
| IFNB1 | Interferon Beta 1 | 41 |
| PDGFB | Platelet Derived Growth Factor Subunit B | 50 |
| COQ2 | Coenzyme Q2, Polyprenyltransferase | 41 |
| TSHR | Thyroid Stimulating Hormone Receptor | 47 |
| GATA1 | GATA Binding Protein 1 | 46 |
| ESR2 | Estrogen Receptor 2 | 49 |
| PPCS | Phosphopantothenoylcysteine Synthetase | 39 |
| LMNB2 | Lamin B2 | 43 |
| NEK8 | NIMA Related Kinase 8 | 41 |
| GCLC | Glutamate-Cysteine Ligase Catalytic Subunit | 43 |
| SLC19A3 | Solute Carrier Family 19 Member 3 | 46 |
| TWIST1 | Twist Family BHLH Transcription Factor 1 | 45 |
| HSPA8 | Heat Shock Protein Family A (Hsp70) Member 8 | 47 |
| RRM2B | Ribonucleotide Reductase Regulatory TP53 Inducible Subunit M2B | 49 |
| DLD | Dihydrolipoamide Dehydrogenase | 50 |
| BCS1L | BCS1 Homolog, Ubiquinol-Cytochrome C Reductase Complex Chaperone | 43 |
| CNR1 | Cannabinoid Receptor 1 | 46 |
| FLT4 | Fms Related Receptor Tyrosine Kinase 4 | 52 |
| TAB2 | TGF-Beta Activated Kinase 1 (MAP3K7) Binding Protein 2 | 47 |
| IGFBP3 | Insulin Like Growth Factor Binding Protein 3 | 45 |
| NAMPT | Nicotinamide Phosphoribosyltransferase | 48 |
| LTBP4 | Latent Transforming Growth Factor Beta Binding Protein 4 | 40 |
| RFC2 | Replication Factor C Subunit 2 | 44 |
| APC | APC Regulator Of WNT Signaling Pathway | 48 |
| CSF1 | Colony Stimulating Factor 1 | 43 |
| BTNL2 | Butyrophilin Like 2 | 39 |
| KIF6 | Kinesin Family Member 6 | 37 |
| PPA2 | Inorganic Pyrophosphatase 2 | 41 |
| PHYH | Phytanoyl-CoA 2-Hydroxylase | 45 |
| NEB | Nebulin | 41 |
| FHL2 | Four And A Half LIM Domains 2 | 45 |
| ADCY10 | Adenylate Cyclase 10 | 45 |
| ACTN1 | Actinin Alpha 1 | 50 |
| HBA2 | Hemoglobin Subunit Alpha 2 | 42 |
| NFKB1 | Nuclear Factor Kappa B Subunit 1 | 52 |
| CASP9 | Caspase 9 | 48 |
| PLAU | Plasminogen Activator, Urokinase | 51 |
| DKK1 | Dickkopf WNT Signaling Pathway Inhibitor 1 | 45 |
| MUC1 | Mucin 1, Cell Surface Associated | 47 |
| CMA1 | Chymase 1 | 43 |
| PLA2G6 | Phospholipase A2 Group VI | 47 |
| DLL4 | Delta Like Canonical Notch Ligand 4 | 45 |
| SEC63 | SEC63 Homolog, Protein Translocation Regulator | 43 |
| COL11A2 | Collagen Type XI Alpha 2 Chain | 44 |
| CLN3 | CLN3 Lysosomal/Endosomal Transmembrane Protein, Battenin | 43 |
| NDUFS2 | NADH:Ubiquinone Oxidoreductase Core Subunit S2 | 44 |
| SARDH | Sarcosine Dehydrogenase | 42 |
| WFS1 | Wolframin ER Transmembrane Glycoprotein | 45 |
| PMS2 | PMS1 Homolog 2, Mismatch Repair System Component | 48 |
| LIPE | Lipase E, Hormone Sensitive Type | 47 |
| PMM2 | Phosphomannomutase 2 | 47 |
| ALPK3 | Alpha Kinase 3 | 36 |
| NAGA | Alpha-N-Acetylgalactosaminidase | 43 |
| NCF2 | Neutrophil Cytosolic Factor 2 | 47 |
| IGFBP1 | Insulin Like Growth Factor Binding Protein 1 | 43 |
| TG | Thyroglobulin | 42 |
| CTF1 | Cardiotrophin 1 | 39 |
| CD14 | CD14 Molecule | 44 |
| SLC40A1 | Solute Carrier Family 40 Member 1 | 46 |
| RUNX1 | RUNX Family Transcription Factor 1 | 48 |
| TFR2 | Transferrin Receptor 2 | 44 |
| ERCC1 | ERCC Excision Repair 1, Endonuclease Non-Catalytic Subunit | 45 |
| CRYAA | Crystallin Alpha A | 44 |
| SFTPC | Surfactant Protein C | 43 |
| DPP4 | Dipeptidyl Peptidase 4 | 50 |
| RMRP | RNA Component Of Mitochondrial RNA Processing Endoribonuclease | 25 |
| CREB1 | CAMP Responsive Element Binding Protein 1 | 48 |
| ACAD9 | Acyl-CoA Dehydrogenase Family Member 9 | 43 |
| GRK2 | G Protein-Coupled Receptor Kinase 2 | 37 |
| SERPIND1 | Serpin Family D Member 1 | 45 |
| SLC19A2 | Solute Carrier Family 19 Member 2 | 46 |
| TRAPPC9 | Trafficking Protein Particle Complex 9 | 39 |
| KAT6B | Lysine Acetyltransferase 6B | 41 |
| VEGFC | Vascular Endothelial Growth Factor C | 47 |
| ACADS | Acyl-CoA Dehydrogenase Short Chain | 46 |
| VDR | Vitamin D Receptor | 51 |
| U2AF1 | U2 Small Nuclear RNA Auxiliary Factor 1 | 41 |
| CFI | Complement Factor I | 46 |
| PTX3 | Pentraxin 3 | 41 |
| XIAP | X-Linked Inhibitor Of Apoptosis | 49 |
| MDM2 | MDM2 Proto-Oncogene | 52 |
| ITGAL | Integrin Subunit Alpha L | 45 |
| PPIG | Peptidylprolyl Isomerase G | 41 |
| CCR2 | C-C Motif Chemokine Receptor 2 | 45 |
| NDUFB11 | NADH:Ubiquinone Oxidoreductase Subunit B11 | 39 |
| MIR106B | MicroRNA 106b | 21 |
| PAX2 | Paired Box 2 | 46 |
| NPC1L1 | NPC1 Like Intracellular Cholesterol Transporter 1 | 44 |
| AGK | Acylglycerol Kinase | 41 |
| CD28 | CD28 Molecule | 47 |
| LITAF | Lipopolysaccharide Induced TNF Factor | 44 |
| NTRK3 | Neurotrophic Receptor Tyrosine Kinase 3 | 51 |
| VIM | Vimentin | 50 |
| GNPTG | N-Acetylglucosamine-1-Phosphate Transferase Subunit Gamma | 39 |
| HTRA1 | HtrA Serine Peptidase 1 | 43 |
| PEX5 | Peroxisomal Biogenesis Factor 5 | 42 |
| LRP2 | LDL Receptor Related Protein 2 | 45 |
| SYP | Synaptophysin | 43 |
| GLI2 | GLI Family Zinc Finger 2 | 47 |
| GCLM | Glutamate-Cysteine Ligase Modifier Subunit | 41 |
| CDC42 | Cell Division Cycle 42 | 51 |
| ERCC2 | ERCC Excision Repair 2, TFIIH Core Complex Helicase Subunit | 47 |
| MLX | MAX Dimerization Protein MLX | 43 |
| HLA-C | Major Histocompatibility Complex, Class I, C | 44 |
| COX10 | Cytochrome C Oxidase Assembly Factor Heme A:Farnesyltransferase COX10 | 43 |
| MYH14 | Myosin Heavy Chain 14 | 45 |
| HNRNPA2B1 | Heterogeneous Nuclear Ribonucleoprotein A2/B1 | 45 |
| CKM | Creatine Kinase, M-Type | 44 |
| ARID1B | AT-Rich Interaction Domain 1B | 44 |
| PYGL | Glycogen Phosphorylase L | 48 |
| CHIT1 | Chitinase 1 | 43 |
| USF1 | Upstream Transcription Factor 1 | 43 |
| CS | Citrate Synthase | 44 |
| MAOA | Monoamine Oxidase A | 50 |
| RBPJ | Recombination Signal Binding Protein For Immunoglobulin Kappa J Region | 47 |
| FLCN | Folliculin | 41 |
| TNNI2 | Troponin I2, Fast Skeletal Type | 44 |
| TMEM70 | Transmembrane Protein 70 | 38 |
| CCM2 | CCM2 Scaffold Protein | 42 |
| CDH1 | Cadherin 1 | 50 |
| PRPS1 | Phosphoribosyl Pyrophosphate Synthetase 1 | 46 |
| PEX2 | Peroxisomal Biogenesis Factor 2 | 43 |
| ZEB2 | Zinc Finger E-Box Binding Homeobox 2 | 48 |
| HADH | Hydroxyacyl-CoA Dehydrogenase | 47 |
| SCN9A | Sodium Voltage-Gated Channel Alpha Subunit 9 | 47 |
| PEX7 | Peroxisomal Biogenesis Factor 7 | 43 |
| THBS1 | Thrombospondin 1 | 44 |
| PSMB8 | Proteasome 20S Subunit Beta 8 | 49 |
| FGF10 | Fibroblast Growth Factor 10 | 47 |
| NTRK1 | Neurotrophic Receptor Tyrosine Kinase 1 | 48 |
| CCDC151 | Coiled-Coil Domain Containing 151 | 37 |
| F2R | Coagulation Factor II Thrombin Receptor | 46 |
| SORT1 | Sortilin 1 | 43 |
| GH1 | Growth Hormone 1 | 44 |
| MYLK2 | Myosin Light Chain Kinase 2 | 47 |
| FLNB | Filamin B | 45 |
| IL33 | Interleukin 33 | 40 |
| SLC4A1 | Solute Carrier Family 4 Member 1 (Diego Blood Group) | 47 |
| TLL1 | Tolloid Like 1 | 43 |
| PEX12 | Peroxisomal Biogenesis Factor 12 | 39 |
| THPO | Thrombopoietin | 42 |
| KRIT1 | KRIT1 Ankyrin Repeat Containing | 41 |
| SCN3B | Sodium Voltage-Gated Channel Beta Subunit 3 | 44 |
| HCRT | Hypocretin Neuropeptide Precursor | 40 |
| PIK3CG | Phosphatidylinositol-4,5-Bisphosphate 3-Kinase Catalytic Subunit Gamma | 48 |
| IFT172 | Intraflagellar Transport 172 | 39 |
| F12 | Coagulation Factor XII | 48 |
| NDUFV2 | NADH:Ubiquinone Oxidoreductase Core Subunit V2 | 44 |
| RAI1 | Retinoic Acid Induced 1 | 40 |
| ANGPT2 | Angiopoietin 2 | 44 |
| ITGB1 | Integrin Subunit Beta 1 | 50 |
| INPP5E | Inositol Polyphosphate-5-Phosphatase E | 41 |
| SUFU | SUFU Negative Regulator Of Hedgehog Signaling | 43 |
| TMEM231 | Transmembrane Protein 231 | 37 |
| FANCA | FA Complementation Group A | 48 |
| SLC22A4 | Solute Carrier Family 22 Member 4 | 43 |
| AIFM1 | Apoptosis Inducing Factor Mitochondria Associated 1 | 49 |
| HTR3A | 5-Hydroxytryptamine Receptor 3A | 45 |
| CLCN5 | Chloride Voltage-Gated Channel 5 | 42 |
| FLI1 | Fli-1 Proto-Oncogene, ETS Transcription Factor | 49 |
| PEX1 | Peroxisomal Biogenesis Factor 1 | 44 |
| MVK | Mevalonate Kinase | 48 |
| TPI1 | Triosephosphate Isomerase 1 | 48 |
| SCN1A | Sodium Voltage-Gated Channel Alpha Subunit 1 | 47 |
| RAD51 | RAD51 Recombinase | 52 |
| PEX6 | Peroxisomal Biogenesis Factor 6 | 43 |
| HDAC9 | Histone Deacetylase 9 | 46 |
| MIR10A | MicroRNA 10a | 21 |
| NDUFB8 | NADH:Ubiquinone Oxidoreductase Subunit B8 | 43 |
| ACHE | Acetylcholinesterase (Cartwright Blood Group) | 45 |
| PLIN1 | Perilipin 1 | 44 |
| CHAT | Choline O-Acetyltransferase | 47 |
| ANO5 | Anoctamin 5 | 38 |
| IL2RB | Interleukin 2 Receptor Subunit Beta | 48 |
| ENO1 | Enolase 1 | 47 |
| DNMT3A | DNA Methyltransferase 3 Alpha | 51 |
| ITGA4 | Integrin Subunit Alpha 4 | 48 |
| KCNQ1OT1 | KCNQ1 Opposite Strand/Antisense Transcript 1 | 25 |
| SFTPB | Surfactant Protein B | 43 |
| C9orf72 | C9orf72-SMCR8 Complex Subunit | 41 |
| SAMHD1 | SAM And HD Domain Containing Deoxynucleoside Triphosphate Triphosphohydrolase 1 | 43 |
| NDUFS3 | NADH:Ubiquinone Oxidoreductase Core Subunit S3 | 46 |
| EPAS1 | Endothelial PAS Domain Protein 1 | 48 |
| HSPA1A | Heat Shock Protein Family A (Hsp70) Member 1A | 43 |
| CFL2 | Cofilin 2 | 44 |
| MMUT | Methylmalonyl-CoA Mutase | 35 |
| AOC3 | Amine Oxidase Copper Containing 3 | 44 |
| CHGA | Chromogranin A | 42 |
| CEP120 | Centrosomal Protein 120 | 40 |
| MMP7 | Matrix Metallopeptidase 7 | 48 |
| ITPKC | Inositol-Trisphosphate 3-Kinase C | 43 |
| TCF7L2 | Transcription Factor 7 Like 2 | 45 |
| LGALS2 | Galectin 2 | 41 |
| TAGLN | Transgelin | 43 |
| GABRA1 | Gamma-Aminobutyric Acid Type A Receptor Subunit Alpha1 | 46 |
| RIT1 | Ras Like Without CAAX 1 | 45 |
| SETD2 | SET Domain Containing 2, Histone Lysine Methyltransferase | 47 |
| NOX4 | NADPH Oxidase 4 | 42 |
| BDKRB2 | Bradykinin Receptor B2 | 44 |
| ADORA2A | Adenosine A2a Receptor | 45 |
| SLC2A1 | Solute Carrier Family 2 Member 1 | 52 |
| RNU4ATAC | RNA, U4atac Small Nuclear (U12-Dependent Splicing) | 20 |
| HSPB8 | Heat Shock Protein Family B (Small) Member 8 | 44 |
| MT-ND3 | Mitochondrially Encoded NADH:Ubiquinone Oxidoreductase Core Subunit 3 | 31 |
| CACNA2D2 | Calcium Voltage-Gated Channel Auxiliary Subunit Alpha2delta 2 | 41 |
| PEX10 | Peroxisomal Biogenesis Factor 10 | 41 |
| NXN | Nucleoredoxin | 41 |
| IKBKG | Inhibitor Of Nuclear Factor Kappa B Kinase Regulatory Subunit Gamma | 48 |
| COL18A1 | Collagen Type XVIII Alpha 1 Chain | 45 |
| HSD17B10 | Hydroxysteroid 17-Beta Dehydrogenase 10 | 45 |
| IL12RB1 | Interleukin 12 Receptor Subunit Beta 1 | 44 |
| APLNR | Apelin Receptor | 43 |
| SETBP1 | SET Binding Protein 1 | 40 |
| S100A12 | S100 Calcium Binding Protein A12 | 39 |
| GNAQ | G Protein Subunit Alpha Q | 49 |
| RAD51C | RAD51 Paralog C | 43 |
| CDH5 | Cadherin 5 | 47 |
| ABCB4 | ATP Binding Cassette Subfamily B Member 4 | 45 |
| ATXN1 | Ataxin 1 | 43 |
| PNLIP | Pancreatic Lipase | 47 |
| PITX3 | Paired Like Homeodomain 3 | 40 |
| APOL1 | Apolipoprotein L1 | 42 |
| CIDEC | Cell Death Inducing DFFA Like Effector C | 41 |
| HBG2 | Hemoglobin Subunit Gamma 2 | 42 |
| PRF1 | Perforin 1 | 45 |
| PDCD10 | Programmed Cell Death 10 | 42 |
| GP1BB | Glycoprotein Ib Platelet Subunit Beta | 41 |
| NEBL | Nebulette | 39 |
| SREBF1 | Sterol Regulatory Element Binding Transcription Factor 1 | 44 |
| MCTP2 | Multiple C2 And Transmembrane Domain Containing 2 | 40 |
| SLC12A3 | Solute Carrier Family 12 Member 3 | 47 |
| IRF5 | Interferon Regulatory Factor 5 | 48 |
| BAX | BCL2 Associated X, Apoptosis Regulator | 48 |
| IDH2 | Isocitrate Dehydrogenase (NADP(+)) 2 | 52 |
| FTL | Ferritin Light Chain | 47 |
| ADORA1 | Adenosine A1 Receptor | 47 |
| DSG1 | Desmoglein 1 | 43 |
| CACNB4 | Calcium Voltage-Gated Channel Auxiliary Subunit Beta 4 | 45 |
| HNF4A | Hepatocyte Nuclear Factor 4 Alpha | 50 |
| TWIST2 | Twist Family BHLH Transcription Factor 2 | 41 |
| MAX | MYC Associated Factor X | 48 |
| BUB1B | BUB1 Mitotic Checkpoint Serine/Threonine Kinase B | 49 |
| NDUFV1 | NADH:Ubiquinone Oxidoreductase Core Subunit V1 | 45 |
| MMP12 | Matrix Metallopeptidase 12 | 44 |
| ABHD5 | Abhydrolase Domain Containing 5, Lysophosphatidic Acid Acyltransferase | 45 |
| HMGCL | 3-Hydroxy-3-Methylglutaryl-CoA Lyase | 46 |
| IBSP | Integrin Binding Sialoprotein | 37 |
| OPA1 | OPA1 Mitochondrial Dynamin Like GTPase | 44 |
| FABP4 | Fatty Acid Binding Protein 4 | 44 |
| STRA6 | Signaling Receptor And Transporter Of Retinol STRA6 | 40 |
| IL3 | Interleukin 3 | 44 |
| POLG2 | DNA Polymerase Gamma 2, Accessory Subunit | 41 |
| UCP2 | Uncoupling Protein 2 | 45 |
| PEX3 | Peroxisomal Biogenesis Factor 3 | 42 |
| HSD11B2 | Hydroxysteroid 11-Beta Dehydrogenase 2 | 45 |
| MMP8 | Matrix Metallopeptidase 8 | 47 |
| CD163 | CD163 Molecule | 42 |
| CHI3L1 | Chitinase 3 Like 1 | 43 |
| AARS2 | Alanyl-TRNA Synthetase 2, Mitochondrial | 42 |
| CKB | Creatine Kinase B | 45 |
| AMPD1 | Adenosine Monophosphate Deaminase 1 | 45 |
| NFATC1 | Nuclear Factor Of Activated T Cells 1 | 47 |
| NDUFA13 | NADH:Ubiquinone Oxidoreductase Subunit A13 | 44 |
| PRL | Prolactin | 44 |
| HIRA | Histone Cell Cycle Regulator | 42 |
| LAMB2 | Laminin Subunit Beta 2 | 44 |
| RNF213 | Ring Finger Protein 213 | 39 |
| SEMA3A | Semaphorin 3A | 45 |
| SOCS1 | Suppressor Of Cytokine Signaling 1 | 43 |
| IFT88 | Intraflagellar Transport 88 | 40 |
| NDUFAF1 | NADH:Ubiquinone Oxidoreductase Complex Assembly Factor 1 | 41 |
| IL1R1 | Interleukin 1 Receptor Type 1 | 45 |
| CPT1A | Carnitine Palmitoyltransferase 1A | 48 |
| CD59 | CD59 Molecule (CD59 Blood Group) | 46 |
| SLC6A2 | Solute Carrier Family 6 Member 2 | 48 |
| CD46 | CD46 Molecule | 46 |
| SKI | SKI Proto-Oncogene | 45 |
| STS | Steroid Sulfatase | 44 |
| KLF5 | Kruppel Like Factor 5 | 44 |
| POMT1 | Protein O-Mannosyltransferase 1 | 45 |
| MIPEP | Mitochondrial Intermediate Peptidase | 42 |
| TK2 | Thymidine Kinase 2 | 42 |
| IL12A | Interleukin 12A | 44 |
| PEX26 | Peroxisomal Biogenesis Factor 26 | 41 |
| S100A1 | S100 Calcium Binding Protein A1 | 41 |
| PEX19 | Peroxisomal Biogenesis Factor 19 | 43 |
| MIR208B | MicroRNA 208b | 19 |
| NDUFS1 | NADH:Ubiquinone Oxidoreductase Core Subunit S1 | 45 |
| NDUFA1 | NADH:Ubiquinone Oxidoreductase Subunit A1 | 44 |
| MSH2 | MutS Homolog 2 | 48 |
| RPS19 | Ribosomal Protein S19 | 48 |
| BCL2L1 | BCL2 Like 1 | 47 |
| CBL | Cbl Proto-Oncogene | 50 |
| MIR132 | MicroRNA 132 | 21 |
| LMOD1 | Leiomodin 1 | 40 |
| CYP27A1 | Cytochrome P450 Family 27 Subfamily A Member 1 | 47 |
| ALG6 | ALG6 Alpha-1,3-Glucosyltransferase | 41 |
| DUOX2 | Dual Oxidase 2 | 43 |
| SRA1 | Steroid Receptor RNA Activator 1 | 37 |
| CRAT | Carnitine O-Acetyltransferase | 44 |
| SST | Somatostatin | 42 |
| LEMD3 | LEM Domain Containing 3 | 41 |
| UFD1 | Ubiquitin Recognition Factor In ER Associated Degradation 1 | 35 |
| BGLAP | Bone Gamma-Carboxyglutamate Protein | 40 |
| PEX13 | Peroxisomal Biogenesis Factor 13 | 40 |
| SMAD2 | SMAD Family Member 2 | 47 |
| PROCR | Protein C Receptor | 42 |
| ABCB11 | ATP Binding Cassette Subfamily B Member 11 | 45 |
| NDUFAF3 | NADH:Ubiquinone Oxidoreductase Complex Assembly Factor 3 | 40 |
| PEX11B | Peroxisomal Biogenesis Factor 11 Beta | 40 |
| PROC | Protein C, Inactivator Of Coagulation Factors Va And VIIIa | 49 |
| IL7R | Interleukin 7 Receptor | 47 |
| CCT7 | Chaperonin Containing TCP1 Subunit 7 | 40 |
| EPG5 | Ectopic P-Granules Autophagy Protein 5 Homolog | 36 |
| FANCD2 | FA Complementation Group D2 | 47 |
| CCR1 | C-C Motif Chemokine Receptor 1 | 45 |
| SULT1A3 | Sulfotransferase Family 1A Member 3 | 38 |
| TYMP | Thymidine Phosphorylase | 46 |
| GSTM1 | Glutathione S-Transferase Mu 1 | 41 |
| HPD | 4-Hydroxyphenylpyruvate Dioxygenase | 45 |
| CD86 | CD86 Molecule | 43 |
| GNE | Glucosamine (UDP-N-Acetyl)-2-Epimerase/N-Acetylmannosamine Kinase | 43 |
| NPHS1 | NPHS1 Adhesion Molecule, Nephrin | 45 |
| TTPA | Alpha Tocopherol Transfer Protein | 41 |
| SOS2 | SOS Ras/Rho Guanine Nucleotide Exchange Factor 2 | 45 |
| IL17F | Interleukin 17F | 41 |
| GNA11 | G Protein Subunit Alpha 11 | 47 |
| LAMA3 | Laminin Subunit Alpha 3 | 44 |
| PEX14 | Peroxisomal Biogenesis Factor 14 | 43 |
| ADRA2C | Adrenoceptor Alpha 2C | 45 |
| OXT | Oxytocin/Neurophysin I Prepropeptide | 40 |
| NDUFAF2 | NADH:Ubiquinone Oxidoreductase Complex Assembly Factor 2 | 40 |
| JUN | Jun Proto-Oncogene, AP-1 Transcription Factor Subunit | 49 |
| CYP11B1 | Cytochrome P450 Family 11 Subfamily B Member 1 | 48 |
| OPTN | Optineurin | 44 |
| NDUFS7 | NADH:Ubiquinone Oxidoreductase Core Subunit S7 | 46 |
| CTSK | Cathepsin K | 48 |
| FKBP1B | FKBP Prolyl Isomerase 1B | 40 |
| FANCG | FA Complementation Group G | 43 |
| FANCB | FA Complementation Group B | 40 |
| COL4A2 | Collagen Type IV Alpha 2 Chain | 44 |
| CTSB | Cathepsin B | 51 |
| PCCB | Propionyl-CoA Carboxylase Subunit Beta | 45 |
| EGLN1 | Egl-9 Family Hypoxia Inducible Factor 1 | 48 |
| NDUFB9 | NADH:Ubiquinone Oxidoreductase Subunit B9 | 44 |
| KCNMA1 | Potassium Calcium-Activated Channel Subfamily M Alpha 1 | 49 |
| ATP6AP2 | ATPase H+ Transporting Accessory Protein 2 | 44 |
| DSCAM | DS Cell Adhesion Molecule | 40 |
| GRN | Granulin Precursor | 47 |
| VTN | Vitronectin | 44 |
| UGT1A1 | UDP Glucuronosyltransferase Family 1 Member A1 | 48 |
| POMT2 | Protein O-Mannosyltransferase 2 | 43 |
| ERCC8 | ERCC Excision Repair 8, CSA Ubiquitin Ligase Complex Subunit | 41 |
| RASA2 | RAS P21 Protein Activator 2 | 40 |
| DNAH11 | Dynein Axonemal Heavy Chain 11 | 41 |
| MPV17 | Mitochondrial Inner Membrane Protein MPV17 | 41 |
| COL7A1 | Collagen Type VII Alpha 1 Chain | 44 |
| HCCS | Holocytochrome C Synthase | 41 |
| FCGR3B | Fc Fragment Of IgG Receptor IIIb | 42 |
| SOCS3 | Suppressor Of Cytokine Signaling 3 | 44 |
| STK11 | Serine/Threonine Kinase 11 | 49 |
| ITGB6 | Integrin Subunit Beta 6 | 47 |
| PEX16 | Peroxisomal Biogenesis Factor 16 | 38 |
| PALB2 | Partner And Localizer Of BRCA2 | 43 |
| OCRL | OCRL Inositol Polyphosphate-5-Phosphatase | 44 |
| CLCN2 | Chloride Voltage-Gated Channel 2 | 45 |
| IFNA1 | Interferon Alpha 1 | 39 |
| MARS1 | Methionyl-TRNA Synthetase 1 | 35 |
| PAX6 | Paired Box 6 | 47 |
| ERBB3 | Erb-B2 Receptor Tyrosine Kinase 3 | 54 |
| PLTP | Phospholipid Transfer Protein | 43 |
| GNRH1 | Gonadotropin Releasing Hormone 1 | 41 |
| EPHB4 | EPH Receptor B4 | 52 |
| YARS2 | Tyrosyl-TRNA Synthetase 2 | 44 |
| ADAM10 | ADAM Metallopeptidase Domain 10 | 52 |
| IL5 | Interleukin 5 | 44 |
| GJC2 | Gap Junction Protein Gamma 2 | 41 |
| SRF | Serum Response Factor | 41 |
| TRIP4 | Thyroid Hormone Receptor Interactor 4 | 40 |
| SCNN1A | Sodium Channel Epithelial 1 Subunit Alpha | 47 |
| NDUFS8 | NADH:Ubiquinone Oxidoreductase Core Subunit S8 | 45 |
| DRD5 | Dopamine Receptor D5 | 47 |
| CHST3 | Carbohydrate Sulfotransferase 3 | 42 |
| ABO | ABO, Alpha 1-3-N-Acetylgalactosaminyltransferase And Alpha 1-3-Galactosyltransferase | 37 |
| HTR1B | 5-Hydroxytryptamine Receptor 1B | 45 |
| COX6B1 | Cytochrome C Oxidase Subunit 6B1 | 43 |
| THBS4 | Thrombospondin 4 | 43 |
| COX15 | Cytochrome C Oxidase Assembly Homolog COX15 | 42 |
| FOXRED1 | FAD Dependent Oxidoreductase Domain Containing 1 | 40 |
| MLYCD | Malonyl-CoA Decarboxylase | 43 |
| TFPI | Tissue Factor Pathway Inhibitor | 44 |
| NPR1 | Natriuretic Peptide Receptor 1 | 45 |
| TNXB | Tenascin XB | 43 |
| MMP21 | Matrix Metallopeptidase 21 | 40 |
| TPM4 | Tropomyosin 4 | 40 |
| CR1 | Complement C3b/C4b Receptor 1 (Knops Blood Group) | 44 |
| CTCF | CCCTC-Binding Factor | 46 |
| NUBPL | Nucleotide Binding Protein Like | 39 |
| HELLS | Helicase, Lymphoid Specific | 45 |
| TNFAIP3 | TNF Alpha Induced Protein 3 | 48 |
| COCH | Cochlin | 42 |
| AKT3 | AKT Serine/Threonine Kinase 3 | 52 |
| KCNE5 | Potassium Voltage-Gated Channel Subfamily E Regulatory Subunit 5 | 32 |
| CAPN3 | Calpain 3 | 47 |
| NDUFA12 | NADH:Ubiquinone Oxidoreductase Subunit A12 | 44 |
| KRT5 | Keratin 5 | 47 |
| ARID1A | AT-Rich Interaction Domain 1A | 44 |
| MC2R | Melanocortin 2 Receptor | 47 |
| RNLS | Renalase, FAD Dependent Amine Oxidase | 38 |
| FANCC | FA Complementation Group C | 47 |
| DPM1 | Dolichyl-Phosphate Mannosyltransferase Subunit 1, Catalytic | 44 |
| IL7 | Interleukin 7 | 42 |
| TMEM126B | Transmembrane Protein 126B | 39 |
| KCNJ1 | Potassium Inwardly Rectifying Channel Subfamily J Member 1 | 47 |
| SREBF2 | Sterol Regulatory Element Binding Transcription Factor 2 | 43 |
| HEY2 | Hes Related Family BHLH Transcription Factor With YRPW Motif 2 | 38 |
| GJA4 | Gap Junction Protein Alpha 4 | 43 |
| ADRA2A | Adrenoceptor Alpha 2A | 46 |
| B9D2 | B9 Domain Containing 2 | 40 |
| NDUFA9 | NADH:Ubiquinone Oxidoreductase Subunit A9 | 43 |
| WDTC1 | WD And Tetratricopeptide Repeats 1 | 34 |
| NDUFAF6 | NADH:Ubiquinone Oxidoreductase Complex Assembly Factor 6 | 35 |
| NTRK2 | Neurotrophic Receptor Tyrosine Kinase 2 | 53 |
| STAR | Steroidogenic Acute Regulatory Protein | 46 |
| P2RX7 | Purinergic Receptor P2X 7 | 45 |
| ETS1 | ETS Proto-Oncogene 1, Transcription Factor | 49 |
| SP1 | Sp1 Transcription Factor | 44 |
| SLMAP | Sarcolemma Associated Protein | 38 |
| PLA2G2A | Phospholipase A2 Group IIA | 45 |
| FANCI | FA Complementation Group I | 41 |
| NHLRC1 | NHL Repeat Containing E3 Ubiquitin Protein Ligase 1 | 40 |
| MIR9-1 | MicroRNA 9-1 | 20 |
| FAT4 | FAT Atypical Cadherin 4 | 40 |
| MHRT | Myosin Heavy Chain Associated RNA Transcript | 10 |
| CHKA | Choline Kinase Alpha | 41 |
| GCH1 | GTP Cyclohydrolase 1 | 47 |
| CDKN1B | Cyclin Dependent Kinase Inhibitor 1B | 48 |
| PRDM6 | PR/SET Domain 6 | 39 |
| NDUFA6 | NADH:Ubiquinone Oxidoreductase Subunit A6 | 44 |
| SKIV2L | Ski2 Like RNA Helicase | 43 |
| ECHS1 | Enoyl-CoA Hydratase, Short Chain 1 | 47 |
| BMPR1B | Bone Morphogenetic Protein Receptor Type 1B | 50 |
| TLR8 | Toll Like Receptor 8 | 47 |
| NDUFB3 | NADH:Ubiquinone Oxidoreductase Subunit B3 | 42 |
| EZH2 | Enhancer Of Zeste 2 Polycomb Repressive Complex 2 Subunit | 54 |
| TGIF1 | TGFB Induced Factor Homeobox 1 | 45 |
| SLC26A4 | Solute Carrier Family 26 Member 4 | 43 |
| GTPBP3 | GTP Binding Protein 3, Mitochondrial | 40 |
| BRD4 | Bromodomain Containing 4 | 44 |
| A2ML1 | Alpha-2-Macroglobulin Like 1 | 39 |
| IRF1 | Interferon Regulatory Factor 1 | 47 |
| SLC25A3 | Solute Carrier Family 25 Member 3 | 45 |
| KCNAB2 | Potassium Voltage-Gated Channel Subfamily A Regulatory Beta Subunit 2 | 43 |
| ARSH | Arylsulfatase Family Member H | 34 |
| RTEL1 | Regulator Of Telomere Elongation Helicase 1 | 40 |
| HES7 | Hes Family BHLH Transcription Factor 7 | 37 |
| MT-TS1 | Mitochondrially Encoded TRNA-Ser (UCN) 1 | 14 |
| NDUFA10 | NADH:Ubiquinone Oxidoreductase Subunit A10 | 44 |
| RELA | RELA Proto-Oncogene, NF-KB Subunit | 50 |
| LIMS2 | LIM Zinc Finger Domain Containing 2 | 40 |
| TBK1 | TANK Binding Kinase 1 | 49 |
| PUF60 | Poly(U) Binding Splicing Factor 60 | 41 |
| STX1A | Syntaxin 1A | 47 |
| XPA | XPA, DNA Damage Recognition And Repair Factor | 45 |
| SOST | Sclerostin | 44 |
| KCNB1 | Potassium Voltage-Gated Channel Subfamily B Member 1 | 47 |
| TLR9 | Toll Like Receptor 9 | 45 |
| AHDC1 | AT-Hook DNA Binding Motif Containing 1 | 36 |
| ADAR | Adenosine Deaminase RNA Specific | 44 |
| MT-TS2 | Mitochondrially Encoded TRNA-Ser (AGU/C) 2 | 13 |
| NDUFAF5 | NADH:Ubiquinone Oxidoreductase Complex Assembly Factor 5 | 36 |
| PDLIM1 | PDZ And LIM Domain 1 | 40 |
| MAPK8IP1 | Mitogen-Activated Protein Kinase 8 Interacting Protein 1 | 45 |
| SCNN1B | Sodium Channel Epithelial 1 Subunit Beta | 48 |
| PHOX2B | Paired Like Homeobox 2B | 43 |
| ATP1A1 | ATPase Na+/K+ Transporting Subunit Alpha 1 | 49 |
| VAC14 | VAC14 Component Of PIKFYVE Complex | 43 |
| EBP | EBP Cholestenol Delta-Isomerase | 43 |
| HOTAIR | HOX Transcript Antisense RNA | 25 |
| VCAN | Versican | 47 |
| FOXH1 | Forkhead Box H1 | 41 |
| MIB1 | Mindbomb E3 Ubiquitin Protein Ligase 1 | 45 |
| TMEM260 | Transmembrane Protein 260 | 34 |
| CD79A | CD79a Molecule | 46 |
| FKBP10 | FKBP Prolyl Isomerase 10 | 40 |
| SPECC1L | Sperm Antigen With Calponin Homology And Coiled-Coil Domains 1 Like | 37 |
| DOCK6 | Dedicator Of Cytokinesis 6 | 40 |
| MIR124-1 | MicroRNA 124-1 | 21 |
| GAS5 | Growth Arrest Specific 5 | 23 |
| MYCN | MYCN Proto-Oncogene, BHLH Transcription Factor | 46 |
| COX7B | Cytochrome C Oxidase Subunit 7B | 40 |
| CXCL10 | C-X-C Motif Chemokine Ligand 10 | 44 |
| FANCF | FA Complementation Group F | 42 |
| ANKRD11 | Ankyrin Repeat Domain 11 | 38 |
| ARVCF | ARVCF Delta Catenin Family Member | 38 |
| MIR133A1 | MicroRNA 133a-1 | 17 |
| ERBB4 | Erb-B2 Receptor Tyrosine Kinase 4 | 55 |
| GPC4 | Glypican 4 | 45 |
| NEDD4L | NEDD4 Like E3 Ubiquitin Protein Ligase | 46 |
| PDSS2 | Decaprenyl Diphosphate Synthase Subunit 2 | 40 |
| CDKN3 | Cyclin Dependent Kinase Inhibitor 3 | 42 |
| RBCK1 | RANBP2-Type And C3HC4-Type Zinc Finger Containing 1 | 43 |
| ABCB7 | ATP Binding Cassette Subfamily B Member 7 | 43 |
| NDUFS6 | NADH:Ubiquinone Oxidoreductase Subunit S6 | 43 |
| KCNIP2 | Potassium Voltage-Gated Channel Interacting Protein 2 | 40 |
| CRKL | CRK Like Proto-Oncogene, Adaptor Protein | 46 |
| GABRD | Gamma-Aminobutyric Acid Type A Receptor Subunit Delta | 45 |
| FANCL | FA Complementation Group L | 45 |
| ATP5F1E | ATP Synthase F1 Subunit Epsilon | 32 |
| VPS13A | Vacuolar Protein Sorting 13 Homolog A | 40 |
| CHRM2 | Cholinergic Receptor Muscarinic 2 | 48 |
| M6PR | Mannose-6-Phosphate Receptor, Cation Dependent | 43 |
| KLF1 | Kruppel Like Factor 1 | 44 |
| CYP27B1 | Cytochrome P450 Family 27 Subfamily B Member 1 | 47 |
| C5 | Complement C5 | 46 |
| SLC39A8 | Solute Carrier Family 39 Member 8 | 43 |
| IKBKB | Inhibitor Of Nuclear Factor Kappa B Kinase Subunit Beta | 52 |
| MTFMT | Mitochondrial Methionyl-TRNA Formyltransferase | 41 |
| HLA-G | Major Histocompatibility Complex, Class I, G | 44 |
| MERTK | MER Proto-Oncogene, Tyrosine Kinase | 50 |
| NLRP1 | NLR Family Pyrin Domain Containing 1 | 44 |
| SPTB | Spectrin Beta, Erythrocytic | 40 |
| NDUFA2 | NADH:Ubiquinone Oxidoreductase Subunit A2 | 42 |
| GMPPB | GDP-Mannose Pyrophosphorylase B | 43 |
| BSND | Barttin CLCNK Type Accessory Subunit Beta | 40 |
| ICOSLG | Inducible T Cell Costimulator Ligand | 39 |
| CFB | Complement Factor B | 45 |
| CD27 | CD27 Molecule | 45 |
| MTPN | Myotrophin | 36 |
| SELPLG | Selectin P Ligand | 42 |
| PDHA1 | Pyruvate Dehydrogenase E1 Subunit Alpha 1 | 48 |
| AHSP | Alpha Hemoglobin Stabilizing Protein | 35 |
| APPL1 | Adaptor Protein, Phosphotyrosine Interacting With PH Domain And Leucine Zipper 1 | 45 |
| PHKA2 | Phosphorylase Kinase Regulatory Subunit Alpha 2 | 45 |
| TERC | Telomerase RNA Component | 28 |
| MNX1 | Motor Neuron And Pancreas Homeobox 1 | 41 |
| RAB23 | RAB23, Member RAS Oncogene Family | 43 |
| LBP | Lipopolysaccharide Binding Protein | 44 |
| BOLA3 | BolA Family Member 3 | 38 |
| FOXO1 | Forkhead Box O1 | 48 |
| MEGF8 | Multiple EGF Like Domains 8 | 37 |
| FADD | Fas Associated Via Death Domain | 47 |
| COQ4 | Coenzyme Q4 | 39 |
| TOP3A | DNA Topoisomerase III Alpha | 42 |
| XRCC2 | X-Ray Repair Cross Complementing 2 | 42 |
| MT-TW | Mitochondrially Encoded TRNA-Trp (UGA/G) | 12 |
| IRS2 | Insulin Receptor Substrate 2 | 44 |
| GJA8 | Gap Junction Protein Alpha 8 | 45 |
| XRCC4 | X-Ray Repair Cross Complementing 4 | 43 |
| PLEKHM2 | Pleckstrin Homology And RUN Domain Containing M2 | 35 |
| RASA1 | RAS P21 Protein Activator 1 | 46 |
| NNT | Nicotinamide Nucleotide Transhydrogenase | 45 |
| MCI2 | Myocardial Infarction, Susceptiblity To, 2 | 2 |
| MT-TF | Mitochondrially Encoded TRNA-Phe (UUU/C) | 14 |
| LTBP1 | Latent Transforming Growth Factor Beta Binding Protein 1 | 43 |
| AIP | Aryl Hydrocarbon Receptor Interacting Protein | 44 |
| TANGO2 | Transport And Golgi Organization 2 Homolog | 35 |
| SLC11A1 | Solute Carrier Family 11 Member 1 | 47 |
| CCDC65 | Coiled-Coil Domain Containing 65 | 36 |
| CHRM3 | Cholinergic Receptor Muscarinic 3 | 48 |
| CPOX | Coproporphyrinogen Oxidase | 43 |
| SEC24C | SEC24 Homolog C, COPII Coat Complex Component | 44 |
| TNFSF12 | TNF Superfamily Member 12 | 40 |
| RREB1 | Ras Responsive Element Binding Protein 1 | 43 |
| C1QBP | Complement C1q Binding Protein | 45 |
| MSX2 | Msh Homeobox 2 | 47 |
| ATPAF2 | ATP Synthase Mitochondrial F1 Complex Assembly Factor 2 | 37 |
| NDUFA11 | NADH:Ubiquinone Oxidoreductase Subunit A11 | 37 |
| ADAMTS7 | ADAM Metallopeptidase With Thrombospondin Type 1 Motif 7 | 40 |
| PTHLH | Parathyroid Hormone Like Hormone | 45 |
| GPR101 | G Protein-Coupled Receptor 101 | 39 |
| USP8 | Ubiquitin Specific Peptidase 8 | 48 |
| SOAT1 | Sterol O-Acyltransferase 1 | 44 |
| MAPK10 | Mitogen-Activated Protein Kinase 10 | 51 |
| CPB2 | Carboxypeptidase B2 | 44 |
| LIPG | Lipase G, Endothelial Type | 43 |
| EPX | Eosinophil Peroxidase | 41 |
| ABCG1 | ATP Binding Cassette Subfamily G Member 1 | 43 |
| POGZ | Pogo Transposable Element Derived With ZNF Domain | 40 |
| BRIP1 | BRCA1 Interacting Protein C-Terminal Helicase 1 | 47 |
| MET | MET Proto-Oncogene, Receptor Tyrosine Kinase | 54 |
| CYP1B1 | Cytochrome P450 Family 1 Subfamily B Member 1 | 48 |
| MESP2 | Mesoderm Posterior BHLH Transcription Factor 2 | 35 |
| CYP3A4 | Cytochrome P450 Family 3 Subfamily A Member 4 | 48 |
| ADRA1A | Adrenoceptor Alpha 1A | 47 |
| SH2D1A | SH2 Domain Containing 1A | 47 |
| BBS9 | Bardet-Biedl Syndrome 9 | 39 |
| GATA2 | GATA Binding Protein 2 | 47 |
| PPOX | Protoporphyrinogen Oxidase | 43 |
| CAMK2D | Calcium/Calmodulin Dependent Protein Kinase II Delta | 48 |
| MST1 | Macrophage Stimulating 1 | 44 |
| SDHAF1 | Succinate Dehydrogenase Complex Assembly Factor 1 | 35 |
| GET1 | Guided Entry Of Tail-Anchored Proteins Factor 1 | 27 |
| FANCE | FA Complementation Group E | 41 |
| DYRK1A | Dual Specificity Tyrosine Phosphorylation Regulated Kinase 1A | 50 |
| ITPA | Inosine Triphosphatase | 47 |
| PTPN1 | Protein Tyrosine Phosphatase Non-Receptor Type 1 | 51 |
| PRKCE | Protein Kinase C Epsilon | 50 |
| DHFR | Dihydrofolate Reductase | 49 |
| RNASEH2C | Ribonuclease H2 Subunit C | 39 |
| AHCY | Adenosylhomocysteinase | 50 |
| HABP2 | Hyaluronan Binding Protein 2 | 44 |
| SERPINH1 | Serpin Family H Member 1 | 45 |
| HDAC4 | Histone Deacetylase 4 | 51 |
| TPO | Thyroid Peroxidase | 48 |
| NOX1 | NADPH Oxidase 1 | 41 |
| YY1 | YY1 Transcription Factor | 48 |
| RTN4 | Reticulon 4 | 44 |
| DLST | Dihydrolipoamide S-Succinyltransferase | 45 |
| MORC2 | MORC Family CW-Type Zinc Finger 2 | 40 |
| CYP3A5 | Cytochrome P450 Family 3 Subfamily A Member 5 | 47 |
| SCNN1G | Sodium Channel Epithelial 1 Subunit Gamma | 46 |
| ANK1 | Ankyrin 1 | 43 |
| TGM2 | Transglutaminase 2 | 48 |
| ERCC3 | ERCC Excision Repair 3, TFIIH Core Complex Helicase Subunit | 47 |
| MIR15A | MicroRNA 15a | 16 |
| ACVR1 | Activin A Receptor Type 1 | 51 |
| TSFM | Ts Translation Elongation Factor, Mitochondrial | 43 |
| DDX11 | DEAD/H-Box Helicase 11 | 43 |
| KCNH1 | Potassium Voltage-Gated Channel Subfamily H Member 1 | 46 |
| TAT | Tyrosine Aminotransferase | 44 |
| ATP6V1A | ATPase H+ Transporting V1 Subunit A | 44 |
| HACD1 | 3-Hydroxyacyl-CoA Dehydratase 1 | 36 |
| RRAS | RAS Related | 43 |
| EIF2AK3 | Eukaryotic Translation Initiation Factor 2 Alpha Kinase 3 | 48 |
| COX20 | Cytochrome C Oxidase Assembly Factor COX20 | 39 |
| FKBP1A | FKBP Prolyl Isomerase 1A | 46 |
| CD2 | CD2 Molecule | 44 |
| PDX1 | Pancreatic And Duodenal Homeobox 1 | 47 |
| MIR20A | MicroRNA 20a | 19 |
| MAP1B | Microtubule Associated Protein 1B | 43 |
| MT-TV | Mitochondrially Encoded TRNA-Val (GUN) | 14 |
| SCO1 | Synthesis Of Cytochrome C Oxidase 1 | 47 |
| PLAG1 | PLAG1 Zinc Finger | 40 |
| SLC29A3 | Solute Carrier Family 29 Member 3 | 43 |
| ETV6 | ETS Variant Transcription Factor 6 | 46 |
| IL21 | Interleukin 21 | 43 |
| ADAMTS4 | ADAM Metallopeptidase With Thrombospondin Type 1 Motif 4 | 42 |
| HAVCR2 | Hepatitis A Virus Cellular Receptor 2 | 43 |
| MGME1 | Mitochondrial Genome Maintenance Exonuclease 1 | 35 |
| USP9X | Ubiquitin Specific Peptidase 9 X-Linked | 47 |
| COL17A1 | Collagen Type XVII Alpha 1 Chain | 43 |
| VPS33A | VPS33A Core Subunit Of CORVET And HOPS Complexes | 41 |
| MT-TE | Mitochondrially Encoded TRNA-Glu (GAA/G) | 12 |
| MT-TH | Mitochondrially Encoded TRNA-His (CAU/C) | 13 |
| THRB | Thyroid Hormone Receptor Beta | 50 |
| MICU1 | Mitochondrial Calcium Uptake 1 | 39 |
| IGFBP7 | Insulin Like Growth Factor Binding Protein 7 | 45 |
| GAL | Galanin And GMAP Prepropeptide | 44 |
| JMJD1C | Jumonji Domain Containing 1C | 40 |
| HRC | Histidine Rich Calcium Binding Protein | 36 |
| ARHGAP31 | Rho GTPase Activating Protein 31 | 40 |
| CTSL | Cathepsin L | 46 |
| TCIRG1 | T Cell Immune Regulator 1, ATPase H+ Transporting V0 Subunit A3 | 45 |
| MIR31 | MicroRNA 31 | 20 |
| MRPL44 | Mitochondrial Ribosomal Protein L44 | 39 |
| SRSF2 | Serine And Arginine Rich Splicing Factor 2 | 40 |
| PDYN | Prodynorphin | 43 |
| ALDH7A1 | Aldehyde Dehydrogenase 7 Family Member A1 | 47 |
| NDUFAF4 | NADH:Ubiquinone Oxidoreductase Complex Assembly Factor 4 | 42 |
| SF3B4 | Splicing Factor 3b Subunit 4 | 43 |
| SUN2 | Sad1 And UNC84 Domain Containing 2 | 37 |
| SERPING1 | Serpin Family G Member 1 | 46 |
| IKZF1 | IKAROS Family Zinc Finger 1 | 47 |
| IRAK1 | Interleukin 1 Receptor Associated Kinase 1 | 50 |
| SLC30A10 | Solute Carrier Family 30 Member 10 | 42 |
| MRPS22 | Mitochondrial Ribosomal Protein S22 | 41 |
| FANCM | FA Complementation Group M | 42 |
| ATN1 | Atrophin 1 | 43 |
| CHUK | Component Of Inhibitor Of Nuclear Factor Kappa B Kinase Complex | 52 |
| STAT6 | Signal Transducer And Activator Of Transcription 6 | 50 |
| IL15 | Interleukin 15 | 40 |
| AGRP | Agouti Related Neuropeptide | 43 |
| XRCC1 | X-Ray Repair Cross Complementing 1 | 43 |
| RHO | Rhodopsin | 47 |
| CXCR3 | C-X-C Motif Chemokine Receptor 3 | 44 |
| TUBB | Tubulin Beta Class I | 49 |
| TFAP2A | Transcription Factor AP-2 Alpha | 47 |
| TBX21 | T-Box Transcription Factor 21 | 45 |
| FTH1 | Ferritin Heavy Chain 1 | 50 |
| CX3CL1 | C-X3-C Motif Chemokine Ligand 1 | 42 |
| SLC34A1 | Solute Carrier Family 34 Member 1 | 44 |
| PIGV | Phosphatidylinositol Glycan Anchor Biosynthesis Class V | 42 |
| NSDHL | NAD(P) Dependent Steroid Dehydrogenase-Like | 44 |
| PLCG1 | Phospholipase C Gamma 1 | 47 |
| CYP1A1 | Cytochrome P450 Family 1 Subfamily A Member 1 | 47 |
| MIR191 | MicroRNA 191 | 20 |
| CLCN1 | Chloride Voltage-Gated Channel 1 | 44 |
| UCP1 | Uncoupling Protein 1 | 44 |
| COMP | Cartilage Oligomeric Matrix Protein | 47 |
| LPXN | Leupaxin | 39 |
| DYRK1B | Dual Specificity Tyrosine Phosphorylation Regulated Kinase 1B | 46 |
| S100A9 | S100 Calcium Binding Protein A9 | 43 |
| ELAC2 | ElaC Ribonuclease Z 2 | 42 |
| MT-TQ | Mitochondrially Encoded TRNA-Gln (CAA/G) | 12 |
| DLL3 | Delta Like Canonical Notch Ligand 3 | 41 |
| UBE2T | Ubiquitin Conjugating Enzyme E2 T | 43 |
| DHCR24 | 24-Dehydrocholesterol Reductase | 45 |
| FBXL4 | F-Box And Leucine Rich Repeat Protein 4 | 39 |
| CACNA1H | Calcium Voltage-Gated Channel Subunit Alpha1 H | 50 |
| C4B | Complement C4B (Chido Blood Group) | 41 |
| KCNA1 | Potassium Voltage-Gated Channel Subfamily A Member 1 | 44 |
| ANK3 | Ankyrin 3 | 44 |
| GZMB | Granzyme B | 45 |
| SLC26A1 | Solute Carrier Family 26 Member 1 | 40 |
| CD44 | CD44 Molecule (Indian Blood Group) | 47 |
| SLX4 | SLX4 Structure-Specific Endonuclease Subunit | 39 |
| VIP | Vasoactive Intestinal Peptide | 44 |
| RARRES2 | Retinoic Acid Receptor Responder 2 | 39 |
| TRIB1 | Tribbles Pseudokinase 1 | 37 |
| COX8A | Cytochrome C Oxidase Subunit 8A | 41 |
| PAX8 | Paired Box 8 | 46 |
| ATG5 | Autophagy Related 5 | 44 |
| CEP164 | Centrosomal Protein 164 | 40 |
| COLQ | Collagen Like Tail Subunit Of Asymmetric Acetylcholinesterase | 39 |
| SERPINF1 | Serpin Family F Member 1 | 44 |
| LEMD2 | LEM Domain Nuclear Envelope Protein 2 | 39 |
| APOC2 | Apolipoprotein C2 | 44 |
| PRPF8 | Pre-MRNA Processing Factor 8 | 41 |
| MIR205 | MicroRNA 205 | 20 |
| NSMCE2 | NSE2 (MMS21) Homolog, SMC5-SMC6 Complex SUMO Ligase | 39 |
| PDSS1 | Decaprenyl Diphosphate Synthase Subunit 1 | 42 |
| ATP1A2 | ATPase Na+/K+ Transporting Subunit Alpha 2 | 47 |
| ANKS6 | Ankyrin Repeat And Sterile Alpha Motif Domain Containing 6 | 38 |
| WARS1 | Tryptophanyl-TRNA Synthetase 1 | 37 |
| COA5 | Cytochrome C Oxidase Assembly Factor 5 | 36 |
| MIR185 | MicroRNA 185 | 21 |
| CGA | Glycoprotein Hormones, Alpha Polypeptide | 43 |
| GAST | Gastrin | 40 |
| MAOB | Monoamine Oxidase B | 43 |
| SUMO1 | Small Ubiquitin Like Modifier 1 | 46 |
| SEMA3C | Semaphorin 3C | 41 |
| COX14 | Cytochrome C Oxidase Assembly Factor COX14 | 37 |
| TNFRSF13C | TNF Receptor Superfamily Member 13C | 44 |
| RAB3GAP2 | RAB3 GTPase Activating Non-Catalytic Protein Subunit 2 | 39 |
| RPS27A | Ribosomal Protein S27a | 43 |
| CALCRL | Calcitonin Receptor Like Receptor | 43 |
| DDIT3 | DNA Damage Inducible Transcript 3 | 45 |
| PPARD | Peroxisome Proliferator Activated Receptor Delta | 47 |
| CDK5 | Cyclin Dependent Kinase 5 | 52 |
| COA8 | Cytochrome C Oxidase Assembly Factor 8 | 27 |
| TMPRSS6 | Transmembrane Serine Protease 6 | 44 |
| TAC1 | Tachykinin Precursor 1 | 43 |
| CPS1 | Carbamoyl-Phosphate Synthase 1 | 45 |
| MIR15B | MicroRNA 15b | 18 |
| TACO1 | Translational Activator Of Cytochrome C Oxidase I | 39 |
| CPLANE1 | Ciliogenesis And Planar Polarity Effector 1 | 28 |
| TUG1 | Taurine Up-Regulated 1 | 22 |
| MMRN1 | Multimerin 1 | 39 |
| NBN | Nibrin | 47 |
| NFKB2 | Nuclear Factor Kappa B Subunit 2 | 52 |
| HPGD | 15-Hydroxyprostaglandin Dehydrogenase | 48 |
| GSTP1 | Glutathione S-Transferase Pi 1 | 50 |
| TIMMDC1 | Translocase Of Inner Mitochondrial Membrane Domain Containing 1 | 36 |
| TOMM40 | Translocase Of Outer Mitochondrial Membrane 40 | 40 |
| ADRA1B | Adrenoceptor Alpha 1B | 46 |
| STAT5A | Signal Transducer And Activator Of Transcription 5A | 45 |
| MIR204 | MicroRNA 204 | 21 |
| RCAN1 | Regulator Of Calcineurin 1 | 43 |
| CERS1 | Ceramide Synthase 1 | 41 |
| SMO | Smoothened, Frizzled Class Receptor | 48 |
| FRAS1 | Fraser Extracellular Matrix Complex Subunit 1 | 39 |
| EXT2 | Exostosin Glycosyltransferase 2 | 46 |
| LOXL1 | Lysyl Oxidase Like 1 | 43 |
| BCR | BCR Activator Of RhoGEF And GTPase | 51 |
| OGDH | Oxoglutarate Dehydrogenase | 45 |
| KBTBD13 | Kelch Repeat And BTB Domain Containing 13 | 32 |
| LPP | LIM Domain Containing Preferred Translocation Partner In Lipoma | 42 |
| VLDLR | Very Low Density Lipoprotein Receptor | 50 |
| ITGA8 | Integrin Subunit Alpha 8 | 42 |
| TRPC3 | Transient Receptor Potential Cation Channel Subfamily C Member 3 | 48 |
| ANG | Angiogenin | 45 |
| WNT5A | Wnt Family Member 5A | 49 |
| PSMD4 | Proteasome 26S Subunit, Non-ATPase 4 | 44 |
| MYOC | Myocilin | 42 |
| MIR93 | MicroRNA 93 | 20 |
| DPH1 | Diphthamide Biosynthesis 1 | 40 |
| PDE9A | Phosphodiesterase 9A | 43 |
| NBAS | NBAS Subunit Of NRZ Tethering Complex | 39 |
| TFEB | Transcription Factor EB | 42 |
| ACKR1 | Atypical Chemokine Receptor 1 (Duffy Blood Group) | 37 |
| TGFA | Transforming Growth Factor Alpha | 46 |
| MAP3K20 | Mitogen-Activated Protein Kinase Kinase Kinase 20 | 39 |
| ITPR3 | Inositol 1,4,5-Trisphosphate Receptor Type 3 | 45 |
| SLC2A9 | Solute Carrier Family 2 Member 9 | 45 |
| COA6 | Cytochrome C Oxidase Assembly Factor 6 | 36 |
| ADCY6 | Adenylate Cyclase 6 | 47 |
| RPL26 | Ribosomal Protein L26 | 43 |
| DICER1 | Dicer 1, Ribonuclease III | 47 |
| PRKAA2 | Protein Kinase AMP-Activated Catalytic Subunit Alpha 2 | 50 |
| UBE3B | Ubiquitin Protein Ligase E3B | 43 |
| CA3 | Carbonic Anhydrase 3 | 41 |
| CDON | Cell Adhesion Associated, Oncogene Regulated | 45 |
| CTC1 | CST Telomere Replication Complex Component 1 | 35 |
| CDH13 | Cadherin 13 | 42 |
| MESP1 | Mesoderm Posterior BHLH Transcription Factor 1 | 36 |
| LIPT1 | Lipoyltransferase 1 | 41 |
| CSNK2A1 | Casein Kinase 2 Alpha 1 | 51 |
| SERPINB2 | Serpin Family B Member 2 | 44 |
| LAMB3 | Laminin Subunit Beta 3 | 45 |
| PRKCD | Protein Kinase C Delta | 53 |
| C2 | Complement C2 | 44 |
| CCL4 | C-C Motif Chemokine Ligand 4 | 40 |
| MIR146B | MicroRNA 146b | 19 |
| SLC5A2 | Solute Carrier Family 5 Member 2 | 47 |
| HMGA2 | High Mobility Group AT-Hook 2 | 44 |
| CSF1R | Colony Stimulating Factor 1 Receptor | 52 |
| SDCCAG8 | SHH Signaling And Ciliogenesis Regulator SDCCAG8 | 41 |
| MTO1 | Mitochondrial TRNA Translation Optimization 1 | 42 |
| S100A8 | S100 Calcium Binding Protein A8 | 42 |
| ROCK1 | Rho Associated Coiled-Coil Containing Protein Kinase 1 | 50 |
| SPRED1 | Sprouty Related EVH1 Domain Containing 1 | 42 |
| PPP3CA | Protein Phosphatase 3 Catalytic Subunit Alpha | 52 |
| SRD5A2 | Steroid 5 Alpha-Reductase 2 | 43 |
| UBR1 | Ubiquitin Protein Ligase E3 Component N-Recognin 1 | 43 |
| COG7 | Component Of Oligomeric Golgi Complex 7 | 39 |
| PIGT | Phosphatidylinositol Glycan Anchor Biosynthesis Class T | 40 |
| MIR29C | MicroRNA 29c | 18 |
| FRZB | Frizzled Related Protein | 41 |
| GIGYF2 | GRB10 Interacting GYF Protein 2 | 39 |
| NRP1 | Neuropilin 1 | 47 |
| EEF2 | Eukaryotic Translation Elongation Factor 2 | 48 |
| HS3ST1 | Heparan Sulfate-Glucosamine 3-Sulfotransferase 1 | 42 |
| COL4A4 | Collagen Type IV Alpha 4 Chain | 43 |
| PDGFA | Platelet Derived Growth Factor Subunit A | 44 |
| GSK3B | Glycogen Synthase Kinase 3 Beta | 50 |
| POMK | Protein O-Mannose Kinase | 36 |
| PCSK1 | Proprotein Convertase Subtilisin/Kexin Type 1 | 45 |
| ENTPD1 | Ectonucleoside Triphosphate Diphosphohydrolase 1 | 46 |
| TLR7 | Toll Like Receptor 7 | 46 |
| C1R | Complement C1r | 46 |
| ATAD3A | ATPase Family AAA Domain Containing 3A | 39 |
| UBE2L3 | Ubiquitin Conjugating Enzyme E2 L3 | 45 |
| ADH1C | Alcohol Dehydrogenase 1C (Class I), Gamma Polypeptide | 42 |
| RAP1A | RAP1A, Member Of RAS Oncogene Family | 47 |
| MYOZ1 | Myozenin 1 | 36 |
| ZFHX3 | Zinc Finger Homeobox 3 | 41 |
| TRPS1 | Transcriptional Repressor GATA Binding 1 | 45 |
| ABCC1 | ATP Binding Cassette Subfamily C Member 1 | 47 |
| TULP1 | TUB Like Protein 1 | 42 |
| TRAF6 | TNF Receptor Associated Factor 6 | 47 |
| TLR6 | Toll Like Receptor 6 | 44 |
| SLC22A12 | Solute Carrier Family 22 Member 12 | 44 |
| MAP3K7 | Mitogen-Activated Protein Kinase Kinase Kinase 7 | 51 |
| WRAP53 | WD Repeat Containing Antisense To TP53 | 41 |
| VPS33B | VPS33B Late Endosome And Lysosome Associated | 41 |
| ACADL | Acyl-CoA Dehydrogenase Long Chain | 43 |
| CLCN7 | Chloride Voltage-Gated Channel 7 | 45 |
| CLEC7A | C-Type Lectin Domain Containing 7A | 44 |
| KLHL41 | Kelch Like Family Member 41 | 37 |
| ADAMTSL1 | ADAMTS Like 1 | 41 |
| SOX18 | SRY-Box Transcription Factor 18 | 40 |
| STIM1 | Stromal Interaction Molecule 1 | 48 |
| ATHS | Atherosclerosis Susceptibility (Lipoprotein Associated) | 4 |
| PCYT1A | Phosphate Cytidylyltransferase 1, Choline, Alpha | 46 |
| TLR1 | Toll Like Receptor 1 | 47 |
| TRPM7 | Transient Receptor Potential Cation Channel Subfamily M Member 7 | 45 |
| CD80 | CD80 Molecule | 41 |
| ABCG2 | ATP Binding Cassette Subfamily G Member 2 (Junior Blood Group) | 50 |
| MIR18A | MicroRNA 18a | 18 |
| PYGB | Glycogen Phosphorylase B | 44 |
| SLC7A7 | Solute Carrier Family 7 Member 7 | 47 |
| KCNN4 | Potassium Calcium-Activated Channel Subfamily N Member 4 | 48 |
| MT-TN | Mitochondrially Encoded TRNA-Asn (AAU/C) | 12 |
| CRLF1 | Cytokine Receptor Like Factor 1 | 41 |
| IRF8 | Interferon Regulatory Factor 8 | 45 |
| DTNBP1 | Dystrobrevin Binding Protein 1 | 41 |
| FABP2 | Fatty Acid Binding Protein 2 | 43 |
| GC | GC Vitamin D Binding Protein | 43 |
| MYH10 | Myosin Heavy Chain 10 | 46 |
| INHBA | Inhibin Subunit Beta A | 45 |
| RFWD3 | Ring Finger And WD Repeat Domain 3 | 40 |
| LTA4H | Leukotriene A4 Hydrolase | 46 |
| MIR342 | MicroRNA 342 | 19 |
| CFLAR | CASP8 And FADD Like Apoptosis Regulator | 46 |
| SYNE4 | Spectrin Repeat Containing Nuclear Envelope Family Member 4 | 32 |
| DHCR7 | 7-Dehydrocholesterol Reductase | 46 |
| MOCS1 | Molybdenum Cofactor Synthesis 1 | 40 |
| LTF | Lactotransferrin | 43 |
| PRKCA | Protein Kinase C Alpha | 50 |
| FCGR3A | Fc Fragment Of IgG Receptor IIIa | 44 |
| POU5F1 | POU Class 5 Homeobox 1 | 47 |
| CAPN1 | Calpain 1 | 49 |
| CISD2 | CDGSH Iron Sulfur Domain 2 | 41 |
| NDUFA4 | NDUFA4 Mitochondrial Complex Associated | 42 |
| SMARCAL1 | SWI/SNF Related, Matrix Associated, Actin Dependent Regulator Of Chromatin, Subfamily A Like 1 | 43 |
| GRIK2 | Glutamate Ionotropic Receptor Kainate Type Subunit 2 | 47 |
| IRAK4 | Interleukin 1 Receptor Associated Kinase 4 | 48 |
| GDF2 | Growth Differentiation Factor 2 | 42 |
| SIRT3 | Sirtuin 3 | 48 |
| MAP3K5 | Mitogen-Activated Protein Kinase Kinase Kinase 5 | 47 |
| PARP1 | Poly(ADP-Ribose) Polymerase 1 | 49 |
| UMPS | Uridine Monophosphate Synthetase | 45 |
| PPP1R3A | Protein Phosphatase 1 Regulatory Subunit 3A | 42 |
| TMEM106B | Transmembrane Protein 106B | 38 |
| CUBN | Cubilin | 46 |
| KLKB1 | Kallikrein B1 | 47 |
| DPM3 | Dolichyl-Phosphate Mannosyltransferase Subunit 3, Regulatory | 40 |
| RNASE3 | Ribonuclease A Family Member 3 | 40 |
| ITGAV | Integrin Subunit Alpha V | 46 |
| WNT1 | Wnt Family Member 1 | 46 |
| GRP | Gastrin Releasing Peptide | 41 |
| NUP188 | Nucleoporin 188 | 35 |
| BVES | Blood Vessel Epicardial Substance | 40 |
| NR0B2 | Nuclear Receptor Subfamily 0 Group B Member 2 | 43 |
| AMH | Anti-Mullerian Hormone | 43 |
| NOG | Noggin | 47 |
| ATP5F1D | ATP Synthase F1 Subunit Delta | 33 |
| PNPLA3 | Patatin Like Phospholipase Domain Containing 3 | 41 |
| RPS26 | Ribosomal Protein S26 | 42 |
| TRPM3 | Transient Receptor Potential Cation Channel Subfamily M Member 3 | 41 |
| RHOD | Ras Homolog Family Member D | 39 |
| FLAD1 | Flavin Adenine Dinucleotide Synthetase 1 | 41 |
| EGR1 | Early Growth Response 1 | 44 |
| ALX4 | ALX Homeobox 4 | 39 |
| ATP6V0A2 | ATPase H+ Transporting V0 Subunit A2 | 44 |
| LIG4 | DNA Ligase 4 | 48 |
| MAD2L2 | Mitotic Arrest Deficient 2 Like 2 | 44 |
| CXCR1 | C-X-C Motif Chemokine Receptor 1 | 43 |
| HOTTIP | HOXA Distal Transcript Antisense RNA | 22 |
| TGFBR3 | Transforming Growth Factor Beta Receptor 3 | 45 |
| COL6A1 | Collagen Type VI Alpha 1 Chain | 43 |
| FGF4 | Fibroblast Growth Factor 4 | 44 |
| C8A | Complement C8 Alpha Chain | 41 |
| CYP2E1 | Cytochrome P450 Family 2 Subfamily E Member 1 | 45 |
| CXADR | CXADR Ig-Like Cell Adhesion Molecule | 43 |
| NTF3 | Neurotrophin 3 | 43 |
| PET100 | PET100 Cytochrome C Oxidase Chaperone | 31 |
| PLCE1 | Phospholipase C Epsilon 1 | 44 |
| HPS1 | HPS1 Biogenesis Of Lysosomal Organelles Complex 3 Subunit 1 | 41 |
| HSPA9 | Heat Shock Protein Family A (Hsp70) Member 9 | 47 |
| NDP | Norrin Cystine Knot Growth Factor NDP | 43 |
| SEMA3D | Semaphorin 3D | 39 |
| CTSH | Cathepsin H | 47 |
| MICA | MHC Class I Polypeptide-Related Sequence A | 39 |
| ITGA6 | Integrin Subunit Alpha 6 | 49 |
| ARID2 | AT-Rich Interaction Domain 2 | 41 |
| SRY | Sex Determining Region Y | 35 |
| AHR | Aryl Hydrocarbon Receptor | 48 |
| NR4A2 | Nuclear Receptor Subfamily 4 Group A Member 2 | 46 |
| CACNA1B | Calcium Voltage-Gated Channel Subunit Alpha1 B | 48 |
| OBSCN | Obscurin, Cytoskeletal Calmodulin And Titin-Interacting RhoGEF | 40 |
| CARD9 | Caspase Recruitment Domain Family Member 9 | 44 |
| PLA2G4A | Phospholipase A2 Group IVA | 49 |
| LFNG | LFNG O-Fucosylpeptide 3-Beta-N-Acetylglucosaminyltransferase | 46 |
| PAFAH1B1 | Platelet Activating Factor Acetylhydrolase 1b Regulatory Subunit 1 | 45 |
| RBBP8 | RB Binding Protein 8, Endonuclease | 44 |
| PPP1R12A | Protein Phosphatase 1 Regulatory Subunit 12A | 42 |
| THRA | Thyroid Hormone Receptor Alpha | 48 |
| ADCY3 | Adenylate Cyclase 3 | 47 |
| PGR | Progesterone Receptor | 50 |
| PRSS1 | Serine Protease 1 | 45 |
| EPB42 | Erythrocyte Membrane Protein Band 4.2 | 39 |
| LOC110806262 | Solute Carrier Family 6 Member 4 Gene Promoter | 1 |
| ICOS | Inducible T Cell Costimulator | 43 |
| LAMC2 | Laminin Subunit Gamma 2 | 45 |
| PROKR2 | Prokineticin Receptor 2 | 41 |
| TNC | Tenascin C | 48 |
| HTR1A | 5-Hydroxytryptamine Receptor 1A | 47 |
| ADAMTS3 | ADAM Metallopeptidase With Thrombospondin Type 1 Motif 3 | 41 |
| IL4I1 | Interleukin 4 Induced 1 | 37 |
| DNAJC13 | DnaJ Heat Shock Protein Family (Hsp40) Member C13 | 37 |
| MIR99A | MicroRNA 99a | 21 |
| CTH | Cystathionine Gamma-Lyase | 50 |
| SLC2A2 | Solute Carrier Family 2 Member 2 | 48 |
| FAN1 | FANCD2 And FANCI Associated Nuclease 1 | 37 |
| IFNA2 | Interferon Alpha 2 | 41 |
| SEMA3E | Semaphorin 3E | 41 |
| NLRP12 | NLR Family Pyrin Domain Containing 12 | 44 |
| KIAA1109 | KIAA1109 | 35 |
| WNT3 | Wnt Family Member 3 | 46 |
| LDHA | Lactate Dehydrogenase A | 51 |
| SUCLG1 | Succinate-CoA Ligase GDP/ADP-Forming Subunit Alpha | 45 |
| HDAC6 | Histone Deacetylase 6 | 51 |
| JPH3 | Junctophilin 3 | 41 |
| AGRN | Agrin | 44 |
| DNAH9 | Dynein Axonemal Heavy Chain 9 | 39 |
| MTHFD1 | Methylenetetrahydrofolate Dehydrogenase, Cyclohydrolase And Formyltetrahydrofolate Synthetase 1 | 44 |
| KIF3A | Kinesin Family Member 3A | 41 |
| ZEB1 | Zinc Finger E-Box Binding Homeobox 1 | 48 |
| AKAP10 | A-Kinase Anchoring Protein 10 | 39 |
| RFC1 | Replication Factor C Subunit 1 | 45 |
| PPIF | Peptidylprolyl Isomerase F | 43 |
| TRAF3IP2 | TRAF3 Interacting Protein 2 | 43 |
| BSG | Basigin (Ok Blood Group) | 44 |
| ADCY9 | Adenylate Cyclase 9 | 44 |
| GLS | Glutaminase | 46 |
| COQ8B | Coenzyme Q8B | 32 |
| TNNI1 | Troponin I1, Slow Skeletal Type | 40 |
| SFTPD | Surfactant Protein D | 44 |
| PSMD12 | Proteasome 26S Subunit, Non-ATPase 12 | 41 |
| APOC1 | Apolipoprotein C1 | 40 |
| PCNA | Proliferating Cell Nuclear Antigen | 51 |
| MIR324 | MicroRNA 324 | 18 |
| DIO2 | Iodothyronine Deiodinase 2 | 40 |
| MT-LIPCAR | Mitochondrially Encoded Long Non-Coding Cardiac Associated RNA | 4 |
| MAP2 | Microtubule Associated Protein 2 | 42 |
| MALAT1 | Metastasis Associated Lung Adenocarcinoma Transcript 1 | 24 |
| MYO7A | Myosin VIIA | 42 |
| GREM1 | Gremlin 1, DAN Family BMP Antagonist | 44 |
| GSTT1 | Glutathione S-Transferase Theta 1 | 32 |
| SLC27A6 | Solute Carrier Family 27 Member 6 | 42 |
| ATP2A3 | ATPase Sarcoplasmic/Endoplasmic Reticulum Ca2+ Transporting 3 | 44 |
| CXCL9 | C-X-C Motif Chemokine Ligand 9 | 39 |
| TNFSF15 | TNF Superfamily Member 15 | 45 |
| TTN-AS1 | TTN Antisense RNA 1 | 15 |
| COL6A2 | Collagen Type VI Alpha 2 Chain | 43 |
| PCSK2 | Proprotein Convertase Subtilisin/Kexin Type 2 | 41 |
| ILK | Integrin Linked Kinase | 46 |
| PVR | PVR Cell Adhesion Molecule | 44 |
| ITGA3 | Integrin Subunit Alpha 3 | 46 |
| GCM2 | Glial Cells Missing Transcription Factor 2 | 41 |
| SELENBP1 | Selenium Binding Protein 1 | 43 |
| PGK1 | Phosphoglycerate Kinase 1 | 48 |
| LAMA1 | Laminin Subunit Alpha 1 | 45 |
| RMND1 | Required For Meiotic Nuclear Division 1 Homolog | 37 |
| FURIN | Furin, Paired Basic Amino Acid Cleaving Enzyme | 46 |
| KRT18 | Keratin 18 | 48 |
| KCNQ3 | Potassium Voltage-Gated Channel Subfamily Q Member 3 | 45 |
| ALG1 | ALG1 Chitobiosyldiphosphodolichol Beta-Mannosyltransferase | 43 |
| CAPN10 | Calpain 10 | 41 |
| CTNND1 | Catenin Delta 1 | 45 |
| IL1RAPL2 | Interleukin 1 Receptor Accessory Protein Like 2 | 37 |
| MPLKIP | M-Phase Specific PLK1 Interacting Protein | 36 |
| TOR1A | Torsin Family 1 Member A | 44 |
| POR | Cytochrome P450 Oxidoreductase | 48 |
| PRKCB | Protein Kinase C Beta | 47 |
| NUP107 | Nucleoporin 107 | 43 |
| GYPA | Glycophorin A (MNS Blood Group) | 45 |
| MRE11 | MRE11 Homolog, Double Strand Break Repair Nuclease | 41 |
| ITGA1 | Integrin Subunit Alpha 1 | 41 |
| CANT1 | Calcium Activated Nucleotidase 1 | 44 |
| PCDH15 | Protocadherin Related 15 | 39 |
| TBXA2R | Thromboxane A2 Receptor | 48 |
| ECM1 | Extracellular Matrix Protein 1 | 43 |
| DZIP1L | DAZ Interacting Zinc Finger Protein 1 Like | 36 |
| MIR130A | MicroRNA 130a | 20 |
| ODC1 | Ornithine Decarboxylase 1 | 47 |
| RAB27A | RAB27A, Member RAS Oncogene Family | 48 |
| FREM1 | FRAS1 Related Extracellular Matrix 1 | 41 |
| IGF2-AS | IGF2 Antisense RNA | 24 |
| SUGCT | Succinyl-CoA:Glutarate-CoA Transferase | 36 |
| STN1 | STN1 Subunit Of CST Complex | 32 |
| BPGM | Bisphosphoglycerate Mutase | 45 |
| DRD1 | Dopamine Receptor D1 | 44 |
| PDLIM3 | PDZ And LIM Domain 3 | 37 |
| UROD | Uroporphyrinogen Decarboxylase | 45 |
| EPRS1 | Glutamyl-Prolyl-TRNA Synthetase 1 | 36 |
| GJA3 | Gap Junction Protein Alpha 3 | 41 |
| CD274 | CD274 Molecule | 44 |
| NCAM1 | Neural Cell Adhesion Molecule 1 | 45 |
| PTS | 6-Pyruvoyltetrahydropterin Synthase | 47 |
| HAVCR1 | Hepatitis A Virus Cellular Receptor 1 | 41 |
| NFATC4 | Nuclear Factor Of Activated T Cells 4 | 44 |
| RNASEH2A | Ribonuclease H2 Subunit A | 43 |
| TJP2 | Tight Junction Protein 2 | 45 |
| TCN2 | Transcobalamin 2 | 44 |
| RHOB | Ras Homolog Family Member B | 44 |
| HBEGF | Heparin Binding EGF Like Growth Factor | 43 |
| SIN3A | SIN3 Transcription Regulator Family Member A | 45 |
| MAFB | MAF BZIP Transcription Factor B | 43 |
| KISS1 | KiSS-1 Metastasis Suppressor | 41 |
| FHIT | Fragile Histidine Triad Diadenosine Triphosphatase | 44 |
| GALNT11 | Polypeptide N-Acetylgalactosaminyltransferase 11 | 41 |
| ANXA6 | Annexin A6 | 43 |
| PAX5 | Paired Box 5 | 46 |
| WWOX | WW Domain Containing Oxidoreductase | 47 |
| MT-TT | Mitochondrially Encoded TRNA-Thr (ACN) | 15 |
| SCD | Stearoyl-CoA Desaturase | 48 |
| HTRA2 | HtrA Serine Peptidase 2 | 47 |
| IL4R | Interleukin 4 Receptor | 47 |
| BACE1-AS | BACE1 Antisense RNA | 13 |
| IGHE | Immunoglobulin Heavy Constant Epsilon | 26 |
| STAP1 | Signal Transducing Adaptor Family Member 1 | 39 |
| MAPK7 | Mitogen-Activated Protein Kinase 7 | 48 |
| ADRA1D | Adrenoceptor Alpha 1D | 45 |
| EFEMP1 | EGF Containing Fibulin Extracellular Matrix Protein 1 | 44 |
| FGF9 | Fibroblast Growth Factor 9 | 44 |
| SYNM | Synemin | 37 |
| ADD3 | Adducin 3 | 41 |
| PHGDH | Phosphoglycerate Dehydrogenase | 49 |
| ADAMTSL4 | ADAMTS Like 4 | 38 |
| KCNMB1 | Potassium Calcium-Activated Channel Subfamily M Regulatory Beta Subunit 1 | 42 |
| TAP1 | Transporter 1, ATP Binding Cassette Subfamily B Member | 47 |
| MIR378A | MicroRNA 378a | 18 |
| VANGL2 | VANGL Planar Cell Polarity Protein 2 | 43 |
| PPP1R1B | Protein Phosphatase 1 Regulatory Inhibitor Subunit 1B | 43 |
| CLPB | Caseinolytic Mitochondrial Matrix Peptidase Chaperone Subunit B | 43 |
| NEUROD1 | Neuronal Differentiation 1 | 44 |
| GPX4 | Glutathione Peroxidase 4 | 47 |
| KCNQ4 | Potassium Voltage-Gated Channel Subfamily Q Member 4 | 44 |
| OGT | O-Linked N-Acetylglucosamine (GlcNAc) Transferase | 44 |
| CD3D | CD3d Molecule | 47 |
| NR1H3 | Nuclear Receptor Subfamily 1 Group H Member 3 | 47 |
| HSPB7 | Heat Shock Protein Family B (Small) Member 7 | 39 |
| GPR35 | G Protein-Coupled Receptor 35 | 43 |
| BRINP3 | BMP/Retinoic Acid Inducible Neural Specific 3 | 28 |
| MIR127 | MicroRNA 127 | 20 |
| RXRA | Retinoid X Receptor Alpha | 49 |
| CHRNA3 | Cholinergic Receptor Nicotinic Alpha 3 Subunit | 44 |
| CH25H | Cholesterol 25-Hydroxylase | 37 |
| RPS10 | Ribosomal Protein S10 | 44 |
| OBSL1 | Obscurin Like Cytoskeletal Adaptor 1 | 38 |
| SPON1 | Spondin 1 | 36 |
| RBFOX1 | RNA Binding Fox-1 Homolog 1 | 37 |
| GK | Glycerol Kinase | 47 |
| CD68 | CD68 Molecule | 40 |
| KISS1R | KISS1 Receptor | 44 |
| NAT2 | N-Acetyltransferase 2 | 43 |
| D2HGDH | D-2-Hydroxyglutarate Dehydrogenase | 41 |
| GRIN1 | Glutamate Ionotropic Receptor NMDA Type Subunit 1 | 49 |
| XRCC5 | X-Ray Repair Cross Complementing 5 | 44 |
| AQP4 | Aquaporin 4 | 45 |
| TXN | Thioredoxin | 45 |
| HMCN1 | Hemicentin 1 | 38 |
| TAP2 | Transporter 2, ATP Binding Cassette Subfamily B Member | 44 |
| AKR1B1 | Aldo-Keto Reductase Family 1 Member B | 47 |
| AMT | Aminomethyltransferase | 45 |
| PROK2 | Prokineticin 2 | 44 |
| ADH1B | Alcohol Dehydrogenase 1B (Class I), Beta Polypeptide | 43 |
| SLC30A8 | Solute Carrier Family 30 Member 8 | 42 |
| MMACHC | Metabolism Of Cobalamin Associated C | 43 |
| PLAUR | Plasminogen Activator, Urokinase Receptor | 44 |
| KCNJ12 | Potassium Inwardly Rectifying Channel Subfamily J Member 12 | 43 |
| MIR148A | MicroRNA 148a | 18 |
| CD244 | CD244 Molecule | 43 |
| MIR23B | MicroRNA 23b | 20 |
| NTS | Neurotensin | 40 |
| SCAP | SREBF Chaperone | 41 |
| PDE4A | Phosphodiesterase 4A | 44 |
| CD209 | CD209 Molecule | 43 |
| MYH7B | Myosin Heavy Chain 7B | 39 |
| SIX5 | SIX Homeobox 5 | 39 |
| HS6ST1 | Heparan Sulfate 6-O-Sulfotransferase 1 | 43 |
| PHB | Prohibitin | 47 |
| NRTN | Neurturin | 40 |
| KRT1 | Keratin 1 | 44 |
| PRPF3 | Pre-MRNA Processing Factor 3 | 43 |
| COL6A3 | Collagen Type VI Alpha 3 Chain | 44 |
| FHOD3 | Formin Homology 2 Domain Containing 3 | 37 |
| MIR197 | MicroRNA 197 | 19 |
| CFP | Complement Factor Properdin | 43 |
| MECOM | MDS1 And EVI1 Complex Locus | 47 |
| GLUL | Glutamate-Ammonia Ligase | 49 |
| NDUFB10 | NADH:Ubiquinone Oxidoreductase Subunit B10 | 41 |
| FGFR4 | Fibroblast Growth Factor Receptor 4 | 51 |
| MIRLET7B | MicroRNA Let-7b | 20 |
| EIF2B4 | Eukaryotic Translation Initiation Factor 2B Subunit Delta | 44 |
| ACAD8 | Acyl-CoA Dehydrogenase Family Member 8 | 45 |
| RPL27 | Ribosomal Protein L27 | 42 |
| TPH2 | Tryptophan Hydroxylase 2 | 48 |
| TNFSF10 | TNF Superfamily Member 10 | 46 |
| PLCG2 | Phospholipase C Gamma 2 | 51 |
| NQO1 | NAD(P)H Quinone Dehydrogenase 1 | 49 |
| PNP | Purine Nucleoside Phosphorylase | 47 |
| MIR494 | MicroRNA 494 | 17 |
| ATG16L1 | Autophagy Related 16 Like 1 | 43 |
| MIR33B | MicroRNA 33b | 18 |
| MIRLET7D | MicroRNA Let-7d | 21 |
| TMSB4X | Thymosin Beta 4 X-Linked | 40 |
| ACTG2 | Actin Gamma 2, Smooth Muscle | 45 |
| APBB1 | Amyloid Beta Precursor Protein Binding Family B Member 1 | 43 |
| FCN3 | Ficolin 3 | 41 |
| LIPI | Lipase I | 37 |
| TIMP4 | TIMP Metallopeptidase Inhibitor 4 | 40 |
| CTSG | Cathepsin G | 44 |
| MIRLET7A1 | MicroRNA Let-7a-1 | 21 |
| CERT1 | Ceramide Transporter 1 | 33 |
| MYDGF | Myeloid Derived Growth Factor | 34 |
| IAPP | Islet Amyloid Polypeptide | 40 |
| CSTB | Cystatin B | 45 |
| MIR30B | MicroRNA 30b | 20 |
| COX4I1 | Cytochrome C Oxidase Subunit 4I1 | 44 |
| NID1 | Nidogen 1 | 41 |
| RNASEH2B | Ribonuclease H2 Subunit B | 37 |
| IL16 | Interleukin 16 | 42 |
| CXCL1 | C-X-C Motif Chemokine Ligand 1 | 43 |
| AOMS1 | Abdominal Obesity-Metabolic Syndrome QTL1 | 2 |
| FBXO32 | F-Box Protein 32 | 40 |
| MIR335 | MicroRNA 335 | 18 |
| PSMB4 | Proteasome 20S Subunit Beta 4 | 45 |
| H3-2 | H3.2 Histone (Putative) | 17 |
| LARGE1 | LARGE Xylosyl- And Glucuronyltransferase 1 | 32 |
| SHANK3 | SH3 And Multiple Ankyrin Repeat Domains 3 | 40 |
| AXL | AXL Receptor Tyrosine Kinase | 51 |
| CDH11 | Cadherin 11 | 48 |
| LIG3 | DNA Ligase 3 | 44 |
| MYBPC2 | Myosin Binding Protein C2 | 39 |
| MIR192 | MicroRNA 192 | 21 |
| TAPVR1 | Total Anomalous Pulmonary Venous Return 1 | 3 |
| KCNJ13 | Potassium Inwardly Rectifying Channel Subfamily J Member 13 | 44 |
| YWHAQ | Tyrosine 3-Monooxygenase/Tryptophan 5-Monooxygenase Activation Protein Theta | 47 |
| PSMB9 | Proteasome 20S Subunit Beta 9 | 46 |
| LAMP1 | Lysosomal Associated Membrane Protein 1 | 43 |
| RARA | Retinoic Acid Receptor Alpha | 50 |
| PDPK1 | 3-Phosphoinositide Dependent Protein Kinase 1 | 48 |
| RLBP1 | Retinaldehyde Binding Protein 1 | 44 |
| BMP1 | Bone Morphogenetic Protein 1 | 45 |
| CNTNAP2 | Contactin Associated Protein 2 | 44 |
| AGGF1 | Angiogenic Factor With G-Patch And FHA Domains 1 | 41 |
| MIR24-1 | MicroRNA 24-1 | 18 |
| BIRC3 | Baculoviral IAP Repeat Containing 3 | 46 |
| CASP7 | Caspase 7 | 50 |
| GOSR2 | Golgi SNAP Receptor Complex Member 2 | 43 |
| PEPD | Peptidase D | 45 |
| HDC | Histidine Decarboxylase | 44 |
| ADORA3 | Adenosine A3 Receptor | 45 |
| ANKRD2 | Ankyrin Repeat Domain 2 | 36 |
| MTHFD1L | Methylenetetrahydrofolate Dehydrogenase (NADP+ Dependent) 1 Like | 41 |
| MIR338 | MicroRNA 338 | 17 |
| SPATA7 | Spermatogenesis Associated 7 | 37 |
| NPR3 | Natriuretic Peptide Receptor 3 | 44 |
| CARS2 | Cysteinyl-TRNA Synthetase 2, Mitochondrial | 41 |
| TGFBI | Transforming Growth Factor Beta Induced | 45 |
| CYP2J2 | Cytochrome P450 Family 2 Subfamily J Member 2 | 44 |
| IFNGR2 | Interferon Gamma Receptor 2 | 43 |
| MASP1 | Mannan Binding Lectin Serine Peptidase 1 | 46 |
| BCAR1 | BCAR1 Scaffold Protein, Cas Family Member | 43 |
| MIR183 | MicroRNA 183 | 18 |
| MIR181B1 | MicroRNA 181b-1 | 19 |
| SHC1 | SHC Adaptor Protein 1 | 45 |
| CCND2 | Cyclin D2 | 50 |
| P4HB | Prolyl 4-Hydroxylase Subunit Beta | 49 |
| IL9 | Interleukin 9 | 43 |
| CENPE | Centromere Protein E | 44 |
| SLPI | Secretory Leukocyte Peptidase Inhibitor | 39 |
| MAGI2 | Membrane Associated Guanylate Kinase, WW And PDZ Domain Containing 2 | 43 |
| ADK | Adenosine Kinase | 50 |
| CCK | Cholecystokinin | 41 |
| ATP1B1 | ATPase Na+/K+ Transporting Subunit Beta 1 | 47 |
| FUT2 | Fucosyltransferase 2 | 44 |
| TARS1 | Threonyl-TRNA Synthetase 1 | 36 |
| CLCN4 | Chloride Voltage-Gated Channel 4 | 43 |
| BHMT | Betaine--Homocysteine S-Methyltransferase | 41 |
| IL18BP | Interleukin 18 Binding Protein | 39 |
| WARS2 | Tryptophanyl TRNA Synthetase 2, Mitochondrial | 44 |
| EDN2 | Endothelin 2 | 40 |
| IGFBP2 | Insulin Like Growth Factor Binding Protein 2 | 43 |
| APELA | Apelin Receptor Early Endogenous Ligand | 19 |
| DDX59 | DEAD-Box Helicase 59 | 37 |
| GRK5 | G Protein-Coupled Receptor Kinase 5 | 43 |
| FNDC5 | Fibronectin Type III Domain Containing 5 | 34 |
| KIF2C | Kinesin Family Member 2C | 43 |
| MYLIP | Myosin Regulatory Light Chain Interacting Protein | 39 |
| SPRY2 | Sprouty RTK Signaling Antagonist 2 | 46 |
| KYNU | Kynureninase | 46 |
| KRT8 | Keratin 8 | 47 |
| TLR10 | Toll Like Receptor 10 | 40 |
| CACNB1 | Calcium Voltage-Gated Channel Auxiliary Subunit Beta 1 | 42 |
| TPH1 | Tryptophan Hydroxylase 1 | 44 |
| NRON | Non-Coding Repressor Of NFAT | 17 |
| CCR3 | C-C Motif Chemokine Receptor 3 | 47 |
| FGF1 | Fibroblast Growth Factor 1 | 48 |
| CAMTA1 | Calmodulin Binding Transcription Activator 1 | 40 |
| HLA-DRB5 | Major Histocompatibility Complex, Class II, DR Beta 5 | 40 |
| TELO2 | Telomere Maintenance 2 | 40 |
| ALPP | Alkaline Phosphatase, Placental | 46 |
| IDO1 | Indoleamine 2,3-Dioxygenase 1 | 45 |
| TCF21 | Transcription Factor 21 | 36 |
| COG5 | Component Of Oligomeric Golgi Complex 5 | 39 |
| MUC16 | Mucin 16, Cell Surface Associated | 36 |
| IVD | Isovaleryl-CoA Dehydrogenase | 45 |
| FADS1 | Fatty Acid Desaturase 1 | 44 |
| TLN1 | Talin 1 | 41 |
| PRICKLE1 | Prickle Planar Cell Polarity Protein 1 | 42 |
| CAMK2G | Calcium/Calmodulin Dependent Protein Kinase II Gamma | 47 |
| ADIPOR1 | Adiponectin Receptor 1 | 44 |
| THRIL | TNF And HNRNPL Related Immunoregulatory Long Non-Coding RNA | 11 |
| SHOX | Short Stature Homeobox | 38 |
| GPC6 | Glypican 6 | 43 |
| FAM13A | Family With Sequence Similarity 13 Member A | 37 |
| SEMA5A | Semaphorin 5A | 41 |
| ATR | ATR Serine/Threonine Kinase | 51 |
| INPPL1 | Inositol Polyphosphate Phosphatase Like 1 | 49 |
| BECN1 | Beclin 1 | 46 |
| CD69 | CD69 Molecule | 40 |
| CYSLTR2 | Cysteinyl Leukotriene Receptor 2 | 47 |
| GPX3 | Glutathione Peroxidase 3 | 44 |
| DSPP | Dentin Sialophosphoprotein | 37 |
| ENPP3 | Ectonucleotide Pyrophosphatase/Phosphodiesterase 3 | 43 |
| EZR | Ezrin | 45 |
| CALB2 | Calbindin 2 | 40 |
| DOK7 | Docking Protein 7 | 39 |
| MYOM1 | Myomesin 1 | 38 |
| NR1H2 | Nuclear Receptor Subfamily 1 Group H Member 2 | 48 |
| CCR7 | C-C Motif Chemokine Receptor 7 | 45 |
| ADAMTS17 | ADAM Metallopeptidase With Thrombospondin Type 1 Motif 17 | 40 |
| CYP24A1 | Cytochrome P450 Family 24 Subfamily A Member 1 | 47 |
| CD38 | CD38 Molecule | 45 |
| PRKCH | Protein Kinase C Eta | 50 |
| SH2B1 | SH2B Adaptor Protein 1 | 43 |
| RELB | RELB Proto-Oncogene, NF-KB Subunit | 45 |
| FSTL1 | Follistatin Like 1 | 41 |
| RARS2 | Arginyl-TRNA Synthetase 2, Mitochondrial | 42 |
| HULC | Hepatocellular Carcinoma Up-Regulated Long Non-Coding RNA | 21 |
| BMP7 | Bone Morphogenetic Protein 7 | 45 |
| PROS1 | Protein S | 47 |
| GATM | Glycine Amidinotransferase | 45 |
| MGAM | Maltase-Glucoamylase | 41 |
| TNFRSF10B | TNF Receptor Superfamily Member 10b | 50 |
| KLF4 | Kruppel Like Factor 4 | 45 |
| HSPA5 | Heat Shock Protein Family A (Hsp70) Member 5 | 47 |
| OPRM1 | Opioid Receptor Mu 1 | 48 |
| CD247 | CD247 Molecule | 49 |
| MRPS14 | Mitochondrial Ribosomal Protein S14 | 35 |
| IL11 | Interleukin 11 | 41 |
| MAP3K1 | Mitogen-Activated Protein Kinase Kinase Kinase 1 | 49 |
| CFL1 | Cofilin 1 | 45 |
| AKAP13 | A-Kinase Anchoring Protein 13 | 44 |
| PDXK | Pyridoxal Kinase | 47 |
| FGF14 | Fibroblast Growth Factor 14 | 44 |
| ADIPOR2 | Adiponectin Receptor 2 | 43 |
| MIR30D | MicroRNA 30d | 17 |
| LRRC10 | Leucine Rich Repeat Containing 10 | 34 |
| PKP1 | Plakophilin 1 | 41 |
| PER2 | Period Circadian Regulator 2 | 43 |
| JCAD | Junctional Cadherin 5 Associated | 26 |
| SDC1 | Syndecan 1 | 43 |
| CACNA2D3 | Calcium Voltage-Gated Channel Auxiliary Subunit Alpha2delta 3 | 40 |
| CCL17 | C-C Motif Chemokine Ligand 17 | 38 |
| PML | PML Nuclear Body Scaffold | 45 |
| AMBP | Alpha-1-Microglobulin/Bikunin Precursor | 41 |
| IL1RL1 | Interleukin 1 Receptor Like 1 | 41 |
| MYOM2 | Myomesin 2 | 39 |
| P2RX1 | Purinergic Receptor P2X 1 | 43 |
| LACTB | Lactamase Beta | 36 |
| WFDC21P | WAP Four-Disulfide Core Domain 21, Pseudogene | 12 |
| PRKDC | Protein Kinase, DNA-Activated, Catalytic Subunit | 49 |
| ACP1 | Acid Phosphatase 1 | 44 |
| TUBB4A | Tubulin Beta 4A Class IVa | 45 |
| TBX6 | T-Box Transcription Factor 6 | 39 |
| MIRLET7E | MicroRNA Let-7e | 20 |
| SUN1 | Sad1 And UNC84 Domain Containing 1 | 37 |
| TUBB1 | Tubulin Beta 1 Class VI | 47 |
| PDLIM5 | PDZ And LIM Domain 5 | 39 |
| OCLN | Occludin | 44 |
| CXCR2 | C-X-C Motif Chemokine Receptor 2 | 48 |
| TSPO | Translocator Protein | 43 |
| COL12A1 | Collagen Type XII Alpha 1 Chain | 41 |
| ATIC | 5-Aminoimidazole-4-Carboxamide Ribonucleotide Formyltransferase/IMP Cyclohydrolase | 45 |
| GJC1 | Gap Junction Protein Gamma 1 | 42 |
| ITIH4 | Inter-Alpha-Trypsin Inhibitor Heavy Chain 4 | 42 |
| EPHX1 | Epoxide Hydrolase 1 | 47 |
| CIB1 | Calcium And Integrin Binding 1 | 41 |
| TP53BP1 | Tumor Protein P53 Binding Protein 1 | 44 |
| WNT7A | Wnt Family Member 7A | 49 |
| CTSF | Cathepsin F | 46 |
| ITGB5 | Integrin Subunit Beta 5 | 45 |
| RPS6KB1 | Ribosomal Protein S6 Kinase B1 | 49 |
| HOPX | HOP Homeobox | 37 |
| NOTCH4 | Notch Receptor 4 | 45 |
| MCL1 | MCL1 Apoptosis Regulator, BCL2 Family Member | 47 |
| KLK3 | Kallikrein Related Peptidase 3 | 46 |
| MSH3 | MutS Homolog 3 | 43 |
| H19-ICR | H19/IGF2 Imprinting Control Region | 5 |
| GTF2E2 | General Transcription Factor IIE Subunit 2 | 43 |
| MOCOS | Molybdenum Cofactor Sulfurase | 42 |
| AIF1 | Allograft Inflammatory Factor 1 | 39 |
| FGF3 | Fibroblast Growth Factor 3 | 44 |
| GRK1 | G Protein-Coupled Receptor Kinase 1 | 42 |
| MYLK3 | Myosin Light Chain Kinase 3 | 41 |
| DAOA | D-Amino Acid Oxidase Activator | 33 |
| SFRP4 | Secreted Frizzled Related Protein 4 | 43 |
| LMO7 | LIM Domain 7 | 40 |
| TXNIP | Thioredoxin Interacting Protein | 37 |
| CD151 | CD151 Molecule (Raph Blood Group) | 45 |
| HEY1 | Hes Related Family BHLH Transcription Factor With YRPW Motif 1 | 43 |
| TRPV1 | Transient Receptor Potential Cation Channel Subfamily V Member 1 | 46 |
| MX1 | MX Dynamin Like GTPase 1 | 41 |
| FADS2 | Fatty Acid Desaturase 2 | 44 |
| PUS1 | Pseudouridine Synthase 1 | 41 |
| STUB1 | STIP1 Homology And U-Box Containing Protein 1 | 45 |
| ARNTL | Aryl Hydrocarbon Receptor Nuclear Translocator Like | 42 |
| SLC27A1 | Solute Carrier Family 27 Member 1 | 41 |
| MAP2K7 | Mitogen-Activated Protein Kinase Kinase 7 | 45 |
| HTR2B | 5-Hydroxytryptamine Receptor 2B | 43 |
| DDAH2 | Dimethylarginine Dimethylaminohydrolase 2 | 42 |
| RAC2 | Rac Family Small GTPase 2 | 51 |
| TRPA1 | Transient Receptor Potential Cation Channel Subfamily A Member 1 | 45 |
| GTF2H5 | General Transcription Factor IIH Subunit 5 | 40 |
| FOXO3 | Forkhead Box O3 | 44 |
| MIR25 | MicroRNA 25 | 20 |
| MIR26B | MicroRNA 26b | 21 |
| EIF2B2 | Eukaryotic Translation Initiation Factor 2B Subunit Beta | 44 |
| RAB3GAP1 | RAB3 GTPase Activating Protein Catalytic Subunit 1 | 40 |
| MIR371A | MicroRNA 371a | 16 |
| IFNGR1 | Interferon Gamma Receptor 1 | 50 |
| ACTN3 | Actinin Alpha 3 | 39 |
| HRH2 | Histamine Receptor H2 | 44 |
| COG6 | Component Of Oligomeric Golgi Complex 6 | 37 |
| SLC22A3 | Solute Carrier Family 22 Member 3 | 44 |
| SLC25A1 | Solute Carrier Family 25 Member 1 | 46 |
| MYSM1 | Myb Like, SWIRM And MPN Domains 1 | 39 |
| MS4A1 | Membrane Spanning 4-Domains A1 | 46 |
| PRKAA1 | Protein Kinase AMP-Activated Catalytic Subunit Alpha 1 | 47 |
| LMOD2 | Leiomodin 2 | 33 |
| CYP4F2 | Cytochrome P450 Family 4 Subfamily F Member 2 | 44 |
| MIR10B | MicroRNA 10b | 21 |
| HMOX2 | Heme Oxygenase 2 | 47 |
| MIR451A | MicroRNA 451a | 17 |
| MIR331 | MicroRNA 331 | 18 |
| DIABLO | Diablo IAP-Binding Mitochondrial Protein | 47 |
| AMPD3 | Adenosine Monophosphate Deaminase 3 | 45 |
| RGS5 | Regulator Of G Protein Signaling 5 | 40 |
| UCP3 | Uncoupling Protein 3 | 43 |
| QDPR | Quinoid Dihydropteridine Reductase | 48 |
| NRAP | Nebulin Related Anchoring Protein | 36 |
| RNPC3 | RNA Binding Region (RNP1, RRM) Containing 3 | 35 |
| LIFR | LIF Receptor Subunit Alpha | 47 |
| LEPQTL1 | Leptin, Serum Levels Of | 3 |
| AP3B1 | Adaptor Related Protein Complex 3 Subunit Beta 1 | 45 |
| ALDH1A2 | Aldehyde Dehydrogenase 1 Family Member A2 | 47 |
| ANKRD23 | Ankyrin Repeat Domain 23 | 31 |
| CSNK1D | Casein Kinase 1 Delta | 50 |
| ALOX15 | Arachidonate 15-Lipoxygenase | 44 |
| CCL26 | C-C Motif Chemokine Ligand 26 | 37 |
| TECPR2 | Tectonin Beta-Propeller Repeat Containing 2 | 35 |
| GLMN | Glomulin, FKBP Associated Protein | 40 |
| GCKR | Glucokinase Regulator | 40 |
| IL22 | Interleukin 22 | 41 |
| COL14A1 | Collagen Type XIV Alpha 1 Chain | 42 |
| BUD23 | BUD23 RRNA Methyltransferase And Ribosome Maturation Factor | 30 |
| NUP133 | Nucleoporin 133 | 40 |
| ASCL1 | Achaete-Scute Family BHLH Transcription Factor 1 | 44 |
| PHOSPHO1 | Phosphoethanolamine/Phosphocholine Phosphatase 1 | 39 |
| PKD2L1 | Polycystin 2 Like 1, Transient Receptor Potential Cation Channel | 41 |
| EIF2AK2 | Eukaryotic Translation Initiation Factor 2 Alpha Kinase 2 | 45 |
| MLH3 | MutL Homolog 3 | 42 |
| MIR224 | MicroRNA 224 | 17 |
| XRCC3 | X-Ray Repair Cross Complementing 3 | 41 |
| NDST1 | N-Deacetylase And N-Sulfotransferase 1 | 45 |
| STAG3 | Stromal Antigen 3 | 41 |
| MIR1-2 | MicroRNA 1-2 | 17 |
| SPAG17 | Sperm Associated Antigen 17 | 34 |
| MIR19A | MicroRNA 19a | 19 |
| PTGDS | Prostaglandin D2 Synthase | 45 |
| IFI27 | Interferon Alpha Inducible Protein 27 | 37 |
| IL17RC | Interleukin 17 Receptor C | 40 |
| CEL | Carboxyl Ester Lipase | 46 |
| FGF16 | Fibroblast Growth Factor 16 | 41 |
| SPRY4 | Sprouty RTK Signaling Antagonist 4 | 44 |
| DMRT1 | Doublesex And Mab-3 Related Transcription Factor 1 | 40 |
| NES | Nestin | 39 |
| ENSG00000225544 |  | 6 |
| SCGB1A1 | Secretoglobin Family 1A Member 1 | 40 |
| MYO18B | Myosin XVIIIB | 38 |
| DPYD | Dihydropyrimidine Dehydrogenase | 51 |
| ITLN1 | Intelectin 1 | 39 |
| EMC10 | ER Membrane Protein Complex Subunit 10 | 32 |
| MYOM3 | Myomesin 3 | 33 |
| CNNM2 | Cyclin And CBS Domain Divalent Metal Cation Transport Mediator 2 | 41 |
| SIK1 | Salt Inducible Kinase 1 | 46 |
| TRPC1 | Transient Receptor Potential Cation Channel Subfamily C Member 1 | 42 |
| SOX17 | SRY-Box Transcription Factor 17 | 43 |
| ABRAXAS2 | Abraxas 2, BRISC Complex Subunit | 28 |
| MIR130B | MicroRNA 130b | 17 |
| SGK1 | Serum/Glucocorticoid Regulated Kinase 1 | 48 |
| LCT | Lactase | 43 |
| AEBP1 | AE Binding Protein 1 | 39 |
| RIPPLY2 | Ripply Transcriptional Repressor 2 | 37 |
| HTR2C | 5-Hydroxytryptamine Receptor 2C | 48 |
| TOP1 | DNA Topoisomerase I | 48 |
| CNTF | Ciliary Neurotrophic Factor | 41 |
| UCN | Urocortin | 37 |
| NGFR | Nerve Growth Factor Receptor | 45 |
| KCNN2 | Potassium Calcium-Activated Channel Subfamily N Member 2 | 43 |
| CHRDL1 | Chordin Like 1 | 40 |
| GRIK1 | Glutamate Ionotropic Receptor Kainate Type Subunit 1 | 45 |
| SYK | Spleen Associated Tyrosine Kinase | 50 |
| ASH1L | ASH1 Like Histone Lysine Methyltransferase | 41 |
| MIR134 | MicroRNA 134 | 18 |
| SLC19A1 | Solute Carrier Family 19 Member 1 | 45 |
| PLXND1 | Plexin D1 | 40 |
| RNASEH1 | Ribonuclease H1 | 44 |
| TNFRSF8 | TNF Receptor Superfamily Member 8 | 42 |
| OSM | Oncostatin M | 43 |
| CHRM1 | Cholinergic Receptor Muscarinic 1 | 45 |
| ARSJ | Arylsulfatase Family Member J | 34 |
| LAMB1 | Laminin Subunit Beta 1 | 47 |
| GRIA1 | Glutamate Ionotropic Receptor AMPA Type Subunit 1 | 48 |
| BCL11B | BAF Chromatin Remodeling Complex Subunit BCL11B | 42 |
| LIAS | Lipoic Acid Synthetase | 44 |
| FGF12 | Fibroblast Growth Factor 12 | 43 |
| PPP1R17 | Protein Phosphatase 1 Regulatory Subunit 17 | 36 |
| BPI | Bactericidal Permeability Increasing Protein | 41 |
| AQP1 | Aquaporin 1 (Colton Blood Group) | 45 |
| MCPH1 | Microcephalin 1 | 40 |
| HECTD4 | HECT Domain E3 Ubiquitin Protein Ligase 4 | 31 |
| CELSR2 | Cadherin EGF LAG Seven-Pass G-Type Receptor 2 | 40 |
| H2AX | H2A.X Variant Histone | 35 |
| POLD1 | DNA Polymerase Delta 1, Catalytic Subunit | 45 |
| CILP | Cartilage Intermediate Layer Protein | 40 |
| H6PD | Hexose-6-Phosphate Dehydrogenase/Glucose 1-Dehydrogenase | 41 |
| TNFRSF4 | TNF Receptor Superfamily Member 4 | 43 |
| IL37 | Interleukin 37 | 37 |
| KIF20A | Kinesin Family Member 20A | 39 |
| A2M | Alpha-2-Macroglobulin | 45 |
| ABCA7 | ATP Binding Cassette Subfamily A Member 7 | 43 |
| MIR328 | MicroRNA 328 | 18 |
| STXBP2 | Syntaxin Binding Protein 2 | 44 |
| PTPA | Protein Phosphatase 2 Phosphatase Activator | 35 |
| CYP1A2 | Cytochrome P450 Family 1 Subfamily A Member 2 | 45 |
| GHSR | Growth Hormone Secretagogue Receptor | 47 |
| PRPF31 | Pre-MRNA Processing Factor 31 | 42 |
| RNF113A | Ring Finger Protein 113A | 37 |
| BIRC5 | Baculoviral IAP Repeat Containing 5 | 47 |
| GFER | Growth Factor, Augmenter Of Liver Regeneration | 45 |
| MYO6 | Myosin VI | 45 |
| CAPN5 | Calpain 5 | 43 |
| CALR3 | Calreticulin 3 | 37 |
| PHF6 | PHD Finger Protein 6 | 40 |
| UBC | Ubiquitin C | 43 |
| VRK1 | VRK Serine/Threonine Kinase 1 | 48 |
| COQ10A | Coenzyme Q10A | 35 |
| GLP1R | Glucagon Like Peptide 1 Receptor | 46 |
| TP53COR1 | Tumor Protein P53 Pathway Corepressor 1 | 8 |
| BNC2 | Basonuclin 2 | 39 |
| CFHR2 | Complement Factor H Related 2 | 37 |
| ITGAX | Integrin Subunit Alpha X | 44 |
| ITGA5 | Integrin Subunit Alpha 5 | 48 |
| FDXR | Ferredoxin Reductase | 44 |
| MYO9A | Myosin IXA | 39 |
| AAT1 | Aortic Aneurysm, Familial Thoracic 1 | 3 |
| AK1 | Adenylate Kinase 1 | 47 |
| TARID | TCF21 Antisense RNA Inducing Promoter Demethylation | 15 |
| VEGFB | Vascular Endothelial Growth Factor B | 44 |
| MIRLET7G | MicroRNA Let-7g | 20 |
| TBC1D7 | TBC1 Domain Family Member 7 | 41 |
| FBLN2 | Fibulin 2 | 43 |
| MACROD2 | Mono-ADP Ribosylhydrolase 2 | 35 |
| IL18R1 | Interleukin 18 Receptor 1 | 43 |
| YWHAB | Tyrosine 3-Monooxygenase/Tryptophan 5-Monooxygenase Activation Protein Beta | 48 |
| ITGB1BP2 | Integrin Subunit Beta 1 Binding Protein 2 | 36 |
| MTAP | Methylthioadenosine Phosphorylase | 47 |
| DCC | DCC Netrin 1 Receptor | 46 |
| NEUROG3 | Neurogenin 3 | 41 |
| ALOX12 | Arachidonate 12-Lipoxygenase, 12S Type | 43 |
| CCL18 | C-C Motif Chemokine Ligand 18 | 36 |
| ZNF335 | Zinc Finger Protein 335 | 37 |
| DAB2 | DAB Adaptor Protein 2 | 43 |
| HYAL1 | Hyaluronidase 1 | 45 |
| ZNHIT3 | Zinc Finger HIT-Type Containing 3 | 36 |
| SERPINA7 | Serpin Family A Member 7 | 39 |
| PRDX1 | Peroxiredoxin 1 | 49 |
| ARHGEF2 | Rho/Rac Guanine Nucleotide Exchange Factor 2 | 45 |
| NSUN6 | NOP2/Sun RNA Methyltransferase 6 | 36 |
| NISCH | Nischarin | 41 |
| PRMT7 | Protein Arginine Methyltransferase 7 | 44 |
| CCL21 | C-C Motif Chemokine Ligand 21 | 43 |
| KDSR | 3-Ketodihydrosphingosine Reductase | 42 |
| FGF21 | Fibroblast Growth Factor 21 | 40 |
| MIR100 | MicroRNA 100 | 21 |
| TCF12 | Transcription Factor 12 | 47 |
| CEP85L | Centrosomal Protein 85 Like | 34 |
| NADSYN1 | NAD Synthetase 1 | 40 |
| IL23A | Interleukin 23 Subunit Alpha | 39 |
| YAP1 | Yes1 Associated Transcriptional Regulator | 47 |
| ROBO2 | Roundabout Guidance Receptor 2 | 42 |
| ERN1 | Endoplasmic Reticulum To Nucleus Signaling 1 | 45 |
| FOLR1 | Folate Receptor Alpha | 47 |
| CYLD | CYLD Lysine 63 Deubiquitinase | 48 |
| LYZ | Lysozyme | 47 |
| SMARCA1 | SWI/SNF Related, Matrix Associated, Actin Dependent Regulator Of Chromatin, Subfamily A, Member 1 | 39 |
| DLG4 | Discs Large MAGUK Scaffold Protein 4 | 47 |
| RHOH | Ras Homolog Family Member H | 43 |
| MIR125B1 | MicroRNA 125b-1 | 21 |
| SOAT2 | Sterol O-Acyltransferase 2 | 44 |
| IGF2BP2 | Insulin Like Growth Factor 2 MRNA Binding Protein 2 | 44 |
| HAPLN1 | Hyaluronan And Proteoglycan Link Protein 1 | 42 |
| HLA-DRA | Major Histocompatibility Complex, Class II, DR Alpha | 47 |
| TUBA4A | Tubulin Alpha 4a | 45 |
| FUCA2 | Alpha-L-Fucosidase 2 | 40 |
| APOD | Apolipoprotein D | 43 |
| APOA1-AS | APOA1 Antisense RNA | 12 |
| CDK1 | Cyclin Dependent Kinase 1 | 45 |
| DCPS | Decapping Enzyme, Scavenger | 43 |
| SLC25A5 | Solute Carrier Family 25 Member 5 | 44 |
| GLRB | Glycine Receptor Beta | 48 |
| RNF213-AS1 | RNF213 Antisense RNA 1 | 11 |
| ADCY1 | Adenylate Cyclase 1 | 48 |
| FBXO7 | F-Box Protein 7 | 41 |
| MIR106A | MicroRNA 106a | 18 |
| lnc-KDM5D-4 |  | 4 |
| RAB5A | RAB5A, Member RAS Oncogene Family | 45 |
| MCU | Mitochondrial Calcium Uniporter | 36 |
| PTGIR | Prostaglandin I2 Receptor | 47 |
| OGA | O-GlcNAcase | 32 |
| KCNT2 | Potassium Sodium-Activated Channel Subfamily T Member 2 | 39 |
| FST | Follistatin | 46 |
| STAG1 | Stromal Antigen 1 | 42 |
| DYM | Dymeclin | 39 |
| RUNX1T1 | RUNX1 Partner Transcriptional Co-Repressor 1 | 39 |
| HMGA1 | High Mobility Group AT-Hook 1 | 46 |
| SRP72 | Signal Recognition Particle 72 | 40 |
| FAM230H | Family With Sequence Similarity 230 Member H | 8 |
| ANXA1 | Annexin A1 | 49 |
| GHRH | Growth Hormone Releasing Hormone | 40 |
| ANTXR1 | ANTXR Cell Adhesion Molecule 1 | 46 |
| DAPK1 | Death Associated Protein Kinase 1 | 48 |
| PNMT | Phenylethanolamine N-Methyltransferase | 44 |
| SLC4A5 | Solute Carrier Family 4 Member 5 | 41 |
| RPS15A | Ribosomal Protein S15a | 41 |
| TSLP | Thymic Stromal Lymphopoietin | 38 |
| C5AR1 | Complement C5a Receptor 1 | 43 |
| CDK2 | Cyclin Dependent Kinase 2 | 52 |
| MICOS13 | Mitochondrial Contact Site And Cristae Organizing System Subunit 13 | 27 |
| NFKBIL1 | NFKB Inhibitor Like 1 | 37 |
| DDAH1 | Dimethylarginine Dimethylaminohydrolase 1 | 43 |
| TRNT1 | TRNA Nucleotidyl Transferase 1 | 41 |
| AQP5 | Aquaporin 5 | 45 |
| BID | BH3 Interacting Domain Death Agonist | 45 |
| KCND2 | Potassium Voltage-Gated Channel Subfamily D Member 2 | 43 |
| CCR4 | C-C Motif Chemokine Receptor 4 | 45 |
| ATP2B1 | ATPase Plasma Membrane Ca2+ Transporting 1 | 44 |
| SLC20A1 | Solute Carrier Family 20 Member 1 | 44 |
| RRAGC | Ras Related GTP Binding C | 41 |
| ADCY8 | Adenylate Cyclase 8 | 44 |
| ANKRD26 | Ankyrin Repeat Domain 26 | 39 |
| UGCG | UDP-Glucose Ceramide Glucosyltransferase | 43 |
| GABRB1 | Gamma-Aminobutyric Acid Type A Receptor Subunit Beta1 | 44 |
| CHD3 | Chromodomain Helicase DNA Binding Protein 3 | 41 |
| FOXP2 | Forkhead Box P2 | 43 |
| RAD50 | RAD50 Double Strand Break Repair Protein | 49 |
| NTN1 | Netrin 1 | 44 |
| IGHM | Immunoglobulin Heavy Constant Mu | 31 |
| OXA1L | OXA1L Mitochondrial Inner Membrane Protein | 39 |
| TMEM126A | Transmembrane Protein 126A | 39 |
| HDLCQ1 | High Density Lipoprotein Cholesterol Level QTL 1 | 3 |
| ITGA9 | Integrin Subunit Alpha 9 | 43 |
| LOC113939944 | Sharpr-MPRA Regulatory Region 9539 | 1 |
| ANXA2 | Annexin A2 | 48 |
| CRLF2 | Cytokine Receptor Like Factor 2 | 39 |
| ZPR1 | ZPR1 Zinc Finger | 35 |
| ROS1 | ROS Proto-Oncogene 1, Receptor Tyrosine Kinase | 45 |
| HEYL | Hes Related Family BHLH Transcription Factor With YRPW Motif Like | 39 |
| RAN | RAN, Member RAS Oncogene Family | 45 |
| NAB2 | NGFI-A Binding Protein 2 | 40 |
| MCAM | Melanoma Cell Adhesion Molecule | 40 |
| KCNN3 | Potassium Calcium-Activated Channel Subfamily N Member 3 | 44 |
| MIR199B | MicroRNA 199b | 19 |
| TRPM1 | Transient Receptor Potential Cation Channel Subfamily M Member 1 | 43 |
| CYP2C8 | Cytochrome P450 Family 2 Subfamily C Member 8 | 48 |
| APEX1 | Apurinic/Apyrimidinic Endodeoxyribonuclease 1 | 45 |
| NOD1 | Nucleotide Binding Oligomerization Domain Containing 1 | 44 |
| SLCO1B1 | Solute Carrier Organic Anion Transporter Family Member 1B1 | 48 |
| KAT2B | Lysine Acetyltransferase 2B | 48 |
| GRHL2 | Grainyhead Like Transcription Factor 2 | 40 |
| TAPT1 | Transmembrane Anterior Posterior Transformation 1 | 36 |
| CABIN1 | Calcineurin Binding Protein 1 | 41 |
| PTK2 | Protein Tyrosine Kinase 2 | 47 |
| MIR26A1 | MicroRNA 26a-1 | 20 |
| CXCL16 | C-X-C Motif Chemokine Ligand 16 | 40 |
| CRYGC | Crystallin Gamma C | 40 |
| FCN2 | Ficolin 2 | 40 |
| MRPL3 | Mitochondrial Ribosomal Protein L3 | 41 |
| RGS2 | Regulator Of G Protein Signaling 2 | 43 |
| FMN2 | Formin 2 | 40 |
| PPIA | Peptidylprolyl Isomerase A | 47 |
| HHEX | Hematopoietically Expressed Homeobox | 42 |
| HDAC2 | Histone Deacetylase 2 | 51 |
| CLOCK | Clock Circadian Regulator | 43 |
| LIPF | Lipase F, Gastric Type | 41 |
| CLDN4 | Claudin 4 | 41 |
| HBS1L | HBS1 Like Translational GTPase | 39 |
| NONO | Non-POU Domain Containing Octamer Binding | 44 |
| RYR3 | Ryanodine Receptor 3 | 41 |
| CLCN6 | Chloride Voltage-Gated Channel 6 | 41 |
| HPSE | Heparanase | 44 |
| SIRT6 | Sirtuin 6 | 45 |
| TACR1 | Tachykinin Receptor 1 | 45 |
| SMTN | Smoothelin | 39 |
| TRPV2 | Transient Receptor Potential Cation Channel Subfamily V Member 2 | 41 |
| TRH | Thyrotropin Releasing Hormone | 42 |
| CRHR1 | Corticotropin Releasing Hormone Receptor 1 | 45 |
| TREM1 | Triggering Receptor Expressed On Myeloid Cells 1 | 41 |
| SRR | Serine Racemase | 43 |
| HIP1 | Huntingtin Interacting Protein 1 | 41 |
| PRKAB1 | Protein Kinase AMP-Activated Non-Catalytic Subunit Beta 1 | 47 |
| CAMK2A | Calcium/Calmodulin Dependent Protein Kinase II Alpha | 49 |
| FDPS | Farnesyl Diphosphate Synthase | 45 |
| FKBP6 | FKBP Prolyl Isomerase 6 | 39 |
| KDM6B | Lysine Demethylase 6B | 42 |
| NPB | Neuropeptide B | 31 |
| PARS2 | Prolyl-TRNA Synthetase 2, Mitochondrial | 41 |
| CXCL11 | C-X-C Motif Chemokine Ligand 11 | 40 |
| MYOG | Myogenin | 39 |
| LIMS1 | LIM Zinc Finger Domain Containing 1 | 40 |
| DUOX1 | Dual Oxidase 1 | 41 |
| AP2B1 | Adaptor Related Protein Complex 2 Subunit Beta 1 | 42 |
| CNTN1 | Contactin 1 | 44 |
| KDM5B | Lysine Demethylase 5B | 43 |
| SLC22A2 | Solute Carrier Family 22 Member 2 | 44 |
| IL5RA | Interleukin 5 Receptor Subunit Alpha | 46 |
| VPS11 | VPS11 Core Subunit Of CORVET And HOPS Complexes | 41 |
| ADPRH | ADP-Ribosylarginine Hydrolase | 37 |
| MAPRE2 | Microtubule Associated Protein RP/EB Family Member 2 | 41 |
| FEN1 | Flap Structure-Specific Endonuclease 1 | 46 |
| GAP43 | Growth Associated Protein 43 | 43 |
| HLA-DQA2 | Major Histocompatibility Complex, Class II, DQ Alpha 2 | 37 |
| ABCG4 | ATP Binding Cassette Subfamily G Member 4 | 39 |
| DLC1 | DLC1 Rho GTPase Activating Protein | 44 |
| DUSP6 | Dual Specificity Phosphatase 6 | 48 |
| KAT2A | Lysine Acetyltransferase 2A | 47 |
| UTRN | Utrophin | 40 |
| SMDT1 | Single-Pass Membrane Protein With Aspartate Rich Tail 1 | 32 |
| BCL2L11 | BCL2 Like 11 | 45 |
| SLC2A3 | Solute Carrier Family 2 Member 3 | 48 |
| FABP1 | Fatty Acid Binding Protein 1 | 43 |
| MBNL1 | Muscleblind Like Splicing Regulator 1 | 39 |
| CXCL5 | C-X-C Motif Chemokine Ligand 5 | 40 |
| TYMS | Thymidylate Synthetase | 47 |
| FBXO38 | F-Box Protein 38 | 37 |
| TRB | T Cell Receptor Beta Locus | 14 |
| FKBP5 | FKBP Prolyl Isomerase 5 | 45 |
| GAS6 | Growth Arrest Specific 6 | 44 |
| ACTA2-AS1 | ACTA2 Antisense RNA 1 | 15 |
| SMARCA5 | SWI/SNF Related, Matrix Associated, Actin Dependent Regulator Of Chromatin, Subfamily A, Member 5 | 40 |
| MIR149 | MicroRNA 149 | 21 |
| SLC5A1 | Solute Carrier Family 5 Member 1 | 49 |
| GPC1 | Glypican 1 | 43 |
| SAA4 | Serum Amyloid A4, Constitutive | 39 |
| RPS3A | Ribosomal Protein S3A | 41 |
| SOX6 | SRY-Box Transcription Factor 6 | 42 |
| CACNG5 | Calcium Voltage-Gated Channel Auxiliary Subunit Gamma 5 | 40 |
| PTPN3 | Protein Tyrosine Phosphatase Non-Receptor Type 3 | 44 |
| NEAT1 | Nuclear Paraspeckle Assembly Transcript 1 | 23 |
| INO80 | INO80 Complex ATPase Subunit | 36 |
| CAMP | Cathelicidin Antimicrobial Peptide | 41 |
| SETDB1 | SET Domain Bifurcated Histone Lysine Methyltransferase 1 | 41 |
| CXCL13 | C-X-C Motif Chemokine Ligand 13 | 41 |
| CCDC92 | Coiled-Coil Domain Containing 92 | 35 |
| GSS | Glutathione Synthetase | 47 |
| MARK3 | Microtubule Affinity Regulating Kinase 3 | 48 |
| OGG1 | 8-Oxoguanine DNA Glycosylase | 47 |
| SNAI1 | Snail Family Transcriptional Repressor 1 | 44 |
| GPD2 | Glycerol-3-Phosphate Dehydrogenase 2 | 46 |
| MIR590 | MicroRNA 590 | 18 |
| HDAC1 | Histone Deacetylase 1 | 49 |
| BAG6 | BAG Cochaperone 6 | 35 |
| LRP4 | LDL Receptor Related Protein 4 | 43 |
| MRPS34 | Mitochondrial Ribosomal Protein S34 | 37 |
| MYBPHL | Myosin Binding Protein H Like | 31 |
| MKI67 | Marker Of Proliferation Ki-67 | 44 |
| FGF5 | Fibroblast Growth Factor 5 | 45 |
| SH3BP2 | SH3 Domain Binding Protein 2 | 40 |
| HIC1 | HIC ZBTB Transcriptional Repressor 1 | 40 |
| PRDM8 | PR/SET Domain 8 | 36 |
| DNAH10 | Dynein Axonemal Heavy Chain 10 | 35 |
| SRD5A1 | Steroid 5 Alpha-Reductase 1 | 44 |
| GGT2 | Gamma-Glutamyltransferase 2 | 31 |
| LOC106029312 | Williams-Beuren Syndrome Medial Block B Recombination Region | 1 |
| MIR16-1 | MicroRNA 16-1 | 21 |
| ANGPTL4 | Angiopoietin Like 4 | 45 |
| CLDN3 | Claudin 3 | 40 |
| ATRIP | ATR Interacting Protein | 41 |
| MACROD1 | Mono-ADP Ribosylhydrolase 1 | 35 |
| MIR92A1 | MicroRNA 92a-1 | 19 |
| TNNC2 | Troponin C2, Fast Skeletal Type | 38 |
| ESRRB | Estrogen Related Receptor Beta | 50 |
| FGF7 | Fibroblast Growth Factor 7 | 41 |
| TRIM2 | Tripartite Motif Containing 2 | 40 |
| ARHGEF9 | Cdc42 Guanine Nucleotide Exchange Factor 9 | 41 |
| WDR4 | WD Repeat Domain 4 | 38 |
| TJP1 | Tight Junction Protein 1 | 43 |
| SERPINB1 | Serpin Family B Member 1 | 40 |
| NPS | Neuropeptide S | 32 |
| MIRLET7C | MicroRNA Let-7c | 21 |
| SRRT | Serrate, RNA Effector Molecule | 37 |
| NUDT6 | Nudix Hydrolase 6 | 37 |
| UBAP1 | Ubiquitin Associated Protein 1 | 38 |
| NT5C2 | 5'-Nucleotidase, Cytosolic II | 47 |
| TNFRSF10A | TNF Receptor Superfamily Member 10a | 45 |
| ST3GAL4 | ST3 Beta-Galactoside Alpha-2,3-Sialyltransferase 4 | 41 |
| RAB8A | RAB8A, Member RAS Oncogene Family | 40 |
| FMOD | Fibromodulin | 40 |
| CACNA1E | Calcium Voltage-Gated Channel Subunit Alpha1 E | 46 |
| HSPB2 | Heat Shock Protein Family B (Small) Member 2 | 40 |
| KLRK1 | Killer Cell Lectin Like Receptor K1 | 40 |
| MMAB | Metabolism Of Cobalamin Associated B | 45 |
| NSUN5 | NOP2/Sun RNA Methyltransferase 5 | 38 |
| B4GALT1 | Beta-1,4-Galactosyltransferase 1 | 44 |
| PKM | Pyruvate Kinase M1/2 | 45 |
| SMS | Spermine Synthase | 44 |
| PI4KA | Phosphatidylinositol 4-Kinase Alpha | 48 |
| FOLH1 | Folate Hydrolase 1 | 46 |
| CACNB3 | Calcium Voltage-Gated Channel Auxiliary Subunit Beta 3 | 42 |
| MLPH | Melanophilin | 43 |
| SIRT2 | Sirtuin 2 | 48 |
| TAF1 | TATA-Box Binding Protein Associated Factor 1 | 45 |
| DDR2 | Discoidin Domain Receptor Tyrosine Kinase 2 | 51 |
| OPHN1 | Oligophrenin 1 | 42 |
| HAND2-AS1 | HAND2 Antisense RNA 1 | 20 |
| ARHGEF10 | Rho Guanine Nucleotide Exchange Factor 10 | 43 |
| SMAD1 | SMAD Family Member 1 | 44 |
| OPCML | Opioid Binding Protein/Cell Adhesion Molecule Like | 41 |
| PTPN2 | Protein Tyrosine Phosphatase Non-Receptor Type 2 | 46 |
| HSP90B1 | Heat Shock Protein 90 Beta Family Member 1 | 45 |
| EPHA3 | EPH Receptor A3 | 45 |
| BCL7B | BAF Chromatin Remodeling Complex Subunit BCL7B | 36 |
| PYY | Peptide YY | 42 |
| MS4A2 | Membrane Spanning 4-Domains A2 | 41 |
| GLRX | Glutaredoxin | 44 |
| ADAMTS2 | ADAM Metallopeptidase With Thrombospondin Type 1 Motif 2 | 42 |
| PSRC1 | Proline And Serine Rich Coiled-Coil 1 | 36 |
| TRIO | Trio Rho Guanine Nucleotide Exchange Factor | 45 |
| TXNDC15 | Thioredoxin Domain Containing 15 | 37 |
| NOP2 | NOP2 Nucleolar Protein | 36 |
| RGS9BP | Regulator Of G Protein Signaling 9 Binding Protein | 35 |
| TAF6 | TATA-Box Binding Protein Associated Factor 6 | 40 |
| KLF6 | Kruppel Like Factor 6 | 44 |
| ICMT | Isoprenylcysteine Carboxyl Methyltransferase | 41 |
| PITPNA | Phosphatidylinositol Transfer Protein Alpha | 40 |
| TRAF1 | TNF Receptor Associated Factor 1 | 43 |
| NAXD | NAD(P)HX Dehydratase | 31 |
| KLK1 | Kallikrein 1 | 45 |
| CACNG1 | Calcium Voltage-Gated Channel Auxiliary Subunit Gamma 1 | 41 |
| SIK3 | SIK Family Kinase 3 | 41 |
| MIR206 | MicroRNA 206 | 20 |
| CTSS | Cathepsin S | 45 |
| ADIRF | Adipogenesis Regulatory Factor | 32 |
| MCM6 | Minichromosome Maintenance Complex Component 6 | 43 |
| POC1B | POC1 Centriolar Protein B | 37 |
| FOXD4 | Forkhead Box D4 | 33 |
| CRYGD | Crystallin Gamma D | 40 |
| ACACA | Acetyl-CoA Carboxylase Alpha | 48 |
| CAVIN4 | Caveolae Associated Protein 4 | 26 |
| ADAM8 | ADAM Metallopeptidase Domain 8 | 42 |
| PLCB1 | Phospholipase C Beta 1 | 48 |
| KANK2 | KN Motif And Ankyrin Repeat Domains 2 | 39 |
| LCK | LCK Proto-Oncogene, Src Family Tyrosine Kinase | 52 |
| TTF2 | Transcription Termination Factor 2 | 39 |
| HYOU1 | Hypoxia Up-Regulated 1 | 43 |
| CSK | C-Terminal Src Kinase | 48 |
| NR1I2 | Nuclear Receptor Subfamily 1 Group I Member 2 | 45 |
| PDPN | Podoplanin | 40 |
| PEMT | Phosphatidylethanolamine N-Methyltransferase | 41 |
| EDC3 | Enhancer Of MRNA Decapping 3 | 41 |
| ALG14 | ALG14 UDP-N-Acetylglucosaminyltransferase Subunit | 41 |
| BARD1 | BRCA1 Associated RING Domain 1 | 46 |
| CAP2 | Cyclase Associated Actin Cytoskeleton Regulatory Protein 2 | 40 |
| EIF4E | Eukaryotic Translation Initiation Factor 4E | 50 |
| PDHB | Pyruvate Dehydrogenase E1 Subunit Beta | 45 |
| ELAVL1 | ELAV Like RNA Binding Protein 1 | 41 |
| MIR27B | MicroRNA 27b | 21 |
| MIR20B | MicroRNA 20b | 15 |
| ADCYAP1 | Adenylate Cyclase Activating Polypeptide 1 | 41 |
| FGG | Fibrinogen Gamma Chain | 48 |
| TBC1D4 | TBC1 Domain Family Member 4 | 44 |
| SLC29A1 | Solute Carrier Family 29 Member 1 (Augustine Blood Group) | 48 |
| NEDD4 | NEDD4 E3 Ubiquitin Protein Ligase | 45 |
| CCL19 | C-C Motif Chemokine Ligand 19 | 41 |
| HHIP | Hedgehog Interacting Protein | 41 |
| LYVE1 | Lymphatic Vessel Endothelial Hyaluronan Receptor 1 | 41 |
| FCGR1A | Fc Fragment Of IgG Receptor Ia | 41 |
| MAPK9 | Mitogen-Activated Protein Kinase 9 | 48 |
| DRP2 | Dystrophin Related Protein 2 | 36 |
| AOC1 | Amine Oxidase Copper Containing 1 | 40 |
| IMMT | Inner Membrane Mitochondrial Protein | 39 |
| GNAL | G Protein Subunit Alpha L | 45 |
| RPS6 | Ribosomal Protein S6 | 44 |
| LNPEP | Leucyl And Cystinyl Aminopeptidase | 45 |
| PRKCG | Protein Kinase C Gamma | 51 |
| SLN | Sarcolipin | 32 |
| EIF2S1 | Eukaryotic Translation Initiation Factor 2 Subunit Alpha | 44 |
| CDKAL1 | CDK5 Regulatory Subunit Associated Protein 1 Like 1 | 39 |
| PXK | PX Domain Containing Serine/Threonine Kinase Like | 38 |
| THSD4 | Thrombospondin Type 1 Domain Containing 4 | 36 |
| FABP12 | Fatty Acid Binding Protein 12 | 31 |
| CYSLTR1 | Cysteinyl Leukotriene Receptor 1 | 43 |
| ACAT1 | Acetyl-CoA Acetyltransferase 1 | 49 |
| GPC5 | Glypican 5 | 40 |
| ARMS2 | Age-Related Maculopathy Susceptibility 2 | 30 |
| MIR148B | MicroRNA 148b | 20 |
| CCND3 | Cyclin D3 | 47 |
| SLC1A4 | Solute Carrier Family 1 Member 4 | 44 |
| CCN1 | Cellular Communication Network Factor 1 | 31 |
| TPR | Translocated Promoter Region, Nuclear Basket Protein | 42 |
| GUCY1B1 | Guanylate Cyclase 1 Soluble Subunit Beta 1 | 33 |
| HPS6 | HPS6 Biogenesis Of Lysosomal Organelles Complex 2 Subunit 3 | 39 |
| KLHDC8B | Kelch Domain Containing 8B | 38 |
| ANPEP | Alanyl Aminopeptidase, Membrane | 48 |
| MIR139 | MicroRNA 139 | 19 |
| ESRRA | Estrogen Related Receptor Alpha | 47 |
| CD1C | CD1c Molecule | 39 |
| GRM5 | Glutamate Metabotropic Receptor 5 | 47 |
| FBLN1 | Fibulin 1 | 43 |
| CNR2 | Cannabinoid Receptor 2 | 45 |
| DNAH6 | Dynein Axonemal Heavy Chain 6 | 32 |
| FDFT1 | Farnesyl-Diphosphate Farnesyltransferase 1 | 44 |
| DGAT2 | Diacylglycerol O-Acyltransferase 2 | 43 |
| ADAMTS6 | ADAM Metallopeptidase With Thrombospondin Type 1 Motif 6 | 38 |
| MYO15A | Myosin XVA | 36 |
| PSMD5 | Proteasome 26S Subunit, Non-ATPase 5 | 38 |
| PLA2G5 | Phospholipase A2 Group V | 44 |
| RAPGEF2 | Rap Guanine Nucleotide Exchange Factor 2 | 41 |
| LGALS1 | Galectin 1 | 43 |
| CFD | Complement Factor D | 44 |
| DPP9 | Dipeptidyl Peptidase 9 | 40 |
| PCK2 | Phosphoenolpyruvate Carboxykinase 2, Mitochondrial | 46 |
| AASS | Aminoadipate-Semialdehyde Synthase | 44 |
| REG1A | Regenerating Family Member 1 Alpha | 41 |
| VPS37D | VPS37D Subunit Of ESCRT-I | 32 |
| PTK2B | Protein Tyrosine Kinase 2 Beta | 49 |
| PTGER4 | Prostaglandin E Receptor 4 | 46 |
| MIR181A2 | MicroRNA 181a-2 | 20 |
| FCRL3 | Fc Receptor Like 3 | 36 |
| SEC24D | SEC24 Homolog D, COPII Coat Complex Component | 44 |
| ASPN | Asporin | 39 |
| SLC1A1 | Solute Carrier Family 1 Member 1 | 47 |
| CD63 | CD63 Molecule | 41 |
| MFGE8 | Milk Fat Globule EGF And Factor V/VIII Domain Containing | 44 |
| F2RL1 | F2R Like Trypsin Receptor 1 | 45 |
| ZBTB17 | Zinc Finger And BTB Domain Containing 17 | 41 |
| GATAD2B | GATA Zinc Finger Domain Containing 2B | 42 |
| CCL7 | C-C Motif Chemokine Ligand 7 | 41 |
| KIRREL3 | Kirre Like Nephrin Family Adhesion Molecule 3 | 41 |
| SEC23A | Sec23 Homolog A, COPII Coat Complex Component | 43 |
| CES1 | Carboxylesterase 1 | 46 |
| H3-3B | H3.3 Histone B | 33 |
| GTF2IRD2 | GTF2I Repeat Domain Containing 2 | 32 |
| DUSP1 | Dual Specificity Phosphatase 1 | 47 |
| GMPR | Guanosine Monophosphate Reductase | 43 |
| ARHGAP26 | Rho GTPase Activating Protein 26 | 44 |
| WDR81 | WD Repeat Domain 81 | 36 |
| MIR32 | MicroRNA 32 | 19 |
| YWHAZ | Tyrosine 3-Monooxygenase/Tryptophan 5-Monooxygenase Activation Protein Zeta | 48 |
| NOX5 | NADPH Oxidase 5 | 37 |
| MYO9B | Myosin IXB | 44 |
| STK24 | Serine/Threonine Kinase 24 | 44 |
| PIP5K1C | Phosphatidylinositol-4-Phosphate 5-Kinase Type 1 Gamma | 48 |
| PLK4 | Polo Like Kinase 4 | 47 |
| HAS2 | Hyaluronan Synthase 2 | 40 |
| HRH1 | Histamine Receptor H1 | 45 |
| SIRT5 | Sirtuin 5 | 45 |
| PI3 | Peptidase Inhibitor 3 | 39 |
| DVL2 | Dishevelled Segment Polarity Protein 2 | 45 |
| MIR532 | MicroRNA 532 | 16 |
| MIR181C | MicroRNA 181c | 20 |
| CD70 | CD70 Molecule | 43 |
| APOC4 | Apolipoprotein C4 | 36 |
| CLEC16A | C-Type Lectin Domain Containing 16A | 37 |
| LIN9 | Lin-9 DREAM MuvB Core Complex Component | 37 |
| MADD | MAP Kinase Activating Death Domain | 40 |
| CNN1 | Calponin 1 | 41 |
| MYB | MYB Proto-Oncogene, Transcription Factor | 50 |
| SLC10A1 | Solute Carrier Family 10 Member 1 | 43 |
| AAGAB | Alpha And Gamma Adaptin Binding Protein | 40 |
| TNS1 | Tensin 1 | 39 |
| POLR2A | RNA Polymerase II Subunit A | 44 |
| DBN1 | Drebrin 1 | 39 |
| MIR375 | MicroRNA 375 | 19 |
| SPTBN4 | Spectrin Beta, Non-Erythrocytic 4 | 39 |
| CLIP1 | CAP-Gly Domain Containing Linker Protein 1 | 44 |
| POLB | DNA Polymerase Beta | 47 |
| PBRM1 | Polybromo 1 | 44 |
| TRPC4AP | Transient Receptor Potential Cation Channel Subfamily C Member 4 Associated Protein | 37 |
| LPIN1 | Lipin 1 | 48 |
| APEX2 | Apurinic/Apyrimidinic Endodeoxyribonuclease 2 | 37 |
| SIRT4 | Sirtuin 4 | 40 |
| EIF4H | Eukaryotic Translation Initiation Factor 4H | 41 |
| ATP6V1G2-DDX39B | ATP6V1G2-DDX39B Readthrough (NMD Candidate) | 18 |
| MTNR1B | Melatonin Receptor 1B | 46 |
| EGLN3 | Egl-9 Family Hypoxia Inducible Factor 3 | 46 |
| PRG2 | Proteoglycan 2, Pro Eosinophil Major Basic Protein | 40 |
| FZD9 | Frizzled Class Receptor 9 | 44 |
| BMPER | BMP Binding Endothelial Regulator | 39 |
| MNT | MAX Network Transcriptional Repressor | 36 |
| KCNAB1 | Potassium Voltage-Gated Channel Subfamily A Member Regulatory Beta Subunit 1 | 41 |
| NR1I3 | Nuclear Receptor Subfamily 1 Group I Member 3 | 44 |
| PNPLA5 | Patatin Like Phospholipase Domain Containing 5 | 34 |
| PDE4B | Phosphodiesterase 4B | 44 |
| EMILIN1 | Elastin Microfibril Interfacer 1 | 38 |
| DOCK7 | Dedicator Of Cytokinesis 7 | 41 |
| CCNA2 | Cyclin A2 | 44 |
| LEXM | Lymphocyte Expansion Molecule | 24 |
| HBE1 | Hemoglobin Subunit Epsilon 1 | 38 |
| CSN1S1 | Casein Alpha S1 | 34 |
| PLXNA2 | Plexin A2 | 42 |
| RPL7 | Ribosomal Protein L7 | 41 |
| FOXL1 | Forkhead Box L1 | 37 |
| ADD2 | Adducin 2 | 40 |
| KEAP1 | Kelch Like ECH Associated Protein 1 | 48 |
| LOC110973015 | NOS3 5' Regulatory Region | 1 |
| HOXC-AS1 | HOXC Cluster Antisense RNA 1 | 16 |
| ST8SIA4 | ST8 Alpha-N-Acetyl-Neuraminide Alpha-2,8-Sialyltransferase 4 | 40 |
| OXLD1 | Oxidoreductase Like Domain Containing 1 | 28 |
| POU2F3 | POU Class 2 Homeobox 3 | 38 |
| PER1 | Period Circadian Regulator 1 | 41 |
| ENPEP | Glutamyl Aminopeptidase | 44 |
| FAM20A | FAM20A Golgi Associated Secretory Pathway Pseudokinase | 39 |
| ADM2 | Adrenomedullin 2 | 33 |
| HYPLIP2 | Hyperlipidemia, Combined, 2 | 2 |
| CACNG7 | Calcium Voltage-Gated Channel Auxiliary Subunit Gamma 7 | 37 |
| RASD1 | Ras Related Dexamethasone Induced 1 | 39 |
| CYBC1 | Cytochrome B-245 Chaperone 1 | 25 |
| CYBRD1 | Cytochrome B Reductase 1 | 40 |
| CECR7 | Cat Eye Syndrome Chromosome Region, Candidate 7 | 17 |
| TRAF3 | TNF Receptor Associated Factor 3 | 47 |
| RPLP0 | Ribosomal Protein Lateral Stalk Subunit P0 | 41 |
| CLDN5 | Claudin 5 | 41 |
| RXRG | Retinoid X Receptor Gamma | 41 |
| UNC5C | Unc-5 Netrin Receptor C | 40 |
| MTMR3 | Myotubularin Related Protein 3 | 42 |
| NFIB | Nuclear Factor I B | 43 |
| SHMT1 | Serine Hydroxymethyltransferase 1 | 45 |
| FASN | Fatty Acid Synthase | 49 |
| TBXAS1 | Thromboxane A Synthase 1 | 50 |
| CAD | Carbamoyl-Phosphate Synthetase 2, Aspartate Transcarbamylase, And Dihydroorotase | 50 |
| RAMP2 | Receptor Activity Modifying Protein 2 | 41 |
| UBE2D1 | Ubiquitin Conjugating Enzyme E2 D1 | 45 |
| IL19 | Interleukin 19 | 40 |
| PRELP | Proline And Arginine Rich End Leucine Rich Repeat Protein | 40 |
| VPS13C | Vacuolar Protein Sorting 13 Homolog C | 35 |
| VASP | Vasodilator Stimulated Phosphoprotein | 43 |
| OGN | Osteoglycin | 39 |
| MFAP4 | Microfibril Associated Protein 4 | 40 |
| CACNG8 | Calcium Voltage-Gated Channel Auxiliary Subunit Gamma 8 | 37 |
| CHGB | Chromogranin B | 39 |
| SH3PXD2A | SH3 And PX Domains 2A | 37 |
| DYNLL1 | Dynein Light Chain LC8-Type 1 | 42 |
| PLCB4 | Phospholipase C Beta 4 | 47 |
| NOMO3 | NODAL Modulator 3 | 31 |
| CA2 | Carbonic Anhydrase 2 | 51 |
| IQGAP1 | IQ Motif Containing GTPase Activating Protein 1 | 43 |
| PRDM1 | PR/SET Domain 1 | 45 |
| SCRN3 | Secernin 3 | 33 |
| MSN | Moesin | 47 |
| GOT2 | Glutamic-Oxaloacetic Transaminase 2 | 45 |
| PIK3CB | Phosphatidylinositol-4,5-Bisphosphate 3-Kinase Catalytic Subunit Beta | 47 |
| IL36RN | Interleukin 36 Receptor Antagonist | 41 |
| P2RX4 | Purinergic Receptor P2X 4 | 43 |
| CYP26B1 | Cytochrome P450 Family 26 Subfamily B Member 1 | 45 |
| TRIM63 | Tripartite Motif Containing 63 | 40 |
| EML1 | EMAP Like 1 | 40 |
| SBF2-AS1 | SBF2 Antisense RNA 1 | 15 |
| S100A4 | S100 Calcium Binding Protein A4 | 44 |
| SUMO4 | Small Ubiquitin Like Modifier 4 | 38 |
| ATP5IF1 | ATP Synthase Inhibitory Factor Subunit 1 | 30 |
| PRKCZ | Protein Kinase C Zeta | 48 |
| TMEM116 | Transmembrane Protein 116 | 27 |
| KLK15 | Kallikrein Related Peptidase 15 | 41 |
| HPS5 | HPS5 Biogenesis Of Lysosomal Organelles Complex 2 Subunit 2 | 39 |
| SNRPA | Small Nuclear Ribonucleoprotein Polypeptide A | 40 |
| ARHGEF1 | Rho Guanine Nucleotide Exchange Factor 1 | 44 |
| RPS2 | Ribosomal Protein S2 | 43 |
| CATSPER2 | Cation Channel Sperm Associated 2 | 42 |
| WNT9B | Wnt Family Member 9B | 41 |
| P2RX3 | Purinergic Receptor P2X 3 | 41 |
| ID4 | Inhibitor Of DNA Binding 4, HLH Protein | 39 |
| STK32B | Serine/Threonine Kinase 32B | 33 |
| S1PR1 | Sphingosine-1-Phosphate Receptor 1 | 44 |
| NR4A1 | Nuclear Receptor Subfamily 4 Group A Member 1 | 46 |
| LAMA5 | Laminin Subunit Alpha 5 | 42 |
| CCDC174 | Coiled-Coil Domain Containing 174 | 32 |
| CNTLN | Centlein | 36 |
| PRDX5 | Peroxiredoxin 5 | 46 |
| ADAMTS20 | ADAM Metallopeptidase With Thrombospondin Type 1 Motif 20 | 36 |
| CTNS | Cystinosin, Lysosomal Cystine Transporter | 44 |
| TAF1A | TATA-Box Binding Protein Associated Factor, RNA Polymerase I Subunit A | 36 |
| NFIA-AS1 | NFIA Antisense RNA 1 | 14 |
| NOMO2 | NODAL Modulator 2 | 32 |
| CCL22 | C-C Motif Chemokine Ligand 22 | 38 |
| RCC1L | RCC1 Like | 24 |
| HCN2 | Hyperpolarization Activated Cyclic Nucleotide Gated Potassium And Sodium Channel 2 | 45 |
| TONSL | Tonsoku Like, DNA Repair Protein | 36 |
| MDK | Midkine | 43 |
| CLCN3 | Chloride Voltage-Gated Channel 3 | 41 |
| AKR1A1 | Aldo-Keto Reductase Family 1 Member A1 | 44 |
| CPT1B | Carnitine Palmitoyltransferase 1B | 44 |
| HK2 | Hexokinase 2 | 47 |
| MIR502 | MicroRNA 502 | 16 |
| LIPJ | Lipase Family Member J | 32 |
| P2RY2 | Purinergic Receptor P2Y2 | 45 |
| GIP | Gastric Inhibitory Polypeptide | 39 |
| MLF1 | Myeloid Leukemia Factor 1 | 43 |
| BCL3 | BCL3 Transcription Coactivator | 41 |
| FGR | FGR Proto-Oncogene, Src Family Tyrosine Kinase | 48 |
| RCE1 | Ras Converting CAAX Endopeptidase 1 | 38 |
| NSUN3 | NOP2/Sun RNA Methyltransferase 3 | 36 |
| BLVRA | Biliverdin Reductase A | 45 |
| HERPUD1 | Homocysteine Inducible ER Protein With Ubiquitin Like Domain 1 | 39 |
| PRKCQ | Protein Kinase C Theta | 49 |
| VSTM4 | V-Set And Transmembrane Domain Containing 4 | 32 |
| MIR505 | MicroRNA 505 | 16 |
| NOMO1 | NODAL Modulator 1 | 32 |
| LPAR1 | Lysophosphatidic Acid Receptor 1 | 45 |
| NUP153 | Nucleoporin 153 | 40 |
| LIPM | Lipase Family Member M | 32 |
| MIR1-1 | MicroRNA 1-1 | 19 |
| ARNT | Aryl Hydrocarbon Receptor Nuclear Translocator | 43 |
| ESM1 | Endothelial Cell Specific Molecule 1 | 40 |
| CHRNA5 | Cholinergic Receptor Nicotinic Alpha 5 Subunit | 44 |
| BLOC1S3 | Biogenesis Of Lysosomal Organelles Complex 1 Subunit 3 | 35 |
| SLC5A3 | Solute Carrier Family 5 Member 3 | 43 |
| CCL1 | C-C Motif Chemokine Ligand 1 | 38 |
| RBP5 | Retinol Binding Protein 5 | 36 |
| ULK1 | Unc-51 Like Autophagy Activating Kinase 1 | 45 |
| IRX5 | Iroquois Homeobox 5 | 39 |
| ADAMTS9 | ADAM Metallopeptidase With Thrombospondin Type 1 Motif 9 | 37 |
| CPE | Carboxypeptidase E | 44 |
| PTGER2 | Prostaglandin E Receptor 2 | 48 |
| S1PR3 | Sphingosine-1-Phosphate Receptor 3 | 44 |
| PDE3B | Phosphodiesterase 3B | 43 |
| BTRC | Beta-Transducin Repeat Containing E3 Ubiquitin Protein Ligase | 46 |
| GALNT2 | Polypeptide N-Acetylgalactosaminyltransferase 2 | 45 |
| VEGFD | Vascular Endothelial Growth Factor D | 34 |
| DAB2IP | DAB2 Interacting Protein | 39 |
| CRHR2 | Corticotropin Releasing Hormone Receptor 2 | 42 |
| AREG | Amphiregulin | 43 |
| IL27 | Interleukin 27 | 38 |
| NCOR2 | Nuclear Receptor Corepressor 2 | 43 |
| TNXA | Tenascin XA (Pseudogene) | 23 |
| TIE1 | Tyrosine Kinase With Immunoglobulin Like And EGF Like Domains 1 | 43 |
| RBBP4 | RB Binding Protein 4, Chromatin Remodeling Factor | 43 |
| IRGM | Immunity Related GTPase M | 38 |
| CYP4A11 | Cytochrome P450 Family 4 Subfamily A Member 11 | 43 |
| SUV39H1 | Suppressor Of Variegation 3-9 Homolog 1 | 43 |
| TYRO3 | TYRO3 Protein Tyrosine Kinase | 47 |
| CAPNS1 | Calpain Small Subunit 1 | 41 |
| PLA2G10 | Phospholipase A2 Group X | 44 |
| NAT10 | N-Acetyltransferase 10 | 39 |
| NCOR1 | Nuclear Receptor Corepressor 1 | 43 |
| SS18 | SS18 Subunit Of BAF Chromatin Remodeling Complex | 40 |
| MIR28 | MicroRNA 28 | 19 |
| SGMS2 | Sphingomyelin Synthase 2 | 37 |
| INPP5B | Inositol Polyphosphate-5-Phosphatase B | 41 |
| POLR2B | RNA Polymerase II Subunit B | 42 |
| NME7 | NME/NM23 Family Member 7 | 43 |
| RUNX3 | RUNX Family Transcription Factor 3 | 43 |
| CBX5 | Chromobox 5 | 44 |
| TNFSF13 | TNF Superfamily Member 13 | 44 |
| ATG7 | Autophagy Related 7 | 43 |
| TAB1 | TGF-Beta Activated Kinase 1 (MAP3K7) Binding Protein 1 | 42 |
| RGS9 | Regulator Of G Protein Signaling 9 | 44 |
| NFATC2 | Nuclear Factor Of Activated T Cells 2 | 45 |
| CSMD1 | CUB And Sushi Multiple Domains 1 | 37 |
| P4HA3 | Prolyl 4-Hydroxylase Subunit Alpha 3 | 39 |
| APCS | Amyloid P Component, Serum | 41 |
| TRIM50 | Tripartite Motif Containing 50 | 32 |
| SLC22A1 | Solute Carrier Family 22 Member 1 | 43 |
| MYLK-AS1 | MYLK Antisense RNA 1 | 16 |
| RORA | RAR Related Orphan Receptor A | 48 |
| ZCCHC8 | Zinc Finger CCHC-Type Containing 8 | 35 |
| PSMC6 | Proteasome 26S Subunit, ATPase 6 | 40 |
| KLF2 | Kruppel Like Factor 2 | 40 |
| GALNT17 | Polypeptide N-Acetylgalactosaminyltransferase 17 | 31 |
| MAP2K6 | Mitogen-Activated Protein Kinase Kinase 6 | 47 |
| THSD1 | Thrombospondin Type 1 Domain Containing 1 | 39 |
| UTS2R | Urotensin 2 Receptor | 41 |
| WDR12 | WD Repeat Domain 12 | 38 |
| PLPP3 | Phospholipid Phosphatase 3 | 35 |
| ADORA2B | Adenosine A2b Receptor | 47 |
| RPA1 | Replication Protein A1 | 45 |
| SMG6 | SMG6 Nonsense Mediated MRNA Decay Factor | 40 |
| LAT2 | Linker For Activation Of T Cells Family Member 2 | 39 |
| HSPA1B | Heat Shock Protein Family A (Hsp70) Member 1B | 40 |
| MIR138-1 | MicroRNA 138-1 | 19 |
| LDB1 | LIM Domain Binding 1 | 39 |
| METTL27 | Methyltransferase Like 27 | 25 |
| LAMC1 | Laminin Subunit Gamma 1 | 43 |
| LY96 | Lymphocyte Antigen 96 | 42 |
| PRPS1L1 | Phosphoribosyl Pyrophosphate Synthetase 1 Like 1 | 37 |
| CDH7 | Cadherin 7 | 39 |
| MRC1 | Mannose Receptor C-Type 1 | 39 |
| MIR19B1 | MicroRNA 19b-1 | 17 |
| IL32 | Interleukin 32 | 40 |
| WNT10B | Wnt Family Member 10B | 48 |
| DDX39B | DExD-Box Helicase 39B | 39 |
| ENSG00000247287 |  | 8 |
| TRPC5 | Transient Receptor Potential Cation Channel Subfamily C Member 5 | 41 |
| CCDC154 | Coiled-Coil Domain Containing 154 | 29 |
| PTCRA | Pre T Cell Antigen Receptor Alpha | 37 |
| STAG3L4 | Stromal Antigen 3-Like 4 (Pseudogene) | 23 |
| CFAP251 | Cilia And Flagella Associated Protein 251 | 28 |
| ANO10 | Anoctamin 10 | 40 |
| SRSF3 | Serine And Arginine Rich Splicing Factor 3 | 40 |
| KAT5 | Lysine Acetyltransferase 5 | 46 |
| ZNF513 | Zinc Finger Protein 513 | 36 |
| MRPS6 | Mitochondrial Ribosomal Protein S6 | 37 |
| ATG9B | Autophagy Related 9B | 35 |
| ADAMTS5 | ADAM Metallopeptidase With Thrombospondin Type 1 Motif 5 | 42 |
| OMD | Osteomodulin | 37 |
| PPP3CB | Protein Phosphatase 3 Catalytic Subunit Beta | 44 |
| IFIT3 | Interferon Induced Protein With Tetratricopeptide Repeats 3 | 39 |
| C3AR1 | Complement C3a Receptor 1 | 43 |
| CLTCL1 | Clathrin Heavy Chain Like 1 | 41 |
| KCTD1 | Potassium Channel Tetramerization Domain Containing 1 | 40 |
| ASZ1 | Ankyrin Repeat, SAM And Basic Leucine Zipper Domain Containing 1 | 37 |
| MIR151A | MicroRNA 151a | 17 |
| SERPINA12 | Serpin Family A Member 12 | 38 |
| TNFRSF10C | TNF Receptor Superfamily Member 10c | 39 |
| IGFBP5 | Insulin Like Growth Factor Binding Protein 5 | 43 |
| TSR1 | TSR1 Ribosome Maturation Factor | 38 |
| GRK4 | G Protein-Coupled Receptor Kinase 4 | 43 |
| CRYBB2 | Crystallin Beta B2 | 40 |
| NSUN7 | NOP2/Sun RNA Methyltransferase Family Member 7 | 34 |
| BLZF1 | Basic Leucine Zipper Nuclear Factor 1 | 39 |
| MXD4 | MAX Dimerization Protein 4 | 36 |
| LPGAT1 | Lysophosphatidylglycerol Acyltransferase 1 | 38 |
| ITGA11 | Integrin Subunit Alpha 11 | 42 |
| LDB2 | LIM Domain Binding 2 | 38 |
| ADAM12 | ADAM Metallopeptidase Domain 12 | 45 |
| WASF2 | WASP Family Member 2 | 43 |
| REV3L | REV3 Like, DNA Directed Polymerase Zeta Catalytic Subunit | 44 |
| KCNK2 | Potassium Two Pore Domain Channel Subfamily K Member 2 | 41 |
| FHL5 | Four And A Half LIM Domains 5 | 37 |
| HIF1A-AS1 | HIF1A Antisense RNA 1 | 15 |
| RXRB | Retinoid X Receptor Beta | 47 |
| ICAM2 | Intercellular Adhesion Molecule 2 | 45 |
| BAK1 | BCL2 Antagonist/Killer 1 | 44 |
| GTPBP1 | GTP Binding Protein 1 | 38 |
| WASF1 | WASP Family Member 1 | 43 |
| CCNB1 | Cyclin B1 | 47 |
| EFNB2 | Ephrin B2 | 44 |
| TERF1 | Telomeric Repeat Binding Factor 1 | 41 |
| CROCC | Ciliary Rootlet Coiled-Coil, Rootletin | 35 |
| ABHD11 | Abhydrolase Domain Containing 11 | 36 |
| C19orf33 | Chromosome 19 Open Reading Frame 33 | 30 |
| ARHGAP29 | Rho GTPase Activating Protein 29 | 40 |
| SRI | Sorcin | 42 |
| UPF1 | UPF1 RNA Helicase And ATPase | 40 |
| MIR152 | MicroRNA 152 | 20 |
| HSPB3 | Heat Shock Protein Family B (Small) Member 3 | 39 |
| SIRT7 | Sirtuin 7 | 41 |
| PRICKLE2 | Prickle Planar Cell Polarity Protein 2 | 39 |
| BUD13 | BUD13 Homolog | 36 |
| PROZ | Protein Z, Vitamin K Dependent Plasma Glycoprotein | 39 |
| MIR141 | MicroRNA 141 | 21 |
| NECTIN2 | Nectin Cell Adhesion Molecule 2 | 36 |
| GSTZ1 | Glutathione S-Transferase Zeta 1 | 41 |
| ADPRS | ADP-Ribosylserine Hydrolase | 30 |
| FZD7 | Frizzled Class Receptor 7 | 44 |
| ROCK2 | Rho Associated Coiled-Coil Containing Protein Kinase 2 | 47 |
| ASIC2 | Acid Sensing Ion Channel Subunit 2 | 39 |
| NPTXR | Neuronal Pentraxin Receptor | 39 |
| GSK3A | Glycogen Synthase Kinase 3 Alpha | 48 |
| TIMD4 | T Cell Immunoglobulin And Mucin Domain Containing 4 | 36 |
| HLA-E | Major Histocompatibility Complex, Class I, E | 41 |
| MIA3 | MIA SH3 Domain ER Export Factor 3 | 37 |
| SLC9A3R2 | SLC9A3 Regulator 2 | 39 |
| CCN4 | Cellular Communication Network Factor 4 | 31 |
| GLO1 | Glyoxalase I | 45 |
| OXTR | Oxytocin Receptor | 45 |
| LRPAP1 | LDL Receptor Related Protein Associated Protein 1 | 43 |
| POU2F1 | POU Class 2 Homeobox 1 | 44 |
| HACL1 | 2-Hydroxyacyl-CoA Lyase 1 | 40 |
| CNP | 2',3'-Cyclic Nucleotide 3' Phosphodiesterase | 42 |
| ACP6 | Acid Phosphatase 6, Lysophosphatidic | 40 |
| NOL3 | Nucleolar Protein 3 | 43 |
| AIM2 | Absent In Melanoma 2 | 41 |
| SDC3 | Syndecan 3 | 42 |
| SLC5A6 | Solute Carrier Family 5 Member 6 | 44 |
| MYL1 | Myosin Light Chain 1 | 41 |
| TNFRSF9 | TNF Receptor Superfamily Member 9 | 44 |
| CES3 | Carboxylesterase 3 | 39 |
| DAB1 | DAB Adaptor Protein 1 | 43 |
| ETS2 | ETS Proto-Oncogene 2, Transcription Factor | 42 |
| TRIM25 | Tripartite Motif Containing 25 | 44 |
| RENBP | Renin Binding Protein | 41 |
| UBA7 | Ubiquitin Like Modifier Activating Enzyme 7 | 41 |
| WNT2 | Wnt Family Member 2 | 44 |
| PDGFD | Platelet Derived Growth Factor D | 44 |
| GNAI1 | G Protein Subunit Alpha I1 | 46 |
| SYMPK | Symplekin | 39 |
| NIT2 | Nitrilase Family Member 2 | 39 |
| FOXA2 | Forkhead Box A2 | 45 |
| PANX1 | Pannexin 1 | 43 |
| CAV2 | Caveolin 2 | 42 |
| MYO1H | Myosin IH | 35 |
| ORMDL3 | ORMDL Sphingolipid Biosynthesis Regulator 3 | 39 |
| PRDX2 | Peroxiredoxin 2 | 46 |
| PTK7 | Protein Tyrosine Kinase 7 (Inactive) | 43 |
| ANO3 | Anoctamin 3 | 39 |
| LGALS3BP | Galectin 3 Binding Protein | 41 |
| DYNLT1 | Dynein Light Chain Tctex-Type 1 | 37 |
| MED1 | Mediator Complex Subunit 1 | 41 |
| SLC26A8 | Solute Carrier Family 26 Member 8 | 41 |
| EXOSC10 | Exosome Component 10 | 39 |
| MAP2K3 | Mitogen-Activated Protein Kinase Kinase 3 | 49 |
| CD276 | CD276 Molecule | 40 |
| CRABP2 | Cellular Retinoic Acid Binding Protein 2 | 43 |
| SCX | Scleraxis BHLH Transcription Factor | 27 |
| PRKAG1 | Protein Kinase AMP-Activated Non-Catalytic Subunit Gamma 1 | 48 |
| ACVR2A | Activin A Receptor Type 2A | 45 |
| PITRM1 | Pitrilysin Metallopeptidase 1 | 39 |
| HDAC3 | Histone Deacetylase 3 | 49 |
| SSTR2 | Somatostatin Receptor 2 | 47 |
| CDK9 | Cyclin Dependent Kinase 9 | 45 |
| MIR574 | MicroRNA 574 | 18 |
| ABHD11-AS1 | ABHD11 Antisense RNA 1 (Tail To Tail) | 16 |
| ADAM33 | ADAM Metallopeptidase Domain 33 | 36 |
| FES | FES Proto-Oncogene, Tyrosine Kinase | 48 |
| MMP10 | Matrix Metallopeptidase 10 | 46 |
| ATP5PF | ATP Synthase Peripheral Stalk Subunit F6 | 31 |
| HRG | Histidine Rich Glycoprotein | 41 |
| CENPA | Centromere Protein A | 39 |
| SULF1 | Sulfatase 1 | 41 |
| NPNT | Nephronectin | 38 |
| MIR376C | MicroRNA 376c | 17 |
| MAPRE3 | Microtubule Associated Protein RP/EB Family Member 3 | 39 |
| GRM7 | Glutamate Metabotropic Receptor 7 | 44 |
| OPRK1 | Opioid Receptor Kappa 1 | 45 |
| PHETA1 | PH Domain Containing Endocytic Trafficking Adaptor 1 | 27 |
| APLP2 | Amyloid Beta Precursor Like Protein 2 | 41 |
| DMBT1 | Deleted In Malignant Brain Tumors 1 | 40 |
| AGTRAP | Angiotensin II Receptor Associated Protein | 38 |
| CEP295 | Centrosomal Protein 295 | 28 |
| PSD3 | Pleckstrin And Sec7 Domain Containing 3 | 39 |
| HTN3 | Histatin 3 | 35 |
| CNTN5 | Contactin 5 | 36 |
| TCF7 | Transcription Factor 7 | 44 |
| PTPN13 | Protein Tyrosine Phosphatase Non-Receptor Type 13 | 43 |
| FAM126A | Family With Sequence Similarity 126 Member A | 37 |
| CDC25A | Cell Division Cycle 25A | 47 |
| DGCR5 | DiGeorge Syndrome Critical Region Gene 5 | 20 |
| HNRNPC | Heterogeneous Nuclear Ribonucleoprotein C | 41 |
| CCL13 | C-C Motif Chemokine Ligand 13 | 38 |
| MIR495 | MicroRNA 495 | 16 |
| SHROOM3 | Shroom Family Member 3 | 37 |
| LGALS4 | Galectin 4 | 38 |
| PFN1 | Profilin 1 | 47 |
| MGST2 | Microsomal Glutathione S-Transferase 2 | 43 |
| FERD3L | Fer3 Like BHLH Transcription Factor | 33 |
| SLC6A6 | Solute Carrier Family 6 Member 6 | 44 |
| PTPRM | Protein Tyrosine Phosphatase Receptor Type M | 41 |
| TIAM1 | TIAM Rac1 Associated GEF 1 | 44 |
| P2RY1 | Purinergic Receptor P2Y1 | 45 |
| CYP46A1 | Cytochrome P450 Family 46 Subfamily A Member 1 | 41 |
| CFDP1 | Craniofacial Development Protein 1 | 36 |
| LIPK | Lipase Family Member K | 34 |
| APH1B | Aph-1 Homolog B, Gamma-Secretase Subunit | 43 |
| IRAK3 | Interleukin 1 Receptor Associated Kinase 3 | 48 |
| MACF1 | Microtubule Actin Crosslinking Factor 1 | 41 |
| RPS3 | Ribosomal Protein S3 | 44 |
| CTTN | Cortactin | 43 |
| APOM | Apolipoprotein M | 40 |
| SCG2 | Secretogranin II | 39 |
| PTPN6 | Protein Tyrosine Phosphatase Non-Receptor Type 6 | 50 |
| EHBP1 | EH Domain Binding Protein 1 | 38 |
| SLC25A28 | Solute Carrier Family 25 Member 28 | 38 |
| FUT8 | Fucosyltransferase 8 | 43 |
| SULT1A1 | Sulfotransferase Family 1A Member 1 | 43 |
| MRTFA | Myocardin Related Transcription Factor A | 34 |
| MYO1C | Myosin IC | 43 |
| LOC102723692 | Uncharacterized LOC102723692 | 8 |
| HLX | H2.0 Like Homeobox | 40 |
| TERF2 | Telomeric Repeat Binding Factor 2 | 40 |
| PPM1K | Protein Phosphatase, Mg2+/Mn2+ Dependent 1K | 41 |
| CHURC1 | Churchill Domain Containing 1 | 32 |
| TTLL5 | Tubulin Tyrosine Ligase Like 5 | 40 |
| CD7 | CD7 Molecule | 39 |
| PPL | Periplakin | 40 |
| LRP1B | LDL Receptor Related Protein 1B | 39 |
| ESRRG | Estrogen Related Receptor Gamma | 45 |
| OPLAH | 5-Oxoprolinase, ATP-Hydrolysing | 41 |
| AGBL1 | ATP/GTP Binding Protein Like 1 | 36 |
| DSC1 | Desmocollin 1 | 38 |
| GPT2 | Glutamic--Pyruvic Transaminase 2 | 46 |
| UGT1A8 | UDP Glucuronosyltransferase Family 1 Member A8 | 35 |
| NAV2 | Neuron Navigator 2 | 37 |
| INPP5D | Inositol Polyphosphate-5-Phosphatase D | 45 |
| CMKLR1 | Chemerin Chemokine-Like Receptor 1 | 40 |
| PTPRD | Protein Tyrosine Phosphatase Receptor Type D | 44 |
| HSPB6 | Heat Shock Protein Family B (Small) Member 6 | 39 |
| LOC106029311 | Williams-Beuren Syndrome Centromeric Block B Recombination Region | 1 |
| LOC106029313 | Williams-Beuren Syndrome Telomeric Block B Recombination Region | 1 |
| AFA1 | Alopecia, Androgenetic | 2 |
| AGA2 | Alopecia, Androgenetic, 2 | 2 |
| AGA3 | Alopecia, Androgenetic, 3 | 2 |
| ANGPTL8 | Angiopoietin Like 8 | 28 |
| LTB4R | Leukotriene B4 Receptor | 44 |
| SYNE3 | Spectrin Repeat Containing Nuclear Envelope Family Member 3 | 34 |
| ACSS2 | Acyl-CoA Synthetase Short Chain Family Member 2 | 43 |
| IGES | Immunoglobulin E Concentration, Serum | 4 |
| NEDD9 | Neural Precursor Cell Expressed, Developmentally Down-Regulated 9 | 42 |
| MIP | Major Intrinsic Protein Of Lens Fiber | 41 |
| HLF | HLF Transcription Factor, PAR BZIP Family Member | 40 |
| PIM1 | Pim-1 Proto-Oncogene, Serine/Threonine Kinase | 50 |
| CRIM1 | Cysteine Rich Transmembrane BMP Regulator 1 | 39 |
| PRRC2A | Proline Rich Coiled-Coil 2A | 33 |
| CEBPB | CCAAT Enhancer Binding Protein Beta | 43 |
| MIR103A1 | MicroRNA 103a-1 | 17 |
| ANKRD50 | Ankyrin Repeat Domain 50 | 33 |
| TCF7L1 | Transcription Factor 7 Like 1 | 41 |
| FLRT2 | Fibronectin Leucine Rich Transmembrane Protein 2 | 38 |
| RFX5 | Regulatory Factor X5 | 41 |
| F2RL3 | F2R Like Thrombin Or Trypsin Receptor 3 | 45 |
| ZBTB20 | Zinc Finger And BTB Domain Containing 20 | 40 |
| NRXN3 | Neurexin 3 | 41 |
| MLST8 | MTOR Associated Protein, LST8 Homolog | 42 |
| MRRF | Mitochondrial Ribosome Recycling Factor | 39 |
| POLE | DNA Polymerase Epsilon, Catalytic Subunit | 48 |
| ANXA11 | Annexin A11 | 44 |
| ARID5B | AT-Rich Interaction Domain 5B | 40 |
| RHOC | Ras Homolog Family Member C | 41 |
| RIPK2 | Receptor Interacting Serine/Threonine Kinase 2 | 46 |
| BCAM | Basal Cell Adhesion Molecule (Lutheran Blood Group) | 40 |
| BIRC2 | Baculoviral IAP Repeat Containing 2 | 45 |
| SDC2 | Syndecan 2 | 45 |
| NRF1 | Nuclear Respiratory Factor 1 | 43 |
| ENPP2 | Ectonucleotide Pyrophosphatase/Phosphodiesterase 2 | 44 |
| CUX2 | Cut Like Homeobox 2 | 40 |
| SLC45A2 | Solute Carrier Family 45 Member 2 | 40 |
| PRKAG3 | Protein Kinase AMP-Activated Non-Catalytic Subunit Gamma 3 | 43 |
| PLSCR3 | Phospholipid Scramblase 3 | 37 |
| CYP2R1 | Cytochrome P450 Family 2 Subfamily R Member 1 | 45 |
| PCSK5 | Proprotein Convertase Subtilisin/Kexin Type 5 | 41 |
| KHK | Ketohexokinase | 44 |
| LIMK2 | LIM Domain Kinase 2 | 47 |
| RDX | Radixin | 48 |
| PLIN2 | Perilipin 2 | 43 |
| NDUFS5 | NADH:Ubiquinone Oxidoreductase Subunit S5 | 40 |
| NBPF12 | NBPF Member 12 | 28 |
| CHD6 | Chromodomain Helicase DNA Binding Protein 6 | 37 |
| GPS1 | G Protein Pathway Suppressor 1 | 37 |
| LMAN1 | Lectin, Mannose Binding 1 | 45 |
| C1QTNF3 | C1q And TNF Related 3 | 38 |
| CARD8 | Caspase Recruitment Domain Family Member 8 | 39 |
| UGT2B7 | UDP Glucuronosyltransferase Family 2 Member B7 | 44 |
| C5AR2 | Complement Component 5a Receptor 2 | 39 |
| MAPKAPK2 | MAPK Activated Protein Kinase 2 | 48 |
| SERPINA4 | Serpin Family A Member 4 | 41 |
| EXOSC3 | Exosome Component 3 | 42 |
| DPEP1 | Dipeptidase 1 | 42 |
| SLC24A3 | Solute Carrier Family 24 Member 3 | 39 |
| TAL1 | TAL BHLH Transcription Factor 1, Erythroid Differentiation Factor | 45 |
| SORCS1 | Sortilin Related VPS10 Domain Containing Receptor 1 | 38 |
| MIR432 | MicroRNA 432 | 17 |
| ADAMTS1 | ADAM Metallopeptidase With Thrombospondin Type 1 Motif 1 | 44 |
| DLGAP1 | DLG Associated Protein 1 | 43 |
| BAZ2B | Bromodomain Adjacent To Zinc Finger Domain 2B | 39 |
| FMO1 | Flavin Containing Dimethylaniline Monoxygenase 1 | 43 |
| PPM1D | Protein Phosphatase, Mg2+/Mn2+ Dependent 1D | 47 |
| ABCC3 | ATP Binding Cassette Subfamily C Member 3 | 45 |
| TRIB2 | Tribbles Pseudokinase 2 | 37 |
| ART4 | ADP-Ribosyltransferase 4 (Dombrock Blood Group) | 39 |
| COL8A1 | Collagen Type VIII Alpha 1 Chain | 39 |
| PARVB | Parvin Beta | 39 |
| TNFRSF12A | TNF Receptor Superfamily Member 12A | 44 |
| CA1 | Carbonic Anhydrase 1 | 47 |
| DNAJC30 | DnaJ Heat Shock Protein Family (Hsp40) Member C30 | 33 |
| NFE2 | Nuclear Factor, Erythroid 2 | 40 |
| FAP | Fibroblast Activation Protein Alpha | 42 |
| RPS13 | Ribosomal Protein S13 | 42 |
| DMRTA1 | DMRT Like Family A1 | 36 |
| SOBP | Sine Oculis Binding Protein Homolog | 38 |
| TYROBP | Transmembrane Immune Signaling Adaptor TYROBP | 43 |
| ARHGAP9 | Rho GTPase Activating Protein 9 | 41 |
| OPRL1 | Opioid Related Nociceptin Receptor 1 | 45 |
| ST2 | Suppression Of Tumorigenicity 2 | 6 |
| MIR1306 | MicroRNA 1306 | 17 |
| ZHX3 | Zinc Fingers And Homeoboxes 3 | 35 |
| MIR6886 | MicroRNA 6886 | 9 |
| NIF3L1 | NGG1 Interacting Factor 3 Like 1 | 39 |
| CYP26A1 | Cytochrome P450 Family 26 Subfamily A Member 1 | 44 |
| TDP2 | Tyrosyl-DNA Phosphodiesterase 2 | 40 |
| LPAL2 | Lipoprotein(A) Like 2, Pseudogene | 27 |
| MIR374B | MicroRNA 374b | 16 |
| CPNE4 | Copine 4 | 38 |
| OPRD1 | Opioid Receptor Delta 1 | 44 |
| ARL15 | ADP Ribosylation Factor Like GTPase 15 | 36 |
| TBPL1 | TATA-Box Binding Protein Like 1 | 40 |
| PRKAB2 | Protein Kinase AMP-Activated Non-Catalytic Subunit Beta 2 | 46 |
| NDUFC2 | NADH:Ubiquinone Oxidoreductase Subunit C2 | 39 |
| C1QTNF1 | C1q And TNF Related 1 | 39 |
| TOX | Thymocyte Selection Associated High Mobility Group Box | 40 |
| TCF15 | Transcription Factor 15 | 33 |
| EPN1 | Epsin 1 | 40 |
| TOM1L2 | Target Of Myb1 Like 2 Membrane Trafficking Protein | 38 |
| ZNF365 | Zinc Finger Protein 365 | 37 |
| RPTOR | Regulatory Associated Protein Of MTOR Complex 1 | 44 |
| SPDYE12P | Speedy/RINGO Cell Cycle Regulator Family Member E12, Pseudogene | 8 |
| FADS3 | Fatty Acid Desaturase 3 | 37 |
| GPR89A | G Protein-Coupled Receptor 89A | 32 |
| CHST6 | Carbohydrate Sulfotransferase 6 | 43 |
| KCNJ16 | Potassium Inwardly Rectifying Channel Subfamily J Member 16 | 39 |
| NSUN5P2 | NSUN5 Pseudogene 2 | 24 |
| SIGLEC1 | Sialic Acid Binding Ig Like Lectin 1 | 40 |
| RER1 | Retention In Endoplasmic Reticulum Sorting Receptor 1 | 35 |
| BLVRB | Biliverdin Reductase B | 40 |
| SMPD2 | Sphingomyelin Phosphodiesterase 2 | 40 |
| CCR8 | C-C Motif Chemokine Receptor 8 | 43 |
| PTAFR | Platelet Activating Factor Receptor | 43 |
| PCMT1 | Protein-L-Isoaspartate (D-Aspartate) O-Methyltransferase | 40 |
| SLC2A13 | Solute Carrier Family 2 Member 13 | 41 |
| PKD1L3 | Polycystin 1 Like 3, Transient Receptor Potential Channel Interacting | 31 |
| SKP1 | S-Phase Kinase Associated Protein 1 | 43 |
| SSRP1 | Structure Specific Recognition Protein 1 | 40 |
| ITPK1 | Inositol-Tetrakisphosphate 1-Kinase | 41 |
| GNLY | Granulysin | 38 |
| PPP1R12B | Protein Phosphatase 1 Regulatory Subunit 12B | 39 |
| FBLIM1 | Filamin Binding LIM Protein 1 | 38 |
| UGT1A6 | UDP Glucuronosyltransferase Family 1 Member A6 | 40 |
| NLRP6 | NLR Family Pyrin Domain Containing 6 | 38 |
| TOMM20 | Translocase Of Outer Mitochondrial Membrane 20 | 39 |
| POM121C | POM121 Transmembrane Nucleoporin C | 31 |
| MIR17HG | MiR-17-92a-1 Cluster Host Gene | 28 |
| TYRP1 | Tyrosinase Related Protein 1 | 46 |
| BDKRB1 | Bradykinin Receptor B1 | 43 |
| HSPE1 | Heat Shock Protein Family E (Hsp10) Member 1 | 40 |
| IL18RAP | Interleukin 18 Receptor Accessory Protein | 39 |
| GP5 | Glycoprotein V Platelet | 40 |
| ACAD10 | Acyl-CoA Dehydrogenase Family Member 10 | 38 |
| PPP2R5A | Protein Phosphatase 2 Regulatory Subunit B'Alpha | 40 |
| GTF2IRD2B | GTF2I Repeat Domain Containing 2B | 30 |
| ORAI1 | ORAI Calcium Release-Activated Calcium Modulator 1 | 44 |
| FMN1 | Formin 1 | 38 |
| TRIM5 | Tripartite Motif Containing 5 | 41 |
| HPX | Hemopexin | 39 |
| TCN1 | Transcobalamin 1 | 40 |
| CXCR6 | C-X-C Motif Chemokine Receptor 6 | 40 |
| SSBP3 | Single Stranded DNA Binding Protein 3 | 38 |
| AQP9 | Aquaporin 9 | 43 |
| PLCD1 | Phospholipase C Delta 1 | 48 |
| TMPRSS2 | Transmembrane Serine Protease 2 | 44 |
| RBM24 | RNA Binding Motif Protein 24 | 35 |
| LTC4S | Leukotriene C4 Synthase | 42 |
| LRP12 | LDL Receptor Related Protein 12 | 40 |
| RTN4R | Reticulon 4 Receptor | 43 |
| COQ5 | Coenzyme Q5, Methyltransferase | 39 |
| AIMP1 | Aminoacyl TRNA Synthetase Complex Interacting Multifunctional Protein 1 | 43 |
| MSH5 | MutS Homolog 5 | 41 |
| WWTR1 | WW Domain Containing Transcription Regulator 1 | 41 |
| ELMOD2 | ELMO Domain Containing 2 | 36 |
| MMP23B | Matrix Metallopeptidase 23B | 37 |
| AGPAT1 | 1-Acylglycerol-3-Phosphate O-Acyltransferase 1 | 41 |
| AQP7 | Aquaporin 7 | 44 |
| MAPKAPK3 | MAPK Activated Protein Kinase 3 | 49 |
| CASC15 | Cancer Susceptibility 15 | 18 |
| MIRLET7I | MicroRNA Let-7i | 20 |
| MIR186 | MicroRNA 186 | 18 |
| PSMG1 | Proteasome Assembly Chaperone 1 | 37 |
| SSTR5 | Somatostatin Receptor 5 | 41 |
| RAPGEF3 | Rap Guanine Nucleotide Exchange Factor 3 | 44 |
| TRIM74 | Tripartite Motif Containing 74 | 29 |
| PAWR | Pro-Apoptotic WT1 Regulator | 40 |
| MIR361 | MicroRNA 361 | 16 |
| CD226 | CD226 Molecule | 43 |
| MYO1E | Myosin IE | 43 |
| TNFRSF6B | TNF Receptor Superfamily Member 6b | 43 |
| VAV3 | Vav Guanine Nucleotide Exchange Factor 3 | 43 |
| CYP4V2 | Cytochrome P450 Family 4 Subfamily V Member 2 | 42 |
| FCER1A | Fc Fragment Of IgE Receptor Ia | 43 |
| BRCC3 | BRCA1/BRCA2-Containing Complex Subunit 3 | 43 |
| CLDN11 | Claudin 11 | 40 |
| TMEM87B | Transmembrane Protein 87B | 35 |
| NAXE | NAD(P)HX Epimerase | 34 |
| C4BPA | Complement Component 4 Binding Protein Alpha | 40 |
| SFXN4 | Sideroflexin 4 | 41 |
| SLC16A1 | Solute Carrier Family 16 Member 1 | 48 |
| CYP2B6 | Cytochrome P450 Family 2 Subfamily B Member 6 | 47 |
| SVIP | Small VCP Interacting Protein | 33 |
| NCOA6 | Nuclear Receptor Coactivator 6 | 39 |
| CMYA5 | Cardiomyopathy Associated 5 | 33 |
| RBP1 | Retinol Binding Protein 1 | 39 |
| DMWD | DM1 Locus, WD Repeat Containing | 35 |
| USP24 | Ubiquitin Specific Peptidase 24 | 43 |
| HTR4 | 5-Hydroxytryptamine Receptor 4 | 45 |
| FBN3 | Fibrillin 3 | 36 |
| B4GALNT2 | Beta-1,4-N-Acetyl-Galactosaminyltransferase 2 | 40 |
| MROS | Melkersson-Rosenthal Syndrome | 3 |
| TANC1 | Tetratricopeptide Repeat, Ankyrin Repeat And Coiled-Coil Containing 1 | 33 |
| PRSS2 | Serine Protease 2 | 39 |
| ERG | ETS Transcription Factor ERG | 45 |
| SPDYE1 | Speedy/RINGO Cell Cycle Regulator Family Member E1 | 29 |
| STRN | Striatin | 39 |
| LTB | Lymphotoxin Beta | 40 |
| MLC1 | Modulator Of VRAC Current 1 | 41 |
| APH1A | Aph-1 Homolog A, Gamma-Secretase Subunit | 43 |
| CHRNB4 | Cholinergic Receptor Nicotinic Beta 4 Subunit | 43 |
| DNALI1 | Dynein Axonemal Light Intermediate Chain 1 | 38 |
| SULT2B1 | Sulfotransferase Family 2B Member 1 | 45 |
| APEH | Acylaminoacyl-Peptide Hydrolase | 40 |
| GIMAP5 | GTPase, IMAP Family Member 5 | 36 |
| HSF1 | Heat Shock Transcription Factor 1 | 44 |
| TNKS | Tankyrase | 43 |
| LY86 | Lymphocyte Antigen 86 | 37 |
| SH3GL2 | SH3 Domain Containing GRB2 Like 2, Endophilin A1 | 43 |
| ZNF648 | Zinc Finger Protein 648 | 31 |
| PROX2 | Prospero Homeobox 2 | 31 |
| STAG3L3 | Stromal Antigen 3-Like 3 (Pseudogene) | 19 |
| FABP5 | Fatty Acid Binding Protein 5 | 41 |
| MFSD10 | Major Facilitator Superfamily Domain Containing 10 | 35 |
| HPR | Haptoglobin-Related Protein | 40 |
| ITGAE | Integrin Subunit Alpha E | 39 |
| PSMC5 | Proteasome 26S Subunit, ATPase 5 | 41 |
| MXD1 | MAX Dimerization Protein 1 | 37 |
| ALDH9A1 | Aldehyde Dehydrogenase 9 Family Member A1 | 41 |
| BAD | BCL2 Associated Agonist Of Cell Death | 46 |
| CHD9 | Chromodomain Helicase DNA Binding Protein 9 | 37 |
| MAD1L1 | Mitotic Arrest Deficient 1 Like 1 | 45 |
| TRIM55 | Tripartite Motif Containing 55 | 37 |
| DLG2 | Discs Large MAGUK Scaffold Protein 2 | 40 |
| THADA | THADA Armadillo Repeat Containing | 37 |
| SKP2 | S-Phase Kinase Associated Protein 2 | 44 |
| ZNF627 | Zinc Finger Protein 627 | 35 |
| PRIMPOL | Primase And DNA Directed Polymerase | 33 |
| NBPF3 | NBPF Member 3 | 35 |
| ARHGAP10 | Rho GTPase Activating Protein 10 | 39 |
| LGALS9 | Galectin 9 | 39 |
| FGF19 | Fibroblast Growth Factor 19 | 44 |
| FCAR | Fc Fragment Of IgA Receptor | 41 |
| NSUN5P1 | NSUN5 Pseudogene 1 | 20 |
| IGFBP6 | Insulin Like Growth Factor Binding Protein 6 | 42 |
| NCL | Nucleolin | 42 |
| RAB11B | RAB11B, Member RAS Oncogene Family | 42 |
| STAG3L1 | Stromal Antigen 3-Like 1 (Pseudogene) | 24 |
| SPRED2 | Sprouty Related EVH1 Domain Containing 2 | 41 |
| PPM1G | Protein Phosphatase, Mg2+/Mn2+ Dependent 1G | 43 |
| HNRNPM | Heterogeneous Nuclear Ribonucleoprotein M | 39 |
| YRDC | YrdC N6-Threonylcarbamoyltransferase Domain Containing | 32 |
| TEAD3 | TEA Domain Transcription Factor 3 | 39 |
| MEOX2 | Mesenchyme Homeobox 2 | 41 |
| CPQ | Carboxypeptidase Q | 35 |
| GTF2IRD2P1 | GTF2I Repeat Domain Containing 2 Pseudogene 1 | 12 |
| BDH1 | 3-Hydroxybutyrate Dehydrogenase 1 | 43 |
| ACKR2 | Atypical Chemokine Receptor 2 | 37 |
| RFXAP | Regulatory Factor X Associated Protein | 37 |
| PMVK | Phosphomevalonate Kinase | 45 |
| FTMT | Ferritin Mitochondrial | 38 |
| TRIM73 | Tripartite Motif Containing 73 | 28 |
| E2F1 | E2F Transcription Factor 1 | 43 |
| S100A6 | S100 Calcium Binding Protein A6 | 43 |
| CRADD | CASP2 And RIPK1 Domain Containing Adaptor With Death Domain | 44 |
| NR1D1 | Nuclear Receptor Subfamily 1 Group D Member 1 | 47 |
| AMPD2 | Adenosine Monophosphate Deaminase 2 | 46 |
| FBF1 | Fas Binding Factor 1 | 33 |
| ACKR3 | Atypical Chemokine Receptor 3 | 40 |
| COL22A1 | Collagen Type XXII Alpha 1 Chain | 36 |
| KLRD1 | Killer Cell Lectin Like Receptor D1 | 41 |
| TMEM270 | Transmembrane Protein 270 | 23 |
| AGXT2 | Alanine--Glyoxylate Aminotransferase 2 | 40 |
| GTF2IP4 | General Transcription Factor IIi Pseudogene 4 | 9 |
| AFF1 | AF4/FMR2 Family Member 1 | 38 |
| PTN | Pleiotrophin | 43 |
| FPR1 | Formyl Peptide Receptor 1 | 47 |
| STAG3L2 | Stromal Antigen 3-Like 2 (Pseudogene) | 21 |
| CD177 | CD177 Molecule | 39 |
| GTF2IP1 | General Transcription Factor IIi Pseudogene 1 | 13 |
| SPDYE10P | Speedy/RINGO Cell Cycle Regulator Family Member E10, Pseudogene | 6 |
| IL1R2 | Interleukin 1 Receptor Type 2 | 45 |
| CEBPD | CCAAT Enhancer Binding Protein Delta | 38 |
| ETV1 | ETS Variant Transcription Factor 1 | 46 |
| ADHFE1 | Alcohol Dehydrogenase Iron Containing 1 | 37 |
| MIR34B | MicroRNA 34b | 21 |
| KANTR | KDM5C Adjacent Transcript | 17 |
| NRG3 | Neuregulin 3 | 41 |
| PPP3R1 | Protein Phosphatase 3 Regulatory Subunit B, Alpha | 45 |
| SLC44A2 | Solute Carrier Family 44 Member 2 | 40 |
| PLCL1 | Phospholipase C Like 1 (Inactive) | 39 |
| PPY | Pancreatic Polypeptide | 40 |
| LINC00851 | Long Intergenic Non-Protein Coding RNA 851 | 16 |
| WDR6 | WD Repeat Domain 6 | 35 |
| IGFBP4 | Insulin Like Growth Factor Binding Protein 4 | 43 |
| ILF3 | Interleukin Enhancer Binding Factor 3 | 38 |
| ECM2 | Extracellular Matrix Protein 2 | 37 |
| NRBP1 | Nuclear Receptor Binding Protein 1 | 39 |
| NLRP5 | NLR Family Pyrin Domain Containing 5 | 37 |
| HIVEP2 | HIVEP Zinc Finger 2 | 37 |
| CCL24 | C-C Motif Chemokine Ligand 24 | 36 |
| STC1 | Stanniocalcin 1 | 40 |
| IL31 | Interleukin 31 | 35 |
| VIPR1 | Vasoactive Intestinal Peptide Receptor 1 | 45 |
| SPDYE13 | Speedy/RINGO Cell Cycle Regulator Family Member E13 | 9 |
| BMX | BMX Non-Receptor Tyrosine Kinase | 44 |
| RHOJ | Ras Homolog Family Member J | 38 |
| GLB1L3 | Galactosidase Beta 1 Like 3 | 36 |
| KLHL26 | Kelch Like Family Member 26 | 35 |
| CNDP1 | Carnosine Dipeptidase 1 | 44 |
| MED18 | Mediator Complex Subunit 18 | 35 |
| ARHGAP15 | Rho GTPase Activating Protein 15 | 40 |
| MTNR1A | Melatonin Receptor 1A | 44 |
| SPDYE9 | Speedy/RINGO Cell Cycle Regulator Family Member E9 | 6 |
| THSD7A | Thrombospondin Type 1 Domain Containing 7A | 35 |
| CNOT3 | CCR4-NOT Transcription Complex Subunit 3 | 41 |
| ALOX12B | Arachidonate 12-Lipoxygenase, 12R Type | 43 |
| COL21A1 | Collagen Type XXI Alpha 1 Chain | 36 |
| GTDC1 | Glycosyltransferase Like Domain Containing 1 | 36 |
| OAT | Ornithine Aminotransferase | 47 |
| PFKP | Phosphofructokinase, Platelet | 45 |
| NTM | Neurotrimin | 41 |
| SPDYE7P | Speedy/RINGO Cell Cycle Regulator Family Member E7, Pseudogene | 18 |
| SPDYE15 | Speedy/RINGO Cell Cycle Regulator Family Member E15 | 8 |
| EIF4HP1 | Eukaryotic Translation Initiation Factor 4H Pseudogene 1 | 6 |
| GPR182 | G Protein-Coupled Receptor 182 | 37 |
| TUBA1B | Tubulin Alpha 1b | 42 |
| BHMT2 | Betaine--Homocysteine S-Methyltransferase 2 | 41 |
| NDUFAB1 | NADH:Ubiquinone Oxidoreductase Subunit AB1 | 40 |
| PFAS | Phosphoribosylformylglycinamidine Synthase | 42 |
| ESAM | Endothelial Cell Adhesion Molecule | 40 |
| PPP1R3B | Protein Phosphatase 1 Regulatory Subunit 3B | 37 |
| BDNF-AS | BDNF Antisense RNA | 18 |
| SSTR1 | Somatostatin Receptor 1 | 43 |
| HSD17B12 | Hydroxysteroid 17-Beta Dehydrogenase 12 | 40 |
| NBPF20 | NBPF Member 20 | 22 |
| AGAP1 | ArfGAP With GTPase Domain, Ankyrin Repeat And PH Domain 1 | 39 |
| DHX36 | DEAH-Box Helicase 36 | 40 |
| CCNE1 | Cyclin E1 | 48 |
| ABHD16A | Abhydrolase Domain Containing 16A, Phospholipase | 34 |
| TRPC4 | Transient Receptor Potential Cation Channel Subfamily C Member 4 | 44 |
| MTSS1 | MTSS I-BAR Domain Containing 1 | 38 |
| MIR381 | MicroRNA 381 | 17 |
| NUP210 | Nucleoporin 210 | 39 |
| MAZ | MYC Associated Zinc Finger Protein | 40 |
| DUOXA1 | Dual Oxidase Maturation Factor 1 | 36 |
| CXCL6 | C-X-C Motif Chemokine Ligand 6 | 40 |
| LOC108228208 | 7q11.23 Proximal Recombination Region | 1 |
| LOC108228209 | 7q11.23 Distal Recombination Region | 1 |
| PCOLCE2 | Procollagen C-Endopeptidase Enhancer 2 | 35 |
| RND2 | Rho Family GTPase 2 | 36 |
| SPDYE8 | Speedy/RINGO Cell Cycle Regulator Family Member E8 | 10 |
| SPDYE14 | Speedy/RINGO Cell Cycle Regulator Family Member E14 | 7 |
| WBSCR2 | Williams-Beuren Syndrome Chromosome Region 2 | 5 |
| WBSCR23 | Williams-Beuren Syndrome Chromosome Region 23 | 5 |
| NKX2-3 | NK2 Homeobox 3 | 37 |
| RAD51B | RAD51 Paralog B | 37 |
| UBIAD1 | UbiA Prenyltransferase Domain Containing 1 | 41 |
| MT3 | Metallothionein 3 | 40 |
| CADPS | Calcium Dependent Secretion Activator | 41 |
| PLEKHO1 | Pleckstrin Homology Domain Containing O1 | 35 |
| GINS2 | GINS Complex Subunit 2 | 36 |
| EMP1 | Epithelial Membrane Protein 1 | 35 |
| RSAD2 | Radical S-Adenosyl Methionine Domain Containing 2 | 40 |
| HHAT | Hedgehog Acyltransferase | 41 |
| PSMC3 | Proteasome 26S Subunit, ATPase 3 | 41 |
| ALMS1P1 | ALMS1 Pseudogene 1 | 13 |
| PDGFC | Platelet Derived Growth Factor C | 43 |
| PCNX3 | Pecanex 3 | 25 |
| CD47 | CD47 Molecule | 43 |
| DSTN | Destrin, Actin Depolymerizing Factor | 39 |
| MIR107 | MicroRNA 107 | 19 |
| LRRC37A2 | Leucine Rich Repeat Containing 37 Member A2 | 29 |
| CSH1 | Chorionic Somatomammotropin Hormone 1 | 38 |
| PPP1R14C | Protein Phosphatase 1 Regulatory Inhibitor Subunit 14C | 37 |
| KLF12 | Kruppel Like Factor 12 | 38 |
| BACH1 | BTB Domain And CNC Homolog 1 | 41 |
| DCBLD2 | Discoidin, CUB And LCCL Domain Containing 2 | 40 |
| ST3GAL3 | ST3 Beta-Galactoside Alpha-2,3-Sialyltransferase 3 | 45 |
| DUSP19 | Dual Specificity Phosphatase 19 | 39 |
| PRDM9 | PR/SET Domain 9 | 39 |
| GPC2 | Glypican 2 | 36 |
| TXNRD1 | Thioredoxin Reductase 1 | 47 |
| ZFPM2-AS1 | ZFPM2 Antisense RNA 1 | 13 |
| PPP1R14A | Protein Phosphatase 1 Regulatory Inhibitor Subunit 14A | 40 |
| TICAM1 | Toll Like Receptor Adaptor Molecule 1 | 44 |
| RCC1 | Regulator Of Chromosome Condensation 1 | 40 |
| KCNK17 | Potassium Two Pore Domain Channel Subfamily K Member 17 | 36 |
| PAPPA2 | Pappalysin 2 | 37 |
| NAT1 | N-Acetyltransferase 1 | 44 |
| EDC4 | Enhancer Of MRNA Decapping 4 | 38 |
| RPLP1 | Ribosomal Protein Lateral Stalk Subunit P1 | 40 |
| ANGPTL2 | Angiopoietin Like 2 | 36 |
| DNM3 | Dynamin 3 | 43 |
| MAML3 | Mastermind Like Transcriptional Coactivator 3 | 38 |
| TNP1 | Transition Protein 1 | 34 |
| NBPF10 | NBPF Member 10 | 27 |
| MIR638 | MicroRNA 638 | 17 |
| MIR485 | MicroRNA 485 | 17 |
| FAM189A2 | Family With Sequence Similarity 189 Member A2 | 34 |
| C16orf95 | Chromosome 16 Open Reading Frame 95 | 26 |
| CLEC12A | C-Type Lectin Domain Family 12 Member A | 37 |
| SLC29A2 | Solute Carrier Family 29 Member 2 | 45 |
| PGPEP1 | Pyroglutamyl-Peptidase I | 36 |
| SEMA3F | Semaphorin 3F | 41 |
| SLC12A9 | Solute Carrier Family 12 Member 9 | 37 |
| CABLES1 | Cdk5 And Abl Enzyme Substrate 1 | 39 |
| LNX1 | Ligand Of Numb-Protein X 1 | 43 |
| ARHGDIA | Rho GDP Dissociation Inhibitor Alpha | 47 |
| MIR409 | MicroRNA 409 | 18 |
| GPER1 | G Protein-Coupled Estrogen Receptor 1 | 37 |
| MTHFSD | Methenyltetrahydrofolate Synthetase Domain Containing | 35 |
| GSTO1 | Glutathione S-Transferase Omega 1 | 43 |
| PCDH7 | Protocadherin 7 | 39 |
| PTPRN2 | Protein Tyrosine Phosphatase Receptor Type N2 | 44 |
| IL3RA | Interleukin 3 Receptor Subunit Alpha | 42 |
| GSTM3 | Glutathione S-Transferase Mu 3 | 45 |
| AKAP7 | A-Kinase Anchoring Protein 7 | 38 |
| IFNAR2 | Interferon Alpha And Beta Receptor Subunit 2 | 48 |
| CSRP1 | Cysteine And Glycine Rich Protein 1 | 41 |
| TES | Testin LIM Domain Protein | 41 |
| POFUT2 | Protein O-Fucosyltransferase 2 | 38 |
| DDT | D-Dopachrome Tautomerase | 39 |
| ABCC4 | ATP Binding Cassette Subfamily C Member 4 | 45 |
| CD93 | CD93 Molecule | 40 |
| RMI2 | RecQ Mediated Genome Instability 2 | 33 |
| QRICH1 | Glutamine Rich 1 | 35 |
| HTR6 | 5-Hydroxytryptamine Receptor 6 | 43 |
| FAM189B | Family With Sequence Similarity 189 Member B | 32 |
| PGGT1B | Protein Geranylgeranyltransferase Type I Subunit Beta | 37 |
| WDFY4 | WDFY Family Member 4 | 35 |
| CLASRP | CLK4 Associating Serine/Arginine Rich Protein | 32 |
| GUCA2A | Guanylate Cyclase Activator 2A | 37 |
| MPRIP | Myosin Phosphatase Rho Interacting Protein | 37 |
| HEPHL1 | Hephaestin Like 1 | 37 |
| CDH4 | Cadherin 4 | 41 |
| HSPA14 | Heat Shock Protein Family A (Hsp70) Member 14 | 39 |
| HMGCS2 | 3-Hydroxy-3-Methylglutaryl-CoA Synthase 2 | 44 |
| AKAP12 | A-Kinase Anchoring Protein 12 | 40 |
| LIPN | Lipase Family Member N | 36 |
| PAK1 | P21 (RAC1) Activated Kinase 1 | 47 |
| BNIP3 | BCL2 Interacting Protein 3 | 42 |
| PFDN4 | Prefoldin Subunit 4 | 39 |
| ZNF845 | Zinc Finger Protein 845 | 32 |
| LDLR-AS1 | LDLR-AS1 | 6 |
| AOPEP | Aminopeptidase O (Putative) | 29 |
| SEMA6D | Semaphorin 6D | 39 |
| ASIP | Agouti Signaling Protein | 37 |
| BCL2A1 | BCL2 Related Protein A1 | 41 |
| PSMD9 | Proteasome 26S Subunit, Non-ATPase 9 | 43 |
| MAST4 | Microtubule Associated Serine/Threonine Kinase Family Member 4 | 40 |
| SYNCRIP | Synaptotagmin Binding Cytoplasmic RNA Interacting Protein | 38 |
| RNF215 | Ring Finger Protein 215 | 30 |
| MED13 | Mediator Complex Subunit 13 | 39 |
| RLN1 | Relaxin 1 | 35 |
| BRAP | BRCA1 Associated Protein | 41 |
| OFCC1 | Orofacial Cleft 1 Candidate 1 | 32 |
| F13B | Coagulation Factor XIII B Chain | 40 |
| RGS6 | Regulator Of G Protein Signaling 6 | 40 |
| SLC4A7 | Solute Carrier Family 4 Member 7 | 42 |
| S1PR2 | Sphingosine-1-Phosphate Receptor 2 | 45 |
| GTF3A | General Transcription Factor IIIA | 36 |
| RPL10A | Ribosomal Protein L10a | 41 |
| EGFLAM | EGF Like, Fibronectin Type III And Laminin G Domains | 37 |
| CLCA1 | Chloride Channel Accessory 1 | 40 |
| MAPKAP1 | MAPK Associated Protein 1 | 43 |
| RASIP1 | Ras Interacting Protein 1 | 36 |
| VSNL1 | Visinin Like 1 | 41 |
| HES1 | Hes Family BHLH Transcription Factor 1 | 43 |
| ADAM9 | ADAM Metallopeptidase Domain 9 | 47 |
| GPAA1 | Glycosylphosphatidylinositol Anchor Attachment 1 | 42 |
| SNX19 | Sorting Nexin 19 | 36 |
| MEAF6 | MYST/Esa1 Associated Factor 6 | 35 |
| GOT1 | Glutamic-Oxaloacetic Transaminase 1 | 47 |
| ZNF180 | Zinc Finger Protein 180 | 37 |
| ZNF202 | Zinc Finger Protein 202 | 39 |
| SLCO1B3 | Solute Carrier Organic Anion Transporter Family Member 1B3 | 44 |
| GPR108 | G Protein-Coupled Receptor 108 | 36 |
| GIMAP4 | GTPase, IMAP Family Member 4 | 38 |
| PSMD1 | Proteasome 26S Subunit, Non-ATPase 1 | 40 |
| PTPRF | Protein Tyrosine Phosphatase Receptor Type F | 50 |
| MARCKS | Myristoylated Alanine Rich Protein Kinase C Substrate | 39 |
| G6PC2 | Glucose-6-Phosphatase Catalytic Subunit 2 | 38 |
| C12orf43 | Chromosome 12 Open Reading Frame 43 | 33 |
| MLIP | Muscular LMNA Interacting Protein | 33 |
| PIGR | Polymeric Immunoglobulin Receptor | 41 |
| CSPG4 | Chondroitin Sulfate Proteoglycan 4 | 43 |
| MTUS2 | Microtubule Associated Scaffold Protein 2 | 34 |
| RIT2 | Ras Like Without CAAX 2 | 39 |
| IPO5 | Importin 5 | 39 |
| KLF10 | Kruppel Like Factor 10 | 42 |
| HTR1D | 5-Hydroxytryptamine Receptor 1D | 45 |
| NPPA-AS1 | NPPA Antisense RNA 1 | 16 |
| CRTC2 | CREB Regulated Transcription Coactivator 2 | 42 |
| SPC24 | SPC24 Component Of NDC80 Kinetochore Complex | 33 |
| CAPN7 | Calpain 7 | 40 |
| LPIN3 | Lipin 3 | 37 |
| SIRPA | Signal Regulatory Protein Alpha | 43 |
| TUB | TUB Bipartite Transcription Factor | 43 |
| MIR363 | MicroRNA 363 | 15 |
| VEZF1 | Vascular Endothelial Zinc Finger 1 | 35 |
| CCHCR1 | Coiled-Coil Alpha-Helical Rod Protein 1 | 38 |
| AZU1 | Azurocidin 1 | 39 |
| ATP6V1B2 | ATPase H+ Transporting V1 Subunit B2 | 46 |
| MAP1LC3A | Microtubule Associated Protein 1 Light Chain 3 Alpha | 43 |
| VAMP8 | Vesicle Associated Membrane Protein 8 | 41 |
| VSIR | V-Set Immunoregulatory Receptor | 32 |
| ZGPAT | Zinc Finger CCCH-Type And G-Patch Domain Containing | 37 |
| SEC24B | SEC24 Homolog B, COPII Coat Complex Component | 40 |
| DMXL2 | Dmx Like 2 | 36 |
| LGR6 | Leucine Rich Repeat Containing G Protein-Coupled Receptor 6 | 41 |
| APOBEC2 | Apolipoprotein B MRNA Editing Enzyme Catalytic Subunit 2 | 38 |
| CEACAM3 | CEA Cell Adhesion Molecule 3 | 40 |
| SELENOP | Selenoprotein P | 31 |
| SVEP1 | Sushi, Von Willebrand Factor Type A, EGF And Pentraxin Domain Containing 1 | 36 |
| RRS1 | Ribosome Biogenesis Regulator 1 Homolog | 34 |
| TARBP1 | TAR (HIV-1) RNA Binding Protein 1 | 39 |
| TSC22D3 | TSC22 Domain Family Member 3 | 40 |
| IL1RAP | Interleukin 1 Receptor Accessory Protein | 43 |
| ATP5MC1 | ATP Synthase Membrane Subunit C Locus 1 | 29 |
| HDLBP | High Density Lipoprotein Binding Protein | 40 |
| MSRA | Methionine Sulfoxide Reductase A | 42 |
| ANKRD55 | Ankyrin Repeat Domain 55 | 33 |
| ABI3BP | ABI Family Member 3 Binding Protein | 36 |
| EBF1 | EBF Transcription Factor 1 | 41 |
| POC5 | POC5 Centriolar Protein | 33 |
| PRDX6 | Peroxiredoxin 6 | 46 |
| MED9 | Mediator Complex Subunit 9 | 34 |
| LYZL1 | Lysozyme Like 1 | 37 |
| HNRNPUL1 | Heterogeneous Nuclear Ribonucleoprotein U Like 1 | 37 |
| NEXMIF | Neurite Extension And Migration Factor | 27 |
| GIPR | Gastric Inhibitory Polypeptide Receptor | 44 |
| DYDC2 | DPY30 Domain Containing 2 | 33 |
| SEMA4D | Semaphorin 4D | 44 |
| SIK2 | Salt Inducible Kinase 2 | 45 |
| DTNB | Dystrobrevin Beta | 39 |
| PAPLN | Papilin, Proteoglycan Like Sulfated Glycoprotein | 38 |
| SLC15A4 | Solute Carrier Family 15 Member 4 | 39 |
| C1orf210 | Chromosome 1 Open Reading Frame 210 | 30 |
| TMEM258 | Transmembrane Protein 258 | 31 |
| TCP1 | T-Complex 1 | 41 |
| C11orf58 | Chromosome 11 Open Reading Frame 58 | 35 |
| ALKBH8 | AlkB Homolog 8, TRNA Methyltransferase | 37 |
| SMCR5 | Smith-Magenis Syndrome Chromosome Region, Candidate 5 | 19 |
| MAP1S | Microtubule Associated Protein 1S | 35 |
| H4C3 | H4 Clustered Histone 3 | 31 |
| DSCAML1 | DS Cell Adhesion Molecule Like 1 | 39 |
| HDGF | Heparin Binding Growth Factor | 40 |
| WEE1 | WEE1 G2 Checkpoint Kinase | 46 |
| LEPROT | Leptin Receptor Overlapping Transcript | 33 |
| SNRPD2 | Small Nuclear Ribonucleoprotein D2 Polypeptide | 40 |
| TSBP1 | Testis Expressed Basic Protein 1 | 23 |
| HPSE2 | Heparanase 2 (Inactive) | 41 |
| SLC2A12 | Solute Carrier Family 2 Member 12 | 40 |
| IAH1 | Isoamyl Acetate Hydrolyzing Esterase 1 (Putative) | 33 |
| USP16 | Ubiquitin Specific Peptidase 16 | 41 |
| MIR345 | MicroRNA 345 | 19 |
| HEXD | Hexosaminidase D | 26 |
| GLCCI1 | Glucocorticoid Induced 1 | 37 |
| E2F2 | E2F Transcription Factor 2 | 42 |
| SGIP1 | SH3GL Interacting Endocytic Adaptor 1 | 36 |
| SRFBP1 | Serum Response Factor Binding Protein 1 | 32 |
| FAM223A | Family With Sequence Similarity 223 Member A | 14 |
| DYDC1 | DPY30 Domain Containing 1 | 35 |
| ASH2L | ASH2 Like, Histone Lysine Methyltransferase Complex Subunit | 41 |
| ADH7 | Alcohol Dehydrogenase 7 (Class IV), Mu Or Sigma Polypeptide | 44 |
| EPGN | Epithelial Mitogen | 34 |
| OSR1 | Odd-Skipped Related Transcription Factor 1 | 36 |
| TNIK | TRAF2 And NCK Interacting Kinase | 44 |
| KCNB2 | Potassium Voltage-Gated Channel Subfamily B Member 2 | 41 |
| SLC7A9 | Solute Carrier Family 7 Member 9 | 45 |
| EIF4A1 | Eukaryotic Translation Initiation Factor 4A1 | 44 |
| MEDAG | Mesenteric Estrogen Dependent Adipogenesis | 28 |
| FAM234B | Family With Sequence Similarity 234 Member B | 27 |
| CLEC4C | C-Type Lectin Domain Family 4 Member C | 32 |
| FNDC3B | Fibronectin Type III Domain Containing 3B | 36 |
| AP3D1 | Adaptor Related Protein Complex 3 Subunit Delta 1 | 40 |
| RSPO1 | R-Spondin 1 | 44 |
| NAALADL2 | N-Acetylated Alpha-Linked Acidic Dipeptidase Like 2 | 36 |
| NFIC | Nuclear Factor I C | 40 |
| PPIC | Peptidylprolyl Isomerase C | 41 |
| PRKCI | Protein Kinase C Iota | 48 |
| MCM10 | Minichromosome Maintenance 10 Replication Initiation Factor | 37 |
| MYO1D | Myosin ID | 40 |
| BAIAP2L1 | BAR/IMD Domain Containing Adaptor Protein 2 Like 1 | 37 |
| RPL9 | Ribosomal Protein L9 | 41 |
| POMP | Proteasome Maturation Protein | 41 |
| USP48 | Ubiquitin Specific Peptidase 48 | 39 |
| SORCS2 | Sortilin Related VPS10 Domain Containing Receptor 2 | 36 |
| COPS3 | COP9 Signalosome Subunit 3 | 39 |
| DMGDH | Dimethylglycine Dehydrogenase | 42 |
| DDX31 | DEAD-Box Helicase 31 | 37 |
| G3BP1 | G3BP Stress Granule Assembly Factor 1 | 40 |
| HAP1 | Huntingtin Associated Protein 1 | 39 |
| DRC3 | Dynein Regulatory Complex Subunit 3 | 28 |
| POLR3C | RNA Polymerase III Subunit C | 37 |
| NEUROG1 | Neurogenin 1 | 40 |
| ACAD11 | Acyl-CoA Dehydrogenase Family Member 11 | 36 |
| C16orf71 | Chromosome 16 Open Reading Frame 71 | 29 |
| CCN3 | Cellular Communication Network Factor 3 | 33 |
| XPO5 | Exportin 5 | 40 |
| C1GALT1 | Core 1 Synthase, Glycoprotein-N-Acetylgalactosamine 3-Beta-Galactosyltransferase 1 | 40 |
| SLC18A1 | Solute Carrier Family 18 Member A1 | 45 |
| GUK1 | Guanylate Kinase 1 | 42 |
| NCOA1 | Nuclear Receptor Coactivator 1 | 44 |
| QKI | QKI, KH Domain Containing RNA Binding | 43 |
| IRF2BP2 | Interferon Regulatory Factor 2 Binding Protein 2 | 36 |
| SCARA3 | Scavenger Receptor Class A Member 3 | 36 |
| CSNK1A1L | Casein Kinase 1 Alpha 1 Like | 36 |
| COL15A1 | Collagen Type XV Alpha 1 Chain | 38 |
| DEPTOR | DEP Domain Containing MTOR Interacting Protein | 40 |
| SF3A1 | Splicing Factor 3a Subunit 1 | 39 |
| GPN1 | GPN-Loop GTPase 1 | 37 |
| SPRY4-AS1 | SPRY4 Antisense RNA 1 | 13 |
| LMCD1 | LIM And Cysteine Rich Domains 1 | 39 |
| SVIL | Supervillin | 39 |
| MIR101-1 | MicroRNA 101-1 | 18 |
| RECK | Reversion Inducing Cysteine Rich Protein With Kazal Motifs | 40 |
| PDZK1 | PDZ Domain Containing 1 | 40 |
| EYA3 | EYA Transcriptional Coactivator And Phosphatase 3 | 37 |
| DNAH2 | Dynein Axonemal Heavy Chain 2 | 35 |
| CHMP4B | Charged Multivesicular Body Protein 4B | 41 |
| GIMAP2 | GTPase, IMAP Family Member 2 | 35 |
| GPAM | Glycerol-3-Phosphate Acyltransferase, Mitochondrial | 43 |
| IER3 | Immediate Early Response 3 | 38 |
| FAM219B | Family With Sequence Similarity 219 Member B | 30 |
| FERMT3 | Fermitin Family Member 3 | 43 |
| OXR1 | Oxidation Resistance 1 | 40 |
| C1D | C1D Nuclear Receptor Corepressor | 37 |
| EML5 | EMAP Like 5 | 34 |
| ALKBH5 | AlkB Homolog 5, RNA Demethylase | 33 |
| TACC1 | Transforming Acidic Coiled-Coil Containing Protein 1 | 40 |
| PI4KB | Phosphatidylinositol 4-Kinase Beta | 45 |
| EMSY | EMSY Transcriptional Repressor, BRCA2 Interacting | 31 |
| UBE2Z | Ubiquitin Conjugating Enzyme E2 Z | 41 |
| MYH15 | Myosin Heavy Chain 15 | 38 |
| MAML2 | Mastermind Like Transcriptional Coactivator 2 | 37 |
| TATDN1 | TatD DNase Domain Containing 1 | 35 |
| E2F4 | E2F Transcription Factor 4 | 45 |
| TENT5A | Terminal Nucleotidyltransferase 5A | 29 |
| MAPKAPK5 | MAPK Activated Protein Kinase 5 | 44 |
| GRIK5 | Glutamate Ionotropic Receptor Kainate Type Subunit 5 | 43 |
| NT5C1B | 5'-Nucleotidase, Cytosolic IB | 37 |
| MIR92A2 | MicroRNA 92a-2 | 16 |
| SPDEF | SAM Pointed Domain Containing ETS Transcription Factor | 39 |
| ACYP2 | Acylphosphatase 2 | 40 |
| ACSL1 | Acyl-CoA Synthetase Long Chain Family Member 1 | 44 |
| SESN2 | Sestrin 2 | 39 |
| INSIG2 | Insulin Induced Gene 2 | 40 |
| SLC10A7 | Solute Carrier Family 10 Member 7 | 38 |
| ENSG00000272379 |  | 6 |
| STRIP1 | Striatin Interacting Protein 1 | 33 |
| HSPA1L | Heat Shock Protein Family A (Hsp70) Member 1 Like | 43 |
| SDC4 | Syndecan 4 | 44 |
| PRMT1 | Protein Arginine Methyltransferase 1 | 48 |
| HAS1 | Hyaluronan Synthase 1 | 39 |
| DEFA1 | Defensin Alpha 1 | 40 |
| RAB31 | RAB31, Member RAS Oncogene Family | 40 |
| ACTR2 | Actin Related Protein 2 | 43 |
| NPY1R | Neuropeptide Y Receptor Y1 | 45 |
| LRGUK | Leucine Rich Repeats And Guanylate Kinase Domain Containing | 35 |
| C4B_2 | Complement Component 4B (Chido Blood Group), Copy 2 | 16 |
| PRKD2 | Protein Kinase D2 | 45 |
| ARHGEF16 | Rho Guanine Nucleotide Exchange Factor 16 | 39 |
| DRAM2 | DNA Damage Regulated Autophagy Modulator 2 | 39 |
| TMOD4 | Tropomodulin 4 | 37 |
| TENT4A | Terminal Nucleotidyltransferase 4A | 30 |
| GADD45A | Growth Arrest And DNA Damage Inducible Alpha | 44 |
| S100A11 | S100 Calcium Binding Protein A11 | 41 |
| DHX38 | DEAH-Box Helicase 38 | 43 |
| EPHA1 | EPH Receptor A1 | 47 |
| TSPAN2 | Tetraspanin 2 | 37 |
| MLEC | Malectin | 37 |
| SLC27A4 | Solute Carrier Family 27 Member 4 | 45 |
| SNORD94 | Small Nucleolar RNA, C/D Box 94 | 16 |
| MIR615 | MicroRNA 615 | 20 |
| MIR129-2 | MicroRNA 129-2 | 18 |
| HSF2 | Heat Shock Transcription Factor 2 | 43 |
| DTX2 | Deltex E3 Ubiquitin Ligase 2 | 39 |
| EGFL7 | EGF Like Domain Multiple 7 | 39 |
| HORMAD1 | HORMA Domain Containing 1 | 36 |
| IL34 | Interleukin 34 | 39 |
| MRPL12 | Mitochondrial Ribosomal Protein L12 | 38 |
| TRA | T Cell Receptor Alpha Locus | 17 |
| ELF5 | E74 Like ETS Transcription Factor 5 | 37 |
| TSR3 | TSR3 Ribosome Maturation Factor | 32 |
| HAL | Histidine Ammonia-Lyase | 43 |
| ARHGEF12 | Rho Guanine Nucleotide Exchange Factor 12 | 43 |
| RSPH6A | Radial Spoke Head 6 Homolog A | 31 |
| SNX17 | Sorting Nexin 17 | 37 |
| F11-AS1 | F11 Antisense RNA 1 | 12 |
| TMEM163 | Transmembrane Protein 163 | 33 |
| STEAP4 | STEAP4 Metalloreductase | 40 |
| PRRX2 | Paired Related Homeobox 2 | 33 |
| EBI3 | Epstein-Barr Virus Induced 3 | 39 |
| ANKS1A | Ankyrin Repeat And Sterile Alpha Motif Domain Containing 1A | 36 |
| DIO3 | Iodothyronine Deiodinase 3 | 40 |
| PTGES | Prostaglandin E Synthase | 41 |
| SENP3 | SUMO Specific Peptidase 3 | 40 |
| HTR7 | 5-Hydroxytryptamine Receptor 7 | 47 |
| NCAN | Neurocan | 41 |
| CARMIL1 | Capping Protein Regulator And Myosin 1 Linker 1 | 28 |
| ATRAID | All-Trans Retinoic Acid Induced Differentiation Factor | 35 |
| SEMA5B | Semaphorin 5B | 39 |
| CORO1C | Coronin 1C | 39 |
| HIF1AN | Hypoxia Inducible Factor 1 Subunit Alpha Inhibitor | 44 |
| ENSG00000236013 |  | 6 |
| PTGFR | Prostaglandin F Receptor | 44 |
| TRDMT1 | TRNA Aspartic Acid Methyltransferase 1 | 39 |
| GSTO2 | Glutathione S-Transferase Omega 2 | 43 |
| MORF4L1 | Mortality Factor 4 Like 1 | 41 |
| SLC1A5 | Solute Carrier Family 1 Member 5 | 43 |
| MT2A | Metallothionein 2A | 42 |
| SURF4 | Surfeit 4 | 38 |
| IDI1 | Isopentenyl-Diphosphate Delta Isomerase 1 | 44 |
| SETD1B | SET Domain Containing 1B, Histone Lysine Methyltransferase | 35 |
| RRP1B | Ribosomal RNA Processing 1B | 35 |
| FARP2 | FERM, ARH/RhoGEF And Pleckstrin Domain Protein 2 | 40 |
| ACAT2 | Acetyl-CoA Acetyltransferase 2 | 45 |
| TRIB3 | Tribbles Pseudokinase 3 | 43 |
| IMPA1 | Inositol Monophosphatase 1 | 48 |
| SYT14 | Synaptotagmin 14 | 37 |
| GBP1 | Guanylate Binding Protein 1 | 41 |
| ZNF664 | Zinc Finger Protein 664 | 35 |
| TBC1D7-LOC100130357 | TBC1D7-LOC100130357 Readthrough | 16 |
| BSN | Bassoon Presynaptic Cytomatrix Protein | 35 |
| TTLL6 | Tubulin Tyrosine Ligase Like 6 | 36 |
| MTHFD2 | Methylenetetrahydrofolate Dehydrogenase (NADP+ Dependent) 2, Methenyltetrahydrofolate Cyclohydrolase | 43 |
| AZGP1 | Alpha-2-Glycoprotein 1, Zinc-Binding | 43 |
| PIGF | Phosphatidylinositol Glycan Anchor Biosynthesis Class F | 37 |
| C1QTNF9 | C1q And TNF Related 9 | 33 |
| BYSL | Bystin Like | 38 |
| SELENOS | Selenoprotein S | 31 |
| ZHX2 | Zinc Fingers And Homeoboxes 2 | 37 |
| PEAR1 | Platelet Endothelial Aggregation Receptor 1 | 36 |
| LUM | Lumican | 41 |
| GALNT1 | Polypeptide N-Acetylgalactosaminyltransferase 1 | 41 |
| USP4 | Ubiquitin Specific Peptidase 4 | 43 |
| SAA2 | Serum Amyloid A2 | 36 |
| PGLYRP1 | Peptidoglycan Recognition Protein 1 | 40 |
| SPHK1 | Sphingosine Kinase 1 | 47 |
| CYFIP1 | Cytoplasmic FMR1 Interacting Protein 1 | 41 |
| INMT | Indolethylamine N-Methyltransferase | 41 |
| NEURL1 | Neuralized E3 Ubiquitin Protein Ligase 1 | 36 |
| HIPK2 | Homeodomain Interacting Protein Kinase 2 | 44 |
| TAS2R38 | Taste 2 Receptor Member 38 | 36 |
| KRT74 | Keratin 74 | 37 |
| KIAA0319 | KIAA0319 | 39 |
| FHII | Hyperaldosteronism, Familial, Type II | 2 |
| GALNT13 | Polypeptide N-Acetylgalactosaminyltransferase 13 | 40 |
| EFNB3 | Ephrin B3 | 42 |
| MIR323A | MicroRNA 323a | 16 |
| ITGA10 | Integrin Subunit Alpha 10 | 39 |
| AKR1C3 | Aldo-Keto Reductase Family 1 Member C3 | 47 |
| MGLL | Monoglyceride Lipase | 45 |
| MSRB1 | Methionine Sulfoxide Reductase B1 | 40 |
| IFNL3 | Interferon Lambda 3 | 35 |
| PLD2 | Phospholipase D2 | 47 |
| PDS5B | PDS5 Cohesin Associated Factor B | 35 |
| LAG3 | Lymphocyte Activating 3 | 37 |
| ATP5F1B | ATP Synthase F1 Subunit Beta | 33 |
| CLCA2 | Chloride Channel Accessory 2 | 41 |
| GRHL1 | Grainyhead Like Transcription Factor 1 | 35 |
| TAF11 | TATA-Box Binding Protein Associated Factor 11 | 39 |
| SEZ6L | Seizure Related 6 Homolog Like | 38 |
| BAG2 | BAG Cochaperone 2 | 40 |
| PLXNA4 | Plexin A4 | 38 |
| PIK3R3 | Phosphoinositide-3-Kinase Regulatory Subunit 3 | 43 |
| INSIG1 | Insulin Induced Gene 1 | 39 |
| NETO1 | Neuropilin And Tolloid Like 1 | 36 |
| USP1 | Ubiquitin Specific Peptidase 1 | 44 |
| H2AZ1 | H2A.Z Variant Histone 1 | 33 |
| CARM1 | Coactivator Associated Arginine Methyltransferase 1 | 47 |
| LGMN | Legumain | 42 |
| SLC17A1 | Solute Carrier Family 17 Member 1 | 39 |
| CHL1 | Cell Adhesion Molecule L1 Like | 39 |
| STARD3 | StAR Related Lipid Transfer Domain Containing 3 | 39 |
| SOWAHB | Sosondowah Ankyrin Repeat Domain Family Member B | 29 |
| TRMT10A | TRNA Methyltransferase 10A | 36 |
| TSPAN9 | Tetraspanin 9 | 39 |
| TNFAIP6 | TNF Alpha Induced Protein 6 | 40 |
| NCOA2 | Nuclear Receptor Coactivator 2 | 44 |
| GPIHBP1 | Glycosylphosphatidylinositol Anchored High Density Lipoprotein Binding Protein 1 | 35 |
| CCDC97 | Coiled-Coil Domain Containing 97 | 32 |
| HOMER1 | Homer Scaffold Protein 1 | 40 |
| RSL24D1 | Ribosomal L24 Domain Containing 1 | 37 |
| LRRC17 | Leucine Rich Repeat Containing 17 | 34 |
| MTF1 | Metal Regulatory Transcription Factor 1 | 41 |
| ACTR3 | Actin Related Protein 3 | 42 |
| ZC3HC1 | Zinc Finger C3HC-Type Containing 1 | 37 |
| UFSP2 | UFM1 Specific Peptidase 2 | 40 |
| ZNF45 | Zinc Finger Protein 45 | 36 |
| WDR33 | WD Repeat Domain 33 | 37 |
| CD180 | CD180 Molecule | 39 |
| AS3MT | Arsenite Methyltransferase | 40 |
| ZNF526 | Zinc Finger Protein 526 | 35 |
| LAP3 | Leucine Aminopeptidase 3 | 43 |
| DPT | Dermatopontin | 37 |
| BCAR3 | BCAR3 Adaptor Protein, NSP Family Member | 39 |
| UCN3 | Urocortin 3 | 37 |
| MIR500A | MicroRNA 500a | 16 |
| PKNOX1 | PBX/Knotted 1 Homeobox 1 | 40 |
| QARS1 | Glutaminyl-TRNA Synthetase 1 | 26 |
| POLR1F | RNA Polymerase I Subunit F | 28 |
| ATF3 | Activating Transcription Factor 3 | 44 |
| MBOAT7 | Membrane Bound O-Acyltransferase Domain Containing 7 | 40 |
| RBPMS2 | RNA Binding Protein, MRNA Processing Factor 2 | 35 |
| NXPH4 | Neurexophilin 4 | 35 |
| ZFAND6 | Zinc Finger AN1-Type Containing 6 | 36 |
| SLC17A3 | Solute Carrier Family 17 Member 3 | 40 |
| TCEA3 | Transcription Elongation Factor A3 | 36 |
| CARMIL3 | Capping Protein Regulator And Myosin 1 Linker 3 | 25 |
| SNF8 | SNF8 Subunit Of ESCRT-II | 38 |
| MICAL3 | Microtubule Associated Monooxygenase, Calponin And LIM Domain Containing 3 | 35 |
| TMEM184C | Transmembrane Protein 184C | 35 |
| PRMT8 | Protein Arginine Methyltransferase 8 | 37 |
| TMEFF2 | Transmembrane Protein With EGF Like And Two Follistatin Like Domains 2 | 39 |
| WNT8B | Wnt Family Member 8B | 41 |
| BTC | Betacellulin | 44 |
| L3MBTL3 | L3MBTL Histone Methyl-Lysine Binding Protein 3 | 36 |
| LGR5 | Leucine Rich Repeat Containing G Protein-Coupled Receptor 5 | 43 |
| IL15RA | Interleukin 15 Receptor Subunit Alpha | 41 |
| INTS4 | Integrator Complex Subunit 4 | 36 |
| UNC119B | Unc-119 Lipid Binding Chaperone B | 36 |
| FOSL1 | FOS Like 1, AP-1 Transcription Factor Subunit | 44 |
| FER | FER Tyrosine Kinase | 47 |
| PHC1 | Polyhomeotic Homolog 1 | 40 |
| SLC35G5 | Solute Carrier Family 35 Member G5 | 31 |
| SRSF1 | Serine And Arginine Rich Splicing Factor 1 | 41 |
| ENSG00000254851 |  | 4 |
| CD300LF | CD300 Molecule Like Family Member F | 39 |
| LMF1 | Lipase Maturation Factor 1 | 39 |
| CAMK1D | Calcium/Calmodulin Dependent Protein Kinase ID | 41 |
| COPS8 | COP9 Signalosome Subunit 8 | 37 |
| TOX3 | TOX High Mobility Group Box Family Member 3 | 37 |
| PHYHIP | Phytanoyl-CoA 2-Hydroxylase Interacting Protein | 35 |
| ANO6 | Anoctamin 6 | 39 |
| ADAM1A | ADAM Metallopeptidase Domain 1A (Pseudogene) | 13 |
| SDF4 | Stromal Cell Derived Factor 4 | 37 |
| HHIPL1 | HHIP Like 1 | 35 |
| MIR125B2 | MicroRNA 125b-2 | 21 |
| BFSP1 | Beaded Filament Structural Protein 1 | 38 |
| ADAL | Adenosine Deaminase Like | 34 |
| DENND1A | DENN Domain Containing 1A | 39 |
| GCN1 | GCN1 Activator Of EIF2AK4 | 28 |
| SLC22A11 | Solute Carrier Family 22 Member 11 | 40 |
| NUMB | NUMB Endocytic Adaptor Protein | 47 |
| MIR128-1 | MicroRNA 128-1 | 19 |
| NRP2 | Neuropilin 2 | 43 |
| VPS25 | Vacuolar Protein Sorting 25 Homolog | 39 |
| CDK5RAP3 | CDK5 Regulatory Subunit Associated Protein 3 | 38 |
| LOC105372273 | Uncharacterized LOC105372273 | 8 |
| POLK | DNA Polymerase Kappa | 41 |
| RHBDF2 | Rhomboid 5 Homolog 2 | 41 |
| FRK | Fyn Related Src Family Tyrosine Kinase | 44 |
| UCN2 | Urocortin 2 | 33 |
| PXN | Paxillin | 45 |
| ARHGEF18 | Rho/Rac Guanine Nucleotide Exchange Factor 18 | 43 |
| ZFAT | Zinc Finger And AT-Hook Domain Containing | 36 |
| PPT2 | Palmitoyl-Protein Thioesterase 2 | 38 |
| ANKLE2 | Ankyrin Repeat And LEM Domain Containing 2 | 39 |
| RNF43 | Ring Finger Protein 43 | 37 |
| RBM6 | RNA Binding Motif Protein 6 | 36 |
| B4GALT5 | Beta-1,4-Galactosyltransferase 5 | 39 |
| ADAMTS7P3 | ADAMTS7 Pseudogene 3 | 7 |
| GLS2 | Glutaminase 2 | 41 |
| DUSP2 | Dual Specificity Phosphatase 2 | 41 |
| FRMD5 | FERM Domain Containing 5 | 36 |
| HSP90AB1 | Heat Shock Protein 90 Alpha Family Class B Member 1 | 45 |
| FNDC1 | Fibronectin Type III Domain Containing 1 | 33 |
| ANAPC5 | Anaphase Promoting Complex Subunit 5 | 38 |
| PDIA2 | Protein Disulfide Isomerase Family A Member 2 | 38 |
| HMMR | Hyaluronan Mediated Motility Receptor | 42 |
| PARG | Poly(ADP-Ribose) Glycohydrolase | 39 |
| PKN2 | Protein Kinase N2 | 45 |
| HSPA2 | Heat Shock Protein Family A (Hsp70) Member 2 | 43 |
| LGALS7 | Galectin 7 | 37 |
| INTS5 | Integrator Complex Subunit 5 | 34 |
| TDRKH | Tudor And KH Domain Containing | 38 |
| MIR487B | MicroRNA 487b | 17 |
| RTN3 | Reticulon 3 | 41 |
| PMAIP1 | Phorbol-12-Myristate-13-Acetate-Induced Protein 1 | 39 |
| ZNF283 | Zinc Finger Protein 283 | 32 |
| IL1F10 | Interleukin 1 Family Member 10 | 39 |
| HES5 | Hes Family BHLH Transcription Factor 5 | 34 |
| EXOSC4 | Exosome Component 4 | 37 |
| IFIT2 | Interferon Induced Protein With Tetratricopeptide Repeats 2 | 39 |
| PIP5K1A | Phosphatidylinositol-4-Phosphate 5-Kinase Type 1 Alpha | 44 |
| MYO1F | Myosin IF | 39 |
| KCTD10 | Potassium Channel Tetramerization Domain Containing 10 | 36 |
| MIR136 | MicroRNA 136 | 19 |
| PLCH2 | Phospholipase C Eta 2 | 39 |
| NLRC5 | NLR Family CARD Domain Containing 5 | 39 |
| CPN1 | Carboxypeptidase N Subunit 1 | 43 |
| MNS1 | Meiosis Specific Nuclear Structural 1 | 35 |
| THSD7B | Thrombospondin Type 1 Domain Containing 7B | 34 |
| KCNA3 | Potassium Voltage-Gated Channel Subfamily A Member 3 | 44 |
| URB2 | URB2 Ribosome Biogenesis Homolog | 33 |
| ALDH1B1 | Aldehyde Dehydrogenase 1 Family Member B1 | 44 |
| ZNF222 | Zinc Finger Protein 222 | 32 |
| RUFY4 | RUN And FYVE Domain Containing 4 | 31 |
| CDC25C | Cell Division Cycle 25C | 48 |
| TBL3 | Transducin Beta Like 3 | 39 |
| MIR24-2 | MicroRNA 24-2 | 18 |
| NFKBIB | NFKB Inhibitor Beta | 41 |
| RECQL5 | RecQ Like Helicase 5 | 39 |
| SLC22A7 | Solute Carrier Family 22 Member 7 | 41 |
| FABP7 | Fatty Acid Binding Protein 7 | 43 |
| KCNH7 | Potassium Voltage-Gated Channel Subfamily H Member 7 | 43 |
| MIR3936HG | MIR3936 Host Gene | 11 |
| KCNK5 | Potassium Two Pore Domain Channel Subfamily K Member 5 | 37 |
| CLSTN2 | Calsyntenin 2 | 39 |
| ID1 | Inhibitor Of DNA Binding 1, HLH Protein | 43 |
| DHRS3 | Dehydrogenase/Reductase 3 | 43 |
| RAMP1 | Receptor Activity Modifying Protein 1 | 43 |
| GRM8 | Glutamate Metabotropic Receptor 8 | 45 |
| NR4A3 | Nuclear Receptor Subfamily 4 Group A Member 3 | 45 |
| VWDE | Von Willebrand Factor D And EGF Domains | 31 |
| ZEB2-AS1 | ZEB2 Antisense RNA 1 | 19 |
| GPRC6A | G Protein-Coupled Receptor Class C Group 6 Member A | 39 |
| CLEC4A | C-Type Lectin Domain Family 4 Member A | 37 |
| PABPC4 | Poly(A) Binding Protein Cytoplasmic 4 | 41 |
| GPR55 | G Protein-Coupled Receptor 55 | 40 |
| GLTP | Glycolipid Transfer Protein | 37 |
| CCDC63 | Coiled-Coil Domain Containing 63 | 34 |
| MTMR10 | Myotubularin Related Protein 10 | 33 |
| MYZAP | Myocardial Zonula Adherens Protein | 31 |
| SULT1A2 | Sulfotransferase Family 1A Member 2 | 42 |
| PLEKHG2 | Pleckstrin Homology And RhoGEF Domain Containing G2 | 38 |
| SFRP1 | Secreted Frizzled Related Protein 1 | 43 |
| CFAP91 | Cilia And Flagella Associated Protein 91 | 27 |
| METTL16 | Methyltransferase Like 16 | 29 |
| SNAPC4 | Small Nuclear RNA Activating Complex Polypeptide 4 | 36 |
| CSGALNACT2 | Chondroitin Sulfate N-Acetylgalactosaminyltransferase 2 | 41 |
| MALRD1 | MAM And LDL Receptor Class A Domain Containing 1 | 28 |
| TMEM242 | Transmembrane Protein 242 | 32 |
| MIR654 | MicroRNA 654 | 17 |
| GPLD1 | Glycosylphosphatidylinositol Specific Phospholipase D1 | 41 |
| ANKS1B | Ankyrin Repeat And Sterile Alpha Motif Domain Containing 1B | 39 |
| IL24 | Interleukin 24 | 42 |
| LIPH | Lipase H | 42 |
| MTCH1 | Mitochondrial Carrier 1 | 37 |
| MPP7 | Membrane Palmitoylated Protein 7 | 36 |
| GORASP1 | Golgi Reassembly Stacking Protein 1 | 40 |
| NAAA | N-Acylethanolamine Acid Amidase | 40 |
| SNX13 | Sorting Nexin 13 | 36 |
| SELENOI | Selenoprotein I | 31 |
| SUGP1 | SURP And G-Patch Domain Containing 1 | 35 |
| CD84 | CD84 Molecule | 41 |
| PDIA3 | Protein Disulfide Isomerase Family A Member 3 | 44 |
| DACH1 | Dachshund Family Transcription Factor 1 | 39 |
| MAP3K14 | Mitogen-Activated Protein Kinase Kinase Kinase 14 | 44 |
| PCSK7 | Proprotein Convertase Subtilisin/Kexin Type 7 | 43 |
| MIR99B | MicroRNA 99b | 18 |
| TNFSF14 | TNF Superfamily Member 14 | 41 |
| MN298114-202 |  | 4 |
| KCNK1 | Potassium Two Pore Domain Channel Subfamily K Member 1 | 43 |
| TBC1D1 | TBC1 Domain Family Member 1 | 40 |
| C8orf49 | Chromosome 8 Open Reading Frame 49 | 20 |
| CIC | Capicua Transcriptional Repressor | 41 |
| RMC1 | Regulator Of MON1-CCZ1 | 24 |
| PAMR1 | Peptidase Domain Containing Associated With Muscle Regeneration 1 | 36 |
| MANSC1 | MANSC Domain Containing 1 | 34 |
| PRUNE2 | Prune Homolog 2 With BCH Domain | 37 |
| IQCH | IQ Motif Containing H | 33 |
| RAB3IL1 | RAB3A Interacting Protein Like 1 | 37 |
| SEC23IP | SEC23 Interacting Protein | 39 |
| ZFP36L1 | ZFP36 Ring Finger Protein Like 1 | 43 |
| CCL23 | C-C Motif Chemokine Ligand 23 | 34 |
| ABCF2 | ATP Binding Cassette Subfamily F Member 2 | 39 |
| FAM167A | Family With Sequence Similarity 167 Member A | 35 |
| TEX41 | Testis Expressed 41 | 16 |
| ID3 | Inhibitor Of DNA Binding 3, HLH Protein | 41 |
| TMEM38A | Transmembrane Protein 38A | 36 |
| RAMP3 | Receptor Activity Modifying Protein 3 | 40 |
| TIPARP | TCDD Inducible Poly(ADP-Ribose) Polymerase | 34 |
| ALCAM | Activated Leukocyte Cell Adhesion Molecule | 42 |
| ELOVL2 | ELOVL Fatty Acid Elongase 2 | 40 |
| ZNF844 | Zinc Finger Protein 844 | 31 |
| TOM1 | Target Of Myb1 Membrane Trafficking Protein | 41 |
| GREM2 | Gremlin 2, DAN Family BMP Antagonist | 40 |
| MAP3K11 | Mitogen-Activated Protein Kinase Kinase Kinase 11 | 46 |
| NME6 | NME/NM23 Nucleoside Diphosphate Kinase 6 | 40 |
| WTIP | WT1 Interacting Protein | 35 |
| PLEKHA7 | Pleckstrin Homology Domain Containing A7 | 38 |
| MUSTN1 | Musculoskeletal, Embryonic Nuclear Protein 1 | 33 |
| DUSP5 | Dual Specificity Phosphatase 5 | 41 |
| NUMA1 | Nuclear Mitotic Apparatus Protein 1 | 43 |
| INKA1 | Inka Box Actin Regulator 1 | 25 |
| FREM3 | FRAS1 Related Extracellular Matrix 3 | 31 |
| ENSG00000260773 |  | 6 |
| SGSM2 | Small G Protein Signaling Modulator 2 | 36 |
| LINC00888 | Long Intergenic Non-Protein Coding RNA 888 | 13 |
| LRG1 | Leucine Rich Alpha-2-Glycoprotein 1 | 39 |
| MAP4 | Microtubule Associated Protein 4 | 41 |
| JUND | JunD Proto-Oncogene, AP-1 Transcription Factor Subunit | 41 |
| NAA25 | N-Alpha-Acetyltransferase 25, NatB Auxiliary Subunit | 35 |
| EPB41L3 | Erythrocyte Membrane Protein Band 4.1 Like 3 | 41 |
| KCNK15 | Potassium Two Pore Domain Channel Subfamily K Member 15 | 38 |
| NUB1 | Negative Regulator Of Ubiquitin Like Proteins 1 | 37 |
| PNOC | Prepronociceptin | 37 |
| C1orf56 | Chromosome 1 Open Reading Frame 56 | 32 |
| GRIN3A | Glutamate Ionotropic Receptor NMDA Type Subunit 3A | 37 |
| CRISPLD1 | Cysteine Rich Secretory Protein LCCL Domain Containing 1 | 34 |
| MIR769 | MicroRNA 769 | 16 |
| GNPDA2 | Glucosamine-6-Phosphate Deaminase 2 | 39 |
| LZTS1 | Leucine Zipper Tumor Suppressor 1 | 38 |
| RNF214 | Ring Finger Protein 214 | 33 |
| SORBS3 | Sorbin And SH3 Domain Containing 3 | 40 |
| LILRB2 | Leukocyte Immunoglobulin Like Receptor B2 | 40 |
| PREB | Prolactin Regulatory Element Binding | 39 |
| PRPF38B | Pre-MRNA Processing Factor 38B | 33 |
| ZBTB8OS | Zinc Finger And BTB Domain Containing 8 Opposite Strand | 36 |
| FOXQ1 | Forkhead Box Q1 | 33 |
| MTERF1 | Mitochondrial Transcription Termination Factor 1 | 34 |
| GMDS | GDP-Mannose 4,6-Dehydratase | 44 |
| RIPK3 | Receptor Interacting Serine/Threonine Kinase 3 | 43 |
| RETNLB | Resistin Like Beta | 35 |
| CORT | Cortistatin | 36 |
| CALCB | Calcitonin Related Polypeptide Beta | 37 |
| MIR212 | MicroRNA 212 | 19 |
| TAF3 | TATA-Box Binding Protein Associated Factor 3 | 39 |
| SOX7 | SRY-Box Transcription Factor 7 | 35 |
| ADAMTS8 | ADAM Metallopeptidase With Thrombospondin Type 1 Motif 8 | 39 |
| TBC1D23 | TBC1 Domain Family Member 23 | 36 |
| SUPT5H | SPT5 Homolog, DSIF Elongation Factor Subunit | 39 |
| GSTM2 | Glutathione S-Transferase Mu 2 | 41 |
| LEKR1 | Leucine, Glutamate And Lysine Rich 1 | 32 |
| ANKRD6 | Ankyrin Repeat Domain 6 | 37 |
| NUDT1 | Nudix Hydrolase 1 | 43 |
| RSPO3 | R-Spondin 3 | 38 |
| DENND5A | DENN Domain Containing 5A | 37 |
| INTS10 | Integrator Complex Subunit 10 | 35 |
| SMC5 | Structural Maintenance Of Chromosomes 5 | 36 |
| ARID3A | AT-Rich Interaction Domain 3A | 39 |
| DACT3 | Dishevelled Binding Antagonist Of Beta Catenin 3 | 35 |
| TMEM59 | Transmembrane Protein 59 | 39 |
| SLC16A9 | Solute Carrier Family 16 Member 9 | 39 |
| TTLL7 | Tubulin Tyrosine Ligase Like 7 | 35 |
| CREB3 | CAMP Responsive Element Binding Protein 3 | 38 |
| MAP3K6 | Mitogen-Activated Protein Kinase Kinase Kinase 6 | 44 |
| ZNF385D | Zinc Finger Protein 385D | 32 |
| ZNF592 | Zinc Finger Protein 592 | 36 |
| TSHZ2 | Teashirt Zinc Finger Homeobox 2 | 38 |
| EFNA1 | Ephrin A1 | 43 |
| EREG | Epiregulin | 41 |
| TPSAB1 | Tryptase Alpha/Beta 1 | 43 |
| MPP3 | Membrane Palmitoylated Protein 3 | 38 |
| CTAGE1 | Cutaneous T Cell Lymphoma-Associated Antigen 1 | 33 |
| BEND6 | BEN Domain Containing 6 | 31 |
| XAB2 | XPA Binding Protein 2 | 38 |
| CMIP | C-Maf Inducing Protein | 35 |
| MIR503 | MicroRNA 503 | 17 |
| GOLGB1 | Golgin B1 | 37 |
| SLC6A20 | Solute Carrier Family 6 Member 20 | 41 |
| APOO | Apolipoprotein O | 34 |
| ENSG00000258634 |  | 6 |
| AURKAIP1 | Aurora Kinase A Interacting Protein 1 | 37 |
| GSC2 | Goosecoid Homeobox 2 | 32 |
| KLHL29 | Kelch Like Family Member 29 | 32 |
| FSTL4 | Follistatin Like 4 | 35 |
| BDP1 | B Double Prime 1, Subunit Of RNA Polymerase III Transcription Initiation Factor IIIB | 37 |
| MIR484 | MicroRNA 484 | 18 |
| SMCR2 | Smith-Magenis Syndrome Chromosome Region, Candidate 2 | 13 |
| MCUR1 | Mitochondrial Calcium Uniporter Regulator 1 | 35 |
| LMO2 | LIM Domain Only 2 | 42 |
| LRIG1 | Leucine Rich Repeats And Immunoglobulin Like Domains 1 | 41 |
| MINPP1 | Multiple Inositol-Polyphosphate Phosphatase 1 | 45 |
| SLC30A1 | Solute Carrier Family 30 Member 1 | 40 |
| FBXO46 | F-Box Protein 46 | 29 |
| GAS2 | Growth Arrest Specific 2 | 39 |
| BFAR | Bifunctional Apoptosis Regulator | 37 |
| AMFR | Autocrine Motility Factor Receptor | 43 |
| APPBP2 | Amyloid Beta Precursor Protein Binding Protein 2 | 37 |
| SLC30A3 | Solute Carrier Family 30 Member 3 | 39 |
| TDRD10 | Tudor Domain Containing 10 | 29 |
| EMILIN3 | Elastin Microfibril Interfacer 3 | 31 |
| PRMT5 | Protein Arginine Methyltransferase 5 | 42 |
| CNTROB | Centrobin, Centriole Duplication And Spindle Assembly Protein | 36 |
| ERVFRD-1 | Endogenous Retrovirus Group FRD Member 1, Envelope | 32 |
| MIR30C1 | MicroRNA 30c-1 | 21 |
| P2RY11 | Purinergic Receptor P2Y11 | 44 |
| EXOC3L2 | Exocyst Complex Component 3 Like 2 | 33 |
| C1orf167 | Chromosome 1 Open Reading Frame 167 | 27 |
| MIR128-2 | MicroRNA 128-2 | 20 |
| IMPDH2 | Inosine Monophosphate Dehydrogenase 2 | 47 |
| UBASH3B | Ubiquitin Associated And SH3 Domain Containing B | 40 |
| SESN1 | Sestrin 1 | 39 |
| TRIM24 | Tripartite Motif Containing 24 | 43 |
| PPFIA2 | PTPRF Interacting Protein Alpha 2 | 37 |
| ENSG00000250155 |  | 8 |
| NME3 | NME/NM23 Nucleoside Diphosphate Kinase 3 | 43 |
| EDIL3 | EGF Like Repeats And Discoidin Domains 3 | 40 |
| CSRP2 | Cysteine And Glycine Rich Protein 2 | 39 |
| FGD6 | FYVE, RhoGEF And PH Domain Containing 6 | 36 |
| GOLM2 | Golgi Membrane Protein 2 | 27 |
| NR2C2 | Nuclear Receptor Subfamily 2 Group C Member 2 | 43 |
| ENSA | Endosulfine Alpha | 41 |
| MIR181D | MicroRNA 181d | 17 |
| PARL | Presenilin Associated Rhomboid Like | 41 |
| MIR325 | MicroRNA 325 | 13 |
| MDN1 | Midasin AAA ATPase 1 | 37 |
| PARPBP | PARP1 Binding Protein | 32 |
| LPCAT3 | Lysophosphatidylcholine Acyltransferase 3 | 36 |
| ATP10A | ATPase Phospholipid Transporting 10A (Putative) | 39 |
| CDC42BPA | CDC42 Binding Protein Kinase Alpha | 40 |
| LECT2 | Leukocyte Cell Derived Chemotaxin 2 | 36 |
| THAP5 | THAP Domain Containing 5 | 36 |
| SPPL3 | Signal Peptide Peptidase Like 3 | 35 |
| NBL1 | NBL1, DAN Family BMP Antagonist | 39 |
| CLEC3B | C-Type Lectin Domain Family 3 Member B | 40 |
| SSNA1 | SS Nuclear Autoantigen 1 | 35 |
| MARK4 | Microtubule Affinity Regulating Kinase 4 | 44 |
| BSX | Brain Specific Homeobox | 32 |
| ST3GAL1 | ST3 Beta-Galactoside Alpha-2,3-Sialyltransferase 1 | 43 |
| ZNF441 | Zinc Finger Protein 441 | 29 |
| CARD16 | Caspase Recruitment Domain Family Member 16 | 36 |
| PLEKHG1 | Pleckstrin Homology And RhoGEF Domain Containing G1 | 33 |
| UHRF1BP1 | UHRF1 Binding Protein 1 | 35 |
| RSU1 | Ras Suppressor Protein 1 | 40 |
| SHC4 | SHC Adaptor Protein 4 | 37 |
| DPH2 | Diphthamide Biosynthesis 2 | 38 |
| TRERF1 | Transcriptional Regulating Factor 1 | 37 |
| FAM193A | Family With Sequence Similarity 193 Member A | 31 |
| TRIM72 | Tripartite Motif Containing 72 | 36 |
| GNA12 | G Protein Subunit Alpha 12 | 41 |
| KIR2DL3 | Killer Cell Immunoglobulin Like Receptor, Two Ig Domains And Long Cytoplasmic Tail 3 | 36 |
| BCAP29 | B Cell Receptor Associated Protein 29 | 36 |
| CREG1 | Cellular Repressor Of E1A Stimulated Genes 1 | 38 |
| MXD3 | MAX Dimerization Protein 3 | 34 |
| CALU | Calumenin | 40 |
| RRM1 | Ribonucleotide Reductase Catalytic Subunit M1 | 47 |
| PRORP | Protein Only RNase P Catalytic Subunit | 27 |
| ATG4C | Autophagy Related 4C Cysteine Peptidase | 40 |
| ZC2HC1C | Zinc Finger C2HC-Type Containing 1C | 32 |
| FGD5 | FYVE, RhoGEF And PH Domain Containing 5 | 38 |
| CTHRC1 | Collagen Triple Helix Repeat Containing 1 | 41 |
| SLCO2B1 | Solute Carrier Organic Anion Transporter Family Member 2B1 | 44 |
| ELL | Elongation Factor For RNA Polymerase II | 39 |
| GRID2IP | Grid2 Interacting Protein | 31 |
| PIAS3 | Protein Inhibitor Of Activated STAT 3 | 41 |
| ZNF491 | Zinc Finger Protein 491 | 26 |
| NPY2R | Neuropeptide Y Receptor Y2 | 43 |
| SPNS2 | Sphingolipid Transporter 2 | 35 |
| CRTC3 | CREB Regulated Transcription Coactivator 3 | 39 |
| BLID | BH3-Like Motif Containing, Cell Death Inducer | 29 |
| RNF139 | Ring Finger Protein 139 | 39 |
| ZFP36L2 | ZFP36 Ring Finger Protein Like 2 | 37 |
| GIMAP1 | GTPase, IMAP Family Member 1 | 33 |
| ABCC5 | ATP Binding Cassette Subfamily C Member 5 | 43 |
| SLCO3A1 | Solute Carrier Organic Anion Transporter Family Member 3A1 | 40 |
| ROPN1L | Rhophilin Associated Tail Protein 1 Like | 36 |
| ENSG00000256879 |  | 7 |
| PIF1 | PIF1 5'-To-3' DNA Helicase | 36 |
| CCDC3 | Coiled-Coil Domain Containing 3 | 31 |
| DOCK1 | Dedicator Of Cytokinesis 1 | 44 |
| IL20 | Interleukin 20 | 39 |
| JMJD6 | Jumonji Domain Containing 6, Arginine Demethylase And Lysine Hydroxylase | 42 |
| LILRB3 | Leukocyte Immunoglobulin Like Receptor B3 | 39 |
| C1QTNF5 | C1q And TNF Related 5 | 39 |
| PARP2 | Poly(ADP-Ribose) Polymerase 2 | 46 |
| ING1 | Inhibitor Of Growth Family Member 1 | 43 |
| PDE1C | Phosphodiesterase 1C | 45 |
| NMUR1 | Neuromedin U Receptor 1 | 41 |
| FGD3 | FYVE, RhoGEF And PH Domain Containing 3 | 39 |
| OVCA2 | OVCA2 Serine Hydrolase Domain Containing | 35 |
| ZNF404 | Zinc Finger Protein 404 | 32 |
| NAB1 | NGFI-A Binding Protein 1 | 39 |
| GPR22 | G Protein-Coupled Receptor 22 | 35 |
| CCDC89 | Coiled-Coil Domain Containing 89 | 30 |
| ALPI | Alkaline Phosphatase, Intestinal | 43 |
| RALYL | RALY RNA Binding Protein Like | 35 |
| MSI2 | Musashi RNA Binding Protein 2 | 41 |
| WDR76 | WD Repeat Domain 76 | 33 |
| PPP1R1A | Protein Phosphatase 1 Regulatory Inhibitor Subunit 1A | 41 |
| CAPN9 | Calpain 9 | 42 |
| HCG27 | HLA Complex Group 27 | 21 |
| MAP6 | Microtubule Associated Protein 6 | 35 |
| UIMC1 | Ubiquitin Interaction Motif Containing 1 | 41 |
| PSORS1C3 | Psoriasis Susceptibility 1 Candidate 3 | 21 |
| GALR1 | Galanin Receptor 1 | 44 |
| PROKR1 | Prokineticin Receptor 1 | 37 |
| UBXN4 | UBX Domain Protein 4 | 36 |
| OARD1 | O-Acyl-ADP-Ribose Deacylase 1 | 33 |
| AP2A2 | Adaptor Related Protein Complex 2 Subunit Alpha 2 | 37 |
| VTI1A | Vesicle Transport Through Interaction With T-SNAREs 1A | 39 |
| QTRT1 | Queuine TRNA-Ribosyltransferase Catalytic Subunit 1 | 38 |
| GRK6 | G Protein-Coupled Receptor Kinase 6 | 45 |
| COL6A5 | Collagen Type VI Alpha 5 Chain | 33 |
| FHL3 | Four And A Half LIM Domains 3 | 41 |
| MIR217 | MicroRNA 217 | 18 |
| SSR3 | Signal Sequence Receptor Subunit 3 | 36 |
| AKR1C4 | Aldo-Keto Reductase Family 1 Member C4 | 47 |
| CDC123 | Cell Division Cycle 123 | 35 |
| PPHLN1 | Periphilin 1 | 37 |
| DNLZ | DNL-Type Zinc Finger | 29 |
| PLCB2 | Phospholipase C Beta 2 | 46 |
| DDRGK1 | DDRGK Domain Containing 1 | 37 |
| MAP9 | Microtubule Associated Protein 9 | 33 |
| RF00017-5492 |  | 4 |
| EVL | Enah/Vasp-Like | 38 |
| LOC111365141 | NOS2 5' Regulatory Region | 1 |
| LOC101928725 | Uncharacterized LOC101928725 | 10 |
| FPR2 | Formyl Peptide Receptor 2 | 45 |
| CCR5AS | CCR5 Antisense RNA | 10 |
| MIR625 | MicroRNA 625 | 14 |
| ACOT1 | Acyl-CoA Thioesterase 1 | 36 |
| HSD17B1 | Hydroxysteroid 17-Beta Dehydrogenase 1 | 43 |
| SMYD4 | SET And MYND Domain Containing 4 | 37 |
| MCF2L | MCF.2 Cell Line Derived Transforming Sequence Like | 42 |
| ARHGEF6 | Rac/Cdc42 Guanine Nucleotide Exchange Factor 6 | 41 |
| TFCP2L1 | Transcription Factor CP2 Like 1 | 36 |
| MIR98 | MicroRNA 98 | 17 |
| RLN2 | Relaxin 2 | 37 |
| PDCD6IP | Programmed Cell Death 6 Interacting Protein | 42 |
| DFFB | DNA Fragmentation Factor Subunit Beta | 43 |
| SERPINA2 | Serpin Family A Member 2 (Gene/Pseudogene) | 27 |
| RPS27P19 | Ribosomal Protein S27 Pseudogene 19 | 5 |
| PI16 | Peptidase Inhibitor 16 | 36 |
| FBXO3 | F-Box Protein 3 | 37 |
| ORM1 | Orosomucoid 1 | 39 |
| KIAA1586 | KIAA1586 | 33 |
| ZNF788P | Zinc Finger Family Member 788, Pseudogene | 16 |
| CBLL1 | Cbl Proto-Oncogene Like 1 | 37 |
| ASIC1 | Acid Sensing Ion Channel Subunit 1 | 43 |
| CILP2 | Cartilage Intermediate Layer Protein 2 | 35 |
| EGR3 | Early Growth Response 3 | 39 |
| FUT3 | Fucosyltransferase 3 (Lewis Blood Group) | 43 |
| MARCHF8 | Membrane Associated Ring-CH-Type Finger 8 | 28 |
| STMND1 | Stathmin Domain Containing 1 | 26 |
| EDEM2 | ER Degradation Enhancing Alpha-Mannosidase Like Protein 2 | 40 |
| CA10 | Carbonic Anhydrase 10 | 39 |
| TUSC1 | Tumor Suppressor Candidate 1 | 28 |
| MAD2L1 | Mitotic Arrest Deficient 2 Like 1 | 45 |
| ENSG00000244716 |  | 4 |
| PIGB | Phosphatidylinositol Glycan Anchor Biosynthesis Class B | 40 |
| RF00017-1272 |  | 4 |
| SCD5 | Stearoyl-CoA Desaturase 5 | 40 |
| PLCB3 | Phospholipase C Beta 3 | 47 |
| BLMH | Bleomycin Hydrolase | 43 |
| GABPB2 | GA Binding Protein Transcription Factor Subunit Beta 2 | 35 |
| FGD5-AS1 | FGD5 Antisense RNA 1 | 14 |
| TNFRSF14 | TNF Receptor Superfamily Member 14 | 43 |
| IGLL1 | Immunoglobulin Lambda Like Polypeptide 1 | 43 |
| EFNA5 | Ephrin A5 | 43 |
| DGKQ | Diacylglycerol Kinase Theta | 41 |
| NEURL4 | Neuralized E3 Ubiquitin Protein Ligase 4 | 32 |
| DHX58 | DExH-Box Helicase 58 | 39 |
| CCDC159 | Coiled-Coil Domain Containing 159 | 25 |
| SLC7A6 | Solute Carrier Family 7 Member 6 | 43 |
| THUMPD2 | THUMP Domain Containing 2 | 32 |
| MIR374A | MicroRNA 374a | 16 |
| SRPX | Sushi Repeat Containing Protein X-Linked | 39 |
| CARF | Calcium Responsive Transcription Factor | 33 |
| FAM117B | Family With Sequence Similarity 117 Member B | 32 |
| ZNF224 | Zinc Finger Protein 224 | 38 |
| MACO1 | Macoilin 1 | 27 |
| ENSG00000259202 |  | 7 |
| TEAD4 | TEA Domain Transcription Factor 4 | 41 |
| CD200 | CD200 Molecule | 39 |
| HLA-S | Major Histocompatibility Complex, Class I, S (Pseudogene) | 7 |
| CORO6 | Coronin 6 | 34 |
| GSTA4 | Glutathione S-Transferase Alpha 4 | 43 |
| C5orf15 | Chromosome 5 Open Reading Frame 15 | 33 |
| NUCB2 | Nucleobindin 2 | 38 |
| PLEKHA1 | Pleckstrin Homology Domain Containing A1 | 41 |
| RBM5 | RNA Binding Motif Protein 5 | 39 |
| CLK3 | CDC Like Kinase 3 | 43 |
| SHISA9 | Shisa Family Member 9 | 33 |
| SERPINB8 | Serpin Family B Member 8 | 43 |
| F2RL2 | Coagulation Factor II Thrombin Receptor Like 2 | 41 |
| PRM2 | Protamine 2 | 34 |
| CHN2 | Chimerin 2 | 41 |
| SNX29 | Sorting Nexin 29 | 33 |
| COBLL1 | Cordon-Bleu WH2 Repeat Protein Like 1 | 37 |
| ATAD5 | ATPase Family AAA Domain Containing 5 | 33 |
| BLOC1S2 | Biogenesis Of Lysosomal Organelles Complex 1 Subunit 2 | 35 |
| PLEKHH1 | Pleckstrin Homology, MyTH4 And FERM Domain Containing H1 | 34 |
| SUSD2 | Sushi Domain Containing 2 | 36 |
| KCTD11 | Potassium Channel Tetramerization Domain Containing 11 | 33 |
| PLXDC2 | Plexin Domain Containing 2 | 37 |
| F11R | F11 Receptor | 43 |
| SPTSSB | Serine Palmitoyltransferase Small Subunit B | 32 |
| TEX2 | Testis Expressed 2 | 32 |
| SLCO1A2 | Solute Carrier Organic Anion Transporter Family Member 1A2 | 39 |
| GNL2 | G Protein Nucleolar 2 | 37 |
| ZNF823 | Zinc Finger Protein 823 | 32 |
| HTR1F | 5-Hydroxytryptamine Receptor 1F | 44 |
| EHBP1L1 | EH Domain Binding Protein 1 Like 1 | 32 |
| ALOX15B | Arachidonate 15-Lipoxygenase Type B | 41 |
| SNX5 | Sorting Nexin 5 | 43 |
| ENSG00000267114 |  | 7 |
| AP1G2 | Adaptor Related Protein Complex 1 Subunit Gamma 2 | 37 |
| OBP2B | Odorant Binding Protein 2B | 31 |
| SERPINB9 | Serpin Family B Member 9 | 39 |
| MS4A6A | Membrane Spanning 4-Domains A6A | 37 |
| SURF6 | Surfeit 6 | 36 |
| DSCR10 | Down Syndrome Critical Region 10 | 20 |
| ARHGEF3 | Rho Guanine Nucleotide Exchange Factor 3 | 41 |
| REEP5 | Receptor Accessory Protein 5 | 39 |
| LATS1 | Large Tumor Suppressor Kinase 1 | 44 |
| CCL8 | C-C Motif Chemokine Ligand 8 | 39 |
| BCAS3 | BCAS3 Microtubule Associated Cell Migration Factor | 36 |
| RNF130 | Ring Finger Protein 130 | 37 |
| ACTRT2 | Actin Related Protein T2 | 35 |
| STARD7 | StAR Related Lipid Transfer Domain Containing 7 | 37 |
| TMEM214 | Transmembrane Protein 214 | 35 |
| IFIT5 | Interferon Induced Protein With Tetratricopeptide Repeats 5 | 33 |
| ARID3B | AT-Rich Interaction Domain 3B | 38 |
| CYP2C18 | Cytochrome P450 Family 2 Subfamily C Member 18 | 44 |
| ZNF223 | Zinc Finger Protein 223 | 32 |
| ZFYVE9 | Zinc Finger FYVE-Type Containing 9 | 41 |
| MATN2 | Matrilin 2 | 39 |
| GSTM4 | Glutathione S-Transferase Mu 4 | 41 |
| PPID | Peptidylprolyl Isomerase D | 43 |
| NOC4L | Nucleolar Complex Associated 4 Homolog | 33 |
| JAKMIP1 | Janus Kinase And Microtubule Interacting Protein 1 | 37 |
| OLFM4 | Olfactomedin 4 | 37 |
| ANXA4 | Annexin A4 | 44 |
| PIWIL2 | Piwi Like RNA-Mediated Gene Silencing 2 | 37 |
| IFI30 | IFI30 Lysosomal Thiol Reductase | 39 |
| IPMK | Inositol Polyphosphate Multikinase | 39 |
| UBXN2B | UBX Domain Protein 2B | 37 |
| KLF14 | Kruppel Like Factor 14 | 32 |
| ENSG00000242798 |  | 6 |
| CYP20A1 | Cytochrome P450 Family 20 Subfamily A Member 1 | 36 |
| MMP16 | Matrix Metallopeptidase 16 | 44 |
| NUCKS1 | Nuclear Casein Kinase And Cyclin Dependent Kinase Substrate 1 | 36 |
| EAF1 | ELL Associated Factor 1 | 36 |
| CEP128 | Centrosomal Protein 128 | 32 |
| MIR16-2 | MicroRNA 16-2 | 18 |
| FBXO33 | F-Box Protein 33 | 35 |
| HHIPL2 | HHIP Like 2 | 36 |
| KCNK13 | Potassium Two Pore Domain Channel Subfamily K Member 13 | 39 |
| MACIR | Macrophage Immunometabolism Regulator | 25 |
| NMU | Neuromedin U | 38 |
| C5orf38 | Chromosome 5 Open Reading Frame 38 | 31 |
| FOXB1 | Forkhead Box B1 | 36 |
| PSMD6 | Proteasome 26S Subunit, Non-ATPase 6 | 38 |
| HOXC13 | Homeobox C13 | 40 |
| TANK | TRAF Family Member Associated NFKB Activator | 43 |
| UHMK1 | U2AF Homology Motif Kinase 1 | 39 |
| RRM2 | Ribonucleotide Reductase Regulatory Subunit M2 | 48 |
| ACYP1 | Acylphosphatase 1 | 39 |
| UGT1A7 | UDP Glucuronosyltransferase Family 1 Member A7 | 39 |
| C3orf62 | Chromosome 3 Open Reading Frame 62 | 29 |
| ULK3 | Unc-51 Like Kinase 3 | 40 |
| MIR218-1 | MicroRNA 218-1 | 17 |
| ATF1 | Activating Transcription Factor 1 | 46 |
| E2F3 | E2F Transcription Factor 3 | 43 |
| LINC00841 | Long Intergenic Non-Protein Coding RNA 841 | 14 |
| PLPP1 | Phospholipid Phosphatase 1 | 33 |
| ZBTB33 | Zinc Finger And BTB Domain Containing 33 | 36 |
| GSTA1 | Glutathione S-Transferase Alpha 1 | 41 |
| ZNF507 | Zinc Finger Protein 507 | 34 |
| MCM7 | Minichromosome Maintenance Complex Component 7 | 44 |
| NYAP2 | Neuronal Tyrosine-Phosphorylated Phosphoinositide-3-Kinase Adaptor 2 | 32 |
| CPA3 | Carboxypeptidase A3 | 40 |
| RGPD8 | RANBP2 Like And GRIP Domain Containing 8 | 31 |
| GRB14 | Growth Factor Receptor Bound Protein 14 | 43 |
| C19orf38 | Chromosome 19 Open Reading Frame 38 | 27 |
| ZNF284 | Zinc Finger Protein 284 | 30 |
| DHX8 | DEAH-Box Helicase 8 | 37 |
| CISD1 | CDGSH Iron Sulfur Domain 1 | 38 |
| WFDC1 | WAP Four-Disulfide Core Domain 1 | 36 |
| EZH1 | Enhancer Of Zeste 1 Polycomb Repressive Complex 2 Subunit | 45 |
| COL16A1 | Collagen Type XVI Alpha 1 Chain | 39 |
| CDK18 | Cyclin Dependent Kinase 18 | 39 |
| TRIM69 | Tripartite Motif Containing 69 | 36 |
| ENSG00000284686 |  | 6 |
| LRTM1 | Leucine Rich Repeats And Transmembrane Domains 1 | 36 |
| SH3BP5 | SH3 Domain Binding Protein 5 | 40 |
| LTBR | Lymphotoxin Beta Receptor | 41 |
| MMP26 | Matrix Metallopeptidase 26 | 34 |
| TSPAN16 | Tetraspanin 16 | 32 |
| PSORS1C1 | Psoriasis Susceptibility 1 Candidate 1 | 28 |
| MALL | Mal, T Cell Differentiation Protein Like | 35 |
| ICA1L | Islet Cell Autoantigen 1 Like | 35 |
| NOL7 | Nucleolar Protein 7 | 34 |
| SLC14A2 | Solute Carrier Family 14 Member 2 | 41 |
| HEMGN | Hemogen | 35 |
| SMURF1 | SMAD Specific E3 Ubiquitin Protein Ligase 1 | 45 |
| RYK | Receptor Like Tyrosine Kinase | 41 |
| LILRB1 | Leukocyte Immunoglobulin Like Receptor B1 | 43 |
| ELK1 | ETS Transcription Factor ELK1 | 44 |
| RSRC2 | Arginine And Serine Rich Coiled-Coil 2 | 32 |
| IL17D | Interleukin 17D | 38 |
| IQCA1 | IQ Motif Containing With AAA Domain 1 | 34 |
| ANKRD22 | Ankyrin Repeat Domain 22 | 32 |
| CLEC5A | C-Type Lectin Domain Containing 5A | 34 |
| CMTR1 | Cap Methyltransferase 1 | 35 |
| TFPI2 | Tissue Factor Pathway Inhibitor 2 | 41 |
| SUPT3H | SPT3 Homolog, SAGA And STAGA Complex Component | 40 |
| ADGRE5 | Adhesion G Protein-Coupled Receptor E5 | 38 |
| MIR202 | MicroRNA 202 | 17 |
| ADSS2 | Adenylosuccinate Synthase 2 | 34 |
| POLR2D | RNA Polymerase II Subunit D | 41 |
| ARHGAP1 | Rho GTPase Activating Protein 1 | 43 |
| ARHGAP42 | Rho GTPase Activating Protein 42 | 36 |
| SLC23A2 | Solute Carrier Family 23 Member 2 | 42 |
| LCMT2 | Leucine Carboxyl Methyltransferase 2 | 37 |
| TOR2A | Torsin Family 2 Member A | 36 |
| DEGS2 | Delta 4-Desaturase, Sphingolipid 2 | 38 |
| NCF1C | Neutrophil Cytosolic Factor 1C Pseudogene | 21 |
| PAX8-AS1 | PAX8 Antisense RNA 1 | 16 |
| LOC107984189 | Uncharacterized Protein C10orf142-Like | 6 |
| LOC107984814 | Uncharacterized LOC107984814 | 6 |
| ANAPC1 | Anaphase Promoting Complex Subunit 1 | 40 |
| DDX25 | DEAD-Box Helicase 25 | 39 |
| ARHGEF26 | Rho Guanine Nucleotide Exchange Factor 26 | 36 |
| LOC100996842 | Uncharacterized LOC100996842 | 12 |
| STK38 | Serine/Threonine Kinase 38 | 43 |
| SNX1 | Sorting Nexin 1 | 41 |
| VWA7 | Von Willebrand Factor A Domain Containing 7 | 31 |
| UGT1A9 | UDP Glucuronosyltransferase Family 1 Member A9 | 43 |
| RANGAP1 | Ran GTPase Activating Protein 1 | 41 |
| NSUN4 | NOP2/Sun RNA Methyltransferase 4 | 39 |
| NID2 | Nidogen 2 | 37 |
| RNF20 | Ring Finger Protein 20 | 38 |
| TSPAN8 | Tetraspanin 8 | 39 |
| FGFBP2 | Fibroblast Growth Factor Binding Protein 2 | 33 |
| RANBP3L | RAN Binding Protein 3 Like | 32 |
| INTS11 | Integrator Complex Subunit 11 | 28 |
| SSH1 | Slingshot Protein Phosphatase 1 | 41 |
| PCOLCE | Procollagen C-Endopeptidase Enhancer | 39 |
| NRIP3 | Nuclear Receptor Interacting Protein 3 | 35 |
| GTF3C2 | General Transcription Factor IIIC Subunit 2 | 37 |
| TTC39B | Tetratricopeptide Repeat Domain 39B | 36 |
| HDGFL1 | HDGF Like 1 | 29 |
| PLEKHO2 | Pleckstrin Homology Domain Containing O2 | 35 |
| VAMP3 | Vesicle Associated Membrane Protein 3 | 41 |
| MS | Multiple Sclerosis | 4 |
| TTC29 | Tetratricopeptide Repeat Domain 29 | 34 |
| SCAMP5 | Secretory Carrier Membrane Protein 5 | 36 |
| MIR497 | MicroRNA 497 | 17 |
| ABCA8 | ATP Binding Cassette Subfamily A Member 8 | 39 |
| MYO3B | Myosin IIIB | 40 |
| ENSG00000253775 |  | 6 |
| PCNPP1 | PEST Containing Nuclear Protein Pseudogene 1 | 9 |
| ITM2A | Integral Membrane Protein 2A | 38 |
| CASTOR3 | CASTOR Family Member 3 | 24 |
| MSL2 | MSL Complex Subunit 2 | 33 |
| LYSMD4 | LysM Domain Containing 4 | 34 |
| SAMSN1 | SAM Domain, SH3 Domain And Nuclear Localization Signals 1 | 37 |
| DDX60L | DExD/H-Box 60 Like | 33 |
| PIAS4 | Protein Inhibitor Of Activated STAT 4 | 43 |
| SRGN | Serglycin | 38 |
| ZNF112 | Zinc Finger Protein 112 | 33 |
| GPR17 | G Protein-Coupled Receptor 17 | 40 |
| GIMAP6 | GTPase, IMAP Family Member 6 | 36 |
| SEC31B | SEC31 Homolog B, COPII Coat Complex Component | 37 |
| KNTC1 | Kinetochore Associated 1 | 36 |
| ANP32B | Acidic Nuclear Phosphoprotein 32 Family Member B | 37 |
| TDRD15 | Tudor Domain Containing 15 | 24 |
| C4BPB | Complement Component 4 Binding Protein Beta | 40 |
| AKTIP | AKT Interacting Protein | 39 |
| CWF19L2 | CWF19 Like Cell Cycle Control Factor 2 | 32 |
| CPM | Carboxypeptidase M | 42 |
| HGFAC | HGF Activator | 40 |
| SLC25A35 | Solute Carrier Family 25 Member 35 | 36 |
| ADGRL3 | Adhesion G Protein-Coupled Receptor L3 | 33 |
| POLN | DNA Polymerase Nu | 34 |
| STEAP1 | STEAP Family Member 1 | 40 |
| IGFALS | Insulin Like Growth Factor Binding Protein Acid Labile Subunit | 44 |
| SPECC1L-ADORA2A | SPECC1L-ADORA2A Readthrough (NMD Candidate) | 14 |
| GOLPH3L | Golgi Phosphoprotein 3 Like | 35 |
| THBS3 | Thrombospondin 3 | 41 |
| GABRG3 | Gamma-Aminobutyric Acid Type A Receptor Subunit Gamma3 | 42 |
| ANAPC4 | Anaphase Promoting Complex Subunit 4 | 35 |
| EHD3 | EH Domain Containing 3 | 37 |
| VPS41 | VPS41 Subunit Of HOPS Complex | 38 |
| LINC01589 | Long Intergenic Non-Protein Coding RNA 1589 | 13 |
| TRIM4 | Tripartite Motif Containing 4 | 32 |
| MIR501 | MicroRNA 501 | 16 |
| ZNF385B | Zinc Finger Protein 385B | 35 |
| MEGF6 | Multiple EGF Like Domains 6 | 32 |
| ENSG00000251405 |  | 6 |
| SNORD10 | Small Nucleolar RNA, C/D Box 10 | 14 |
| SHE | Src Homology 2 Domain Containing E | 34 |
| YIPF5 | Yip1 Domain Family Member 5 | 36 |
| GRID1 | Glutamate Ionotropic Receptor Delta Type Subunit 1 | 40 |
| PEBP4 | Phosphatidylethanolamine Binding Protein 4 | 37 |
| PCDH9 | Protocadherin 9 | 37 |
| SEMA4G | Semaphorin 4G | 39 |
| ENSG00000272501 |  | 8 |
| E2F6 | E2F Transcription Factor 6 | 40 |
| NPY5R | Neuropeptide Y Receptor Y5 | 42 |
| MIR411 | MicroRNA 411 | 14 |
| SEC16A | SEC16 Homolog A, Endoplasmic Reticulum Export Factor | 36 |
| ARL5B | ADP Ribosylation Factor Like GTPase 5B | 37 |
| FLG-AS1 | FLG Antisense RNA 1 | 16 |
| SLK | STE20 Like Kinase | 43 |
| PINX1 | PIN2 (TERF1) Interacting Telomerase Inhibitor 1 | 39 |
| NIPSNAP3B | Nipsnap Homolog 3B | 35 |
| ESYT3 | Extended Synaptotagmin 3 | 33 |
| MYH4 | Myosin Heavy Chain 4 | 39 |
| TTC32 | Tetratricopeptide Repeat Domain 32 | 32 |
| CCDC158 | Coiled-Coil Domain Containing 158 | 29 |
| RPRD2 | Regulation Of Nuclear Pre-MRNA Domain Containing 2 | 36 |
| APOL4 | Apolipoprotein L4 | 36 |
| MIR29B2 | MicroRNA 29b-2 | 18 |
| SNORA48 | Small Nucleolar RNA, H/ACA Box 48 | 15 |
| ENSG00000265749 |  | 8 |
| GRIK4 | Glutamate Ionotropic Receptor Kainate Type Subunit 4 | 43 |
| GSTM5 | Glutathione S-Transferase Mu 5 | 41 |
| ABHD2 | Abhydrolase Domain Containing 2, Acylglycerol Lipase | 37 |
| FOXS1 | Forkhead Box S1 | 33 |
| NCKAP5 | NCK Associated Protein 5 | 33 |
| SMAD5 | SMAD Family Member 5 | 41 |
| piR-51327 |  | 4 |
| lnc-FAM109A-1 |  | 4 |
| piR-56480-015 |  | 3 |
| piR-51449 |  | 3 |
| piR-50346 |  | 3 |
| piR-36455 |  | 3 |
| piR-38259 |  | 3 |
| piR-48007 |  | 3 |
| FBXO15 | F-Box Protein 15 | 37 |
| MAPK13 | Mitogen-Activated Protein Kinase 13 | 48 |
| LINC02881 | Long Intergenic Non-Protein Coding RNA 2881 | 14 |
| LINC-ROR | Long Intergenic Non-Protein Coding RNA, Regulator Of Reprogramming | 16 |
| LRRC2 | Leucine Rich Repeat Containing 2 | 36 |
| ACVR1B | Activin A Receptor Type 1B | 47 |
| ADAMTS14 | ADAM Metallopeptidase With Thrombospondin Type 1 Motif 14 | 37 |
| ZNF887P | Zinc Finger Protein 887, Pseudogene | 6 |
| ANKRD28 | Ankyrin Repeat Domain 28 | 35 |
| LIPC-AS1 | LIPC Antisense RNA 1 | 10 |
| KLK11 | Kallikrein Related Peptidase 11 | 41 |
| SLC8A2 | Solute Carrier Family 8 Member A2 | 40 |
| STXBP5 | Syntaxin Binding Protein 5 | 38 |
| SFRP5 | Secreted Frizzled Related Protein 5 | 39 |
| LINC01089 | Long Intergenic Non-Protein Coding RNA 1089 | 14 |
| DUXA | Double Homeobox A | 28 |
| DIO1 | Iodothyronine Deiodinase 1 | 40 |
| MAGEC2 | MAGE Family Member C2 | 36 |
| GTF3C2-AS1 | GTF3C2 Antisense RNA 1 | 13 |
| LINC00472 | Long Intergenic Non-Protein Coding RNA 472 | 22 |
| METRNL | Meteorin Like, Glial Cell Differentiation Regulator | 35 |
| SELENOM | Selenoprotein M | 27 |
| MAGI1 | Membrane Associated Guanylate Kinase, WW And PDZ Domain Containing 1 | 40 |
| TREH | Trehalase | 43 |
| GCOM1 | GRINL1A Complex Locus 1 | 29 |
| STAB2 | Stabilin 2 | 39 |
| UBE2D3P3 | Ubiquitin Conjugating Enzyme E2 D3 Pseudogene 3 | 8 |
| CXCL14 | C-X-C Motif Chemokine Ligand 14 | 39 |
| SLC44A5 | Solute Carrier Family 44 Member 5 | 33 |
| UGT1A4 | UDP Glucuronosyltransferase Family 1 Member A4 | 41 |
| IRAG1 | Inositol 1,4,5-Triphosphate Receptor Associated 1 | 29 |
| MIR26A2 | MicroRNA 26a-2 | 20 |
| INTS8 | Integrator Complex Subunit 8 | 36 |
| IGF2BP1 | Insulin Like Growth Factor 2 MRNA Binding Protein 1 | 40 |
| ENSG00000234268 |  | 4 |
| MIR454 | MicroRNA 454 | 18 |
| GIT1 | GIT ArfGAP 1 | 43 |
| STOML1 | Stomatin Like 1 | 36 |
| CNPY4 | Canopy FGF Signaling Regulator 4 | 34 |
| RNF139-AS1 | RNF139 Antisense RNA 1 (Head To Head) | 13 |
| CLEC11A | C-Type Lectin Domain Containing 11A | 39 |
| RPL18AP13 | Ribosomal Protein L18a Pseudogene 13 | 7 |
| WASHC5-AS1 | WASHC5 Antisense RNA 1 | 8 |
| P2RX6 | Purinergic Receptor P2X 6 | 40 |
| CPED1 | Cadherin Like And PC-Esterase Domain Containing 1 | 32 |
| SBSPON | Somatomedin B And Thrombospondin Type 1 Domain Containing | 30 |
| CXCL3 | C-X-C Motif Chemokine Ligand 3 | 39 |
| MFAP1 | Microfibril Associated Protein 1 | 36 |
| PRELID1 | PRELI Domain Containing 1 | 36 |
| ABCA2 | ATP Binding Cassette Subfamily A Member 2 | 41 |
| HMGB2 | High Mobility Group Box 2 | 42 |
| ZBTB14 | Zinc Finger And BTB Domain Containing 14 | 35 |
| VTA1 | Vesicle Trafficking 1 | 37 |
| GOLGA6L3 | Golgin A6 Family Like 3 | 20 |
| ENSG00000226849 |  | 7 |
| CDH9 | Cadherin 9 | 39 |
| TRHDE | Thyrotropin Releasing Hormone Degrading Enzyme | 37 |
| ENSG00000272657 |  | 6 |
| ADH5 | Alcohol Dehydrogenase 5 (Class III), Chi Polypeptide | 45 |
| MLXIP | MLX Interacting Protein | 33 |
| IFIT1B | Interferon Induced Protein With Tetratricopeptide Repeats 1B | 32 |
| KRTCAP3 | Keratinocyte Associated Protein 3 | 31 |
| ZNF225 | Zinc Finger Protein 225 | 32 |
| ENSG00000253106 |  | 6 |
| RPN1 | Ribophorin I | 42 |
| P2RY4 | Pyrimidinergic Receptor P2Y4 | 43 |
| PDLIM7 | PDZ And LIM Domain 7 | 40 |
| FAM13C | Family With Sequence Similarity 13 Member C | 32 |
| ZNF227 | Zinc Finger Protein 227 | 33 |
| CHST9 | Carbohydrate Sulfotransferase 9 | 36 |
| BCO1 | Beta-Carotene Oxygenase 1 | 36 |
| KRTCAP2 | Keratinocyte Associated Protein 2 | 33 |
| C10orf71 | Chromosome 10 Open Reading Frame 71 | 32 |
| ENSG00000264545 |  | 10 |
| ZNF235 | Zinc Finger Protein 235 | 31 |
| USP42 | Ubiquitin Specific Peptidase 42 | 37 |
| RASSF10 | Ras Association Domain Family Member 10 | 31 |
| UBE2G1 | Ubiquitin Conjugating Enzyme E2 G1 | 43 |
| CAP1 | Cyclase Associated Actin Cytoskeleton Regulatory Protein 1 | 41 |
| NME2 | NME/NM23 Nucleoside Diphosphate Kinase 2 | 46 |
| GTF2B | General Transcription Factor IIB | 42 |
| KCTD12 | Potassium Channel Tetramerization Domain Containing 12 | 35 |
| GRK3 | G Protein-Coupled Receptor Kinase 3 | 38 |
| ENSG00000214955 |  | 7 |
| lnc-JCAD-2 |  | 4 |
| KCNK10 | Potassium Two Pore Domain Channel Subfamily K Member 10 | 39 |
| DENND2B | DENN Domain Containing 2B | 28 |
| SLC29A4 | Solute Carrier Family 29 Member 4 | 42 |
| TSC22D1 | TSC22 Domain Family Member 1 | 40 |
| COL6A4P1 | Collagen Type VI Alpha 4 Pseudogene 1 | 12 |
| P2RY6 | Pyrimidinergic Receptor P2Y6 | 42 |
| ENSG00000253111 |  | 7 |
| RNF182 | Ring Finger Protein 182 | 35 |
| RPL7P8 | Ribosomal Protein L7 Pseudogene 8 | 8 |
| PAFAH1B2 | Platelet Activating Factor Acetylhydrolase 1b Catalytic Subunit 2 | 44 |
| MN298114-200 |  | 4 |
| ZNF300 | Zinc Finger Protein 300 | 36 |
| MAT2B | Methionine Adenosyltransferase 2B | 40 |
| PLA2G2D | Phospholipase A2 Group IID | 42 |
| TM7SF3 | Transmembrane 7 Superfamily Member 3 | 35 |
| OSBPL3 | Oxysterol Binding Protein Like 3 | 37 |
| AASDH | Aminoadipate-Semialdehyde Dehydrogenase | 36 |
| ZNF230 | Zinc Finger Protein 230 | 33 |
| MOGAT2 | Monoacylglycerol O-Acyltransferase 2 | 38 |
| CTR9 | CTR9 Homolog, Paf1/RNA Polymerase II Complex Component | 37 |
| NFKBIE | NFKB Inhibitor Epsilon | 39 |
| CD160 | CD160 Molecule | 39 |
| CCDC71L | Coiled-Coil Domain Containing 71 Like | 28 |
| HSALNG0099364 |  | 4 |
| SYT9 | Synaptotagmin 9 | 36 |
| CACFD1 | Calcium Channel Flower Domain Containing 1 | 33 |
| ENSG00000248734 |  | 6 |
| ACAA2 | Acetyl-CoA Acyltransferase 2 | 43 |
| ZNF558 | Zinc Finger Protein 558 | 35 |
| SNHG3 | Small Nucleolar RNA Host Gene 3 | 17 |
| DNAJC5B | DnaJ Heat Shock Protein Family (Hsp40) Member C5 Beta | 29 |
| MAS1 | MAS1 Proto-Oncogene, G Protein-Coupled Receptor | 40 |
| GUSBP4 | GUSB Pseudogene 4 | 16 |
| TINAGL1 | Tubulointerstitial Nephritis Antigen Like 1 | 39 |
| RRBP1 | Ribosome Binding Protein 1 | 39 |
| PPFIA1 | PTPRF Interacting Protein Alpha 1 | 36 |
| ZSCAN29 | Zinc Finger And SCAN Domain Containing 29 | 33 |
| HAPLN3 | Hyaluronan And Proteoglycan Link Protein 3 | 37 |
| UBE2Q2P1 | Ubiquitin Conjugating Enzyme E2 Q2 Pseudogene 1 | 14 |
| GTPBP10 | GTP Binding Protein 10 | 32 |
| ZNF226 | Zinc Finger Protein 226 | 32 |
| ISLR | Immunoglobulin Superfamily Containing Leucine Rich Repeat | 36 |
| XKR6 | XK Related 6 | 33 |
| FCRL6 | Fc Receptor Like 6 | 34 |
| CBLC | Cbl Proto-Oncogene C | 37 |
| KBTBD11 | Kelch Repeat And BTB Domain Containing 11 | 34 |
| ENSG00000262089 |  | 8 |
| NPFF | Neuropeptide FF-Amide Peptide Precursor | 35 |
| BMERB1 | BMERB Domain Containing 1 | 25 |
| ABCC10 | ATP Binding Cassette Subfamily C Member 10 | 40 |
| FRY | FRY Microtubule Binding Protein | 34 |
| ANKRD65 | Ankyrin Repeat Domain 65 | 28 |
| CDC25B | Cell Division Cycle 25B | 46 |
| CBWD2 | COBW Domain Containing 2 | 29 |
| CBR4 | Carbonyl Reductase 4 | 38 |
| LOC107987125 | Uncharacterized LOC107987125 | 5 |
| MIR493 | MicroRNA 493 | 17 |
| PHACTR4 | Phosphatase And Actin Regulator 4 | 32 |
| NONHSAG017238.2 |  | 3 |
| lnc-APOC1-1 |  | 4 |
| MN298678-088 |  | 4 |
| ST6GAL1 | ST6 Beta-Galactoside Alpha-2,6-Sialyltransferase 1 | 43 |
| SCML4 | Scm Polycomb Group Protein Like 4 | 35 |
| ZNF155 | Zinc Finger Protein 155 | 33 |
| UGT1A | UDP Glucuronosyltransferase Family 1 Member A Complex Locus | 9 |
| LARP6 | La Ribonucleoprotein 6, Translational Regulator | 33 |
| FHOD1 | Formin Homology 2 Domain Containing 1 | 37 |
| ZNF665 | Zinc Finger Protein 665 | 32 |
| CLEC14A | C-Type Lectin Domain Containing 14A | 34 |
| CGREF1 | Cell Growth Regulator With EF-Hand Domain 1 | 35 |
| CCNL1 | Cyclin L1 | 38 |
| PF4V1 | Platelet Factor 4 Variant 1 | 35 |
| KCNH5 | Potassium Voltage-Gated Channel Subfamily H Member 5 | 43 |
| TRIM22 | Tripartite Motif Containing 22 | 39 |
| IKZF2 | IKAROS Family Zinc Finger 2 | 39 |
| VAV2 | Vav Guanine Nucleotide Exchange Factor 2 | 43 |
| VAMP5 | Vesicle Associated Membrane Protein 5 | 39 |
| EVPL | Envoplakin | 35 |
| USP9Y | Ubiquitin Specific Peptidase 9 Y-Linked | 37 |
| MPST | Mercaptopyruvate Sulfurtransferase | 43 |
| CD74 | CD74 Molecule | 43 |
| EXOC6 | Exocyst Complex Component 6 | 39 |
| HSALNG0063781 |  | 4 |
| UGT1A10 | UDP Glucuronosyltransferase Family 1 Member A10 | 40 |
| H3-4 | H3.4 Histone | 32 |
| MIR7-1 | MicroRNA 7-1 | 18 |
| RF00017-8038 |  | 4 |
| IGSF5 | Immunoglobulin Superfamily Member 5 | 32 |
| RXFP2 | Relaxin Family Peptide Receptor 2 | 43 |
| ENSG00000236838 |  | 8 |
| ENSG00000232110 |  | 8 |
| LAMP3 | Lysosomal Associated Membrane Protein 3 | 39 |
| FGL1 | Fibrinogen Like 1 | 40 |
| XKR8 | XK Related 8 | 34 |
| S100A13 | S100 Calcium Binding Protein A13 | 37 |
| ADAD1 | Adenosine Deaminase Domain Containing 1 | 36 |
| NONHSAG039062.2 |  | 2 |
| ZDHHC4 | Zinc Finger DHHC-Type Palmitoyltransferase 4 | 36 |
| CRIP2 | Cysteine Rich Protein 2 | 36 |
| C4orf45 | Chromosome 4 Open Reading Frame 45 | 28 |
| THEMIS2 | Thymocyte Selection Associated Family Member 2 | 33 |
| RFLNA | Refilin A | 24 |
| MBD6 | Methyl-CpG Binding Domain Protein 6 | 35 |
| ENSG00000238221 |  | 7 |
| ZNF831 | Zinc Finger Protein 831 | 32 |
| C1orf54 | Chromosome 1 Open Reading Frame 54 | 28 |
| LRRC25 | Leucine Rich Repeat Containing 25 | 33 |
| ZNF444 | Zinc Finger Protein 444 | 33 |
| TRIP6 | Thyroid Hormone Receptor Interactor 6 | 39 |
| FAM114A1 | Family With Sequence Similarity 114 Member A1 | 33 |
| ZBTB2 | Zinc Finger And BTB Domain Containing 2 | 34 |
| YTHDC2 | YTH Domain Containing 2 | 35 |
| ABHD1 | Abhydrolase Domain Containing 1 | 32 |
| SHISA6 | Shisa Family Member 6 | 33 |
| TNIP2 | TNFAIP3 Interacting Protein 2 | 38 |
| COPS5 | COP9 Signalosome Subunit 5 | 42 |
| ABLIM1 | Actin Binding LIM Protein 1 | 40 |
| SENP1 | SUMO Specific Peptidase 1 | 42 |
| TXNDC5 | Thioredoxin Domain Containing 5 | 39 |
| CENPU | Centromere Protein U | 37 |
| lnc-SPC24-1 |  | 4 |
| MIR4673 | MicroRNA 4673 | 12 |
| ENSG00000236457 |  | 8 |
| NUMBL | NUMB Like Endocytic Adaptor Protein | 39 |
| RGS14 | Regulator Of G Protein Signaling 14 | 43 |
| REEP4 | Receptor Accessory Protein 4 | 36 |
| ZNF234 | Zinc Finger Protein 234 | 32 |
| NRG4 | Neuregulin 4 | 39 |
| ALOX12-AS1 | ALOX12 Antisense RNA 1 | 13 |
| CCDC157 | Coiled-Coil Domain Containing 157 | 30 |
| ZNF221 | Zinc Finger Protein 221 | 33 |
| LRRC18 | Leucine Rich Repeat Containing 18 | 31 |
| USP32P2 | Ubiquitin Specific Peptidase 32 Pseudogene 2 | 17 |
| ENSG00000255389 |  | 7 |
| SCGN | Secretagogin, EF-Hand Calcium Binding Protein | 38 |
| GIMAP7 | GTPase, IMAP Family Member 7 | 36 |
| STIN2-VNTR | Serotonin Transporter Intronic VNTR Enhancer | 1 |
| SLC17A2 | Solute Carrier Family 17 Member 2 | 37 |
| APOF | Apolipoprotein F | 37 |
| CCNE2 | Cyclin E2 | 41 |
| OSTF1 | Osteoclast Stimulating Factor 1 | 40 |
| DCTN5 | Dynactin Subunit 5 | 36 |
| NUBP2 | Nucleotide Binding Protein 2 | 39 |
| SYCP2L | Synaptonemal Complex Protein 2 Like | 32 |
| ELMOD3 | ELMO Domain Containing 3 | 38 |
| lnc-ZNF296-6 |  | 2 |
| NONHSAG026010.2 |  | 1 |
| USO1 | USO1 Vesicle Transport Factor | 38 |
| RAPH1 | Ras Association (RalGDS/AF-6) And Pleckstrin Homology Domains 1 | 36 |
| PREX1 | Phosphatidylinositol-3,4,5-Trisphosphate Dependent Rac Exchange Factor 1 | 43 |
| FASTK | Fas Activated Serine/Threonine Kinase | 39 |
| E2F8 | E2F Transcription Factor 8 | 35 |
| CLLU1 | Chronic Lymphocytic Leukemia Up-Regulated 1 | 26 |
| FOXB2 | Forkhead Box B2 | 29 |
| IRF1-AS1 | IRF1 Antisense RNA 1 | 20 |
| SERPINA5 | Serpin Family A Member 5 | 43 |
| ZNF296 | Zinc Finger Protein 296 | 32 |
| DMAP1 | DNA Methyltransferase 1 Associated Protein 1 | 37 |
| LOC157273 | Uncharacterized LOC157273 | 10 |
| LOC101927839 | Uncharacterized LOC101927839 | 12 |
| TSPYL2 | TSPY Like 2 | 36 |
| EP400P1 | EP400 Pseudogene 1 | 18 |
| RN7SKP253 | RN7SK Pseudogene 253 | 6 |
| lnc-SETD7-4 |  | 4 |
| RF00100-103 |  | 3 |
| HSPA6 | Heat Shock Protein Family A (Hsp70) Member 6 | 44 |
| FMNL2 | Formin Like 2 | 37 |
| ENSG00000265356 |  | 7 |
| LRFN2 | Leucine Rich Repeat And Fibronectin Type III Domain Containing 2 | 37 |
| ENSG00000270679 |  | 4 |
| RPRML | Reprimo Like | 28 |
| MELK | Maternal Embryonic Leucine Zipper Kinase | 45 |
| RPL26P19 | Ribosomal Protein L26 Pseudogene 19 | 7 |
| DEFA3 | Defensin Alpha 3 | 36 |
| RF00017-5489 |  | 4 |
| lnc-TMEM184C-2 |  | 3 |
| ANXA9 | Annexin A9 | 39 |
| FCGR2C | Fc Fragment Of IgG Receptor IIc (Gene/Pseudogene) | 34 |
| ECHDC3 | Enoyl-CoA Hydratase Domain Containing 3 | 34 |
| MIR340 | MicroRNA 340 | 18 |
| ALDH8A1 | Aldehyde Dehydrogenase 8 Family Member A1 | 37 |
| piR-43107-137 |  | 4 |
| SERPINB6 | Serpin Family B Member 6 | 43 |
| NBEAL1 | Neurobeachin Like 1 | 34 |
| IFNE | Interferon Epsilon | 35 |
| LST1 | Leukocyte Specific Transcript 1 | 35 |
| CPLX3 | Complexin 3 | 36 |
| KCNH4 | Potassium Voltage-Gated Channel Subfamily H Member 4 | 37 |
| CCDC61 | Coiled-Coil Domain Containing 61 | 32 |
| SH2D4B | SH2 Domain Containing 4B | 31 |
| C14orf93 | Chromosome 14 Open Reading Frame 93 | 31 |
| GGT5 | Gamma-Glutamyltransferase 5 | 39 |
| SILC1 | Sciatic Injury Induced LincRNA Upregulator Of SOX11 | 12 |
| ZNF213-AS1 | ZNF213 Antisense RNA 1 (Head To Head) | 13 |
| S100A7 | S100 Calcium Binding Protein A7 | 40 |
| FALEC | Focally Amplified Long Non-Coding RNA In Epithelial Cancer | 14 |
| RF00017-2999 |  | 1 |
| piR-61028-297 |  | 1 |
| 5MWI_A-124 |  | 2 |
| FUT1 | Fucosyltransferase 1 (H Blood Group) | 40 |
| CCT8 | Chaperonin Containing TCP1 Subunit 8 | 37 |
| EPHB3 | EPH Receptor B3 | 44 |
| SLC12A5-AS1 | SLC12A5 And MMP9 Antisense RNA 1 | 12 |
| DQ485453 |  | 5 |
| RF00017-1333 |  | 4 |
| ENSG00000262879 |  | 6 |
| CPNE3 | Copine 3 | 39 |
| ENSG00000229212 |  | 5 |
| MAPRE3-AS1 | MAPRE3 Antisense RNA 1 | 9 |
| piR-52364 |  | 4 |
| lnc-MYBPHL-1 |  | 4 |
| ZNF77 | Zinc Finger Protein 77 | 36 |
| UNC13C | Unc-13 Homolog C | 34 |
| ERRFI1 | ERBB Receptor Feedback Inhibitor 1 | 37 |
| ENSG00000224228 |  | 8 |
| CAPRIN1 | Cell Cycle Associated Protein 1 | 37 |
| B4GALT6 | Beta-1,4-Galactosyltransferase 6 | 40 |
| SIKE1 | Suppressor Of IKBKE 1 | 35 |
| ARL4A | ADP Ribosylation Factor Like GTPase 4A | 38 |
| CCDC91 | Coiled-Coil Domain Containing 91 | 35 |
| MMP28 | Matrix Metallopeptidase 28 | 37 |
| UTS2B | Urotensin 2B | 31 |
| SLC39A12 | Solute Carrier Family 39 Member 12 | 38 |
| LSM5 | LSM5 Homolog, U6 Small Nuclear RNA And MRNA Degradation Associated | 38 |
| ATXN7L3B | Ataxin 7 Like 3B | 30 |
| FAM118B | Family With Sequence Similarity 118 Member B | 33 |
| KIAA2013 | KIAA2013 | 31 |
| STC2 | Stanniocalcin 2 | 40 |
| MIR181B2 | MicroRNA 181b-2 | 19 |
| SLC25A21 | Solute Carrier Family 25 Member 21 | 41 |
| TSBP1-AS1 | TSBP1 And BTNL2 Antisense RNA 1 | 9 |
| LOC109461477 | Dystrophia Myotonica Protein Kinase Repeat Instability Region | 1 |
| SWAP70 | Switching B Cell Complex Subunit SWAP70 | 39 |
| MRPL9 | Mitochondrial Ribosomal Protein L9 | 34 |
| ELL2 | Elongation Factor For RNA Polymerase II 2 | 37 |
| ISYNA1 | Inositol-3-Phosphate Synthase 1 | 40 |
| RPL28P4 | Ribosomal Protein L28 Pseudogene 4 | 5 |
| OR13G1 | Olfactory Receptor Family 13 Subfamily G Member 1 | 31 |
| HLA-DQB2 | Major Histocompatibility Complex, Class II, DQ Beta 2 | 36 |
| ZNF827 | Zinc Finger Protein 827 | 33 |
| SNORD91A | Small Nucleolar RNA, C/D Box 91A | 14 |
| SNORD91B | Small Nucleolar RNA, C/D Box 91B | 14 |
| ENSG00000278215 |  | 4 |
| LOC100507477 | Uncharacterized LOC100507477 | 7 |
| NRDC | Nardilysin Convertase | 30 |
| C12orf75 | Chromosome 12 Open Reading Frame 75 | 30 |
| MIR8085 | MicroRNA 8085 | 10 |
| RF00017-5486 |  | 4 |
| TIPARP-AS1 | TIPARP Antisense RNA 1 | 14 |
| UGT1A3 | UDP Glucuronosyltransferase Family 1 Member A3 | 38 |
| HELQ | Helicase, POLQ Like | 36 |
| APOC1P1 | Apolipoprotein C1 Pseudogene 1 | 12 |
| STK19 | Serine/Threonine Kinase 19 | 38 |
| CHFR | Checkpoint With Forkhead And Ring Finger Domains | 40 |
| APPL2 | Adaptor Protein, Phosphotyrosine Interacting With PH Domain And Leucine Zipper 2 | 35 |
| C12orf42 | Chromosome 12 Open Reading Frame 42 | 29 |
| PGBD5 | PiggyBac Transposable Element Derived 5 | 33 |
| ITSN2 | Intersectin 2 | 38 |
| PAN3 | Poly(A) Specific Ribonuclease Subunit PAN3 | 36 |
| GBP3 | Guanylate Binding Protein 3 | 38 |
| AK3 | Adenylate Kinase 3 | 42 |
| ZCCHC24 | Zinc Finger CCHC-Type Containing 24 | 32 |
| SERTAD3 | SERTA Domain Containing 3 | 30 |
| ENSG00000266903 |  | 8 |
| FAM135A | Family With Sequence Similarity 135 Member A | 35 |
| VAT1 | Vesicle Amine Transport 1 | 38 |
| HNF1A-AS1 | HNF1A Antisense RNA 1 | 18 |
| RPL19P8 | Ribosomal Protein L19 Pseudogene 8 | 5 |
| TXNL4B | Thioredoxin Like 4B | 36 |
| B3GALT4 | Beta-1,3-Galactosyltransferase 4 | 40 |
| RWDD3 | RWD Domain Containing 3 | 35 |
| UGT1A5 | UDP Glucuronosyltransferase Family 1 Member A5 | 33 |
| ENSG00000286215 |  | 4 |
| BNIPL | BCL2 Interacting Protein Like | 35 |
| VENTXP2 | VENT Homeobox Pseudogene 2 | 6 |
| IL36A | Interleukin 36 Alpha | 37 |
| HLA-DPB2 | Major Histocompatibility Complex, Class II, DP Beta 2 (Pseudogene) | 18 |
| RHOBTB3 | Rho Related BTB Domain Containing 3 | 36 |
| MRPL10 | Mitochondrial Ribosomal Protein L10 | 36 |
| PLA2G15 | Phospholipase A2 Group XV | 37 |
| ENSG00000260971 |  | 8 |
| NTN4 | Netrin 4 | 39 |
| ZFYVE21 | Zinc Finger FYVE-Type Containing 21 | 36 |
| NOP58 | NOP58 Ribonucleoprotein | 38 |
| ENSG00000283573 |  | 5 |
| HBP1 | HMG-Box Transcription Factor 1 | 38 |
| GPATCH3 | G-Patch Domain Containing 3 | 33 |
| NICN1 | Nicolin 1 | 33 |
| MAP3K7CL | MAP3K7 C-Terminal Like | 32 |
| ENSG00000263050 |  | 8 |
| RN7SL608P | RNA, 7SL, Cytoplasmic 608, Pseudogene | 7 |
| lnc-SRPRA-7 |  | 4 |
| RF00017-633 |  | 3 |
| SERPINA9 | Serpin Family A Member 9 | 35 |
| STAB1 | Stabilin 1 | 39 |
| HSPA12B | Heat Shock Protein Family A (Hsp70) Member 12B | 35 |
| TOMM5 | Translocase Of Outer Mitochondrial Membrane 5 | 33 |
| LINC00189 | Long Intergenic Non-Protein Coding RNA 189 | 16 |
| WDR1 | WD Repeat Domain 1 | 37 |
| ANKRD30A | Ankyrin Repeat Domain 30A | 36 |
| TBL1Y | Transducin Beta Like 1 Y-Linked | 32 |
| C15orf39 | Chromosome 15 Open Reading Frame 39 | 33 |
| ENSG00000224063 |  | 7 |
| TLE2 | TLE Family Member 2, Transcriptional Corepressor | 41 |
| MIR362 | MicroRNA 362 | 14 |
| MSBP1 | Minisatellite Binding Protein 1 | 4 |
| MIR425 | MicroRNA 425 | 17 |
| SEMA3F-AS1 | SEMA3F Antisense RNA 1 | 13 |
| RAD23B | RAD23 Homolog B, Nucleotide Excision Repair Protein | 44 |
| ZNF618 | Zinc Finger Protein 618 | 35 |
| RAB5C | RAB5C, Member RAS Oncogene Family | 41 |
| ACKR4 | Atypical Chemokine Receptor 4 | 35 |
| ZNF285 | Zinc Finger Protein 285 | 28 |
| BRMS1L | BRMS1 Like Transcriptional Repressor | 35 |
| hsa-miR-5096-052 |  | 4 |
| HSALNG0048142 |  | 2 |
| MIR3149 | MicroRNA 3149 | 12 |
| EPC1 | Enhancer Of Polycomb Homolog 1 | 39 |
| ASCC3 | Activating Signal Cointegrator 1 Complex Subunit 3 | 38 |
| EAF1-AS1 | EAF1 Antisense RNA 1 | 10 |
| MIR744 | MicroRNA 744 | 15 |
| FBXL16 | F-Box And Leucine Rich Repeat Protein 16 | 33 |
| IP6K2 | Inositol Hexakisphosphate Kinase 2 | 39 |
| MIR199A2 | MicroRNA 199a-2 | 19 |
| RAB3D | RAB3D, Member RAS Oncogene Family | 39 |
| SCARNA21 | Small Cajal Body-Specific RNA 21 | 13 |
| LY6G5B | Lymphocyte Antigen 6 Family Member G5B | 29 |
| PLCG1-AS1 | PLCG1 Antisense RNA 1 | 12 |
| ENSG00000224077 |  | 8 |
| ENSG00000234072 |  | 7 |
| ENSG00000271895 |  | 8 |
| PRR15 | Proline Rich 15 | 33 |
| MIR133A2 | MicroRNA 133a-2 | 18 |
| SCUBE1 | Signal Peptide, CUB Domain And EGF Like Domain Containing 1 | 36 |
| CENPW | Centromere Protein W | 33 |
| LOC101929574 | Uncharacterized LOC101929574 | 10 |
| ARHGAP4 | Rho GTPase Activating Protein 4 | 41 |
| ENSG00000261303 |  | 4 |
| STRCP1 | Stereocilin Pseudogene 1 | 13 |
| PA2G4 | Proliferation-Associated 2G4 | 40 |
| RNPEP | Arginyl Aminopeptidase | 39 |
| LOC100505715 | Uncharacterized LOC100505715 | 10 |
| OR7E14P | Olfactory Receptor Family 7 Subfamily E Member 14 Pseudogene | 14 |
| DCLK2 | Doublecortin Like Kinase 2 | 40 |
| SLC16A3 | Solute Carrier Family 16 Member 3 | 44 |
| hsa-miR-5095-488 |  | 2 |
| UNC45A | Unc-45 Myosin Chaperone A | 34 |
| TGM7 | Transglutaminase 7 | 32 |
| INTS12 | Integrator Complex Subunit 12 | 34 |
| PABPC4L | Poly(A) Binding Protein Cytoplasmic 4 Like | 31 |
| KLHDC1 | Kelch Domain Containing 1 | 34 |
| TMTC2 | Transmembrane O-Mannosyltransferase Targeting Cadherins 2 | 36 |
| MTARC2 | Mitochondrial Amidoxime Reducing Component 2 | 30 |
| ANKRD35 | Ankyrin Repeat Domain 35 | 31 |
| H2BS1 | H2B.S Histone 1 | 22 |
| RF00017-333 |  | 4 |
| ZKSCAN1 | Zinc Finger With KRAB And SCAN Domains 1 | 37 |
| PHLPP1 | PH Domain And Leucine Rich Repeat Protein Phosphatase 1 | 40 |
| RPP25 | Ribonuclease P And MRP Subunit P25 | 34 |
| NBR2 | Neighbor Of BRCA1 LncRNA 2 | 25 |
| ENSG00000226645 |  | 8 |
| piR-58281 |  | 3 |
| piR-57133-172 |  | 2 |
| PLD5 | Phospholipase D Family Member 5 | 35 |
| PARP4 | Poly(ADP-Ribose) Polymerase Family Member 4 | 43 |
| LINGO4 | Leucine Rich Repeat And Ig Domain Containing 4 | 31 |
| NOL4L | Nucleolar Protein 4 Like | 29 |
| OLIG3 | Oligodendrocyte Transcription Factor 3 | 36 |
| ARHGEF15 | Rho Guanine Nucleotide Exchange Factor 15 | 37 |
| SERPINA10 | Serpin Family A Member 10 | 39 |
| TBC1D10A | TBC1 Domain Family Member 10A | 35 |
| SNHG17 | Small Nucleolar RNA Host Gene 17 | 17 |
| FAM76A | Family With Sequence Similarity 76 Member A | 32 |
| SIGLEC12 | Sialic Acid Binding Ig Like Lectin 12 | 38 |
| ZC3H12D | Zinc Finger CCCH-Type Containing 12D | 33 |
| RF00017-1770 |  | 2 |
| TTC4P1 | Tetratricopeptide Repeat Domain 4 Pseudogene 1 | 9 |
| RPL15P15 | Ribosomal Protein L15 Pseudogene 15 | 4 |
| CDH10 | Cadherin 10 | 39 |
| RAB37 | RAB37, Member RAS Oncogene Family | 37 |
| MARCHF2 | Membrane Associated Ring-CH-Type Finger 2 | 27 |
| ZNF608 | Zinc Finger Protein 608 | 35 |
| ENSG00000250899 |  | 9 |
| UBAP2L | Ubiquitin Associated Protein 2 Like | 35 |
| RPL21P81 | Ribosomal Protein L21 Pseudogene 81 | 6 |
| ENSG00000201451 |  | 6 |
| ADTRP | Androgen Dependent TFPI Regulating Protein | 29 |
| SLC39A7 | Solute Carrier Family 39 Member 7 | 41 |
| DUS4L-BCAP29 | DUS4L-BCAP29 Readthrough | 8 |
| piR-36588-047 |  | 4 |
| piR-47211-634 |  | 4 |
| KDM3A | Lysine Demethylase 3A | 41 |
| ABCB10 | ATP Binding Cassette Subfamily B Member 10 | 40 |
| ELFN1 | Extracellular Leucine Rich Repeat And Fibronectin Type III Domain Containing 1 | 32 |
| CALM2P1 | Calmodulin 2 Pseudogene 1 | 8 |
| LRP1-AS | LRP1 Antisense RNA | 11 |
| TRNAU1AP | TRNA Selenocysteine 1 Associated Protein 1 | 32 |
| HSPA12A | Heat Shock Protein Family A (Hsp70) Member 12A | 37 |
| ZNF536 | Zinc Finger Protein 536 | 36 |
| RASAL1 | RAS Protein Activator Like 1 | 39 |
| ZNF101 | Zinc Finger Protein 101 | 34 |
| ZNF143 | Zinc Finger Protein 143 | 39 |
| lnc-UBC-2 |  | 4 |
| GPR31 | G Protein-Coupled Receptor 31 | 34 |
| ENSG00000272849 |  | 5 |
| VASN | Vasorin | 36 |
| CXXC4 | CXXC Finger Protein 4 | 39 |
| TRO | Trophinin | 37 |
| ENSG00000252200 |  | 5 |
| TYW3 | TRNA-YW Synthesizing Protein 3 Homolog | 36 |
| ERI3 | ERI1 Exoribonuclease Family Member 3 | 36 |
| ZNF317 | Zinc Finger Protein 317 | 36 |
| DNAJC5G | DnaJ Heat Shock Protein Family (Hsp40) Member C5 Gamma | 32 |
| LRRTM4 | Leucine Rich Repeat Transmembrane Neuronal 4 | 39 |
| TMSB10 | Thymosin Beta 10 | 35 |
| BCDIN3D | BCDIN3 Domain Containing RNA Methyltransferase | 35 |
| EIF5A | Eukaryotic Translation Initiation Factor 5A | 43 |
| RRS1-AS1 | RRS1 Antisense RNA 1 (Head To Head) | 12 |
| DOT1L | DOT1 Like Histone Lysine Methyltransferase | 44 |
| ENSG00000257894 |  | 9 |
| ENSG00000244036 |  | 7 |
| EFS | Embryonal Fyn-Associated Substrate | 35 |
| RNU7-2P | RNA, U7 Small Nuclear 2 Pseudogene | 5 |
| XKR9 | XK Related 9 | 29 |
| MYRIP | Myosin VIIA And Rab Interacting Protein | 39 |
| ILRUN | Inflammation And Lipid Regulator With UBA-Like And NBR1-Like Domains | 26 |
| RUFY3 | RUN And FYVE Domain Containing 3 | 34 |
| P4HA1 | Prolyl 4-Hydroxylase Subunit Alpha 1 | 40 |
| NABP2 | Nucleic Acid Binding Protein 2 | 34 |
| TCTA | T Cell Leukemia Translocation Altered | 32 |
| EBPL | EBP Like | 33 |
| TMEM132B | Transmembrane Protein 132B | 33 |
| COL24A1 | Collagen Type XXIV Alpha 1 Chain | 36 |
| MIR665 | MicroRNA 665 | 12 |
| LMAN2 | Lectin, Mannose Binding 2 | 38 |
| ENSG00000259212 |  | 8 |
| LSAMP | Limbic System Associated Membrane Protein | 38 |
| CCZ1B | CCZ1 Homolog B, Vacuolar Protein Trafficking And Biogenesis Associated | 29 |
| FBLN7 | Fibulin 7 | 35 |
| UTP20 | UTP20 Small Subunit Processome Component | 31 |
| ENSG00000267131 |  | 8 |
| MIR135B | MicroRNA 135b | 19 |
| ARHGAP28 | Rho GTPase Activating Protein 28 | 36 |
| MIRLET7A3 | MicroRNA Let-7a-3 | 19 |
| ENSG00000235070 |  | 8 |
| ENSG00000251244 |  | 5 |
| COL20A1 | Collagen Type XX Alpha 1 Chain | 35 |
| TTC41P | Tetratricopeptide Repeat Domain 41, Pseudogene | 11 |
| MIR429 | MicroRNA 429 | 19 |
| ENSG00000201843 |  | 4 |
| ENSG00000271629 |  | 3 |
| MAN2A2 | Mannosidase Alpha Class 2A Member 2 | 38 |
| LOC105369391 | Uncharacterized LOC105369391 | 8 |
| ARMC12 | Armadillo Repeat Containing 12 | 30 |
| ZSWIM2 | Zinc Finger SWIM-Type Containing 2 | 31 |
| CCDC6 | Coiled-Coil Domain Containing 6 | 39 |
| MIR9-3 | MicroRNA 9-3 | 19 |
| THEGL | Theg Spermatid Protein Like | 25 |
| RPL21P119 | Ribosomal Protein L21 Pseudogene 119 | 9 |
| lnc-ABO-33 |  | 4 |
| piR-59907-030 |  | 3 |
| MARK2 | Microtubule Affinity Regulating Kinase 2 | 44 |
| PELI1 | Pellino E3 Ubiquitin Protein Ligase 1 | 41 |
| PTRHD1 | Peptidyl-TRNA Hydrolase Domain Containing 1 | 33 |
| MIR6871 | MicroRNA 6871 | 9 |
| lnc-ZHX3-4 |  | 4 |
| CKLF | Chemokine Like Factor | 37 |
| ENSG00000266446 |  | 9 |
| MN309102 |  | 4 |
| lnc-FN1-4 |  | 4 |
| ENSG00000273948 |  | 4 |
| lnc-CXCL12-6 |  | 3 |
| lnc-SVIL-6 |  | 2 |
| NAPG | NSF Attachment Protein Gamma | 36 |
| ZNRD2 | Zinc Ribbon Domain Containing 2 | 28 |
| ENSG00000254987 |  | 8 |
| ARHGEF38 | Rho Guanine Nucleotide Exchange Factor 38 | 29 |
| EXOSC5 | Exosome Component 5 | 39 |
| KCTD16 | Potassium Channel Tetramerization Domain Containing 16 | 36 |
| lnc-BCL3-1 |  | 5 |
| lnc-BCL3-5 |  | 4 |
| ITM2C | Integral Membrane Protein 2C | 38 |
| PLCL2 | Phospholipase C Like 2 | 38 |
| FLOT2 | Flotillin 2 | 41 |
| OTUD7B | OTU Deubiquitinase 7B | 35 |
| SYTL1 | Synaptotagmin Like 1 | 36 |
| ENSG00000268810 |  | 7 |
| COL28A1 | Collagen Type XXVIII Alpha 1 Chain | 33 |
| ARMH3 | Armadillo Like Helical Domain Containing 3 | 23 |
| PGS1 | Phosphatidylglycerophosphate Synthase 1 | 39 |
| lnc-NGEF-1 |  | 4 |
| DHRS7 | Dehydrogenase/Reductase 7 | 36 |
| RN7SL802P | RNA, 7SL, Cytoplasmic 802, Pseudogene | 4 |
| GPN2 | GPN-Loop GTPase 2 | 32 |
| GPR39 | G Protein-Coupled Receptor 39 | 40 |
| ENSG00000247372 |  | 7 |
| P2RY13 | Purinergic Receptor P2Y13 | 42 |
| lnc-TWIST1-4 |  | 4 |
| GNRHR2 | Gonadotropin Releasing Hormone Receptor 2 (Pseudogene) | 31 |
| ENSG00000229854 |  | 6 |
| ARHGEF17 | Rho Guanine Nucleotide Exchange Factor 17 | 37 |
| EP400 | E1A Binding Protein P400 | 36 |
| ZNF318 | Zinc Finger Protein 318 | 35 |
| ZNF451 | Zinc Finger Protein 451 | 35 |
| MIR124-3 | MicroRNA 124-3 | 17 |
| DNAJB9 | DnaJ Heat Shock Protein Family (Hsp40) Member B9 | 38 |
| ZNF383 | Zinc Finger Protein 383 | 31 |
| RFPL4B | Ret Finger Protein Like 4B | 30 |
| LPAR2 | Lysophosphatidic Acid Receptor 2 | 44 |
| SAYSD1 | SAYSVFN Motif Domain Containing 1 | 31 |
| NAV3 | Neuron Navigator 3 | 33 |
| ABI2 | Abl Interactor 2 | 39 |
| ENSG00000244151 |  | 7 |
| lnc-MTAP-3 |  | 4 |
| ENSG00000243831 |  | 2 |
| MIR101-2 | MicroRNA 101-2 | 16 |
| BTN2A1 | Butyrophilin Subfamily 2 Member A1 | 37 |
| MIR103A2 | MicroRNA 103a-2 | 18 |
| NPTX1 | Neuronal Pentraxin 1 | 37 |
| C1QTNF6 | C1q And TNF Related 6 | 37 |
| ENSG00000229533 |  | 8 |
| TMEM161B | Transmembrane Protein 161B | 36 |
| MIR339 | MicroRNA 339 | 19 |
| S1PR5 | Sphingosine-1-Phosphate Receptor 5 | 45 |
| TMEM26 | Transmembrane Protein 26 | 31 |
| HAUS3 | HAUS Augmin Like Complex Subunit 3 | 33 |
| EIF1AX | Eukaryotic Translation Initiation Factor 1A X-Linked | 41 |
| TRAM1 | Translocation Associated Membrane Protein 1 | 39 |
| BET1L | Bet1 Golgi Vesicular Membrane Trafficking Protein Like | 35 |
| OAZ3 | Ornithine Decarboxylase Antizyme 3 | 33 |
| BTBD2 | BTB Domain Containing 2 | 36 |
| EGFL8 | EGF Like Domain Multiple 8 | 34 |
| SAPCD1 | Suppressor APC Domain Containing 1 | 25 |
| NDFIP2 | Nedd4 Family Interacting Protein 2 | 37 |
| ELK3 | ETS Transcription Factor ELK3 | 39 |
| ACOT6 | Acyl-CoA Thioesterase 6 | 29 |
| lnc-TMEM212-5 |  | 5 |
| CNGA2 | Cyclic Nucleotide Gated Channel Subunit Alpha 2 | 39 |
| TNPO1 | Transportin 1 | 39 |
| MRTFB | Myocardin Related Transcription Factor B | 29 |
| MIR490 | MicroRNA 490 | 16 |
| SPSB4 | SplA/Ryanodine Receptor Domain And SOCS Box Containing 4 | 32 |
| SSBP4 | Single Stranded DNA Binding Protein 4 | 35 |
| CNTNAP4 | Contactin Associated Protein Family Member 4 | 36 |
| ZNF589 | Zinc Finger Protein 589 | 35 |
| ALS2CL | ALS2 C-Terminal Like | 35 |
| TNFSF18 | TNF Superfamily Member 18 | 38 |
| NUDT17 | Nudix Hydrolase 17 | 29 |
| GNGT2 | G Protein Subunit Gamma Transducin 2 | 40 |
| ZNF521 | Zinc Finger Protein 521 | 37 |
| NTAN1 | N-Terminal Asparagine Amidase | 37 |
| MBL1P | Mannose Binding Lectin 1, Pseudogene | 12 |
| lnc-ABCA12-8 |  | 5 |
| lnc-SLC2A12-6 |  | 4 |
| lnc-ATIC-10 |  | 4 |
| lnc-MAP3K8-16 |  | 4 |
| JA662170 |  | 4 |
| lnc-TCF21-2 |  | 3 |
| piR-36575-070 |  | 3 |
| piR-59928-062 |  | 2 |
| ZP4 | Zona Pellucida Glycoprotein 4 | 31 |
| OS9 | OS9 Endoplasmic Reticulum Lectin | 40 |
| AQP8 | Aquaporin 8 | 37 |
| ENSG00000267191 |  | 6 |
| lnc-CEACAM20-2 |  | 5 |
| PGBD3P3 | PiggyBac Transposable Element Derived 3 Pseudogene 3 | 4 |
| ACOT13 | Acyl-CoA Thioesterase 13 | 37 |
| ENSG00000287956 |  | 4 |
| MIR5702 | MicroRNA 5702 | 10 |
| NUTF2 | Nuclear Transport Factor 2 | 36 |
| MIR652 | MicroRNA 652 | 16 |
| TOMM22P6 | TOMM22 Pseudogene 6 | 6 |
| FOXA3 | Forkhead Box A3 | 39 |
| GSDMA | Gasdermin A | 36 |
| ENSG00000277653 |  | 5 |
| lnc-TWIST1-1 |  | 3 |
| piR-41525-616 |  | 2 |
| GALNT4 | Polypeptide N-Acetylgalactosaminyltransferase 4 | 39 |
| RNU7-66P | RNA, U7 Small Nuclear 66 Pseudogene | 6 |
| GRM4 | Glutamate Metabotropic Receptor 4 | 44 |
| KIR2DS2 | Killer Cell Immunoglobulin Like Receptor, Two Ig Domains And Short Cytoplasmic Tail 2 | 24 |
| ENSG00000251864 |  | 6 |
| TMEM204 | Transmembrane Protein 204 | 33 |
| MRPL19 | Mitochondrial Ribosomal Protein L19 | 37 |
| UBLCP1 | Ubiquitin Like Domain Containing CTD Phosphatase 1 | 36 |
| A1BG | Alpha-1-B Glycoprotein | 37 |
| MIR584 | MicroRNA 584 | 17 |
| KLHL25 | Kelch Like Family Member 25 | 35 |
| PGBD1 | PiggyBac Transposable Element Derived 1 | 35 |
| NONHSAG027316.2 |  | 4 |
| ENSG00000279757 |  | 4 |
| piR-32214-278 |  | 3 |
| lnc-SLC5A6-3 |  | 3 |
| lnc-TP53-2 |  | 3 |
| lnc-PCSK7-3 |  | 3 |
| ZNF12 | Zinc Finger Protein 12 | 36 |
| PGGHG | Protein-Glucosylgalactosylhydroxylysine Glucosidase | 28 |
| DPEP2 | Dipeptidase 2 | 40 |
| PRIM2 | DNA Primase Subunit 2 | 41 |
| EVI5 | Ecotropic Viral Integration Site 5 | 38 |
| HNRNPA3P1 | Heterogeneous Nuclear Ribonucleoprotein A3 Pseudogene 1 | 15 |
| SLC35F4 | Solute Carrier Family 35 Member F4 | 28 |
| CMTM1 | CKLF Like MARVEL Transmembrane Domain Containing 1 | 32 |
| COL4A2-AS2 | COL4A2 Antisense 2 | 17 |
| ENSG00000255872 |  | 6 |
| lnc-HECA-12 |  | 3 |
| LOC107986652 | Uncharacterized LOC107986652 | 2 |
| MIIP | Migration And Invasion Inhibitory Protein | 35 |
| TMEM209 | Transmembrane Protein 209 | 34 |
| GPR180 | G Protein-Coupled Receptor 180 | 35 |
| GADD45B | Growth Arrest And DNA Damage Inducible Beta | 39 |
| MIR194-2 | MicroRNA 194-2 | 19 |
| QPCTL | Glutaminyl-Peptide Cyclotransferase Like | 34 |
| NAA80 | N-Alpha-Acetyltransferase 80, NatH Catalytic Subunit | 25 |
| DCD | Dermcidin | 36 |
| CAMLG | Calcium Modulating Ligand | 40 |
| ZDHHC1 | Zinc Finger DHHC-Type Containing 1 | 35 |
| MTCO3P1 | MT-CO3 Pseudogene 1 | 7 |
| IRF2BP1 | Interferon Regulatory Factor 2 Binding Protein 1 | 35 |
| FCAMR | Fc Fragment Of IgA And IgM Receptor | 35 |
| WBP1L | WW Domain Binding Protein 1 Like | 32 |
| ENSG00000224477 |  | 5 |
| RF00017-7477 |  | 4 |
| PTPRA | Protein Tyrosine Phosphatase Receptor Type A | 45 |
| LOC100129540 | Uncharacterized LOC100129540 | 8 |
| ENSG00000232290 |  | 7 |
| ENSG00000237719 |  | 6 |
| lnc-PPP1CC-3 |  | 5 |
| lnc-PLD1-2 |  | 5 |
| NONHSAG036668.2 |  | 4 |
| ABI3 | ABI Family Member 3 | 38 |
| lnc-TMEM258-1 |  | 3 |
| PSKH1 | Protein Serine Kinase H1 | 39 |
| ENSG00000229116 |  | 8 |
| SNHG18 | Small Nucleolar RNA Host Gene 18 | 14 |
| MIR4513 | MicroRNA 4513 | 13 |
| MED6 | Mediator Complex Subunit 6 | 37 |
| TAOK1 | TAO Kinase 1 | 43 |
| NME4 | NME/NM23 Nucleoside Diphosphate Kinase 4 | 42 |
| GLRA3 | Glycine Receptor Alpha 3 | 41 |
| SNORA73B | Small Nucleolar RNA, H/ACA Box 73B | 17 |
| CST9 | Cystatin 9 | 32 |
| LINC00881 | Long Intergenic Non-Protein Coding RNA 881 | 13 |
| FSTL5 | Follistatin Like 5 | 35 |
| S100A10 | S100 Calcium Binding Protein A10 | 45 |
| MIR30C2 | MicroRNA 30c-2 | 19 |
| DNAJC8 | DnaJ Heat Shock Protein Family (Hsp40) Member C8 | 35 |
| LINC00910 | Long Intergenic Non-Protein Coding RNA 910 | 13 |
| TMEM161A | Transmembrane Protein 161A | 33 |
| ZNF326 | Zinc Finger Protein 326 | 35 |
| MINDY2 | MINDY Lysine 48 Deubiquitinase 2 | 27 |
| CDKN2A-DT | CDKN2A Divergent Transcript | 17 |
| CISD1P1 | CDGSH Iron Sulfur Domain 1 Pseudogene 1 | 6 |
| ANGPTL1 | Angiopoietin Like 1 | 41 |
| H2AC21 | H2A Clustered Histone 21 | 27 |
| SLC16A10 | Solute Carrier Family 16 Member 10 | 39 |
| FOXO4 | Forkhead Box O4 | 43 |
| GGT7 | Gamma-Glutamyltransferase 7 | 39 |
| XKR4 | XK Related 4 | 32 |
| PDXDC1 | Pyridoxal Dependent Decarboxylase Domain Containing 1 | 37 |
| ENSG00000262633 |  | 9 |
| lnc-DIRC3-3 |  | 4 |
| piR-52371 |  | 4 |
| lnc-DIRC3-4 |  | 3 |
| PRAM1 | PML-RARA Regulated Adaptor Molecule 1 | 34 |
| PLEKHH3 | Pleckstrin Homology, MyTH4 And FERM Domain Containing H3 | 32 |
| ENSG00000283549 |  | 4 |
| ARRDC2 | Arrestin Domain Containing 2 | 32 |
| RNU6-976P | RNA, U6 Small Nuclear 976, Pseudogene | 6 |
| PRMT5P1 | Protein Arginine Methyltransferase 5 Pseudogene 1 | 5 |
| LOC106728418 | LEP 5' Regulatory Region | 1 |
| ENSG00000272106 |  | 8 |
| TCF23 | Transcription Factor 23 | 31 |
| CAND2 | Cullin Associated And Neddylation Dissociated 2 (Putative) | 35 |
| CPEB2 | Cytoplasmic Polyadenylation Element Binding Protein 2 | 35 |
| CDH8 | Cadherin 8 | 41 |
| MIR19B2 | MicroRNA 19b-2 | 16 |
| TMEM176A | Transmembrane Protein 176A | 35 |
| ZNF699 | Zinc Finger Protein 699 | 35 |
| HACD3 | 3-Hydroxyacyl-CoA Dehydratase 3 | 35 |
| ENSG00000255062 |  | 8 |
| ANKRD29 | Ankyrin Repeat Domain 29 | 33 |
| EPB41L4A | Erythrocyte Membrane Protein Band 4.1 Like 4A | 34 |
| VNN1 | Vanin 1 | 44 |
| KLF16 | Kruppel Like Factor 16 | 33 |
| RAB42 | RAB42, Member RAS Oncogene Family | 33 |
| PAQR5 | Progestin And AdipoQ Receptor Family Member 5 | 36 |
| ACTG1P20 | Actin Gamma 1 Pseudogene 20 | 9 |
| SPARCL1 | SPARC Like 1 | 36 |
| RGS19 | Regulator Of G Protein Signaling 19 | 40 |
| RF00017-2146 |  | 4 |
| RF00998-025 |  | 2 |
| ECE2 | Endothelin Converting Enzyme 2 | 38 |
| RNF4 | Ring Finger Protein 4 | 40 |
| ZNF414 | Zinc Finger Protein 414 | 29 |
| ARHGAP25 | Rho GTPase Activating Protein 25 | 35 |
| RPL7AP47 | Ribosomal Protein L7a Pseudogene 47 | 9 |
| TUBA3C | Tubulin Alpha 3c | 37 |
| ENSG00000285804 |  | 6 |
| ENSG00000233842 |  | 6 |
| L13304-005 |  | 4 |
| BANCR | BRAF-Activated Non-Protein Coding RNA | 12 |
| RPL36AP23 | Ribosomal Protein L36a Pseudogene 23 | 5 |
| MUC17 | Mucin 17, Cell Surface Associated | 35 |
| KIF4A | Kinesin Family Member 4A | 40 |
| C5orf67 | Chromosome 5 Putative Open Reading Frame 67 | 18 |
| RFC4 | Replication Factor C Subunit 4 | 41 |
| ZNF232 | Zinc Finger Protein 232 | 32 |
| ACTBP7 | ACTB Pseudogene 7 | 8 |
| RNU6-554P | RNA, U6 Small Nuclear 554, Pseudogene | 6 |
| piR-56133-071 |  | 2 |
| piR-43105-201 |  | 2 |
| NMBR | Neuromedin B Receptor | 42 |
| S100P | S100 Calcium Binding Protein P | 40 |
| NELFE | Negative Elongation Factor Complex Member E | 33 |
| EEF1E1 | Eukaryotic Translation Elongation Factor 1 Epsilon 1 | 40 |
| ENSG00000267340 |  | 5 |
| CEP20 | Centrosomal Protein 20 | 27 |
| DUSP18 | Dual Specificity Phosphatase 18 | 36 |
| GPR63 | G Protein-Coupled Receptor 63 | 36 |
| RNU1-88P | RNA, U1 Small Nuclear 88, Pseudogene | 7 |
| ZNF568 | Zinc Finger Protein 568 | 32 |
| ALPG | Alkaline Phosphatase, Germ Cell | 32 |
| FCRLB | Fc Receptor Like B | 35 |
| TNS1-AS1 | TNS1 Antisense RNA 1 | 9 |
| KLHL8 | Kelch Like Family Member 8 | 36 |
| CWC22 | CWC22 Spliceosome Associated Protein Homolog | 33 |
| FCMR | Fc Fragment Of IgM Receptor | 28 |
| LINC00880 | Long Intergenic Non-Protein Coding RNA 880 | 15 |
| ILF2 | Interleukin Enhancer Binding Factor 2 | 38 |
| LMAN1L | Lectin, Mannose Binding 1 Like | 36 |
| CLEC1B | C-Type Lectin Domain Family 1 Member B | 40 |
| MIR9-2 | MicroRNA 9-2 | 18 |
| MORN1 | MORN Repeat Containing 1 | 31 |
| RFX1 | Regulatory Factor X1 | 38 |
| CDCA8 | Cell Division Cycle Associated 8 | 41 |
| MARCHF1 | Membrane Associated Ring-CH-Type Finger 1 | 28 |
| BCL2L14 | BCL2 Like 14 | 38 |
| UFL1 | UFM1 Specific Ligase 1 | 35 |
| SLC9A8 | Solute Carrier Family 9 Member A8 | 39 |
| POLR1E | RNA Polymerase I Subunit E | 36 |
| PDCD5 | Programmed Cell Death 5 | 39 |
| ENSG00000256966 |  | 7 |
| LINC01929 | Long Intergenic Non-Protein Coding RNA 1929 | 10 |
| LOC100506472 | Uncharacterized LOC100506472 | 7 |
| STX2 | Syntaxin 2 | 37 |
| BORCS5 | BLOC-1 Related Complex Subunit 5 | 26 |
| ARAP1 | ArfGAP With RhoGAP Domain, Ankyrin Repeat And PH Domain 1 | 40 |
| CSNK1G2 | Casein Kinase 1 Gamma 2 | 45 |
| EIF3FP3 | Eukaryotic Translation Initiation Factor 3 Subunit F Pseudogene 3 | 8 |
| TRIM9 | Tripartite Motif Containing 9 | 39 |
| PPCDC | Phosphopantothenoylcysteine Decarboxylase | 41 |
| KIAA1217 | KIAA1217 | 36 |
| SIDT2 | SID1 Transmembrane Family Member 2 | 35 |
| PBX4 | PBX Homeobox 4 | 35 |
| SH3YL1 | SH3 And SYLF Domain Containing 1 | 34 |
| lnc-FYB2-5 |  | 5 |
| lnc-CHRNA5-3 |  | 4 |
| piR-52438-030 |  | 3 |
| PDXP | Pyridoxal Phosphatase | 39 |
| SNORD16 | Small Nucleolar RNA, C/D Box 16 | 14 |
| AAMP | Angio Associated Migratory Cell Protein | 36 |
| R3HDM2 | R3H Domain Containing 2 | 33 |
| MTFP1 | Mitochondrial Fission Process 1 | 32 |
| RPS4XP18 | Ribosomal Protein S4X Pseudogene 18 | 6 |
| TREML1 | Triggering Receptor Expressed On Myeloid Cells Like 1 | 37 |
| EIF5 | Eukaryotic Translation Initiation Factor 5 | 41 |
| ENSG00000267681 |  | 4 |
| GALR2 | Galanin Receptor 2 | 41 |
| ENSG00000269148 |  | 6 |
| ASGR1 | Asialoglycoprotein Receptor 1 | 40 |
| ANAPC13 | Anaphase Promoting Complex Subunit 13 | 37 |
| ATG9A | Autophagy Related 9A | 39 |
| PROB1 | Proline Rich Basic Protein 1 | 25 |
| TDRD9 | Tudor Domain Containing 9 | 40 |
| SHISA5 | Shisa Family Member 5 | 36 |
| GALM | Galactose Mutarotase | 44 |
| GPR132 | G Protein-Coupled Receptor 132 | 40 |
| CDIP1 | Cell Death Inducing P53 Target 1 | 35 |
| KCNN1 | Potassium Calcium-Activated Channel Subfamily N Member 1 | 39 |
| PIGU | Phosphatidylinositol Glycan Anchor Biosynthesis Class U | 36 |
| HE855947 |  | 3 |
| MIR216A | MicroRNA 216a | 19 |
| ENSG00000261783 |  | 8 |
| USP39 | Ubiquitin Specific Peptidase 39 | 38 |
| ENSG00000234589 |  | 4 |
| LOC102724965 | Uncharacterized LOC102724965 | 5 |
| NIPSNAP3A | Nipsnap Homolog 3A | 36 |
| CBWD1 | COBW Domain Containing 1 | 33 |
| NR2C2AP | Nuclear Receptor 2C2 Associated Protein | 32 |
| TRAF3IP3 | TRAF3 Interacting Protein 3 | 35 |
| CALML3-AS1 | CALML3 Antisense RNA 1 | 16 |
| ZBTB46 | Zinc Finger And BTB Domain Containing 46 | 35 |
| BLOC1S5 | Biogenesis Of Lysosomal Organelles Complex 1 Subunit 5 | 34 |
| OCM2 | Oncomodulin 2 | 27 |
| APOOL | Apolipoprotein O Like | 34 |
| PBK | PDZ Binding Kinase | 43 |
| AQP10 | Aquaporin 10 | 36 |
| FEM1A | Fem-1 Homolog A | 37 |
| TASOR | Transcription Activation Suppressor | 27 |
| CENPO | Centromere Protein O | 35 |
| CNN3 | Calponin 3 | 35 |
| ATP7BP1 | ATPase Copper Transporting Beta Pseudogene 1 | 6 |
| RNF181 | Ring Finger Protein 181 | 36 |
| MIR548A3 | MicroRNA 548a-3 | 15 |
| LINC01810 | Long Intergenic Non-Protein Coding RNA 1810 | 8 |
| ENSG00000237590 |  | 6 |
| ENSG00000258603 |  | 6 |
| ENSG00000235911 |  | 6 |
| lnc-HACL1-2 |  | 4 |
| MN309628 |  | 4 |
| lnc-FLT3-2 |  | 4 |
| ENSG00000229857 |  | 4 |
| ENSG00000212229 |  | 4 |
| piR-50437-456 |  | 3 |
| MTCYBP27 | MT-CYB Pseudogene 27 | 3 |
| piR-36746-014 |  | 3 |
| HSALNG0022692 |  | 2 |
| ENSG00000279200 |  | 2 |
| LINC01438 | Long Intergenic Non-Protein Coding RNA 1438 | 8 |
| GABRG1 | Gamma-Aminobutyric Acid Type A Receptor Subunit Gamma1 | 39 |
| KDELR2 | KDEL Endoplasmic Reticulum Protein Retention Receptor 2 | 39 |
| CCDC80 | Coiled-Coil Domain Containing 80 | 36 |
| SLC22A16 | Solute Carrier Family 22 Member 16 | 37 |
| NAT8 | N-Acetyltransferase 8 (Putative) | 37 |
| EEF1AKMT2 | EEF1A Lysine Methyltransferase 2 | 25 |
| IFI35 | Interferon Induced Protein 35 | 37 |
| IL36B | Interleukin 36 Beta | 35 |
| PRXL2A | Peroxiredoxin Like 2A | 27 |
| MIR874 | MicroRNA 874 | 18 |
| ENOX1 | Ecto-NOX Disulfide-Thiol Exchanger 1 | 36 |
| GFOD1 | Glucose-Fructose Oxidoreductase Domain Containing 1 | 35 |
| RPL23AP28 | Ribosomal Protein L23a Pseudogene 28 | 6 |
| NINJ2 | Ninjurin 2 | 35 |
| JMJD4 | Jumonji Domain Containing 4 | 35 |
| DNM1P37 | Dynamin 1 Pseudogene 37 | 4 |
| ANP32C | Acidic Nuclear Phosphoprotein 32 Family Member C | 28 |
| CSMD2 | CUB And Sushi Multiple Domains 2 | 37 |
| ENSG00000269843 |  | 8 |
| NALT1 | NOTCH1 Associated LncRNA In T Cell Acute Lymphoblastic Leukemia 1 | 12 |
| ARPP19 | CAMP Regulated Phosphoprotein 19 | 37 |
| TENT5B | Terminal Nucleotidyltransferase 5B | 23 |
| LINC01719 | Long Intergenic Non-Protein Coding RNA 1719 | 9 |
| RN7SL624P | RNA, 7SL, Cytoplasmic 624, Pseudogene | 7 |
| lnc-SRR-6 |  | 5 |
| RF00017-2220 |  | 4 |
| RF00017-5867 |  | 3 |
| HSALNG0023074 |  | 2 |
| PAG1 | Phosphoprotein Membrane Anchor With Glycosphingolipid Microdomains 1 | 38 |
| LINC00992 | Long Intergenic Non-Protein Coding RNA 992 | 12 |
| CDK14 | Cyclin Dependent Kinase 14 | 40 |
| FAM238C | Family With Sequence Similarity 238 Member C | 10 |
| IFN1@ | Interferon, Type 1, Cluster | 4 |
| ATXN7L1 | Ataxin 7 Like 1 | 32 |
| WDR86-AS1 | WDR86 Antisense RNA 1 | 13 |
| MOV10 | Mov10 RISC Complex RNA Helicase | 40 |
| AEN | Apoptosis Enhancing Nuclease | 36 |
| C1orf220 | Chromosome 1 Putative Open Reading Frame 220 | 24 |
| TMEM91 | Transmembrane Protein 91 | 36 |
| LHFPL2 | LHFPL Tetraspan Subfamily Member 2 | 33 |
| CATSPER2P1 | Cation Channel Sperm Associated 2 Pseudogene 1 | 12 |
| ANGPTL5 | Angiopoietin Like 5 | 34 |
| KCNK7 | Potassium Two Pore Domain Channel Subfamily K Member 7 | 37 |
| RGL3 | Ral Guanine Nucleotide Dissociation Stimulator Like 3 | 33 |
| PLA2G4C | Phospholipase A2 Group IVC | 43 |
| ENSG00000249738 |  | 8 |
| SCAMP2 | Secretory Carrier Membrane Protein 2 | 38 |
| ADAMTS9-AS2 | ADAMTS9 Antisense RNA 2 | 16 |
| LYRM2 | LYR Motif Containing 2 | 33 |
| NUFIP1 | Nuclear FMR1 Interacting Protein 1 | 34 |
| PLEKHA6 | Pleckstrin Homology Domain Containing A6 | 36 |
| MVB12B | Multivesicular Body Subunit 12B | 33 |
| CADM2 | Cell Adhesion Molecule 2 | 36 |
| OPN3 | Opsin 3 | 39 |
| SRIP1 | Sorcin Pseudogene 1 | 6 |
| MINDY4 | MINDY Lysine 48 Deubiquitinase 4 | 24 |
| GLP2R | Glucagon Like Peptide 2 Receptor | 44 |
| ENSG00000235236 |  | 7 |
| MIR297 | MicroRNA 297 | 11 |
| ENSG00000223838 |  | 7 |
| THEMIS3P | Thymocyte Selection Associated Family Member 3, Pseudogene | 5 |
| RF00017-5416 |  | 4 |
| SCAF11 | SR-Related CTD Associated Factor 11 | 33 |
| IGKV2D-29 | Immunoglobulin Kappa Variable 2D-29 | 13 |
| RN7SL444P | RNA, 7SL, Cytoplasmic 444, Pseudogene | 6 |
| SPATA6L | Spermatogenesis Associated 6 Like | 29 |
| MPHOSPH10 | M-Phase Phosphoprotein 10 | 37 |
| HDGFL3 | HDGF Like 3 | 29 |
| UFSP1 | UFM1 Specific Peptidase 1 (Inactive) | 32 |
| NPY4R | Neuropeptide Y Receptor Y4 | 35 |
| PRMT3 | Protein Arginine Methyltransferase 3 | 41 |
| SPINK2 | Serine Peptidase Inhibitor Kazal Type 2 | 38 |
| C1orf43 | Chromosome 1 Open Reading Frame 43 | 34 |
| LINC00333 | Long Intergenic Non-Protein Coding RNA 333 | 13 |
| OBSCN-AS1 | OBSCN Antisense RNA 1 | 16 |
| LINC02516 | Long Intergenic Non-Protein Coding RNA 2516 | 9 |
| ENSG00000259992 |  | 6 |
| MYCBP | MYC Binding Protein | 38 |
| OLFML2B | Olfactomedin Like 2B | 34 |
| SPC25 | SPC25 Component Of NDC80 Kinetochore Complex | 32 |
| KSR2 | Kinase Suppressor Of Ras 2 | 39 |
| ENSG00000267758 |  | 8 |
| ENSG00000259276 |  | 8 |
| lnc-RPS13-5 |  | 5 |
| ANKFN1 | Ankyrin Repeat And Fibronectin Type III Domain Containing 1 | 33 |
| RLFP1 | RLF Pseudogene 1 | 4 |
| ENSG00000268030 |  | 7 |
| ENSG00000286192 |  | 6 |
| SPINK13 | Serine Peptidase Inhibitor Kazal Type 13 | 31 |
| ZCCHC7 | Zinc Finger CCHC-Type Containing 7 | 36 |
| TSGA10IP | Testis Specific 10 Interacting Protein | 29 |
| GPR26 | G Protein-Coupled Receptor 26 | 36 |
| CCDC71 | Coiled-Coil Domain Containing 71 | 29 |
| EFCAB13 | EF-Hand Calcium Binding Domain 13 | 29 |
| MG828730-062 |  | 4 |
| ZNF512 | Zinc Finger Protein 512 | 32 |
| LINC02569 | Long Intergenic Non-Protein Coding RNA 2569 | 9 |
| OAZ2 | Ornithine Decarboxylase Antizyme 2 | 36 |
| DNAH10OS | Dynein Axonemal Heavy Chain 10 Opposite Strand | 19 |
| PRR30 | Proline Rich 30 | 27 |
| TSPAN14 | Tetraspanin 14 | 35 |
| RASL11A | RAS Like Family 11 Member A | 29 |
| CERS2 | Ceramide Synthase 2 | 40 |
| NFYB | Nuclear Transcription Factor Y Subunit Beta | 40 |
| NPM1P39 | Nucleophosmin 1 Pseudogene 39 | 8 |
| ENSG00000260063 |  | 8 |
| MIR2861 | MicroRNA 2861 | 14 |
| RASGEF1B | RasGEF Domain Family Member 1B | 36 |
| FOXA1 | Forkhead Box A1 | 43 |
| HERC6 | HECT And RLD Domain Containing E3 Ubiquitin Protein Ligase Family Member 6 | 36 |
| OAZ1 | Ornithine Decarboxylase Antizyme 1 | 39 |
| ZNF559 | Zinc Finger Protein 559 | 33 |
| TLCD5 | TLC Domain Containing 5 | 24 |
| PRR7 | Proline Rich 7, Synaptic | 28 |
| RNF169 | Ring Finger Protein 169 | 33 |
| SURF2 | Surfeit 2 | 36 |
| SPATA2 | Spermatogenesis Associated 2 | 34 |
| DOK6 | Docking Protein 6 | 36 |
| MMRN2 | Multimerin 2 | 34 |
| ARL4AP5 | ADP Ribosylation Factor Like GTPase 4A Pseudogene 5 | 5 |
| lnc-C10orf142-5 |  | 4 |
| lnc-PLEKHG1-1 |  | 4 |
| lnc-CHRNB4-1 |  | 3 |
| lnc-CHRNB4-2 |  | 2 |
| piR-45301-008 |  | 2 |
| LOC105370913 | Uncharacterized LOC105370913 | 1 |
| NOA1 | Nitric Oxide Associated 1 | 34 |
| IZUMO1 | Izumo Sperm-Egg Fusion 1 | 34 |
| SNORD14B | Small Nucleolar RNA, C/D Box 14B | 14 |
| ZFX | Zinc Finger Protein X-Linked | 37 |
| LAIR1 | Leukocyte Associated Immunoglobulin Like Receptor 1 | 41 |
| AKR1B1P6 | Aldo-Keto Reductase Family 1 Member B1 Pseudogene 6 | 6 |
| ADI1P1 | Acireductone Dioxygenase 1 Pseudogene 1 | 5 |
| lnc-CYS1-6 |  | 4 |
| ENSG00000286494 |  | 4 |
| HMGB3P13 | High Mobility Group Box 3 Pseudogene 13 | 4 |
| piR-61532-286 |  | 2 |
| RF00066-106 |  | 1 |
| lnc-BCL7B-3 |  | 1 |
| HSALNG0019418 |  | 1 |
| ENSG00000213184 |  | 1 |
| ENSG00000249411 |  | 1 |
| PDPR | Pyruvate Dehydrogenase Phosphatase Regulatory Subunit | 38 |
| AIDA | Axin Interactor, Dorsalization Associated | 33 |
| ST6GAL2 | ST6 Beta-Galactoside Alpha-2,6-Sialyltransferase 2 | 39 |
| LOC105373944 | Translation Initiation Factor IF-2-Like | 6 |
| UPF2 | UPF2 Regulator Of Nonsense Mediated MRNA Decay | 40 |
| CYB5D2 | Cytochrome B5 Domain Containing 2 | 33 |
| PARP14 | Poly(ADP-Ribose) Polymerase Family Member 14 | 37 |
| TAS2R50 | Taste 2 Receptor Member 50 | 32 |
| ISLR2 | Immunoglobulin Superfamily Containing Leucine Rich Repeat 2 | 35 |
| ENSG00000279668 |  | 5 |
| SPTLC3 | Serine Palmitoyltransferase Long Chain Base Subunit 3 | 40 |
| TFPT | TCF3 Fusion Partner | 35 |
| PLEKHH2 | Pleckstrin Homology, MyTH4 And FERM Domain Containing H2 | 37 |
| RAPGEF5 | Rap Guanine Nucleotide Exchange Factor 5 | 39 |
| RNF111 | Ring Finger Protein 111 | 37 |
| TMC4 | Transmembrane Channel Like 4 | 35 |
| SENCR | Smooth Muscle And Endothelial Cell Enriched Migration/Differentiation-Associated LncRNA | 14 |
| VEPH1 | Ventricular Zone Expressed PH Domain Containing 1 | 35 |
| NYAP1 | Neuronal Tyrosine Phosphorylated Phosphoinositide-3-Kinase Adaptor 1 | 30 |
| MFSD4A | Major Facilitator Superfamily Domain Containing 4A | 25 |
| PPP1R14BP5 | Protein Phosphatase 1 Regulatory Inhibitor Subunit 14B Pseudogene 5 | 6 |
| OR13C1P | Olfactory Receptor Family 13 Subfamily C Member 1 Pseudogene | 6 |
| OR13I1P | Olfactory Receptor Family 13 Subfamily I Member 1 Pseudogene | 5 |
| lnc-FOXB2-3 |  | 4 |
| piR-32214-205 |  | 2 |
| piR-33303-068 |  | 2 |
| Hsa-Mir-574_5p-059 |  | 2 |
| piR-37656-252 |  | 2 |
| ENSG00000255086 |  | 2 |
| piR-44881 |  | 1 |
| ENSG00000251477 |  | 1 |
| USP19 | Ubiquitin Specific Peptidase 19 | 39 |
| DDX47 | DEAD-Box Helicase 47 | 37 |
| RRNAD1 | Ribosomal RNA Adenine Dimethylase Domain Containing 1 | 29 |
| ENSG00000224163 |  | 4 |
| ANKRD61 | Ankyrin Repeat Domain 61 | 27 |
| SPPL2A | Signal Peptide Peptidase Like 2A | 38 |
| ENSG00000267002 |  | 6 |
| ENSG00000231344 |  | 5 |
| NONHSAG038451.2 |  | 4 |
| SKAP1 | Src Kinase Associated Phosphoprotein 1 | 40 |
| PCDH20 | Protocadherin 20 | 33 |
| TMEM170A | Transmembrane Protein 170A | 33 |
| ZNF574 | Zinc Finger Protein 574 | 32 |
| NT5DC3 | 5'-Nucleotidase Domain Containing 3 | 33 |
| INTS6 | Integrator Complex Subunit 6 | 37 |
| ATP2B1-AS1 | ATP2B1 Antisense RNA 1 | 12 |
| ST8SIA1 | ST8 Alpha-N-Acetyl-Neuraminide Alpha-2,8-Sialyltransferase 1 | 41 |
| RAB20 | RAB20, Member RAS Oncogene Family | 35 |
| MIR6809 | MicroRNA 6809 | 9 |
| GFOD2 | Glucose-Fructose Oxidoreductase Domain Containing 2 | 35 |
| SMG8 | SMG8 Nonsense Mediated MRNA Decay Factor | 34 |
| RPL36AP37 | Ribosomal Protein L36a Pseudogene 37 | 8 |
| LINC01254 | Long Intergenic Non-Protein Coding RNA 1254 | 12 |
| SAPCD1-AS1 | SAPCD1 Antisense RNA 1 | 13 |
| RAP1GDS1 | Rap1 GTPase-GDP Dissociation Stimulator 1 | 41 |
| UNC5B-AS1 | UNC5B Antisense RNA 1 | 12 |
| RGS8 | Regulator Of G Protein Signaling 8 | 37 |
| ARL4C | ADP Ribosylation Factor Like GTPase 4C | 34 |
| ZACN | Zinc Activated Ion Channel | 32 |
| WDR55 | WD Repeat Domain 55 | 32 |
| CRYL1 | Crystallin Lambda 1 | 38 |
| RGS12 | Regulator Of G Protein Signaling 12 | 39 |
| TLCD4 | TLC Domain Containing 4 | 27 |
| HDHD5-AS1 | HDHD5 Antisense RNA 1 | 12 |
| ELF4 | E74 Like ETS Transcription Factor 4 | 40 |
| RSBN1 | Round Spermatid Basic Protein 1 | 33 |
| FNDC4 | Fibronectin Type III Domain Containing 4 | 35 |
| ENSG00000225472 |  | 8 |
| NONHSAG045678.2-003 |  | 4 |
| APOBEC1 | Apolipoprotein B MRNA Editing Enzyme Catalytic Subunit 1 | 39 |
| LOXL1-AS1 | LOXL1 Antisense RNA 1 | 16 |
| ADAMTSL4-AS2 | ADAMTSL4 Antisense RNA 2 | 9 |
| OASL | 2'-5'-Oligoadenylate Synthetase Like | 40 |
| LINC02702 | Long Intergenic Non-Protein Coding RNA 2702 | 8 |
| FKBP7 | FKBP Prolyl Isomerase 7 | 36 |
| UBE2F | Ubiquitin Conjugating Enzyme E2 F (Putative) | 42 |
| MAP1LC3B | Microtubule Associated Protein 1 Light Chain 3 Beta | 43 |
| ARHGAP20 | Rho GTPase Activating Protein 20 | 36 |
| HEATR5B | HEAT Repeat Containing 5B | 32 |
| ATP5MF | ATP Synthase Membrane Subunit F | 29 |
| RN7SL5P | RNA, 7SL, Cytoplasmic 5, Pseudogene | 7 |
| TMEM241 | Transmembrane Protein 241 | 31 |
| ARHGAP42-AS1 | ARHGAP42 Antisense RNA 1 | 8 |
| lnc-KANK3-3 |  | 4 |
| SINHCAF | SIN3-HDAC Complex Associated Factor | 26 |
| GTSF1 | Gametocyte Specific Factor 1 | 34 |
| AOC4P | Amine Oxidase Copper Containing 4, Pseudogene | 10 |
| GSTCD | Glutathione S-Transferase C-Terminal Domain Containing | 35 |
| SFT2D3 | SFT2 Domain Containing 3 | 29 |
| LINC01535 | Long Intergenic Non-Protein Coding RNA 1535 | 12 |
| SNORD61 | Small Nucleolar RNA, C/D Box 61 | 16 |
| ZNF594 | Zinc Finger Protein 594 | 29 |
| PFN3 | Profilin 3 | 32 |
| ZNF652 | Zinc Finger Protein 652 | 36 |
| SPNS3 | Sphingolipid Transporter 3 (Putative) | 33 |
| SETD9 | SET Domain Containing 9 | 32 |
| ENSG00000241456 |  | 7 |
| ZNF22 | Zinc Finger Protein 22 | 35 |
| RPS23P3 | Ribosomal Protein S23 Pseudogene 3 | 5 |
| ENSG00000271821 |  | 5 |
| ENSG00000255726 |  | 4 |
| piR-56320 |  | 3 |
| piR-45578 |  | 2 |
| ENSG00000220030 |  | 2 |
| ENSG00000243635 |  | 1 |
| ENSG00000261879 |  | 6 |
| ABHD15-AS1 | ABHD15 Antisense RNA 1 | 9 |
| CEP170B | Centrosomal Protein 170B | 32 |
| ZNF606 | Zinc Finger Protein 606 | 32 |
| RTF1 | RTF1 Homolog, Paf1/RNA Polymerase II Complex Component | 36 |
| GBP5 | Guanylate Binding Protein 5 | 37 |
| GDPD5 | Glycerophosphodiester Phosphodiesterase Domain Containing 5 | 36 |
| CES1P1 | Carboxylesterase 1 Pseudogene 1 | 22 |
| EFCAB1 | EF-Hand Calcium Binding Domain 1 | 33 |
| TOMM7 | Translocase Of Outer Mitochondrial Membrane 7 | 36 |
| MTARC1 | Mitochondrial Amidoxime Reducing Component 1 | 28 |
| TMEM9B-AS1 | TMEM9B Antisense RNA 1 | 14 |
| FRGCA | FOXM1-Regulated, Gastric Cancer Associated | 12 |
| lnc-RAB20-2 |  | 4 |
| ENSG00000279237 |  | 4 |
| ENSG00000286371 |  | 2 |
| LINC01080 | Long Intergenic Non-Protein Coding RNA 1080 | 13 |
| LINC00310 | Long Intergenic Non-Protein Coding RNA 310 | 16 |
| ATP6V1C2 | ATPase H+ Transporting V1 Subunit C2 | 41 |
| CRCP | CGRP Receptor Component | 37 |
| PSMG2 | Proteasome Assembly Chaperone 2 | 33 |
| INHBC | Inhibin Subunit Beta C | 37 |
| SLC17A4 | Solute Carrier Family 17 Member 4 | 36 |
| RGS10 | Regulator Of G Protein Signaling 10 | 43 |
| VWA1 | Von Willebrand Factor A Domain Containing 1 | 35 |
| MMP24OS | MMP24 Opposite Strand | 13 |
| C1orf109 | Chromosome 1 Open Reading Frame 109 | 33 |
| TRIM6 | Tripartite Motif Containing 6 | 35 |
| DNM1P35 | Dynamin 1 Pseudogene 35 | 12 |
| ENSG00000203392 |  | 8 |
| ADAMTS7P4 | ADAMTS7 Pseudogene 4 | 7 |
| ENSG00000234936 |  | 7 |
| RNU6-958P | RNA, U6 Small Nuclear 958, Pseudogene | 6 |
| PARP12 | Poly(ADP-Ribose) Polymerase Family Member 12 | 37 |
| FLYWCH1 | FLYWCH-Type Zinc Finger 1 | 33 |
| PJA1 | Praja Ring Finger Ubiquitin Ligase 1 | 39 |
| MIR616 | MicroRNA 616 | 17 |
| RCN3 | Reticulocalbin 3 | 34 |
| DAGLB | Diacylglycerol Lipase Beta | 39 |
| WDR86 | WD Repeat Domain 86 | 31 |
| KRT8P26 | Keratin 8 Pseudogene 26 | 9 |
| PKN2-AS1 | PKN2 Antisense RNA 1 | 13 |
| CLPTM1L | CLPTM1 Like | 36 |
| HSF2BP | Heat Shock Transcription Factor 2 Binding Protein | 33 |
| CRYZ | Crystallin Zeta | 43 |
| ZNF177 | Zinc Finger Protein 177 | 32 |
| STK16 | Serine/Threonine Kinase 16 | 40 |
| ENSG00000219702 |  | 4 |
| IL20RB | Interleukin 20 Receptor Subunit Beta | 37 |
| RPL39P10 | Ribosomal Protein L39 Pseudogene 10 | 4 |
| KRT18P34 | Keratin 18 Pseudogene 34 | 9 |
| RAB11FIP3 | RAB11 Family Interacting Protein 3 | 37 |
| TRAF4 | TNF Receptor Associated Factor 4 | 43 |
| LENG1 | Leukocyte Receptor Cluster Member 1 | 31 |
| DIP2A | Disco Interacting Protein 2 Homolog A | 39 |
| QRFPR | Pyroglutamylated RFamide Peptide Receptor | 36 |
| RPL35AP15 | Ribosomal Protein L35a Pseudogene 15 | 5 |
| RNPEPL1 | Arginyl Aminopeptidase Like 1 | 36 |
| RAP1GAP2 | RAP1 GTPase Activating Protein 2 | 33 |
| FBRSL1 | Fibrosin Like 1 | 32 |
| RPS6KL1 | Ribosomal Protein S6 Kinase Like 1 | 34 |
| EXOC3L1 | Exocyst Complex Component 3 Like 1 | 32 |
| ENSG00000259407 |  | 7 |
| ENSG00000259630 |  | 4 |
| MF281438-018 |  | 3 |
| piR-59153 |  | 3 |
| CASTOR1 | Cytosolic Arginine Sensor For MTORC1 Subunit 1 | 24 |
| ANKRD13B | Ankyrin Repeat Domain 13B | 33 |
| MAMSTR | MEF2 Activating Motif And SAP Domain Containing Transcriptional Regulator | 31 |
| FCHO1 | FCH And Mu Domain Containing Endocytic Adaptor 1 | 35 |
| SLC35E4 | Solute Carrier Family 35 Member E4 | 31 |
| PHTF1 | Putative Homeodomain Transcription Factor 1 | 35 |
| ENHO | Energy Homeostasis Associated | 30 |
| RPL15P2 | Ribosomal Protein L15 Pseudogene 2 | 7 |
| ASTN1 | Astrotactin 1 | 35 |
| ZBTB42 | Zinc Finger And BTB Domain Containing 42 | 36 |
| IRX2 | Iroquois Homeobox 2 | 36 |
| GABRR1 | Gamma-Aminobutyric Acid Type A Receptor Subunit Rho1 | 41 |
| ATXN2-AS | ATXN2 Antisense RNA | 10 |
| LINC01526 | Long Intergenic Non-Protein Coding RNA 1526 | 10 |
| lnc-CALCRL-2 |  | 5 |
| ENSG00000287190 |  | 4 |
| ENSG00000288016 |  | 3 |
| ENSG00000285963 |  | 2 |
| HSALNG0094047 |  | 2 |
| PVRIG | PVR Related Immunoglobulin Domain Containing | 31 |
| RPL35P3 | Ribosomal Protein L35 Pseudogene 3 | 6 |
| CLECL1 | C-Type Lectin Like 1 | 31 |
| RPL21P108 | Ribosomal Protein L21 Pseudogene 108 | 5 |
| CABLES2 | Cdk5 And Abl Enzyme Substrate 2 | 34 |
| TSPAN6 | Tetraspanin 6 | 37 |
| ZFYVE28 | Zinc Finger FYVE-Type Containing 28 | 35 |
| ENSG00000248537 |  | 6 |
| L13712-004 |  | 4 |
| LINC02577 | Long Intergenic Non-Protein Coding RNA 2577 | 9 |
| PODN | Podocan | 36 |
| LINC00479 | Long Intergenic Non-Protein Coding RNA 479 | 21 |
| OSCAR | Osteoclast Associated Ig-Like Receptor | 38 |
| UBA52P1 | Ubiquitin A-52 Residue Ribosomal Protein Fusion Product 1 Pseudogene 1 | 6 |
| SLC46A3 | Solute Carrier Family 46 Member 3 | 36 |
| LINC01554 | Long Intergenic Non-Protein Coding RNA 1554 | 21 |
| MN298114-130 |  | 4 |
| ENSG00000187812 |  | 4 |
| FETUB | Fetuin B | 36 |
| MIR3605 | MicroRNA 3605 | 15 |
| OIP5-AS1 | OIP5 Antisense RNA 1 | 17 |
| ATP13A1 | ATPase 13A1 | 39 |
| POLR1B | RNA Polymerase I Subunit B | 40 |
| TET1P1 | Tet Methylcytosine Dioxygenase 1 Pseudogene 1 | 7 |
| SNORD117 | Small Nucleolar RNA, C/D Box 117 | 16 |
| ZNF572 | Zinc Finger Protein 572 | 31 |
| SF3A3 | Splicing Factor 3a Subunit 3 | 37 |
| DPP8 | Dipeptidyl Peptidase 8 | 37 |
| BCCIP | BRCA2 And CDKN1A Interacting Protein | 36 |
| FAIM | Fas Apoptotic Inhibitory Molecule | 39 |
| NAGK | N-Acetylglucosamine Kinase | 43 |
| MSBP2 | Minisatellite Binding Protein 2 | 4 |
| FAT1P1 | FAT Atypical Cadherin 1 Pseudogene 1 | 8 |
| GARS1-DT | GARS1 Divergent Transcript | 12 |
| C4orf51 | Chromosome 4 Open Reading Frame 51 | 24 |
| RPL15P22 | Ribosomal Protein L15 Pseudogene 22 | 5 |
| ZNF678 | Zinc Finger Protein 678 | 29 |
| ZNF217 | Zinc Finger Protein 217 | 39 |
| STEAP1B | STEAP Family Member 1B | 25 |
| LINC01626 | Long Intergenic Non-Protein Coding RNA 1626 | 10 |
| DOCK5 | Dedicator Of Cytokinesis 5 | 37 |
| ENSG00000264007 |  | 6 |
| C14orf180 | Chromosome 14 Open Reading Frame 180 | 28 |
| SNORD123 | Small Nucleolar RNA, C/D Box 123 | 13 |
| SLC35E2A | Solute Carrier Family 35 Member E2A | 24 |
| ENSG00000272072 |  | 6 |
| MIR628 | MicroRNA 628 | 17 |
| MXRA7 | Matrix Remodeling Associated 7 | 31 |
| MIR1272 | MicroRNA 1272 | 13 |
| PRKCE-AS1 | PRKCE Antisense RNA 1 | 7 |
| RPS7P8 | Ribosomal Protein S7 Pseudogene 8 | 5 |
| MIR661 | MicroRNA 661 | 17 |
| RPL23P4 | Ribosomal Protein L23 Pseudogene 4 | 7 |
| LOC110673971 | CYP11B2 Promoter | 1 |
| PGLYRP2 | Peptidoglycan Recognition Protein 2 | 36 |
| TRABD2B | TraB Domain Containing 2B | 25 |
| PARP9 | Poly(ADP-Ribose) Polymerase Family Member 9 | 39 |
| piR-33458 |  | 4 |
| CCDC68 | Coiled-Coil Domain Containing 68 | 36 |
| SKOR2 | SKI Family Transcriptional Corepressor 2 | 30 |
| C1QTNF2 | C1q And TNF Related 2 | 36 |
| OSBPL7 | Oxysterol Binding Protein Like 7 | 37 |
| PHLDB1 | Pleckstrin Homology Like Domain Family B Member 1 | 37 |
| LSM3 | LSM3 Homolog, U6 Small Nuclear RNA And MRNA Degradation Associated | 39 |
| SERTAD4 | SERTA Domain Containing 4 | 31 |
| MAGOHB | Mago Homolog B, Exon Junction Complex Subunit | 36 |
| MCRS1 | Microspherule Protein 1 | 37 |
| CEACAM21 | CEA Cell Adhesion Molecule 21 | 32 |
| LINC00578 | Long Intergenic Non-Protein Coding RNA 578 | 13 |
| PLCH1 | Phospholipase C Eta 1 | 38 |
| MT1X | Metallothionein 1X | 40 |
| IFNWP5 | Interferon Omega 1 Pseudogene 5 | 6 |
| SPRR3 | Small Proline Rich Protein 3 | 37 |
| CEACAM4 | CEA Cell Adhesion Molecule 4 | 32 |
| ERGIC3 | ERGIC And Golgi 3 | 36 |
| TARBP2 | TARBP2 Subunit Of RISC Loading Complex | 41 |
| NYNRIN | NYN Domain And Retroviral Integrase Containing | 34 |
| MICOS10 | Mitochondrial Contact Site And Cristae Organizing System Subunit 10 | 26 |
| NGEF | Neuronal Guanine Nucleotide Exchange Factor | 38 |
| RPL21P124 | Ribosomal Protein L21 Pseudogene 124 | 10 |
| PCIF1 | PDX1 C-Terminal Inhibiting Factor 1 | 32 |
| LRRIQ3 | Leucine Rich Repeats And IQ Motif Containing 3 | 31 |
| RAET1E | Retinoic Acid Early Transcript 1E | 38 |
| ANKRD31 | Ankyrin Repeat Domain 31 | 30 |
| MBLAC1 | Metallo-Beta-Lactamase Domain Containing 1 | 28 |
| HNRNPA1P16 | Heterogeneous Nuclear Ribonucleoprotein A1 Pseudogene 16 | 8 |
| ENSG00000263345 |  | 8 |
| RNU7-169P | RNA, U7 Small Nuclear 169 Pseudogene | 6 |
| L13712-013 |  | 4 |
| DTWD1 | DTW Domain Containing 1 | 33 |
| H2BC19P | H2B Clustered Histone 19, Pseudogene | 12 |
| ENSG00000250001 |  | 7 |
| LOC107985876 | Uncharacterized LOC107985876 | 6 |
| ENSG00000259314 |  | 6 |
| UBA52P6 | Ubiquitin A-52 Residue Ribosomal Protein Fusion Product 1 Pseudogene 6 | 9 |
| NUTM2E | NUT Family Member 2E | 16 |
| LINC00379 | Long Intergenic Non-Protein Coding RNA 379 | 12 |
| LINC00380 | Long Intergenic Non-Protein Coding RNA 380 | 10 |
| ENSG00000240354 |  | 6 |
| TRK-CTT2-1 | TRNA-Lys (Anticodon CTT) 2-1 | 6 |
| TRE-CTC1-1 | TRNA-Glu (Anticodon CTC) 1-1 | 6 |
| TRG-TCC2-1 | TRNA-Gly (Anticodon TCC) 2-1 | 6 |
| TRH-GTG1-1 | TRNA-His (Anticodon GTG) 1-1 | 6 |
| LINC01916 | Long Intergenic Non-Protein Coding RNA 1916 | 6 |
| MTND4P1 | MT-ND4 Pseudogene 1 | 5 |
| lnc-SHE-1 |  | 4 |
| LOC646934 | Golgin A6 Family Member D Pseudogene | 4 |
| lnc-SLC22A2-5 |  | 4 |
| lnc-HFE2-1 |  | 4 |
| ENSG00000274055 |  | 4 |
| lnc-ABCA12-7 |  | 4 |
| MN296862 |  | 3 |
| LOC105375883 | Uncharacterized LOC105375883 | 3 |
| KU881768 |  | 3 |
| piR-33123-002 |  | 2 |
| piR-57133-440 |  | 2 |
| piR-41195-086 |  | 2 |
| RF00017-314 |  | 2 |
| piR-33161-001 |  | 2 |
| piR-41525-685 |  | 2 |
| HSALNG0032588 |  | 2 |
| piR-53114-006 |  | 2 |
| piR-43066 |  | 2 |
| HSALNG0094030 |  | 2 |
| piR-36575-043 |  | 2 |
| piR-44984-001 |  | 2 |
| piR-46060-001 |  | 2 |
| piR-39017-001 |  | 2 |
| RF00017-1271 |  | 2 |
| ENSG00000273512 |  | 2 |
| lnc-ABO-1 |  | 2 |
| RNU6-850P | RNA, U6 Small Nuclear 850, Pseudogene | 7 |
| ENSG00000260461 |  | 6 |
| LOC101929633 | Uncharacterized LOC101929633 | 9 |
| GEM | GTP Binding Protein Overexpressed In Skeletal Muscle | 38 |
| MTMR9 | Myotubularin Related Protein 9 | 37 |
| HPCAL1 | Hippocalcin Like 1 | 41 |
| FCF1P2 | FCF1 Pseudogene 2 | 8 |
| ZBTB12 | Zinc Finger And BTB Domain Containing 12 | 34 |
| DCBLD1 | Discoidin, CUB And LCCL Domain Containing 1 | 35 |
| MIR194-1 | MicroRNA 194-1 | 16 |
| CD164L2 | CD164 Molecule Like 2 | 29 |
| GOLGA3 | Golgin A3 | 35 |
| ENSG00000222022 |  | 9 |
| RPL21P97 | Ribosomal Protein L21 Pseudogene 97 | 7 |
| POM121L9P | POM121 Transmembrane Nucleoporin Like 9, Pseudogene | 14 |
| MSH5-SAPCD1 | MSH5-SAPCD1 Readthrough (NMD Candidate) | 16 |
| C6orf62 | Chromosome 6 Open Reading Frame 62 | 32 |
| RPL21P41 | Ribosomal Protein L21 Pseudogene 41 | 6 |
| LIF-AS1 | LIF Antisense RNA 1 | 9 |
| ENSG00000240731 |  | 7 |
| ENSG00000259375 |  | 6 |
| ENSG00000227775 |  | 4 |
| lnc-PRMT8-3 |  | 4 |
| piR-57133-201 |  | 3 |
| NOSIP | Nitric Oxide Synthase Interacting Protein | 37 |
| CD200R1 | CD200 Receptor 1 | 40 |
| CGGBP1 | CGG Triplet Repeat Binding Protein 1 | 32 |
| MIR2909 | MicroRNA 2909 | 8 |
| RPL29P29 | Ribosomal Protein L29 Pseudogene 29 | 5 |
| SUSD5 | Sushi Domain Containing 5 | 33 |
| ENSG00000267174 |  | 7 |
| ZNF385C | Zinc Finger Protein 385C | 28 |
| CCNB2 | Cyclin B2 | 44 |
| ARHGEF10L | Rho Guanine Nucleotide Exchange Factor 10 Like | 36 |
| ZNF26 | Zinc Finger Protein 26 | 31 |
| SWSAP1 | SWIM-Type Zinc Finger 7 Associated Protein 1 | 28 |
| OR10J5 | Olfactory Receptor Family 10 Subfamily J Member 5 | 32 |
| SATB1-AS1 | SATB1 Antisense RNA 1 | 13 |
| ENSG00000250740 |  | 8 |
| SNHG9 | Small Nucleolar RNA Host Gene 9 | 16 |
| SERTAD4-AS1 | SERTAD4 Antisense RNA 1 | 18 |
| CYP2A13 | Cytochrome P450 Family 2 Subfamily A Member 13 | 41 |
| CHADL | Chondroadherin Like | 35 |
| TMEM184A | Transmembrane Protein 184A | 33 |
| LINC01311 | Long Intergenic Non-Protein Coding RNA 1311 | 13 |
| SEC14L2 | SEC14 Like Lipid Binding 2 | 39 |
| GCNT4 | Glucosaminyl (N-Acetyl) Transferase 4 | 35 |
| HDLC3 | High Density Lipoprotein Cholesterol, Low Serum, 3 | 2 |
| B4GALNT3 | Beta-1,4-N-Acetyl-Galactosaminyltransferase 3 | 38 |
| GPRC5B | G Protein-Coupled Receptor Class C Group 5 Member B | 38 |
| SCUBE2 | Signal Peptide, CUB Domain And EGF Like Domain Containing 2 | 39 |
| HMGN2P15 | High Mobility Group Nucleosomal Binding Domain 2 Pseudogene 15 | 8 |
| RF00017-7861 |  | 4 |
| RF00026-538 |  | 3 |
| L13714-342 |  | 3 |
| ADAMTSL4-AS1 | ADAMTSL4 Antisense RNA 1 | 17 |
| MIR939 | MicroRNA 939 | 16 |
| TMEM123 | Transmembrane Protein 123 | 33 |
| WDR31 | WD Repeat Domain 31 | 33 |
| AP1M2 | Adaptor Related Protein Complex 1 Subunit Mu 2 | 40 |
| piR-60314 |  | 4 |
| CAPS2 | Calcyphosine 2 | 33 |
| DUS4L | Dihydrouridine Synthase 4 Like | 36 |
| FAHD1 | Fumarylacetoacetate Hydrolase Domain Containing 1 | 37 |
| MEF2C-AS1 | MEF2C Antisense RNA 1 | 13 |
| LRRC63 | Leucine Rich Repeat Containing 63 | 31 |
| MYNN | Myoneurin | 37 |
| ENSG00000243797 |  | 8 |
| ENSG00000238387 |  | 7 |
| RF00017-5420 |  | 4 |
| SIGLECL1 | SIGLEC Family Like 1 | 29 |
| CYP3A7 | Cytochrome P450 Family 3 Subfamily A Member 7 | 43 |
| UNC5B | Unc-5 Netrin Receptor B | 37 |
| MIER3 | MIER Family Member 3 | 32 |
| CALCOCO1 | Calcium Binding And Coiled-Coil Domain 1 | 36 |
| PPT2-EGFL8 | PPT2-EGFL8 Readthrough (NMD Candidate) | 15 |
| TBCC | Tubulin Folding Cofactor C | 36 |
| SPDYE3 | Speedy/RINGO Cell Cycle Regulator Family Member E3 | 25 |
| BTG2 | BTG Anti-Proliferation Factor 2 | 37 |
| CPXM2 | Carboxypeptidase X, M14 Family Member 2 | 36 |
| TREML4 | Triggering Receptor Expressed On Myeloid Cells Like 4 | 31 |
| OR2F2 | Olfactory Receptor Family 2 Subfamily F Member 2 | 34 |
| RNU2-3P | RNA, U2 Small Nuclear 3, Pseudogene | 7 |
| TMEM150A | Transmembrane Protein 150A | 32 |
| APTR | Alu-Mediated CDKN1A/P21 Transcriptional Regulator | 15 |
| FPR3 | Formyl Peptide Receptor 3 | 40 |
| DOK3 | Docking Protein 3 | 36 |
| ENSG00000228363 |  | 8 |
| KLHL35 | Kelch Like Family Member 35 | 32 |
| ZNF717 | Zinc Finger Protein 717 | 31 |
| ZBTB5 | Zinc Finger And BTB Domain Containing 5 | 31 |
| SLC26A4-AS1 | SLC26A4 Antisense RNA 1 | 13 |
| SNORD15B | Small Nucleolar RNA, C/D Box 15B | 16 |
| MAST4-AS1 | MAST4 Antisense RNA 1 | 12 |
| ENSG00000257221 |  | 6 |
| lnc-ZFP36L2-3 |  | 4 |
| piR-34822-157 |  | 4 |
| ENSG00000220643 |  | 4 |
| piR-59804-019 |  | 4 |
| piR-53431-382 |  | 3 |
| FER1L4 | Fer-1 Like Family Member 4 (Pseudogene) | 22 |
| WSPAR | WNT Signaling Pathway Activating Non-Coding RNA | 11 |
| S1PR4 | Sphingosine-1-Phosphate Receptor 4 | 42 |
| OSBPL8 | Oxysterol Binding Protein Like 8 | 37 |
| RPS26P10 | Ribosomal Protein S26 Pseudogene 10 | 6 |
| CYCSP42 | CYCS Pseudogene 42 | 6 |
| C1orf35 | Chromosome 1 Open Reading Frame 35 | 31 |
| MIR4271 | MicroRNA 4271 | 10 |
| CHDH | Choline Dehydrogenase | 40 |
| C1QTNF9B | C1q And TNF Related 9B | 32 |
| ZNF140 | Zinc Finger Protein 140 | 35 |
| ABCA13 | ATP Binding Cassette Subfamily A Member 13 | 38 |
| MRPL55 | Mitochondrial Ribosomal Protein L55 | 33 |
| CTRB2 | Chymotrypsinogen B2 | 33 |
| LOC110366355 | Enhancer In Intron 10 Of CYP3A4 | 1 |
| POT1-AS1 | POT1 Antisense RNA 1 | 16 |
| SCEL | Sciellin | 36 |
| CYP2G1P | Cytochrome P450 Family 2 Subfamily G Member 1, Pseudogene | 18 |
| piR-32813-004 |  | 2 |
| ERCC6L | ERCC Excision Repair 6 Like, Spindle Assembly Checkpoint Helicase | 37 |
| C1QL3 | Complement C1q Like 3 | 33 |
| BLOC1S5-TXNDC5 | BLOC1S5-TXNDC5 Readthrough (NMD Candidate) | 18 |
| ENOSF1 | Enolase Superfamily Member 1 | 35 |
| MIR660 | MicroRNA 660 | 14 |
| CAPG | Capping Actin Protein, Gelsolin Like | 40 |
| CYCSP14 | CYCS Pseudogene 14 | 5 |
| piR-59241 |  | 4 |
| ENSG00000228351 |  | 6 |
| OR4A46P | Olfactory Receptor Family 4 Subfamily A Member 46 Pseudogene | 6 |
| C2orf68 | Chromosome 2 Open Reading Frame 68 | 29 |
| FGF13-AS1 | FGF13 Antisense RNA 1 | 13 |
| OVOS2 | Alpha-2-Macroglobulin Like 1 Pseudogene | 24 |
| RPS7P5 | Ribosomal Protein S7 Pseudogene 5 | 10 |
| LENG8 | Leukocyte Receptor Cluster Member 8 | 33 |
| RASL12 | RAS Like Family 12 | 36 |
| MRPS18AP1 | Mitochondrial Ribosomal Protein S18A Pseudogene 1 | 7 |
| ENSG00000254554 |  | 7 |
| ENSG00000267510 |  | 7 |
| ENSG00000258695 |  | 6 |
| RF00017-5469 |  | 4 |
| lnc-ALDH1A2-9 |  | 4 |
| C2orf16 | Chromosome 2 Open Reading Frame 16 | 28 |
| PSD4 | Pleckstrin And Sec7 Domain Containing 4 | 35 |
| SPATA12 | Spermatogenesis Associated 12 | 28 |
| GNG7 | G Protein Subunit Gamma 7 | 40 |
| CRISP2 | Cysteine Rich Secretory Protein 2 | 40 |
| HSDL2 | Hydroxysteroid Dehydrogenase Like 2 | 34 |
| FUT6 | Fucosyltransferase 6 | 41 |
| RTP5 | Receptor Transporter Protein 5 (Putative) | 27 |
| FAM167A-AS1 | FAM167A Antisense RNA 1 | 19 |
| HSPB9 | Heat Shock Protein Family B (Small) Member 9 | 29 |
| DNM1P46 | Dynamin 1 Pseudogene 46 | 20 |
| PRKCZ-AS1 | PRKCZ Antisense RNA 1 | 11 |
| KYAT3 | Kynurenine Aminotransferase 3 | 31 |
| TACC1P1 | Transforming Acidic Coiled-Coil Containing Protein 1 Pseudogene 1 | 6 |
| CABYR | Calcium Binding Tyrosine Phosphorylation Regulated | 35 |
| UBE3AP2 | Ubiquitin Protein Ligase E3A Pseudogene 2 | 8 |
| RPS27P1 | Ribosomal Protein S27 Pseudogene 1 | 7 |
| N4BP2L2 | NEDD4 Binding Protein 2 Like 2 | 34 |
| DDX18P1 | DEAD-Box Helicase 18 Pseudogene 1 | 8 |
| ENSG00000226599 |  | 6 |
| lnc-ATG9B-1 |  | 4 |
| NONHSAG029119.2 |  | 4 |
| piR-50437-157 |  | 3 |
| lnc-SLC10A7-7 |  | 2 |
| RF00017-1009 |  | 2 |
| RPLP2P2 | Ribosomal Protein Lateral Stalk Subunit P2 Pseudogene 2 | 5 |
| SEC14L3 | SEC14 Like Lipid Binding 3 | 36 |
| RBMXL1 | RBMX Like 1 | 29 |
| LRRC3C | Leucine Rich Repeat Containing 3C | 26 |
| C3orf20 | Chromosome 3 Open Reading Frame 20 | 33 |
| TMEM61 | Transmembrane Protein 61 | 28 |
| ENSG00000254810 |  | 7 |
| ENSG00000253398 |  | 6 |
| RPL13AP15 | Ribosomal Protein L13a Pseudogene 15 | 5 |
| MYADM | Myeloid Associated Differentiation Marker | 36 |
| FLJ12825 | Uncharacterized LOC440101 | 13 |
| SDR42E1 | Short Chain Dehydrogenase/Reductase Family 42E, Member 1 | 32 |
| ENSG00000249240 |  | 8 |
| LOC105378979 | Growth/Differentiation Factor 3 | 6 |
| lnc-TDRD15-6 |  | 4 |
| MN308699 |  | 4 |
| lnc-NAXD-2 |  | 4 |
| NONHSAG039225.2 |  | 3 |
| LOC105375696 | Uncharacterized LOC105375696 | 2 |
| KRTAP11-1 | Keratin Associated Protein 11-1 | 32 |
| TLCD4-RWDD3 | TLCD4-RWDD3 Readthrough | 16 |
| LINC02542 | Long Intergenic Non-Protein Coding RNA 2542 | 9 |
| GPR20 | G Protein-Coupled Receptor 20 | 35 |
| ACSS3 | Acyl-CoA Synthetase Short Chain Family Member 3 | 37 |
| GALR3 | Galanin Receptor 3 | 43 |
| ST6GALNAC5 | ST6 N-Acetylgalactosaminide Alpha-2,6-Sialyltransferase 5 | 38 |
| MROH5 | Maestro Heat Like Repeat Family Member 5 (Gene/Pseudogene) | 21 |
| SSPOP | SCO-Spondin, Pseudogene | 20 |
| JMY | Junction Mediating And Regulatory Protein, P53 Cofactor | 36 |
| LOC101928659 | Uncharacterized LOC101928659 | 10 |
| CICP26 | Capicua Transcriptional Repressor Pseudogene 26 | 7 |
| TMEM205 | Transmembrane Protein 205 | 31 |
| RPL31P4 | Ribosomal Protein L31 Pseudogene 4 | 8 |
| TOPAZ1 | Testis And Ovary Specific PAZ Domain Containing 1 | 28 |
[truncated: 32,669 more chars]
